# Analysis of Transcriptome Differences between Resistant and Susceptible Strains of the Citrus Red Mite *Panonychus citri* (Acari: Tetranychidae)

**DOI:** 10.1371/journal.pone.0028516

**Published:** 2011-12-05

**Authors:** Bin Liu, Gaofei Jiang, Yunfei Zhang, Junli Li, Xiaojiao Li, Jiansu Yue, Fei Chen, Haoqiang Liu, Hongjun Li, Shiping Zhu, Jinjun Wang, Chun Ran

**Affiliations:** 1 Key Laboratory of Horticulture Science for Southern Mountainous Regions, College of Horticulture and Landscape Architecture, Ministry of Education, Southwest University, Chongqing, People's Republic of China; 2 Citrus Research Institute, National Engineering Research Center for Citrus, Chinese Academy of Agricultural Sciences, Chongqing, People's Republic of China; 3 Key Laboratory of Entomology and Pest Control Engineering, College of Plant Protection, Southwest University, Chongqing, People's Republic of China; New Mexico State University, United States of America

## Abstract

**Background:**

The citrus red mite is a worldwide citrus pest and a common sensitizing allergen of asthma and rhinitis. It has developed strong resistance to many registered acaricides, However, the molecular mechanisms of resistance remain unknown. we therefore used next generation sequencing technology to investigate the global transcriptomes between resistant strains and susceptible strains.

**Results:**

We obtained 34,159, 30,466 and 32,217 unigenes by assembling the SS reads, RS reads and SS&RS reads respectively. There are total 17,581 annotated unigenes from SS&RS reads by BLAST searching databases of nr, the Clusters of Orthologous Groups (COGs) and Kyoto Encyclopedia of Genes and Genomes (KEGG) with an E-value ≤ 1e-5, in which 7,075 unigenes were annotated in the COG database, 12, 712 unigenes were found in the KEGG database and 3,812 unigenes were assigned to Gene ontology (GO). Moreover, 2,701 unigenes were judged to be the differentially expressed genes (DEGs) based on the uniquely mapped reads. There are 219 pathways in all annotated unigenes and 198 pathways in DEGs that mapped to the KEGG database. We identified 211 metabolism genes and target genes related to general insecticide resistance such as P450 and Cytochrome b, and further compared their differences between RS and SS. Meanwhile, we identified 105 and 194 genes related to growth and reproduction, respectively, based on the mode of action of Hexythiazox. After further analyses, we found variation in sequences but not in gene expression related to mite growth and reproduction between different strains.

**Conclusion:**

To our knowledge, this is the first comparative transcriptome study to discover candidate genes involved in phytophagous mite resistance. This study identified differential unigenes related to general pesticide resistance and organism growth and reproduction in *P*. *citri.* The assembled, annotated transcriptomes provide a valuable genomic resource for further understanding the molecular basis of resistance mechanisms.

## Introduction

The citrus red mite, *Panonychus citri* (McGregor), is a worldwide citrus pest causing a huge fruit yield loss every year. It has a short life cycle and high reproductive rate, infesting over 80 plant species such as citrus, rose, almond, pear, castor bean, and several broadleaf evergreen ornamentals [1]–[2]. It is also an important sensitizing allergen to asthma and rhinitis among people around citrus orchards [3]–[5]. Control of *P. citri* mainly depends on chemical applications, which lead to acaricide resistance in *P. citri*. Currently this situation is deteriorating because of the improper application of acaricides, leading to resistance to many registered acaricides [6]–[11].

Hexythiazox is a selective miticide that acts against various phytophagous mites. It has been widely used on various crops under integrated pest management programs, especially against citrus mites. However, phytophagous mites are capable of rapidly developing resistance to hexythiazox. A high level of resistance has already been reported in *Tetranychus urticae* (resistance factor > 1,000-fold) in Australia, in *P. citri* (resistance factor > 23,000-fold) in Japan, and in *Brevipalpus phoenicis* (resistance factor >10,000-fold) in Brazil [12]–[14]. Our previous study showed the resistance factor of *P. citri* increased 3532-fold after continuous selection with hexythiazox for 20 generations (Ran *et al*. unpublished data). Hexythiazox has little or no effect on survival of the adult mite [15], but interferes with its growth and reproduction [12]. Hexythiazox resistance may alter the target sites and increase the rate of metabolism. Studies on the resistance mechanism in *P. citri* have mainly focused on measuring the activity of detoxification enzymes [16]–[17]. Although its complete mitochondrial genome was sequenced recently [18]–[19], nuclear genome resources for *P. citri* are limited (only 234 records in NCBI in January 2011), which has severely hindered the study of resistance mechanisms at the molecular level.

A transcriptome is a complete set of transcripts in a cell or an organism. Various technologies have been developed to characterize transcriptomes, including serial analysis of gene expression (SAGE), cap analysis of gene expression (CAGE), massively parallel signature sequencing (MPSS) and RNA sequencing (RNA-Seq) [20]–[22]. Next generation sequencing technologies have dramatically accelerated genome-wide studies of transcriptomes and have been widely used to explore gene structure and gene expression even without a genome reference [23]–[27]. RNA-seq has made it increasingly possible to perform *de novo* transcriptome sequencing with the development of short read sequencing technologies such as the Roche 454, SOLiD and Solexa/Illumina platforms for various purposes [20], [28]–[29]. Illumina sequencing technology, generating large-scale sequence at a lower cost and greater time-saving, has recently been applied to transcriptome analysis of insects, such as *Aedes aegypti, Anopheles funestus*, *Bemisia tabaci*, *Nilaparvata lugens* and *Locusta migratoria* [30]–[34], but not of mites.

To gain detailed genetic information for resistance mechanism, we sequenced the transcriptomes of both a resistant strain (RS) and a susceptible strain (SS) of *P. citri* using a high-throughput sequencing platform – Illumina HiSeq™ 2000. We obtained over four billion bases of high-quality DNA sequence with an average read length of 90 bp. These sequences were assembled into 30,466 and 34,159 unigenes in the resistant and susceptible strains respectively. We then merged the above two groups of unigenes to produce longer unigenes; approximately 55% of these unigenes were already annotated, as identified by BLAST searches (E-value ≤ 1e-5) against the SwissProt (http://expasy.org/tools/blast), KEGG (http://www.genome.jp/kegg), COG (http://www.ncbi.nlm.nih.gov/COG/), nr (http://blast.ncbi.nlm.nih.gov/Blast.cgi) and GO databases. We systematically examined differential gene expression in order to identify genes involved in general insecticide resistance. Furthermore, we developed a new method to compare more than 200 genes involved in growth and reproduction, based on the mode of action of hexythiazox. The assembled, annotated transcriptome sequences provide a valuable genomic resource for further understanding the molecular basis of resistance.

## Results and Discussion

### Illumina sequencing and reads assembly

In order to uncover the differences in *P. citri* gene expression between the resistant strains (RS) and susceptible strains (SS), a pooled cDNA sample from each strain, representing eggs, larvae and adults, was sequenced with the Illumina sequencing platform. 25 and 26 million clean reads of 90 bp (EMBL-EBI accession number: ERP000885) were obtained from RS and SS, respectively, after filtering out the dirty raw reads. To facilitate sequence assembly, the remaining clean raw reads were clipped into 21-mers randomly using SOAPdenovo software [35], and used to assemble 215,641 contigs with a mean length of 178 bp in RS, and 282,291 contigs with an average length of 151 bp in SS (Table 1). With paired-end read joining and gap-filling, the above contigs were further assembled into 38,178 scaffolds with a mean size of 453 bp in RS and 44,017 scaffolds with a mean size 406 bp in SS, where the number of contigs with a scaffold size of more than 1000 bp is 4,643 in RS and 4,270 in SS. With gap-filling and long sequence clustering, the scaffolds were assembled into 30,466 RS-unigenes with a mean length of 536 bp and 34,159 SS-unigenes with a mean length of 489 bp. In addition, using the same strategy we obtained 32,217 All-unigenes with a mean size of 631 bp from the RS clean reads (25 million) and SS reads (26 million) combined (All-unigene originated from both RS and SS reads; RS-unigene from RS reads; SS-unigene from SS reads.).

**Figure 1:**
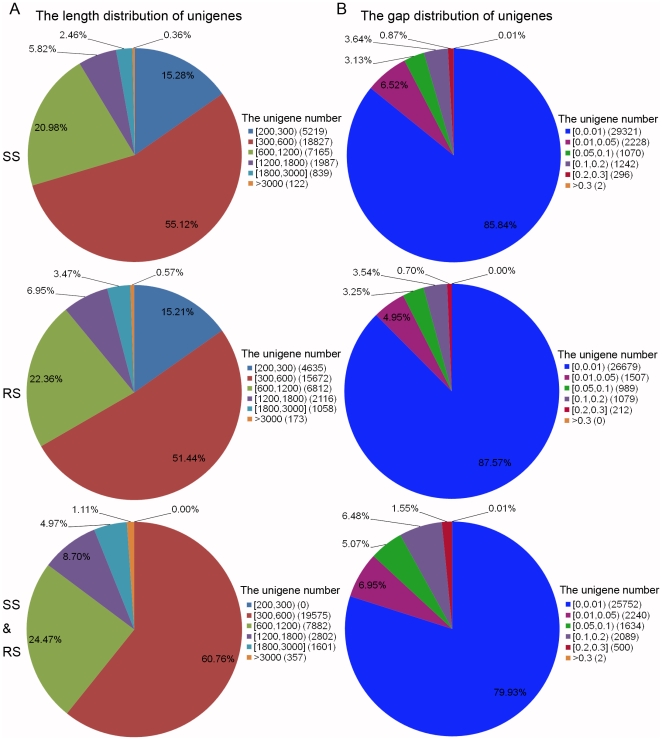
The length and gap distribution of unigenes after *de novo* assembly using the reads from SS, RS and SS&RS samples. A: Length distribution of unigenes. Numbers in square brackets (e.g., [200,300) and [1800,3000]) indicate the range of unigene length, while ‘>3000’ indicates unigenes longer than 3,000 bp. Numbers in parentheses (e.g., (5219) and (18827)) indicate the total number of unigenes falling in this length range. B: Gap distribution of unigenes. Values in square brackets indicate the ratio of the *N* number and all nucleotide base pair number for one unigene. Numbers in parentheses (e.g., (29321) and (2228)) indicate the number of unigenes that lie in the gap ratio (e.g., [0, 0.01) and [0.01, 0.05)).

The sequence of unigene (the unique) represents that they cannot be extended on both ends and contains the least Ns in this paper. The length distribution and gap distribution of unigenes from the three combinations (RS, SS and RS&SS) are shown in Figure 1. RS-unigenes and SS-unigenes had a similar distribution in length, with 300-600-bp sequences representing the highest proportion, followed by 600-1,200-bp sequences. Interestingly, RS&SS-unigenes dramatically improved the length distribution with no more size of 200–300 bp region, and instead of the minimum of 300 bp compared to RS-unigenes or SS-unigenes. The gap distribution, however, had a slightly different tendency, showing more Ns compared to RS-unigenes or SS-unigenes at the same level (Figure 1). In total, All-unigenes are better than the other two kinds of unigenes in quality, and obtained higher identity scores when using BLASTx to search against the non-redundant (nr) NCBI nucleotide database. To validate the quality of sequencing data, 21 unigenes from RS-, SS- and All-unigenes were randomly selected, for which 21 pairs of primers were designed, respectively, for RT-PCR amplification. In this experiment, 18 out of 21 primer pairs resulted in an expected band size and were further confirmed by Sanger sequencing (data not shown).

**Table 1:** Summary of reads in Susceptible Strains (SS) and Resistant Strains (RS) of Citrus red mite transcriptomes.

	SS	RS	SS&RS
Total base pairs (bp)	2,414,000,340	2,336,000,220	-
Total number of Reads	26,822,226	25,955,558	-
GC percentage	42.51%	41.93%	-
Total number of contigs	282,291	215,641	-
Mean length of contigs (bp)	151	178	-
Total number of scaffolds	44,017	38,178	-
Mean length of scaffolds (bp)	406	453	-
The number of unigenes	34,159	30,466	-
Mean length of unigenes	489	536	-
The number of all-unigenes	-	-	32,217
Mean length of all-unigenes	-	**-**	631

### Gene ontology (GO) and clusters of orthologous groups (COGs) classification

**Figure 2:**
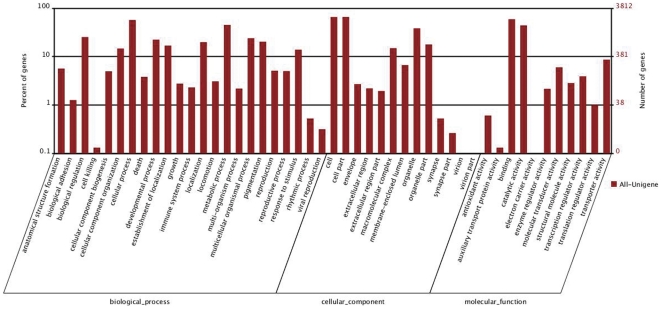
Histogram representation of Gene Ontology classification. GO categories, shown in the *x*-axis, are grouped into three main ontologies: biological process, cellular component and molecular function. The right *y*-axis indicates the number of genes in each category, while the left *y*-axis indicates the percentage of of total genes in that category. The ‘All-unigene’ indicates that the unigenes were those assembled from reads from the SS&RS sample.

**Figure 3:**
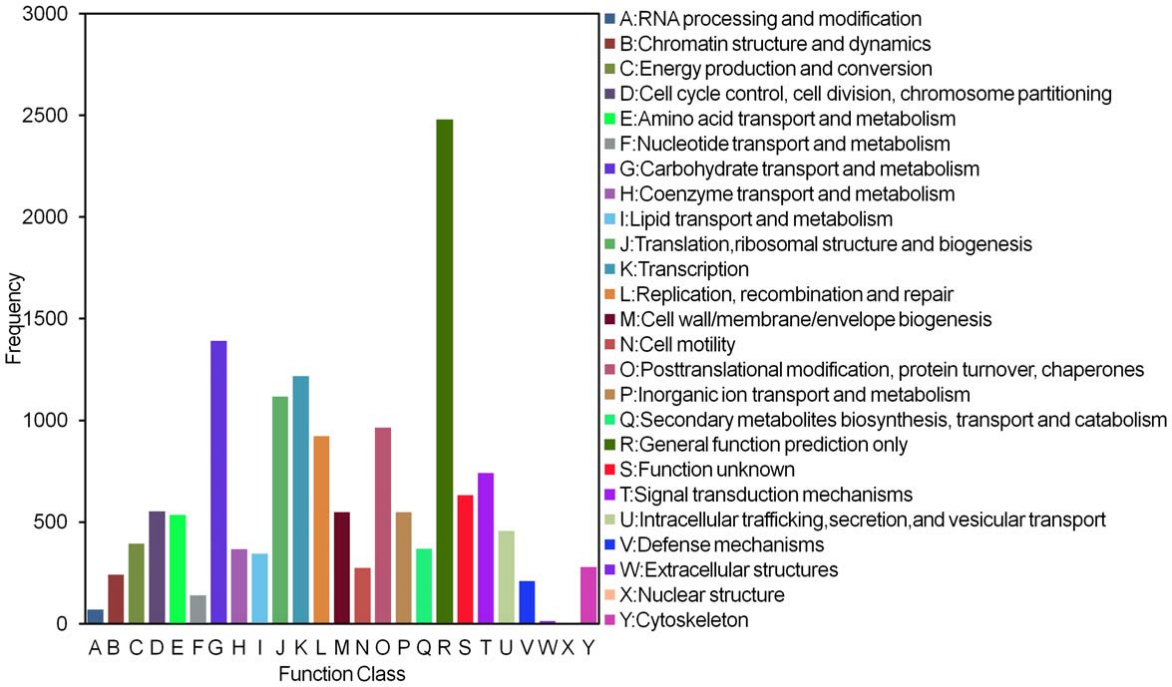
Histogram representation of clusters of orthologous groups (COG) classification. 7,075unigenes were assigned to 25 categories in the COG classification. The *y*-axis ‘Frequency’ indicates the number of genes in a specific function cluster. The right legend shows a description of the 25 function categories.

After functional annotation of unigenes (data not shown), the predicted *P. citri* genes were further classified by GO assignments. Based on homologous genes, 3,812 unigenes (Figure S1) from All-unigenes were categorized into 47 GO terms consisting of three domains: biological process, cellular component and molecular function (Figure 2). It was clear that the dominant distributions are from ‘Cellular process’, ‘Cell’, ‘Cell part’ and ‘Binding’ terms. We also observed a high percentage of genes assigned to ‘Biological regulation’, ‘Metabolic process’, ‘Organelle’, ‘Catalytic activity’ and a few genes assigned to ‘Cell killing’, ‘Synapse part’ and ‘Auxiliary transport protein activity’. In addition, we noticed some genes assigned to ‘Growth’, ‘Reproduction’, and ‘Developmental process’. However, no genes assigned to “Vinion”, “Vinior part” or “Electron carrier activity” were identified in this study (Fig. 2). To examine further the integrality of our transcriptome library and effectiveness of the annotation process, we calculated the unigene (with nr hits) numbers with COG classification. In total, there were 7,075 unigenes (Figure S1) identified from All-unigenes with a COG classification (Figure 3). Among the 25 COG categories, the cluster of ‘general function prediction’ occupied the highest number (2,480, 17%) followed by ‘Carbohydrate transport and metabolism’ (1,392, 9.4%), ‘Transcription’ (1,219, 8.2%) and ‘Translation, ribosomal structure and biogenesis’ (1,119, 7.5%). The categories of ‘Nuclear structure’ (4, 0.03%), ‘Extracellular structures’ (15, 0.1%) and ‘RNA processing and modification’ (71, 0.48%) had the fewest responding genes (Figure 3). To annotate further all-unigenes, we performed BLAST searches for all of them against the nr database of NCBI and obtained 17,457 unigenes, of which 7053 were present in the COG database (Figure S1).

### Network of Unigene

To investigate further biological behavior, we analyzed the biological pathways that are active in our samples. 32217 All-unigenes were mapped to the reference canonical pathways in the Kyoto Encyclopedia of Genes and Genomes (KEGG) [36] and 12712 of them obtained KEGG annotation, in which 102 unigenes were exclusively in the KEGG database (Figure S1). Further analyses found that 12,663 unigenes were assigned to the 219 KEGG pathways. There was a large number of unigenes restricted to only a single one pathway, such as metabolism pathway (1,983 unigenes), cancer pathway (623 unigenes) and lysosome pathway (477 unigenes). In constrast, we also found one unigene in one pathway, for example, polyketide sugar unit biosynthesis pathway (only one unigene), caffeine metabolism pathway (only one unigene). In terms of all above analyses, we had 17,581 unigenes annotated in the citrus red mite transcriptome (Figure S1).

### Differential expression and pathway analyses in RS and SS

**Figure 4:**
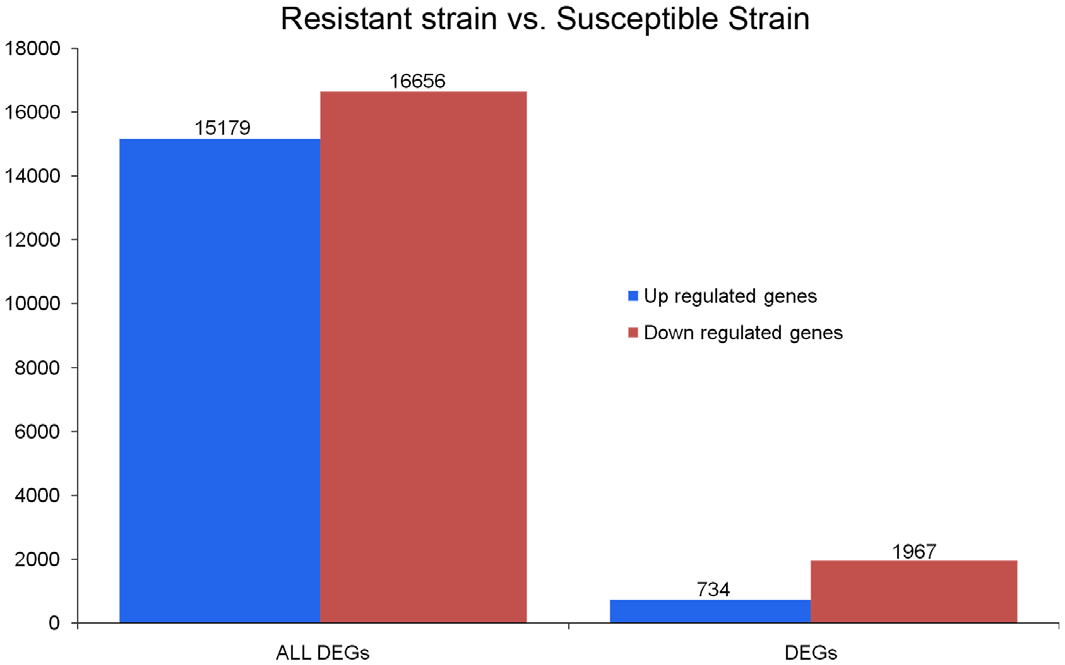
Differences in gene expression profile between the Resistant Strain and Susceptible Strain. ‘ALL DEGs’ indicates those unigenes showing differential expression between the two samples, while ‘DEGs’ indicates those unigenes with FDR ≤ 0.001 and |log_2_Ratio| ≥ 1. The numbers of genes up-regulated and down-regulated in the Resistant Strain relative to the Susceptible Strain are indicated above the blue or red bars, respectively.

**Figure 5:**
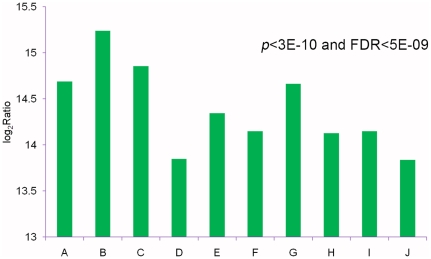
Analysis of expression levels of the top ten up-regulated transcripts between the two samples (RS vs. SS). The *y*-axis indicate the fold-changes of up-regulated genes. Categories in the *x*-axis represent genes homologous to (A) ‘heme/copper-type cytochrome/quinol oxidases, subunit 1’, (B) ‘F_0_F_1_-type ATP synthase, subunit a’, (C) ‘heme/copper-type cytochrome/quinol oxidases, subunit 2’, (D) ‘cytochrome b subunit of the bc complex’, (F) ‘heme/copper-type cytochrome/quinol oxidase, subunit 3’ and (J) ‘NADH: ubiquinone oxidoreductase subunit 1 (chain H)’,respectively. The remaining genes (E, G, H, I) had no functional annotation.

We developed a rigorous algorithm (see Methods) to identify genes differentially expressed between RS and SS by referring to “The significance of digital gene expression profiles” [37]. To explore further the gene expression levels, the Reads Per kb per Million reads (RPKM) method [38] was adapted to eliminate the influence of variation in gene length and the total reads number. 15,179 unigenes were up-regulated and 16,656 were down-regulated (Figure 4). Furthermore, the significance of gene expression differences was judged by using the threshold of false discovery rate (FDR ≤ 0.001) and the absolute value of log_2_Ratio (≥ 1). 2,701 significantly differentially expressed genes were obtained between the two samples, including 1,967 down-regulated and 734 up-regulated genes (Figure 4). The number of down-regulated genes was more than 2-fold that of up-regulated genes, which might be consistent with the observation that hexythiazox-poisoned individuals of *P. citri* are less active. To investigate further the expression difference, we examined the top ten up-regulated transcripts (Figure 5), in which the most up-regulated unigene (up to 15.24-fold) is homologous to F_0_F_1_-type ATP synthase.

To explore the biological function of the significantly differentially expressed genes, 2,701 of these were mapped to the KEGG database, and then enriched to important pathways such as metabolism and signal transduction based on the whole transcriptome background. There were 1,038 unigenes mapped to 198 pathways. Remarkably, specific pathways were observed that are involved in energy and reproduction, such as glycolysis, pentose phosphate pathway, TCA cycle, and cell cycle. Interestingly, all genes mapping to the above four pathways were dramatically down-regulated in the resistant strain. In contrast, genes involved in drug metabolism and steroid hormone biosynthesis pathways were substantially up-regulated, which may have biological relevance for survival under the selection of the pesticide for *P. citri*. Interestingly, we also noticed differentially expressed genes implicated in many disease pathways such as Parkinson’s disease, Huntington's disease, prion diseases and autoimmune thyroid disease.

### Transcripts related to general pesticide detoxification and targets

To date, many genes involved in general insecticide resistance have been identified. Little is known as to whether these resistance genes are related to the resistance of phytophagous mites, such as *P. citri*, to special acaricides. We therefore blasted *P. citri* transcriptomes against the NCBI nucleotide database and obtained many genes related to general pesticide resistance in other insect species.

**Figure 6:**
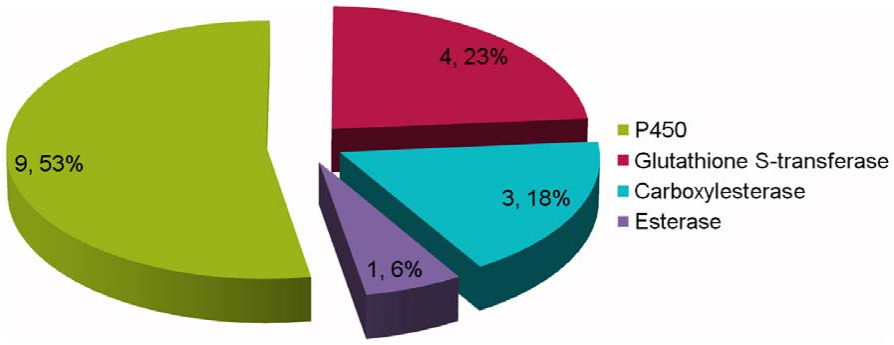
Pie chart representation of several genes, mainly implicated in insecticide metabolism. The number preceding the each percentage indicates the number of unigenes encoding a specific metabolism enzyme with log_2_Ratio greater than 2. The percentages indicate the propotion of each specific unigenes in four kinds of known enzyme unigenes.

Numerous transcripts identified in the *P. citri* transcriptomes are homologous to genes encoding enzymes with previously identified roles in general pesticide detoxification. In total, there were 121, 30, 43, 2 and 4 unigenes related to cytochrome P450 monoxygenases (CYPs), Glutathione S-transferase (GSTs), Carboxylesterase, NADH dehydrogenase and Superoxide dismutase (SODs), respectively. We only selected 46 of the P450s for functional annotation according to their degree of sequence matching (E-value < le-5) (Table 2), owing to the low sequence similarity of other P450s. In addition, only a few unigenes have been sequenced so far, except for NADH dehydrogenases, in the mitochondrial genome of *P. citri* [18]–[19]. We found 17 genes including 9 of P450s, 4 GSTs, 3 carboxylesterases and 1 esterase that were greater than value of 2 based on a log2ratio formula (Figure 6). Particularly, 4 of the P450s belonging to the CYP3 clade (which includes CYP3, CYP6 and CYP9 members) were annotated to the xenobiotic metabolism pathway (ko00982 and ko00980), and are related to many general pesticides such as deltamethrin, permethrin, dichlorodiphenyltrichloroethane (DDT) and Imidacloprid [39]–[43]. One up-regulated GST was annotated to the xenobiotic metabolism pathway (ko00980). These data support the notion that the P450s, GSTs, and others, are likely to be involved in hexythiazox resistance in *P. citri*.

**Table 2:** Genes involved in insecticide metabolism.

Candidate gene name	Number of sequences with nr^1^ hit	Number of known sequences from RefSeq^2^
Cytochrome P450	46	2
Glutathione S-transferase	30	0
Carboxylesterase	43	0
Catalase	14	0
Superoxide dismutase	4	0
NADH dehydrogenase	2	21
Esterase	10	2
Sodium channel	41	0
Cytochrome b	2	11
GABA receptor	19	0

1 Number of unigenes obtained in our transcriptome database that had a hit with the corresponding proteins in non-redundant (nr) protein database of National Center for Biotechnology Information (NCBI).

2 Number of sequences from the Reference Sequence (RefSeq) database of National Center for Biotechnology Information (NCBI) that have homology with corresponding proteins (as of January 2011).

The majority of cases of pesticide resistance result from a point mutation in a single target gene. In the *P. citri* database, we identified a number of unigenes encoding pesticide resistance target proteins (Table 2), of which sodium channel and GABA receptor were first reported in *P. citri*. Sodium channels have been shown to be targets of many pesticides such as pyrethroids [44], organophosphates [45], while GABA receptors are targets of ivermectin and avermectins [46]. We also identified mitochondrial cytochrome b genes (Table 2) that have previously been demonstrated as targets of bifenazate in *P. citri* [19]. To our knowledge, cytochrome b is the only gene in which a mutation has been shown to mediate special acaricide resistance in *P. citri*. A sequence comparison of cytochrome b between our transcriptome data and the public database is shown in Text S1. In addition, a limited number of SNPs were found in different sequences that are related to the target resistance between resistant and susceptible strains (Table S1).

### Analysis of genes related to the growth and reproduction process

The primary mode of action of hexyzhiazox has been proposed to involve interfering with the growth and reproduction of mites [8], [12]. However there are no available genes participated in above proposal in the public database in *P. citri* to date. After GO classification of the unigenes, we identified 105 and 194 unigenes related to growth and reproduction, respectively (Table S2, Text S2), and several of them are important targets of pesticides in insects. For example, azadirachtin, halofenazide, tebufenoxide and pyriproxyfen, which target growth, can explain the mode of action of hexyzhiazox due to a critical brain neuropeptide, prothoracicotropic hormone (PTTH) [47]. To elucidate the relationship between genetic changes and hexyzhiazox resistance in *P. citri*, we compared transcribed sequences between resistant and susceptible strains, and identified some unigenes related to growth and reproduction (Table S3). Because there are some false positive rates in RNA-Seq data, these different sequences need to be further examined by PCR. If these different sequences between different strains are confirmed, it is very likely that further functional studies will identify them as new hexyzhiazox resistance genes.

### Conclusion

Illumina sequencing, a rapid and cost-effective method, provides an ideal means with which to analyze *P. citri* transcriptomes. It produced 32,217 unigenes with 17,581 unigenes annotated. These findings greatly extend the existing sequence resources for *P. citri* and will provide abundant genetic information for furthering understanding of the molecular mechanisms of acaricide resistance. In addition, this study identified 2,701 significantly differentially expressed genes between RS and SS. Most importantly, we identified and compared differences in genes related to both general pesticide resistance and to organism growth and reproduction. Many potential candidate genes related to special acaricide resistance in *P. citri* have been identified. The results indicated that acaricide resistance may be a complex biological process involving many genetic changes. To our knowledge, this is the first study to discover candidate genes involved in phytophagous mite resistance by comparative transcriptomics.

## Materials and Methods

### Citrus red mite rearing and resistant mite selection

A colony of Citrus red mite, *Panonychus citri* (McGregor), was collected from citrange next to citrus orchard fences (Citrus Research Institute, Chinese Academy of Agricultural Sciences), which had never been exposed to acaricides for many years. These mites were reared in citrange for 3 years under acaricide-free conditions (25±1°C, 80±5% RH and photoperiod of 14 hours light: 10 hours darkness) and considered as a susceptible strain. The susceptible strain was treated with hexyzhiazox (about 70% population mortality rate in every spraying) and continuously screened for 20 generations – this resultant strain was regarded as the resistant strain. The selection process and the resistant fold changes are shown in Table S4. The mixture of eggs, larvae and adults was collected in a PE (PerkinElmer) tube as one sample for SS and RS, respectively. The two samples were stored at −80°C until use.

### RNA isolation, integrity examination and RNA-seq library preparation

Total RNA was extracted with TRIzol regent (Invitrogen, USA) according to the manufacturer's instruction and treated with RNase-free DNase I (Takara Biotechnology, China). RNA integrity was confirmed with a minimum RNA integrated number value of 8 by the 2100 bioanalyzer (Agilent). Poly(A) mRNA was isolated with oligo-dT beads and then treated with the fragmentation buffer. The cleaved RNA fragments were then transcribed into first-strand cDNA using reverse transcriptase and random hexamer primers. This was followed by second strand cDNA synthesis using DNA polymerase I and RNaseH. The double stranded cDNA was further subjected to end-repair using T4 DNA polymerase, Klenow fragment, and T4 Polynucleotide kinase followed by a single <A> base addition using Klenow 3′ to 5′ exo− polymerase. It was then ligated with adapter or index adapter using T4 quick DNA ligase. Adaptor ligated fragments were selected according to the size and the desired range of cDNA fragments were excised from the gel. PCR was performed to selectively enrich and amplify the fragments. Finally, after validating on an Agilent 2100 Bioanalyzer and ABI StepOnePlus Real-Time PCR System, the cDNA library was sequenced on a flow cell using Illumina HiSeq2000.

### Assembly and functional annotation

*De novo* assembly of the short reads was carried out using SOAPdenovo [34] as described. After filtration of the low quality reads, the raw reads were cleaned up by removing adapter sequences. The reads obtained were randomly clipped to 21-bp k-mers, which were assembled using de Bruijin graph and SOPAdenovo software. After estimating the performance of different k-mer sizes, we found that the 21-mer was best to assemble the transcriptome, showing the de Bruijin graph complexity using small K-mers and the poor overlap regions using large K-mers. Furthermore, the reads producing large fragment without N was named contig. The contigs were joined into scaffolds using the paired-end reads. The paired-end reads were also used to fill the gaps in scaffolds, where the unigenes have the least Ns and cannot be extended on both ends. To obtain unique gene sequences, the unigenes were clustered using TGI Clustering tools [48]. In the last step, blastx (evalue < 0.00001) was employed to search for homologues of our obtained unigenes in protein databases such as nr, Swiss-Prot, KEGG and COG. The best results were used to further determine the sequence orientation of unigenes. If results from different databases conflicted with each other, a priority order (nr, Swiss-Prot, KEGG and COG) was followed to determine the sequence orientation. When a unigene aligned to none of the above databases, ESTScan software [49] was used to predict its coding regions and to determine its sequence orientation. Functional annotation by gene ontology terms (GO; http://www.geneontology.org) was analyzed with the program Blast2go. The COG and KEGG pathway annotations were performed using Blastall software against the Cluster of orthologous Groups database and the Kyoto Encyclopedia of Genes and Genomes database.

### Differential expression of unigenes

The uniquely mapped reads for a specific transcript were counted by mapping reads to assembled sequences using SOAP [50]. Then the RPKM value for each transcript was measured in reads per kilobase of transcript sequence per million mapped reads [27]. The transcript fold change was then calculated by the formula of log_2_(RS_ RPKM/SS_RPKM). If the value of either RS_ RPKM or SS_RPKM was zero, we used 0.01 instead of 0 to calculate the fold change. We modified Audic's [36] method to analyze differential expression. The formula to calculate the probability of a specific gene being expressed equally between the two samples was defined as
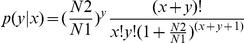
where, *N*1 and *N*2 indicate the total number of clean reads in SS and RS, respectively, and *x* and *y* indicate the mapped clean read counts of the transcript in each sample respectively. We then used the FDR (False Discovery Rate) method to determine the threshold of the *p* value in multiple tests. In this study, we used ‘FDR ≤ 0.001 and the absolute value of log_2_Ratio ≥ 1’ as the threshold to judge the significance of differentiated gene expression.

### Gene Ontology Functional Enrichment Analysis for differentially expressed genes (DEGs)

The analysis firstly maps all DEGs to GO terms in the database by virtue of calculating gene numbers for every term, followed by an ultra-geometric test to find significantly enriched GO terms in DEGs compared to the transcriptome background. The formula for calculation was
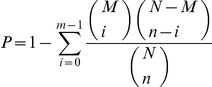
where: *N* is the number of all genes with GO annotation; *n* is the number of DEGs in *N*; *M* is the number of all genes that are annotated to the certain GO terms; *m* is the number of DEGs in *M*. The calculated p value was subjected to a Bonferroni Correction, taking a corrected *p* value of 0.05 as a threshold. GO terms fulfilling this condition were defined as significantly enriched GO terms in DEGs. This analysis is able to recognize the main biological functions that DEGs exercise. Our GO functional enrichment analysis also integrates the clustering analysis of expression patterns. Thus, we can easily obtain the expression patterns of DEGs annotated with a given GO-term.

### DEG pathway analysis

We used the Blastall program to annotate the pathways of DEGs against the KEGG database. Subsequently, we adopted the same formula as that in GO analysis to carry out DEG pathway enrichment analysis. Here *N* is the number of all genes with KEGG annotation, *n* is the number of DEGs in *N*, *M* is the number of all genes annotated to specific pathways, and *m* is the number of DEGs in *M*.

### Data deposition

Our sequencing data has been deposited in EMBL-EBI (accession: http://www.ebi.ac.uk/ena/data/view/ERP000885).

## Supporting Information

Figure S1 **17,581 all-unigenes were annotated with nr, Gene Ontology (GO), Clusters of Orthologous Groups (COG) and Kyoto Encyclopedia of Genes and Genomes (KEGG).** The smallest circle, the second smallest circle, medium-sized circle and the largest circle represent the numbers, as shown in parentheses, of all-unigenes with GO, COG, KEGG and nr annotations, respectively. The areas of H, I and J reflect the numbers of all-unigenes exclusively with annotations from KEGG, COG and Nr databases. The numbers of all-unigenes that have overlapping annotations from GO, COG and KEGG database is represented by area A (annotation shared by GO and Nr), B (annotation shared by GO, KEGG, and Nr), D (annotation shared by GO, COG and Nr), E (annotation shared by Nr and KEGG), F (annotation shared by COG, Nr and KEGG) and G (annotation shared by Nr and COG). The last area of C indicates the number of all-unigenes with overlapping annotations from GO, COG, KEGG and nr.
(TIF)

Table S1 **SNPs and the blastx results of genes related to GABA receptor and sodium channel.**
(XLS)

Table S2 **GOs of growth and reproduction related genes.**
(XLS)

Table S3 **SNPs and the blastx results of genes related to growth and reproduction.**
(XLS)

Table S4 **Resistant strain selection process.**
(DOC)

Text S1 **The comparation of both cytochrome b (cyt b) nucleotide sequence and its protein sequence between public database and current transcriptome datum.** The *Unigene12480_All* means the identity of a gene from the assembly of using the reads from both the resistant and susceptible strains. This gene is homology to the gene of *cyt b* (GenBank acc. no. HM367068) existing in the NCBI. The alignment was performed by a online software ClustalW2 (http://www.ebi.ac.uk/Tools/msa/clustalw2/); The marked in gray motifs are conserved residues of the cd1-helix of the Qo pocket of cyt b (GenBank acc. no. ADJ66666) of Panonchus citri (P. citri). The two arrows indicate the locations of two amino acid substitutions G126S and A133T related to bifenazate resistance in P. citri (Citrus red mite).
(DOC)

Text S2 **The list of full sequences of unigenes invovled in growth and reproduction: (The words in green in the list indicate the blastx results to the corresponding unigenes).**
(DOC)
