# Supplementary figures and images for: Analysis of Transcriptome Differences between Resistant and Susceptible Strains of the Citrus Red Mite Panonychus citri (Acari: Tetranychidae)
Source: PLoS One. 2011 Dec 5;6(12):e28516. doi: 10.1371/journal.pone.0028516 (PMC3230605; doi:10.1371/journal.pone.0028516)

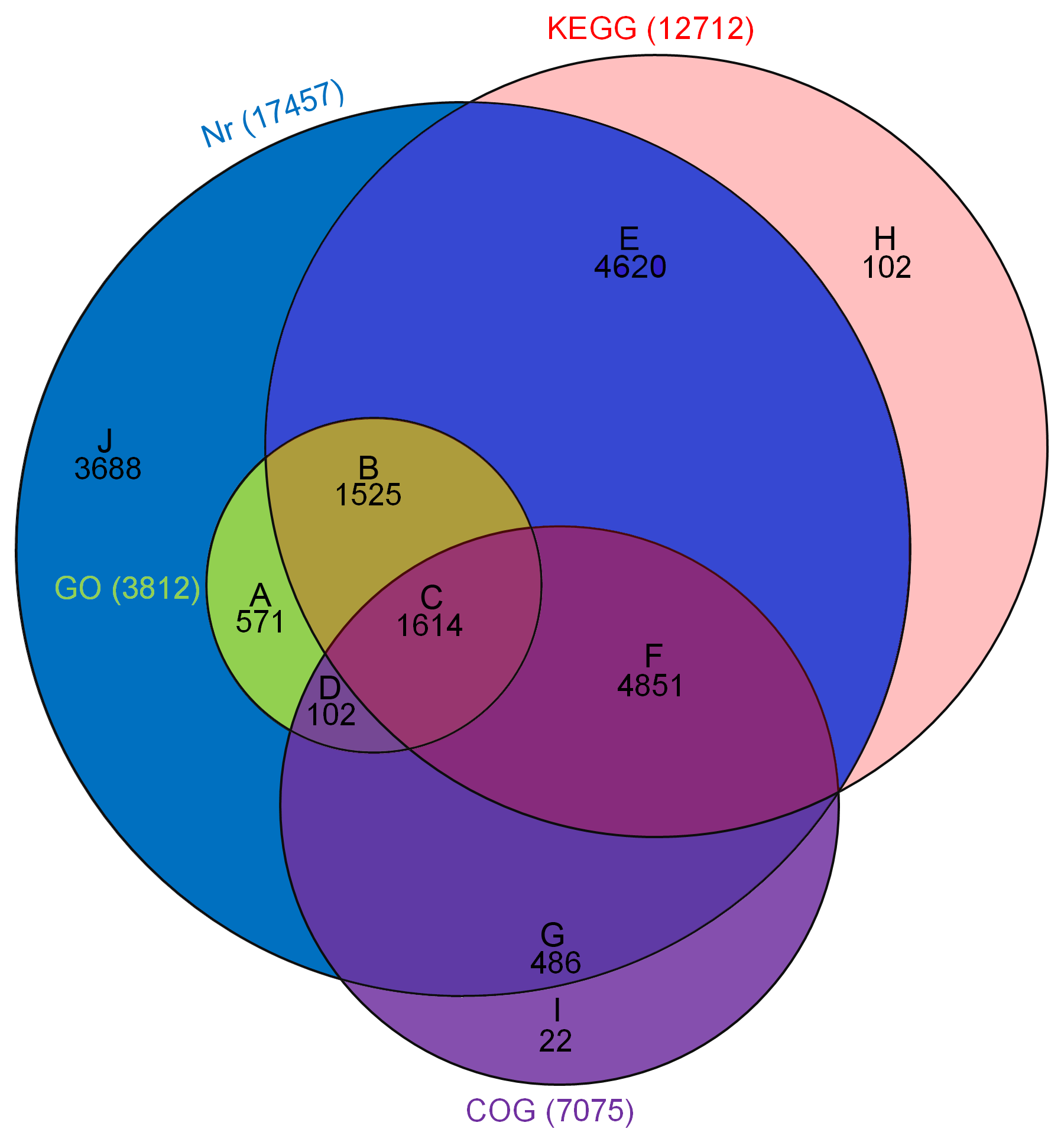

Supplement: Figure S1 — 17,581 all-unigenes were annotated with nr, Gene Ontology (GO), Clusters of Orthologous Groups (COG) and Kyoto Encyclopedia of Genes and Genomes (KEGG). The smallest circle, the second smallest circle, medium-sized circle and the largest circle represent the numbers, as shown in parentheses, of all-unigenes with GO, COG, KEGG and nr annotations, respectively. The areas of H, I and J reflect the numbers of all-unigenes exclusively with annotations from KEGG, COG and Nr databases. The numbers of all-unigenes that have overlapping annotations from GO, COG and KEGG database is represented by area A (annotation shared by GO and Nr), B (annotation shared by GO, KEGG, and Nr), D (annotation shared by GO, COG and Nr), E (annotation shared by Nr and KEGG), F (annotation shared by COG, Nr and KEGG) and G (annotation shared by Nr and COG). The last area of C indicates the number of all-unigenes with overlapping annotations from GO, COG, KEGG and nr. (TIF) [file pone.0028516.s001.tif]
