# Supplementary material for: Analysis of Transcriptome Differences between Resistant and Susceptible Strains of the Citrus Red Mite Panonychus citri (Acari: Tetranychidae)
Source: PLoS One. 2011 Dec 5;6(12):e28516. doi: 10.1371/journal.pone.0028516 (PMC3230605; doi:10.1371/journal.pone.0028516)
Supplement: Table S4 — Resistant strain selection process. (DOC) [file pone.0028516.s005.doc]

**Table S3 Resistant strain selection process**

| Number of generation selected | Independent regression equation | X^2^ | LC_50_(95% confidence limit) (μgL^-1^) | Resistant factor |
| --- | --- | --- | --- | --- |
| F_0_ | Y=1.677+1.338X | 5.238 | 0.056（0.045-0.069） | 1 |
| F_2_ | Y=-0.188+0.920X | 0.660 | 1.601（1.064-2.978） | 28.589 |
| F_4_ | Y=-0593+0.644X | 0.187 | 8.341（4.119-33.222） | 148.946 |
| F_6_ | Y=-1.026+0.598X | 0.474 | 49.902（21.240-321.233） | 891.107 |
| F_8_ | Y=-1.480+0.802X | 1.848 | 70.037（39.806-188.296） | 1250.661 |
| F_10_ | Y=-1.525+0.777X | 4.661 | 91.789（55.964-209.452） | 1639.089 |
| F_12_ | Y=-1.658+0.831X | 2.747 | 99.123（61.161-220.044） | 1770.054 |
| F_14_ | Y=-1.496+0.701X | 3.378 | 136.380（77.052-376.680） | 2435.357 |
| F_16_ | Y=-1.652+0.433X | 0.679 | 163.274（86.970-517.958） | 2915.607 |
| F_18_ | Y=-1.740+0.763X | 2.685 | 191.223（119.574-405.720） | 3414.696 |
| F_20_ | Y=-1.968+0.857X | 2.768 | 197.799（128.371-388.975） | 3532.125 |

*LC_50_ means half the lethal concentration

**F indicates generations in which were selected to test the resistant factor.
