# Supplementary material for: Analysis of Transcriptome Differences between Resistant and Susceptible Strains of the Citrus Red Mite Panonychus citri (Acari: Tetranychidae)
Source: PLoS One. 2011 Dec 5;6(12):e28516. doi: 10.1371/journal.pone.0028516 (PMC3230605; doi:10.1371/journal.pone.0028516)
Supplement: Text S1 — The comparation of both cytochrome b (cyt b) nucleotide sequence and its protein sequence between public database and current transcriptome datum. The Unigene12480_All means the identity of a gene from the assembly of using the reads from both the resistant and susceptible strains. This gene is homology to the gene of cyt b (GenBank acc. no. HM367068) existing in the NCBI. The alignment was performed by a online software ClustalW2 (http://www.ebi.ac.uk/Tools/msa/clustalw2/); The marked in gray motifs are conserved residues of the cd1-helix of the Qo pocket of cyt b (GenBank acc. no. ADJ66666) of Panonchus citri (P. citri). The two arrows indicate the locations of two amino acid substitutions G126S and A133T related to bifenazate resistance in P. citri (Citrus red mite). (DOC) [file pone.0028516.s006.doc]

Text S1 The comparation of both cytochrome b (cyt b) nucleotide sequence and its protein sequence between public database and current transcriptome datum

Nucleotide sequence alignment

*Unigene12480_All* ------------------------------------------------------------

*cyt b* ATTAAAAAAATTTTTAATTCATTAATTTTTATCAGTACTCCTTTAAATATTAGTTTAATA 60

*Unigene12480_All* ------------------------------------------------------------

*cyt b* TGAAATTTTGGTTCTATATTAGGATTAAGAATAACTGTTCAAGTTATTTCAGGATTTTTT 120

*Unigene12480_All* ------------------------------------------------------------

*cyt b* TTATCTATACATTATAATTCTGATATAGAATTAGCTTTTAATAGCTATATTTTTATAAGA 180

*Unigene12480_All* ------------------------------------------------------------

*cyt* *b* AAAATTTTTTTTAATGGGTTAATTTTACAAATAGTTCATGCCCATTTTTCTTCTATTATC 240

*Unigene12480_All* ------------------------------------------------------------

*cyt* *b* TTTATTATTATATACATTCATATTTTAAAATCTTTAATAAATAAATCTTTTAATAAAATA 300

*Unigene12480_All* ---------------GGGAATATTATATTATTTATAATTATAGGCTCAGCTTTTTTAGGA 45

*cyt* *b* TTTATGTGATTTAGTGGGAATATTATATTATTTATAATTATAGGTTCAGCTTTTTTAGGA 360

***************************** ***************

*Unigene12480_All* TATGTTTTACCTTGAGGTCAAATATCTTTTTGAGGAGCTACTGTAATTACTAATATTTTA 105

*cyt* *b* TATGTTTTACCTTGAGGTCAAATATCTTTTTGGGGAGCTACTGTAATTACTAATATTTTA 420

********************************.***************************

*Unigene12480_All* TCTTCAATCCCTTTTGTAGGATTAAAGATTACTAATTGAGTTTGAGGAGGATTTTCAGTA 165

*cyt b* TCTTCAATCCCTTTTGTAGGATTAAAGATTACTAATTGAGTTTGAGGAGGATTTTCAGTA 480

************************************************************

*Unigene12480_All* GATAATCCAACTTTAAATCGTTTTTTTTCTTTACACTTTTTATTACCTTTTTTAATTTTA 225

*cyt* *b* GATAATCCAACTTTAAATCGTTTTTTTTCTTTACACTTTTTATTACCTTTTTTAATTTTA 540

************************************************************

*Unigene12480_All* ATTATTTCT--------------------------------------------------- 234

*cyt* *b* ATTATTTCTGTAGTTCATTTAAATATTTTACATGAAAAGGGTTCCTCTAATCAAATAGGA 600

*********

*Unigene12480_All* ------------------------------------------------------------

*cyt* *b* ATATATTCTTCTATAGATAAAATTTTTTTTGGTAAAGTTTTTATGTTTAAAGATTTCATT 660

*Unigene12480_All* ------------------------------------------------------------

*cyt* *b* TCTTTGTTTTTATTAATATTATTATATTTTTTTTTTTTTTTTTTTTTTATTGATCATCAT 720

*Unigene12480_All* ------------------------------------------------------------

*cyt* *b* TATAGAATAGCAAAAGAAAATTTTTTTCCTGCTGATCCTTTAAATACTCCTATTCATATT 780

*Unigene12480_All* ------------------------------------------------------------

*cyt b* AAACCTGAATGATATTTTATATTCGCTTATTCTCTTTTACGTTCTATCCCTAGAAAAATT 840

*Unigene12480_All* ------------------------------------------------------------

*cyt* *b* GGAGGAATTATTAGTTTAATAATTTTATTTGTTTTATTTTTTAGTTTATTTCTTAATAAA 900

*Unigene12480_All* ------------------------------------------------------------

*cyt* *b* TCTTTTCATTCTAAATTTTTTTTTCTAAAAAAAATTAAAATTTTATTATTAATTATTTTT 960

*Unigene12480_All* ------------------------------------------------------------

*cyt* *b* TTCATTATTATAACTAATTTAGGGTATAAACTTATTGAATATCCTTTTACTGAATTATCT 1020

*Unigene12480_All* ---------------------------------------------

*cyt* *b* TTATTCTTTGGGTTAGTATTAATGATAAACATTTTTTTTTTATAA 1065

The *Unigene12480_All* means the identity of a gene from the assembly of using the reads from both the resistant and susceptible strains. This gene is homology to the gene of *cyt b* (GenBank acc. no. HM367068) existing in the NCBI. The alignment was performed by a online software ClustalW2 (http://www.ebi.ac.uk/Tools/msa/clustalw2/).

Protein comparation

Unigene12480_All ------------------------------------------------------------

cyt b MKKIFNSLIFISTPLNISLMWNFGSMLGLSMTVQVISGFFLSMHYNSDMELAFNSYIFMS 60

Unigene12480_All ---------------------------------------------GNIMLFMIMGSAFLG 15

cyt b KIFFNGLILQMVHAHFSSIIFIIMYIHILKSLMNKSFNKMFMWFSGNIMLFMIMGSAFLG 120

***************

G126S

A133T

Unigene12480_All YVLPWGQMSFWGATVITNILSSIPFVGLKITNWVWGGFSVDNPTLNRFFSLHFLLPFLIL 75

cyt b YVLPWGQMSFWGATVITNILSSIPFVGLKITNWVWGGFSVDNPTLNRFFSLHFLLPFLIL 180

************************************************************

Unigene12480_All IIS--------------------------------------------------------- 78

cyt b IISVVHLNILHEKGSSNQMGMYSSMDKIFFGKVFMFKDFISLFLLMLLYFFFFFFFIDHH 240

***

Unigene12480_All ------------------------------------------------------------

cyt b YSMAKENFFPADPLNTPIHIKPEWYFMFAYSLLRSIPSKIGGIISLMILFVLFFSLFLNK 300

The marked in gray motifs are conserved residues of the cd1-helix of the Qo pocket of cyt b (GenBank acc. no. ADJ66666) of Panonchus citri (P. citri). The two arrows indicate the locations of two amino acid substitutions G126S and A133T related to bifenazate resistance in P. citri (Citrus red mite).
