# Supplementary material for: Analysis of Transcriptome Differences between Resistant and Susceptible Strains of the Citrus Red Mite Panonychus citri (Acari: Tetranychidae)
Source: PLoS One. 2011 Dec 5;6(12):e28516. doi: 10.1371/journal.pone.0028516 (PMC3230605; doi:10.1371/journal.pone.0028516)
Supplement: Text S2 — The list of full sequences of unigenes invovled in growth and reproduction: (The words in green in the list indicate the blastx results to the corresponding unigenes). (DOC) [file pone.0028516.s007.doc]

| Text S2 The list of full sequences of unigenes invovled in growth and reproduction: |
| --- |
| (The words in green in the list indicate the blastx results to the corresponding unigenes)  >Unigene12141_All Netrin [Platynereis dumerilii]  GGATCTTCATAATCCGACTAATACAACTTGCTGGATATCGGAATCATTTA  CTGATCCAACGGAAAACATTTCAATAACATTGAGTTTGGGCAAAAAATAT  GAATTAACTTATGTTAGTTTACAGTTTTGCTCACAAAAACCTGATTCAAT  GGTTATCATGAAATCAACTGATTTTGGTGAAACATGGCAACCATTTCAAT  ATTATTCATCTGATTGTTTAAAAGTTTATCGTCGACCAGCAAAATCAACA  GTAACCAAATCAAATGAACAAGAACCTCTTTGTCTTGAACAGCCAATTGA  TTCACAGCCCGGAGGTAGGGTTGCCTTTTCAACCCTAGAGGGTCGACCCT  CGGCCTATGATTTTGACAATAGTCCAGTTCTTCAAGATTGGGTCACAG |
| >Unigene17455_All AGAP011396-PA [Anopheles gambiae str. PEST]  GCTCCAAGCTCTCGTTGGCGTTTAGCAATCTTTTCAAAGCGTTTGTTGGCTCGAGAAATCCATCCATCAACGGTTGCCGCTCCTTGGCCAGGGGAAATAAGTGCCAATTGTGGACCGATGCTGTTAACTTGATCAAGAAGAGAGCCATATTTAGGAAGAGCAGATTCAATTCTTTGGATTAATGATTCTTGTGCATGAGAGGTAAGTGTAGAGAAAGAATCAAGAGCTGACTCAGATTCGTCCAGCCAAGTGTTGAGACGATCATGAGCTGAAT |
| >Unigene20035_All GH22959 [Drosophila grimshawi]  GAAAATCGATCTGTATCATCAGCTAAATCACTTTCTTCTCGATTGGTTGAGACACGAAACAATTACAAGAATTTACTCCGTTCATTTGAGGCCAAACAATCGGTTTATGAATCTACAATGCGTTTGGGTTCATCTTTGAAAGATAAAAGTCCCAAAGGCGATTCTCCAGTTTTCCAACGAATGCTGAATGAATTATCATCTAAATGGGAATCAACTTTATCCACAATTAGTGAAACTTTATCTTCTCTTGAAACTGATCTTAATTGCTCGGATAAATATTCATCTCTTGTTAAAGAACTG |
| >Unigene1684_All KIAA1440-like [Rattus norvegicus]  CTCACTTTGATTCAATTCTAAAAAAAAGTATCAAAAAATTGATTGGAAAC  ATTGACAATTTAGTTGAATTATTAGATTACATTTTTACGTTTTGATTTGT  GGCTTTTTACTTAAAATGGCGATGAATATGTTGTAACAATTAACAACTGA  TTACTTGAAATGGGTCTGGTGGTTGAATTGGATTCTGAGCAACGGTGAAA  TCATATCCCAACATGTTAGCAAATTTCTTGCCCGATTTAACTCCTCGCAT  CCATTGGACTTCAATTGTGGCCTGGGCAAGTCCAATATCAACCAGGTAAC  TTTTAACTGTTTCCGGATCAGTTTCAGAGGCGCAATCAAGAGCTTCGAGT  AGCCTTTGCATGGAGGCAGCGGGTAAGCCAAATGATTGGAAAAACATGAC  CAACTGGTGAGTTTCAAGATCTCGAAGTGCCGCTTCGATTATAAGTGGTG  ATGCTGATCGTAACAATTTAAGTCTTATCCAATCATTTAACAGAGGAGCC  TCTTCTTGAGTTTTAACTAATATTCCTCTTGGCATTGAATCAGTAAACCA  CATGGTAATCAGTCTGTTGTATTGATCAAGTGCTGATAATGGTGGTTCAC  AGGTTAACAGTACAAGTAAACCTTGAATGTAAAGTGTATGAATTACTGCT  GAATGGGTTGGCCATTTAACTAAAACTAGTTCGTTACTTGATTGATTTTC  CGTTTCCCATTCCTCTTTAACTTGATTCTCCAATGAAATCATGTAATCAT  AATAAAGTTTAATACATGAGAAAAGGAAATCATCACGAAAATGTGGATCC  TTTGTGATATATTTGAATAGTGCTTGTCGATCAATGATTAGTCGGGCAAG  GTCAAAAACGGTCTGTGTATCTCCGGGTTTACCATATTCAGCTAGAAATT  GAATGTAACAGCAAACTGATTCGGGATCCGTTTCAACTAGAATTGCAGAA  CGTAAGCAAATACAGGCTATCTTTTTGACCGATTGAAAGTTTTCCGTTGT  CGGTAAATTGCCCAATAACCAGGTTTCAACGTTGGGCTTTAAAAGGTAGG  ACAAATTTTCGGCCATACTTTCATTGAAACACAGGACCAAATTCAACGCT  TTAGCGACTAAACCTCGAAGTGATGATTGAGGTGAACAGAGACGACCCAT  AAAGTATTCCATTAATTGCTTGGCCACAACAACATCACGATTAAGGGCCG  TTTGAAAGTAGCTCAATAGTTTACAAAATTTTCGTCGCTTTTCCTTTCTT  TTAGGTTTTTCGGCTTGTTTAGATTCTGTGAAAATGTTGACATTTTCCTC  GTCATTTATGTAGCGTAAAGTAAATTCACCTAAACACTGAACGGGTAAAC  TTGCTAAATTGTCACAATTCATTTCGATTAAATCATTTAACCATTGCATT  GTACCAAGACCAAGATTTAAATTGGCTGAGCAAATATTTTGACGCTGTTG  ACGTTGAATTATTTCAAGTAGAAAATCAGGATTTCGACTTTGACAAAGCC  AATAACCAATTCTNNNNNNNNNNNNNACCGGCATTGATACTTTTAAGCGT  TTCCAGTACCATGATCGGAGGCTGTCTTGGTTTACCTTGTGGGTCAAAGG  TTATCAATTTGCTTAAAAGTAAACTATTTCCCTCGGTGATATGACGTTTA  CTTGATGCAGCCGCTAAATGTATTTCAAATTGTAAAATCTGCTGCTTTTC  AATTGCGACAATTTGTTGCTCGTGACCTTTTAGATCCTCATTTGCTCGGG  TCGTTGGTGGAAATTCAAAATGATTAGTTATACACATTTCAATGATTAAA  GCAAGAGTCGGGTAATTTTCCCAAGCCATGGAACCAAATTTAGCTGCATT  ATGAGCCACAATGATCAACAATTGTAACCAAACTTTCCAATACCAATCGT  TAATCGCCAGGGAAGGTGCTTCATATCCTGAAGGAAGTGTTATATTTTCG  GGTATCCGGTAGATTGAATTTTTAAGCAATAGATTAAAAATATCGTCCTT  TTTAATGGGAAGAGTGGGATTGGTCACGTCTCCATCATAAATGGCTGCTG  CTCGTTTAATTAGAGCATCGGATAGTTCAATCGCTTCCGGTGATTGGACA  GGATAATCTTTGGATAATCCCATTTTTAATATTCTAATGAGACTATCCTC  GTGAACGGGAACCTTCGTGGTACATTGAAACATGGCCGCTCTTTCAGATT  CTGCAGGCCAATTGTCCTTATTGTAATAATGTTCAGGTATTTCCATGAGA  AGACATTTATTTAGACAGTGCATGTATTCACCTCTATCGGGTTTGTAAAT  TTTAACGACAACTTTTTGCATCCAAATGATTGCTAATCGTTGCATCTCAG  CAATTTGACCAGAGATTGTTTTGAGAATATCTTTTCGTGTTGGTTTGGAC  ATTGATTCACGAGCGACTGGGTAAACAGTGAGGAACATACAGAGAACGAT  TATATCGGCAATTGAGGTCAATACTCGGGACAAAACTTCTTGTACATCGA  TAACTGCAACTCGAGAGTTTTCCGTTGTTAGAACATCGACCAGTTTAACT  AGATTAATGTGCTCATGTTTAAGTGCTTTGACAATTTCACGGAGAAGATG  GCGAATTTGTCGGAGAAAATCATCTTTGATTAAAAATTCTTGTATCAAAT  GAGCCAAACATTTGGTCGCTTTGTTAGATGAATGTTGAAAAATGACCGCA  ATCAGGTGCATGTTGCTTGATCCTCGATTATTGGAAAATTCAATTGAGAT  CGCATTTCGAAGGACAATCATAATGTTACCATCACTTTGACTCAAAAGTT  CTTTAATACAAACAAAGAAATGATTCATTAGAACTTTGGTTTTAAGTCGC  ATTTTAACCAGAGCGGAGATCACATCACTATCCTCTTCGGTACAGTTTAC  ACAGACCGCTAGTAAAAGGTCTTGAGCGGGTCGACTTAGTTTTGGATTTT  GTAGCCAAATTTCCAACTTTTGAGCGACCAAAAGCCGAATCTGTGGTATA  CCAACTGTAGATATTAACAATTTGAGTACATTTCGATTGATTTCCGATGG  TACCTGACGTCGGGTTAATTGTTCATTGATAATATCATAAACATAATCAT  AAACAACTTGTTGGTTGTGATTATATCGAGGTACAACCGGAAGCTCTTCA  CCGAAAACATCTTTCAACCCGGAGGAAAACAAACTACTCGTTTCATCATT  ATTATCCTCAGAAGCGTGACTTGGACTAGTTGATATGGGAGATTCATTGG  TTCCTTTAATACCACCAATTAGTGGTCCAGTTGAAGATGAAGGGGTTGTC  ACAGTGCCACCGGTAACAGCGGCAAGAATATCTTGAGACATTAAATTCTT  GGGCGGATTTTTGGTATTAAAAGCGGTCAGTATATTGTCAACAAAGCCAC  GGCAAAAATCAGAATCAACCCAAATACGTTCACCAAGAGAATCCTCTATG  TAGGCCTTGATAAATGATAGATGCCAATTGGGTTCATCAAGATGAGCAGC  AAGTAAGAGATTAGCAGCTAAAGCTGAGCAAATTGTTACATTGGAATTTA  CTTTAACCTTAAATGAGCTGGAAGAAGATGGCGATTCCCGGCGGAGAAGT  GAACACATCGCATCGACAACCTTTTCACTAGTAAAATGTGAAGGTCTAGT  TTTCGCCAAATAAACTAGACTTAAAAATAGAACTGAATCAGGTTTAATTT  TACTCGATCGAAGTTGTTTAACAGCACCACAAATGTAATTTGCAATTTTG  GGCTCATGAATTGTTCCCTCGGCTTCAACAATCCGTTTTATCAGAAACTC  GGGTTCAACATCAATGGAAACATCTTCCNNNNNNNNNNNNNNNNNNNNNN  NNNNNNNNNNNNNNNNNNNNNNNNNNNNNNNNNNNNNNNNNNNNNCCGGA  GCTTCCCCCACCACCACTGGAACCACCGATCAATACTTTTGGCCTTTTAG  CTACGGTACCACCGAGGGCGAATAAGCTACTGGTCGCTGGCAGGGGAACC  TTTGATTTACCACCGCGTTTACCAGGAACAGAAGACATGACACAAAGAGA  ATTTAAAAGGCAACAGTTTATCCAATAGATAGATAGTTAAATTCTCATGC  TAATTAAGAAACAATTAAAATAAAGAACAACAATTAGTTCAATTACAACG  GAATAAAAAATTAAGAT |
| >Unigene17792_All Similar to GINS complex subunit 1 (Psf1 homolog) [Monodelphis domestica]  GCTCGATGATTCCTTGGTGTATCAATTTTTCACATTGAGAAAATGGTAAATGGTATAAACTGTCTTTTTGTAGACAAACGGTTGTTCCATCATCTAATTCANNNNNNNNNNNNATCAACAATACATTTGACTTGAATATAAACCCTTTTGGGAGCAGTTTGATTAAGGGTTAAATCAAAACCTTGTCCTTCATTTAGCCTACTCATGTAGGTACTTAAATTTGAACAATAAATGTTAAACCATTCAACCTCTTTGGGACTGAGGTTTCCAGAGACTTCACATGGTAATTCAGCTCCAAATTCCCAGCGAAGTTTTTTTAACCGGTTCAATCTTTCATAATGGTAAGCAATTAGACAACGTTTTATGAATAAAATGATTGAATGTCTTATTTTGACAACAATCAGTCCATCTTGNNNNNNNNNNNNNNNNNNNNNNNNNTGCTCTAAGGCTTCCTTTTGATTTTCTTCAAACAATTTTCTTATTGATGTCAAACATTCACTAACAATCTCTTGATTGTGCAAAGGAAGAAAATCACCCTTAGAATTACTCAATTCTTTTAGCATAGTTGAACATCTATCGATAATATTCATGGCGACGAGAAAATGTTAAAAAAAACACTACACATGAATAAAAATCTA |
| >Unigene21924_All Transforming growth factor-beta [Crassostrea ariakensis]  AAATAATCAATGGAGAAAAGTATAAAGTCGGAACCATACGATACATGGCACCAGAAATTTTGTCCGATACATTTGATGGAAGTAAATTTGAGAGTCATAAAAAAGCAGATATTTATTCACTGTCTCTGGTGGTATGGGAAGTCCTTCGCCGGTGTATCAGTCGAGATGGTAAAGTTGATGATTACAGTTTACCTTTCGGAGGAATCGTTCCAAGTGATCCAACCTTTGAAGATATGGC |
| >Unigene31549_All Nuclear distribution protein nudE-like 1-like [Ailuropoda melanoleuca]  GAACAATCAAATGATGATTTAGAAAGAGCTCGAAGAGCTTTAATTGTATCAGTGGAAGATCTTGAATCTCAGTTAAATCAACAAATAGAAAAGAATGTTCTTCTTGAAACAACCATTTCACCATCAATCGATCCACCCAGTGAAGAGAGTATAACAATTAGTGAAATCTAATCCACCATTTAAATCATCAACATTTAAATCACAAAAACTTAACCTCAATTGTATCCAAAAAAAAATATTAATTCTTATAGAAAACATTACAACTTCATCAAAAGAGGATAAAAACACTCAGCAATCATCAAACATTTTACCTGTTCAATCTCATTAATCAATCACCAGAATGAATCACCAATTGATATAAAAACACTTCAACAACGGAGC |
| >Unigene12673_All Similar to erect wing CG3114-PF, isoform F [Apis mellifera]  GGAGCATAATGTACCTGTTGATACTGCATATTGACACTTTGTGCTGCTAC  CGCTAAAGTGTCAGTCGCAGAGTCTGCTGATAGAGTAGGTAGACTGGTGA  TGTTAGAACTCCCTGGCACTGCTGGAACTGCTGTTACGCTGATGCTTGTG  TGAGTACGTGGCTTCACCTTCTCTTTGGATGATTCTTGTTTTTTATCGTC  ATCTTCACTAAATACCGGAAGCATGTCCTCGCGATTATGGTACTTATAAC  AATTTATCACAATTTGTCGGAGAGCATGAGTCCATGAAACCTTTTGCTTC  TCTTCGTCACTGCGAGCATCACTTCTAACGTTTGCCCAAGGAAGATCTTT  AGGCCACCATACAGGTCTGGTGGATTCTTTTCCCCAACCAGGTTTACCGC  GTCCGGTGGAATATTTCAACATTAGAGGTATAAAAGCTCTTAGTTGTGCT  TGAGTCATTTTTTCCACTGGAGTTGGAATACCATCTACGACTAGAGGGGG  TAATTCATGACGAGAAGGATCTTCTTTGACTTGAGGTGGTGCTTGTTGAG  CCAAAGCTGATTCAAGCTCTTGCATTATAATATTCCTACAATTTCGAACC  ACATTTTCTAATGGTCTAGCACCAAATACTTTGTAATTATTTTGAGGTTT  TCCTGGTGTGACTATCAAAACCACTGCTTGTTGACCAACACGTGTAGTGT  ACTCATCGATCGTTGCTTTCAATTTTCTCGATAATCTTGTTTGTTGTCGT  TTTCTTATCGAAGGATTTGTCTCAAAAGAGTGAGGTCTCTTTCTTTTCTT  GGCGGTAGCAA  >Unigene14585_All Similar to morula CG3060-PA, partial [Apis mellifera]  CACTCTTTCAAGTCTTCGAGAGCAGGTTCAGAATCAGGATATTCTATGAT  CATATCAAACATTTGACTTGTTCTCATTTGAGCATAAATTTCATGCATAA  AGTGTAAAAGTCTTTGCCTTGATTATTAAAGCTTGATCATCGCCGAAAAC  CAATTGACGCCATTTAAATATCACATTGTTTATCCAAATTTGTAATTTTT  TGAGATAAGATTCGTCAAAGCTTTTTTTACACTTTTCAACAACGTAGGTT  TCAATGTGATTATGAATAACCGAAGCAACTGCATCACCACTGATCTCATC  CAACAGGTCTAGATCCCGAAGATCGTGGGTTAATTTGATAAATTTGTTCA  TTATCAGCTGACAGTTACATAAGTTTTCATTTCCAGCAACTTCTGGGTGA  TTAGACATAACTGCTCCTTTACAAATATTACATTCACCATCTAGATTAGA  GCTTGTGTCCATTTCTCTATCCTCATCTTTATCACAATTTTCATTCAATG  AAAGAGGATTTAATGACCTGGAAACAGAAAATCCATACTTGTAACATGTT  AAAATACGTGAATTAAAGGATTGTTCAATCTTGTAAAATAATAAAGATTT  GATCAGATATCTC |
| >Unigene19653_All Similar to CG3060-PA [Nasonia vitripennis] GGTTTTCAGGCTAATTTATATTGACCATTAACAAAGACCAGTTTATGCTCTCGAACTTTGGTTTCAAGAAAATTACGAAGCTGTTGGATTGTTAATTGTTGCTCATTTGATCCTTGCAAGGCAAACATATTTAACATTGATCTGATTCTTTCCAATGGTAGAGAGTTTAAATTGGTCAACATATTTTCAATGAATGACCAATAAACTTTAAGTTTATTTTCACCGACATCTTGATTATTTGAAGATGCGGCTTGATTTATTTCTTGTAATGATTCATCTTCATAATCTTCTTCATCATCAGTTGGATTAACAACTAATTCCGCTTGAGAGGAACTTGAGCCTTGAGTTTGATTAGTAACTAGAATGAAACGATCACTGGAAATCTCTTTGATTATTCCCTTGTTTTGCCAGAAACTGATCTTCCTCCTAAGAAGAGCAGTTGAAACAGAAAGACTTTGACTTAATTCAGATAAATTCCAAGATTCTTTTTCCTGAAACTGCCAAATAATTGCCGCATGAATTGGTGTTACGGTGAATGATTGTTTCTTATCGGCTGTTCCAAAATCTAATTCAAGATCAACTGATCCAAGAGGTGAAATCCAATTTAATGTACGACTTCCCTTGATTGTTTCGAAAGCCTGAGTATAAGTTTTAATTGATTCTATCACTGGTGGTGGAAGTTTCAATTTATTGGCTTCCTCGTTTACATTTAGTCCAAGTTTATCAGGCCAAAATTGTGCCGATAAAATGAGACATTTAATTGGAAATTGGGTCCAGTGATGAGCTTCAATTTCACCGGCGTTGATTCGATTGTCAATTCTTTTAGAAGATGAAATATCTTGTAACATTACTTCACAAGAATGGAGATCAGATTCACCAAAGCGAAGTGTCAAAAGTTCTAAATTTCGCCTTTCATGTTCTACATTACATTCAAAGTTGTGTAAAAATCGTTGAGCTAATAATCTTTGATATTCTTCAACGAAAAGATCTTTACTTTCGTAAACNNNNNNNNNNNNNNNNNNNNNNNNNNNNNNNNNNNGAACTGATTTAGATCGACCAAGATTTCCAGCATCAACTGGATCTGGTTTCCATGATTTCCAATTTTTACCAATCAATTCATCGTCTGTCATAGTGGAAACATCATCAATATTTTGAGAAACATCTTTAGCAAGTTCTGGGACAAGTTCGGAACTTTCATCGGTTAAAGCGGTAATAATACAACGAACTGTATCTTCGCGACTTTTTAAATACTTTTTAATTGGATCACAAACAATTTCAAGAATAACACCACTGGGGTCGAGTATTTTAAGTGCCTTGATTGTTTGAATATACGCAGTTAATATGTCATTCGTTGTGACACCAGGATGAAGAAGTCGAGCATCAAACGAATCTCTTATTGAGTTAATT |
| >Unigene23363_All GA11538 [Drosophila pseudoobscura pseudoobscura]  CCGTTGTAAAGGGTGTGTCATTGATTGCAGTCCAGCAGAATTTATTTAATCTTGTCATCCCTCGTTTCATTGAATCGACGTGCAATTTAACTTTTAATGTTCGACAATCAGGCTTCCGATAGATCATATTTTTCCGCATCTCAATTAAATCAGGACTAAATTGCCTGGATTCCTTTTTATTATCAGCAATCTCAACTTCCAGAATATCAACTGAGCTGTAATAGGGCTTTAATAGATTTTGAAACAGAGTGCGAAGGTCTGGATGATAAAGAACGAAACCATTGTTATTGATACTGAACGAATAACCATTTACACCAATCTTATAACTTGGAATCAATTTGATTATTTCATTTAGCGGAACATCAACACCAGCAACACCTAAAAGTGTTGAGTTCCTACGATTTAAGACTGGAATTGCAATTGTTGTCATCAGATCGAATGGAACCCGAGGCTTAAACTTTTC |
| >Unigene11385_All GK14960 [Drosophila willistoni]  GTGAGACAAAGTTGTTTTAATTCAAAATTTTCTCGTACCAATTCAAATTG  TTTGCCTTCCAATTGAGTTAATTTGTTTGAATAATTAGTTACTTCATGTC  TCATAACTCTTGACATATGAGTACCAAAGGATTGCCATTCTCGAGCATTG  ATCGACATCTCGTTCGTTCATCCTCCAAGAAACAGGCCAATTCCCGAAGC  TCATTGTTTTCTGCGATAAGATGATAATTTTGCTGTTTCAATTGTTGCAA  TGAAGATAAATGATGGTTAATATCTTTGGTCATTGAACCATTGTCATAAA  GAAGTTTCCTATTTTTAGTTTCTAAACTTCGAATTCGTGAGATTAACTCA  TCCAATGAAAGTTGACCCAGAAGTTCATCATCAGGAGTGGGTGTTGCTGG  GGTGGTAATTGAAGAAGTCACTGGACTCCAAACATCGGCTCTTTGTGCAT  AAACGGTTGAACCATGAGGGTGATTTGTGGTTGAAGAGTTGGTACCCTGA  TGTGCCTGAGAAACTTGATAAACCGATGAAGATGATGGTTTACCAAGAGG  AAGAGGAAGATTCTTATAATGAGAGTCAACTGGTTGTTGTTGTTGTGGCT  TAGGTTGATGATGAGAAGGCTGCTGCTGCTGCTGCTGTTGGTGATGGTGA  TGATGATGATGATGATGATGATGAAGATGGATGAGTTGACCCTGCTAAAG  AACCTGAGGCCTGAGGAGGTAAAGGTGGAGGTTGGCCAACAACTTGATGA  CTAACCTCATCACCTCCAAGTATATTGGATGCAGATGAACTAGACATTTG  GGTCAAAGGTAACACAGTGATCAAAAGGTATCATGGAATATCCTAGAATC  TCCTCTTAGTCCCAAGACAACTAATTCTCTTCCAATCAATAAATTAATTA  AGA |
| >Unigene11393_All Similar to forkhead box K1 [Tribolium castaneum]  TGCTGCTAATTCTTGTCCCACAAGCCCTCGAGCTGGTTCACGTTATCGTA  ACAGTGGAGTGACCAGTGACCTACAAGCAATGGTTGAATACGCTGCTGCT  GCTGTGACCTCCGAGGAACACCGAGTTCAAGCAAATATGATTGCAACCTC  TGTAACAAATGACACCAGTGTTAACAAATCATCATCTTTATCAATTAACT  CTAACCTATCAACCTCACAGACTGTTAACCACTCTGACTATTATTCACAA  TCATCATCAACTCTGAGTCATTGTCAAGTTGCTGCAAATGAAAGTGTTTC  CAATAGTCCAAGTGGTGTAGGAGGAGGCGGAGGTGGATCAGGTGCCATTG  GGGAAATAGATGAAACTAAACCACCATATTCATATGCTCAGTTAATTGTT  CAAGCTATTTCTAGTGCTTCAGATAAACAGTTAACTCTTAGTGGTATATA  TTCATTCATAACCAAAAACTATCCCTATTATCGTACTGCTGATAAAGGTT  GGCAAAACTCAATTCGACACAATTTGTCATTAAATCGATATTTTGTTAAA  GTGGCGCGATCTCAAGAAGAGCCCGGGAAAGGTTCATTCTGGAGGATAGA  CCCTTCATCGGAACCCAAGCTTGTCGAGCAAGCATTCAAGAGACGGCGTC  AACGGCCAATATCATGTTTTCGAAATCAAATGAGCAGTTCAAGATCGGCT  CCAGCTTCACCCAATCATCTGGGTGGTGGCGGAGGCAATGTTTCTGGTCT  AGTTACCCCGGAATCCTTATCACGAGAGCCAAGTCCGTCACCTGAGATGG  GTGACAATGATGCAAATCAATTAATGGGTCCTCCAACAGCGTCATTCCTT  ACAGTGCCAAGTGATTATAAAATGTCAAACAAATCAGCTCCAGGATCACC  AGAATCTCATTGCAATTTTCTCTCCCCTTCTTCGGCAGGTATTACAATTA  CAACAGATTCAGGGGTGTTCACAAGCGCCGGCGGAACTCAATTTCAAAAA  AAA  >Unigene14101_All Similar to exostosin-2 [Nasonia vitripennis]  ACTTTACATAGTCTTGACTATTGGTCAGGTGATGGTCGCAATCATTTAAT  CATTAACCTTAATCAAAATGTCGATATAATTGATTACGGGATCAAATCTT  TTCGAGCTCTAGTCCTTCAAAGTAATTTTAATGGTAATCACTTACGAGAA  GATTTTGATGTCATAATCCCATCGCTTACAATTAACAATAATAATCATCT  GATTTCATCATCAAAAATACCAAATTGTCCAGCAAGACGAAAATATCTCA  TCACATATCAGGGATCATTAAATCAATCAACTTCTAATGATGGCCAATAT  CGACAATTATTTGAGATAATGACTTCAATCGCCAATTCATCGACCAATGA  TGAGATCCTTTTTAATTTCAATTGTGCCAACACTAATTGTACAAGTTTTT  TTACTCAATCAACATTTTCCATTGTGTTACCTCCTTTGAATACTTTAAAT  CAGCGAGGATACAAATCTCATCCTGTTCTCATTGACGAGACACTTGAACT  GCTCTCTTTCGGTGTGATTCCAGTGTTAATTGGTGGAAATGATTACAAGT  TACCATTCCATGAGATTATTGATTGGAAGAAAATTGTTTTACATCTTCCA  CTTGCCAGAATGCCAGAGATTCACCTGATTGTTAAATCATTCAGTGATTC  AGATATCATTGATATGAGGCGACAGGGAAAGTTAATTTATGATCGTTACT  TTTCAACGATTGATTCAAGAATCAATTCATTGATCTCATTTGTCAGACAT  CAGCGTCTCAACATTCCCGCTTTACCTGTGGTGGAAAACACTTTACCCTT  TCTATTCAATTCCACTTACTCTTCCAATAAATATTTGTATGCATTTGAAA  ATCCAGAGAGTATTGGTGGTAATCAGGAAACGGAAATCGATCGAAATCTT  GGTCCAATTGAATCACCTTTTCCATCGATCACTTTTCAACGTAACTTTTC  ACTTACTCTGTGCCATGGTTACTCTTTATGGAATTCACCTCAACTCAATC  CATTTACCCTTTATCCGAATACACCTTTTGATCCTGTCCTACCTTCGGAG  GCCAAATTTACAGGCTCAGGTTATGGATTCCGTCCAATTGGTCAAGGAGC  TGGTGGAACAGGTAAAGAGTTTAGTGAAGCTCTTGGTGGTAACTGGCCAA  AGGAACAATTTACAATCGTCATGTTAACTTATGAACGAGAAACAATTTTA  ATTAAATCGTTGGAGAGACTTTTAGGTTTACCTTATTTAAACAAAGTTTT  GGTCATTTGGAATAGTCCTAGAAAACCTTCAGATGATGTTAAATGGCCTG  ATCTTGGTGTACCAATTAACGTAATTGAAGCAGATCAAAACAGTTTGAAC  AATCGTTTCAAACCTTATTCTCAAATAGAAACTGAAGCAATACTTTCAAT  GGACGATGATGTTCATCTTCGTCACGATGAGATAATATTTGGTTTTCGTG  TTTGGAGGGAAGCTCGTGATCGTGTTGTTGGTTTTCCAGGTCGATATCAT  GCTTGGGATAACAATTTCAATTCGTGGCTTTACAATTCAAATTACACTTG  TGAATTATCAATGGTTCTAACTGGAGCAGCTTTTTTTCACAAATATTATA  CTTACATGTACACTCATTCAATGGATAAAGCAATAAGAGAAAAAGTCGAT  GAATTGATGAACTGTGAAGATATTGCAATGAATTTTTTAGTCTCTCATAT  AACTCGTCAACCACCATTGAAGGTCACATCACGATGGACCTTTAAATGTG  TTGGATGTCCAGTCCGACTGTCAGAAGATGATTCTCACTTTCAAGAAAGA  CACAAGTGTATCAATTATTTCACTTCGCTTTATGGTTACAATCCACTTTT  ACAAACCCAATTCCGAGCTGATTCCATACTGTTTAAAACTCGTATTCCTC  ATGATAAAACCAAGTGTTTCAAATTCCTGTAAATTCAAATCCATAACCTT  GTATAAAAAATGAAAAAGAACAATTTTAAATTAAAT |
| >Unigene14310_All Similar to phospholipase c epsilon [Tribolium castaneum]  GGGGCATCAAGATCACCGATATTAAAGGCCGTTGTTGTGGTTGCACCAAT  TTCCACACCTTCTTCTTGAGATGATGAGTTTATGAATAATGTTGAAAGTG  GAAGCTGCTGATTTTGAGGACCTTGAAGTCGTAAATGTCGGTAACCAGGT  TTCAAAGATTTAACGGGAACAACTCGATGAGATACAAGGTGATTAGTGCC  AATGTCGATTACAGCGAAACGTATAAATGCAAGATCAGTAAATACAATTC  TAAATACAAAAGTATTGTTCCAAATAGGATTTAATGAATTCCGTTGAACC  AATTTGGTTCGTTGTTTGGCACAATCGACCGGTAGTCCAATGATCTCAAC  TTCCACTTGTGGACTTCCGTTATTCACATCAGGGCAAACATATTGACCAG  AGATTATCTGAATAGTT |
| >Unigene23637_All Similar to zuotin related factor 1 [Equus caballus]  TCCTCTTGTTTCATTTTTCGAGCTTCTTCCTTGGCTTTTTTTCCATCAACTTCTTATTCTTGACTTCTTCCTTGAATTTAATAATTCTCGGGTCACATGCATAAGCATTATCGACGAGTTTCCTAATTCGAGCGAGTTCTTCTTTCTTTTTCTTTTGCCTTTCCATTTTATTCTGTCTTTCAATCCATTTTCTTTCGCTTCTATCTTGTCCACGCTCTTTATCCTCTTCATCTAAGTATGAATATTCCCGCCACGAGTCGAAATTGTACCAAAAGTGATAAAAATTTTCAACATAATCCTTAGAGCAAAATTCATTTCCTAGCTGAGGTACATATCTGGAATTCGACCATCTGGAGTTACGAGCAAAAACGGGAGCAAATACACCATAGAAATTTTCTTTAGAATTAGAATTAACTGATGGAACAGTATCGTCGAAGTGTTCATCAACACTGTCATAAGATTTTCTTTTAATTGGATCTCCTAGAATCTGAAAAGCTTTGGTTATACATGAAAAATAATCGGTCTCATCATCTTTATTTTTAACACCTCGTTTATCTGGATGATGAGTCAAAACCAATCTCCTATAAGCTTGTTTAATTTCATCATCAGAAGCCGCATTTCTCCTGCTGCCAAGACCGAGAACTCTGTAATGATCTTGTTCTTTCCATCTTCTAGGATCCAAGCTTTTTAAATAATCCTCATCAGTTTCAAAAAATAATTCCTCTGATTTAGTTTCTTCTTCAACCGGTTCATCGGGGATTAATTTAAGCTCAATTAAGTAACGTTTCCACCAAATATGATAAGGAGTAATATGAACCAAAGCTTTATTGAGAGGACCGTAACTCATGATGACAGGACACTTGACCAAGGTAAACACTAAAATTGACTAAAAAAATTAAATATTATCACCAAACAATTACGATGAAAACGCCTGAAGATGGGAAAGTCTGAAGGAGAAAGAGAGAGAGAGAGAATCTTCACCTTTAAGAGTATTGACACAGATTGACTAT  >Unigene26435_All Similar to Rgk2 CG15069-PA, isoform A [Apis mellifera]  TTTCTTTAGATAGAGAGAGAGAGAGATTGGTGACAATTAGTTAATTACAATTAAAGTACATGAAGATTGTCACATGATTTTGATTTCTTGAGAACTGCTTTGTTTAGGAAACGTTTAATTATGTTTGGCCGGCGACCGTTAGATTGACTTAATTGTTGTTTCTTTGAGTTAATTTTGAGGTTTATCTGGACACCGATTCCGGTTAATAGGAAGTCAACGTTATGTGATATTGCACATGAAGTTTCAATGTATTTACAATTGTTCATCATGGCGATTTGTCTTCCCTCTTGTTTGGATACAGTTCGACTTCGAACCAGATCAGTTTTATTAGCGACCAATATAATCGGTTTACTATCGACATCGTCCCATTTGAAGATCTCTGAGATCGTTTTCTTTGCCTTTTCAAACGATTCTCTGCTATTCACTCCATAGACAACAATATAAGCATCAGGCTGATAAACGGTGACCGGAGATCGTGGATAACATGAGTTGAGTGAATTGGCTTCGATAAAGTTAAGATACAAGATCGGTTTGATTCCAGAATCATTTGAACGTTTNNNNNNNNNNNNNNNNNNNNNNNNNNNATATTTGGTTCGGTAATAAGCATAGTCCCGATCGATTGAATCGACATCGGCCAACATTTGCTCATCAATATAAGCCTCGAATTGCTTAATCAAACTGCCGAACTCGAAGAGCCAAGAACCAGAACTCGACATTTACTAAAATTTTCTTGTTTGTTTTGATTGTTGTTGTTAACTAGTGTGTTTGTGGTGGTTTGTTGATTGTTACAGTTGATTATGTTGGTTGTGTTTTGATTTTCTTGGGAATCATTTGAAATCATTAAAGTAAACGGTAAACTGGCCTCAGGTCGATCAATTTTATATTGATAATATTTAACGAAATCAGTTTCGGTGTGAGATCGGCTACTTTCAGTAAAAGATGAGGGTGTTGATGAGACTCGACGGGAACGGAAACTTGGTGTCGATGAGAGTGAACTAAATGAACTATTGCCCGAAGATGATGAACCATTACCTCCGTTACCATTGGTACCGTTTATTAGACCGTTAGGATTTTTCATCGATTGGCATCTTCGAACTGGATTAAGTTTCCGTTTGGTTGAACTATTGTAACGACTAAATGAACCAATTGGACCAAAAGATGGAGGTGATTTACCGGTTGGTGTTGATGGACCTGAACTTGGAGTTGATGGTTGCGAATTTGAAGTCGACGATGATGAACTTCGTCGACTATCGGCCGACATGGCCAATTTGATTGCGGTAACCAGGTTGATGCCGGTACTCAATTAAATATATATTTGAATGATACGAAAAACTTTGAGTCAACAATCAAATTAAATCACAATAACAAAATGAAACTTTTGCTTCAATGTTTCAAAGTTAATTGTTTGACTTGATTAAT  >Unigene31860_All Serine/threonine-protein kinase Sgk1 [Fundulus heteroclitus]  CAACTACATTCTGCGGTACACCAGAATATTTAGCACCTGAAGTACTAAGGAAAGAAGCTTACGATCGTAATGTTGACTGGTGGTGTTTAGGTTCGGTTATCTATGAGATGCTGTACAGTTTGCCACCGTTTTATAATCGAGAAAAATCTAAGATGTATCTAAGTATTCTTAATGATCAGGTTAAGCTTCGTTTAAACATATCAACTGCCGCGCGTGATATTTTACAGGGTTTGTTACGAAAAGATAAAAGTCGAAGACTTGGCGCTCTAAGAGATGCGGACGAGATAAAAGAACATGAGTTTTTCAGGCCAATCGATTGGAAAGATCTCGAAGCAAAAAAAATTCAACCACCTTTCAACCCGAATGTTCGTGGATACTTTGATTTAAAGAATATCGATCCAGAATTCACAAAGGAACCGGTTAATCCTTCACTCTGCCGATCGGTTGCCAGTAATAGTGTTCCAGATAATGCGTTCTCAGGTTTCTCATACGTCGCG  >Unigene31902_All Similar to small heat shock protein 21 [Tribolium castaneum]  AAATCTCCTAAACTCTTAAAACTCTTAAACCTTTCAAATCTTGAAAATTTTCTCAAATATCTTTATTTACTTGTGATACCACTAATCGTTTAAAAAGTGAAAATGTCGTTGATTTTGTCTCGCCGATCTCCATGGTTGATGGAACCCCGTATGTCTGATTTAACTGGTCTCTCGCCGTTTTATCAGCATTTAATTGACACCTTAGACTCGGTTAATGACAACAAAGACACGTATGAGGTATCAGTCGATGTTAAGGACTACAAACCCGATGAGATTTCGATTACCATGAAGGACGATAAGCTTGTCATCAAAGGTAAACATGAAGAACGTAAAGATGAACATGGTTTCATTTCACGTGAATTTGTAAGAACATTCACAGTTCCAAAGAACGTTCAACTCGATAAAATGGAATCTCTTTTGGGTAATGATTCAGTTCTTCGTATTAAAGCACCGAAGGTTCAAGCTGAGAAGCCAAAGGATCGTAGCATTCCAATCACCTTCGAGAAAAGCAAATAAAGAAATGCAAA  >Unigene3210_All Nucleoside diphosphate kinase 6 [Danio rerio]  TACAATTAACTCTTTGTCTAATCAAACCCGATATAACTCCGATCGCTTGT  AAAACAATTGATCTCAGAAATCTAATTTGCAGAAAAGGATTTTACTTTAT  TCGATCTCGATGTTGCAAATTGGATCAAGCTGATGCTGAGAGTTTCTATC  ATCAACATAAAGATAAATTTTTCTTCCATCGTTTGGTTACCTTTATGTCA  AGTGGACCCATTTGGGCTCATATTTTGGCTCATCCCAATGCTATTAAACA  CTGGAGATCCATCATGGGACCAACAAAGGTTTACAGAGCAGTTTATGAAG  CACCAAATACGAAAGCCGATCCGTGGCTTATATGAATTAACAGATACAAG  AAATGCCGTTCATGGCTCCGATTCTGAAGATTCAGCTCTTGAAGAGATCG  CTTTTTACTTTAAAGATTTTTCAATTGACCAGTGGAGACAAAAAGAGGAA  AATTTTTTCATCGATCGATCAAAGCTAATTACATTTGATCCAATTTATTG  TGTCCACGAATTGAAAGGAGAAAATAGAATGGAAAATGCTAATTAAAACA  TTTACTCGAAACTATTGAACAAACAACTTAAAAAGTAAATTCGACGA  >Unigene3319_All Similar to CG11166 CG11166-PD [Tribolium castaneum]  TCGCGAGCCTCCTTCAACTCGAGTCTTTTTAACTGCAATGTTGTTGGTCA  ATCTTTCCAATGTTAATTCACCTGTTTCATTGTCGATGATTAGAACACAT  TCCTTGAGGTGAGGTTTTCTCGAACCTTTGAAAACGGTTTGAGGCAGAGA  CGATGAGTCCACATTCGGCATAACCACCGTCACTTTATTATTCTCACCAA  ACTCTATATCTGCTCTTTTGGTGGTGTCAACAGAAGCTGGTTTGAAATCG  TATCGAATCGTGTGAAGGCCTTCGGGTGGTTGGTTCGATCCTCGGTTAGC  CCGATGGTTTTGGAAACTTTTTCCAAGTTTCAACTGTCGCGTTTCCCCAC  TAATCCCAAGTTTCTCTATGATGTTAAACATTTTGATCGATCACTTTGAG  AAAATGAATGAGGTTGAAGCTCA  >Unigene3898_All Similar to phosphatase [Nasonia vitripennis]  TTATGCAGCCAATCATTAGGAGAGAGAATAGAGAAAGAGAGAGAGAGAAA  GGAAGTTTTAAGGCGTTGAAAAAAGTGTTCCAATCAAATGACCATAAACA  CGGAGAATCGAAAATCACCTTTTAAATTGAATTTTACAATTTCCACTTTT  ATTCCCCTTACGTTATTTAGACACAAATACAAACACACACGACGAGATCT  TGATTAATCTTTTTCATTCCTTCTATACAAGTGGCATCACATTAGTCCAG  ATGATTGAAGGGTAACGATCGCTTCATTAGCAAAATAACGGACATCGGGA  TCAGAATCGGTATTTAACTTTTCTAGAACAGGTTTCACCATGTTTTGGAG  TGTACTCTGATCAAGTGTGGAGCCGATTTTTTGTAAAGCTTTTGCCACAT  TAAACCGGACATTTGCAACATTATCATTAGCTAAAGTCAAAACGGTGGGA  AGCATCAGCCTAGTTGTTATATCAGCTCCACAGATACCCGCCAGATCATC  GATACTGAACAAACATGTCATTCTATGGAGATAATTTTGGTCTTTAGACA  TCTGAATAACTTTTGGTATTACGGTGTTTGTAGCCCATTCTGGACCAAAC  TTTTCAACTAATTTTTTTAAATTTTGGGTTGCAGCTTCACGAATGGCGTA  AACTTGGTCCACAAGCCATGTCATACATAATGTATTGAGCTTTTCATCAA  AGAATTCAACTCCTAATTGACCAGCTAGTAAAGGCATGTAGCGTATTATT  GCAAGCCGAACTCGCCATTTAGAATCCTCTGCCAGTTCAACGATTGCTGG  TAGTAAAGATTGACTCAATTGTTGAATACCAATGACTTCATTTACACAAT  CAAGGTTGGAAATAATGTTTAATCGGACATCCGGACATTCATCCTTGAGT  TGTATAAGGAAAAGAGGGAGAAGATGCTCCACAGTATTTTGTTTACCGAA  AATTGGAGATAGACCCATAATCACAGAAGCAAGAGCTGATTTAACATGTT  GATTATGATCAGCGACCAAATCTTTTATGCAGGGTAATATATTGTTCATA  ATGATAGTTTCTTGGCAACTTGGGGCAAGATTTTGACAAAAATCTCTGAC  CTTTTGTGCCGCAGCAGCTCGAACTTCAGCTTCACAATCCTTGAGCAAGT  TTTGGAATGCGGGGACAAGATCGGTCTTGGTAAGCTCAGAGCCTACAGCC  TTTTGAAGTTCAACGAATTTATCTGCAACCATGTAACGAACTCTCCATGA  TTTATCTTCAGCCGCTTGTCTTAAAATGGGCATAACTTGTTGTTCCAGAT  CTTCTTGGGGTAAAAGGGAGGCAAGAGAAACACATGCCTCACCAGCCAAT  AAACGAACAGAATCTTGTTCATCTCCAGCTAAATGTGACCAAAGTGATAT  GACTTCCGATTTAAGGTACTCTGGTTCTAAGACTTTGGCAAATTCACCCA  GTTTCGAGGCAGCTGCCCTTCTAACCATTGGAGTATCATCTTGACACAAA  GCTCGGAAATTGGTTCTAAGCTCTGCTTTGACTGGAGCTGACACTCTAGG  ATAACAGACACTGAAAAGACCACATGCTGAAGTTCTAGTTGTAAACCAAT  CACCACTAGAAAGTCTTCTGACTAGAGGAACAAAATGAGTCTCTAGATCT  TGAGGTGAATGTTGTTGCGAAATATTACGTAAAGAGTCAACGGCTTTTTC  ACGAACTATTGTCTCTTCAACGGTTGCCAATGATTCTAATGGAGGTAGAA  GACAGTTAACATATTCTACACCACCCACTAGTGGTGTGAAATTTCCCAAC  TGTTCTGCCAGTGCAAGAAGAACCTCATCCTCATCGTAGATGGTTTCAGT  TAAAAAAGGAATCAATTCTGACCTAGTTCTTTCAACACCTAACGCCAATG  AAATGGTCGACAATTTTTTGATGCTGTTAAGTCGGATCTGTACATCTTCA  TTTCTTAATTCATCTATAAGAACAGCAATTGGATAAAGAGTTGGATCATC  ATTAGGCTCATTAGCAGCCATAATTAGTAATGATTAAAAAGACGATTACC  AATGAAAAAAAAACAACAAATTAATGTGAGAAGAAAATATAAAGAGACAG  TCAAAATTAAATATAAGAAACAAAATTAATTCTCTCTCAAAAACG  >Unigene4391_All Similar to AFX [Nasonia vitripennis]  TGAGTGGTGGTGAAGTGTCCAGCGGTGAAATTAAACCAAATGGAAATTCA  AGTGATAATAGTGGTCATAAGAAAAATTCTTCTCGACGAAATGCTTGGGG  TAATATGTCTTATGCTGACCTTATTACCCAAGCAATTCAAAGTTCTTCGG  AGAAAAGGTTAACCTTATCTCAAATATATGATTGGATGGTACAAAATGTT  CCATATTTTAAGGATAAAGGTGATTCAAATTCTTCTGCTGGTTGGAAGAA  TTCTATTAGACACAACTTATCTCTTCACAATCGATTCAAACGAGTTCAAA  ACGAGGGAACAGGTAAAAGTTCCTGGTGGGTAATCAATCCGGAAGCTAAA  CCGGGTAAAGCTGCAAGACGACGGGCTGCCAGCATGGAGACGCAAAAGTA  TGAAAAGAAACGAGGTCGAGTCAAAAAGAAGGTCGAAGCTCTTCGCAATG  GTACTATTCTTGATGATAAAGCATCTCCTGGTTCGTCTGTTTCTGAAGGT  TTAGATATGTTCCCGGAATCACCCCTTCATCATGGTTTTCAATTAAGTCC  TGATTTTCGACCTCGAGCCTCGTCTAATGCCAGTTCTTGTGGTAGACTTT  CCCCAATCATAATTGAATCAGATTTACATGATGATCACGCTCCTTCCTTG  TCCCCAATATCCTCATGGAACTCAGATTTACGTTTATATAATACCAATGA  TGTTCAAGATACGTACACGGAAAAGTTGGTTGAAATGGCAAACACCATGA  AACTTGGTGGTCCTAGTTACAGTTCGAATAGTCCAAATTCACCTCAGTCT  CAAACACAACAGCAGCAACAATCATCACAACTTCATCATCATCTTCAGTC  TCAACAAAGTGGTTCATCTTCATCTCCAAGCAGCAATTCTTTGACCAAAT  CATACACCAATTTAAACTGTTTTGGTGCTACAGGATCAAATCTTACTTAT  TCAACTACCTCCATTTCTTGTAACTCTATAAATCAATCATCTGGTGATGG  ATCAGATGTTAATTCAAGTCCAAACGGACCAATCTTGTTAGATTGTAATT  CCTCTGAATCATATTTTACATCAACTAATACGATAGACAGTAACATCACA  TTGACCACATTAAATACAATTAACAACAATACAAGTACAAGAAGTCCAAA  CAAGCCAATAATTAATTCCGAGGCAACAACTTTTGGCGCCAATCAAAATA  GTGCCAACAGTTCATTCAGCTCGTCAAACATGTCACCCTCTCTGTCTCCC  AGTCAATCAATGGATTTACTTTCCAGTTCAAATCAAGTAAACAGTCCATT  GAGTAATAACAATAATTTCGGCTCAAAAAGTAGTCAACAAAATCAATCAC  AATCAAGCAATCGTCAATTAACCTCCCAAAATAGAGGATTTGGATCCACT  GGTTTGGATAATGATACTCTTCGTCGACTCAATAACAATTCAAAATTATT  AGGGTCACTTTCATCACTTGGAACCACAGCCTCCCAGGTGATGGGTCACA  TACTGTCCTTTAACAATCCATTGCCTAACGATTTGGATTTAAATATGGAC  TCATTACAGGGAGGCTTAGAATGTGATGTAGATCAAGTAATAAGACACGA  ATTATCCGTCGAGGGTAATCTTGATTTTAATTTTGAATCAATGCAAGCCT  CAGATGATAATTCAATCCAAGGATTAAATGGTACTTTTCGTTCCAGCCAA  ATGTCCAACATCTAAGTGATTCTAATATTGATTTGTTTTCTCTTCATCTT  TTTTTTGTTCGTTGTTATTTCAACACACAAATACACCTTAACACCACCCA  AAACGCCTTACTCTCTCTCTCTCTATCACACATACATAC  >Unigene6436_All Similar to cell division cycle 2-like 1 (PITSLRE proteins) isoform 2  ATTTGACCTTTGTTTTTCTCTTTCTTTTTCTTCTCTTTTCATTTTCTTCT  CTTTGGTCTGTCTTTTTACTCTTGTTCTTTTCTATTCTACTCTATTCTAT  TTGGATTTACGTCTGTCTGCATGTCTGCCTGTTCATCTCTATCTATTTAC  CTCTTTCTGGCCTGTCTGTCTGTCTGTTGGTTCTCTTTCTATCTTTTTGC  TCTCTCTTTCTCTCTTTTCCATCTTCACTCTCATTCTCTTATTCTCCCTT  TCTCTTATCCTTGTGTTTTTCTCTTTTCCATGATTCCCCTTCTCCTCTCT  GCCCTCTCACTTAAACCAAAAATCAAAATAAAAATCTAACAGAGGAAGGT  AAATTTCTGGCCTTGGATCT  >Unigene19946_All Netrin-1 isoform 1 [Macaca mulatta]  GCCTTTTACATTTCTTAAATTACTATTTAGTTTTTTTTGTCGCAATTGTTGACTGATTTCAATGATTGATGAATGATATGAGAGAGAGAGAGAGTGAGTGAGATGTTGATTGATTTAATCCGATCGATAATCTTAATCCTCTGAACAGATTCCACTTCGTTGTTTCCGTTGATATCGTCTAATCTTACGATCCCATTCCTCGGACCATTCGACTACCAGTGATTGTCGATCAGCAATTAGCCCTCGGGGCTCAGTNNNNNNNNNNNNNNNNNNNNNNTTCCTATAATGATGTAGTTTTCTTTGATTTTCATCTTAGGACATTTACATGCAAGATCTTGTACTGGAACCCAAAGTGATTCTGGTCCACGCCTTAACTTTGTTACAGTGTGTTTATAAATCTCTATTATTTGTATTGTGAATCGAATCCAGTCTCCAATTGTTTCCCTGGAGACAACATTAG |
| >Unigene24841_All E1A binding protein p300 [Rattus norvegicus]  TCAAGAAGCTAAGTGCTTGGTTATTTACTGTCCGAACATAAAACATAAACTTCGTCAACAACAACTTCATCAAAGACATCAACAGCAACAGATCTTATTGCGTCGTANNNNNNNNNNNNNNNNNNCAACATCAAGCTCTCAACGAGATTATAATTCATCACCAGCTCAGTCAACGCCTCAGACACCTCAGCAAACCTACATTAAACCGACTCCATCTAAACCTTCGATGCCTGGCACTCCTCAAAATACCGGCATCAACCATCAACAGTCGCAACCACCGG |
| >Unigene25239_All Slit [Platynereis dumerilii]  CCTTTTTTCCGGCCTTCAGAATTTGAATTTACTTTCACTTTATGATAATAAGATTCAATCTCTGGCCAATGGTACATTTGATTATTTTAAAAACATTGGTACACTTCATTNNNNNNNNNNNNNNNNNNNNNNNNNNNNNNNNNNTGGTTAGCCAATTATTTGCAAAAAAATCCGATTGAAACCTCGGATGCCCGTTGCACTGAGCCC |
| >Unigene11424_All Similar to Collagen alpha-2(IV) chain [Acyrthosiphon pisum]  CGACCAATGATCCCATTCCTTCGAAAGCCGTTCAAGAGGATGAAATTCGT  AAACATGTAAGTCGCTGTTCTGTCTGTGAATCACCAGCCAACGTTATGGC  CGTTCATTCCCAGGCCGATTATTATCCCGAATGTCCAACGGGCTGGAGTC  AACTTTGGATAGGATATTCATTTGCCATGCACACCGCGTCCGGTGCAGAG  GGAGGTGGACAATCCCTTGCATCCCCTGGAAGTTGCCTTGAAAATTTCCG  AAGTGCACCATTTATTGAGTGTCAAGGTGCCAGAGGCTTTTGTGCCGTTT  TTTCTAATAAATTGTCTTTCTGGTTAGCGATAATCGGTGAGGACTCACAG  TTTGAGAAACCACAAGGAAAAACATTCAAGCAAAGTCAACTTCGAGACAA  GATCAGTCGATGTGCCGTTTGTCTTAGGGATATACCCAATTGATTAACGT  TATTTAAATTAATCAAAAAACGCAAAACATTACATAATATAAATAAAACT  TATAAATACTATCAAATGAACAGTCGATAAATTGCTAAATGTTTATAAAT  ACGATTTGATTAAACTGGAGAGACCAAAAAGTTTTAAAATAATTGTAAGC  GATATACTCACAATGTAATTACAACTATTAAGCCCTGTTAATTTAATTGA  TAATTAGTTGGAAGCTTGTAAACTCCTTGTTGACCGTTTACCTTTTCTGA  TCCTATGACCCTCTTTCCAGCGATAATCAATTAAATTAACAGGGCTTAAT  AGTT |
| >Unigene12196_All Phosphoinositide 3-kinase isoform a [Panulirus argus]  ATATTTATCCTTACGTCGACATGCTAATCTTTTAATCACTTTGTTCACAA  TGATGCTTTCCACCGGTATACCTGAGCTTCAATCCATAGATGATATTGGT  TATCTAAGGAAAACACTACAAGTGGAAAGAAGTGAAGAAGAAGCTCTGAA  ATACTTTAACAATCAATTTTTCGAGGCTCACGGAGGTGCTTGGACCACGA  AGATCGATTGGTTTTTCCATAATATTGCCCATTTCAAGAATTAGTTTACA  CGTACTTTCAGTTAATTGTTATCCATTTTTTTCTTGTTTTCTTTTTTTTT  GTTCGGTGATCATTTCCTTTCGCAATGTCCTATCCATTCACATATTAATT  GTTTAACTAATTGTTTTTCCATATCTACACTCACAGAAACACACTATCAG  TACACACAACCAGCATTAACCAACTCACCTCTCACTCAAACAAATATAAA  ACAAATTTATATACATATTTACATATATTACTGACTAATTGATCTATTAA  TAGTATGAACCTGGTATTTGCTCATCTCGATTGTCCCTTAATACCAGAAT  TGTATAAATCCAAACTCAATCCCTTTCTCCCTGATTCCCATATCATGACT  AATCCCTTTTCTCAGATTCTGGAAGATATTTTCTAAATCACGATTAATTG  TTTAACCTTTTTTTTTTCCTCTTGTGTCTCAAAAGCATTTTACAAACACC |
| >Unigene13909_All Patched [Nasonia vitripennis]  CTGCTTTGCGATACTTTGTAGCTCAAACTGCCATTTTAATTACCTTCATT  ACCATTTGTTTGCTGACAATTTTTCCCGCCATAATGAGCTTGGACTTGCA  GCGACGTAAATCACGTCGATATGATTTATTTTGTTGTTTTAAAGTCAGAT  TATCATCATCATCATCACAATTAAAAGCACAATCATCTTCACAACACAGC  CAACAAAAACAAGAGGAAAATTATTCTCATCGTTCTCATCGTCATGATAG  ATACTCTAAAGTAAATAATCACCGTGAAAGAAAATCATCCTCATCATCTT  CAAGAAATTATTNNNNNNNNNNNNNNNNNNNNNNNNNNNNNNNNNNNNNN  NNNNTTCCTACTCTAAAACAAATGGATTCATTATCATCACCATTATCAAC  TAATTATTATTCTTGCAAACCATATTTATTTGACTCATCATCTTCACCAT  CACCATCATCATCATCATCACCATTAACAAATAGTAATAATAATAAATCA  TCACGATCATCATCCTCATCAACAACAACAACTAAATGTGCAACAACAAA  TAAAAATAGTTTCTCATCAAATGTTTCAACTCCCGTTATGCCAATTAGGA  CAATTAAACCTCCTCTTAATTACAATTTACCTTCACCACCACCAGCTTAT  TTTGGTGATATTGAATCTGCACCACCCTCAGAACCACCACCATCTCCCTC  ATTAGATGACTGTGATTCTGATAGAGAGGATGATATAGATGAGATGGATG  ATGGGATTCGTGAAGATGAGATGGTTCCACCTCTTTCCCATAGATTATCA  TTAAACTATTTTGTAGCCAGTATCTATATACCTTTACTACAGAGACGAAT  ATTTAAATTACTGGTGATGATTGTGAGCTTTATAATTTATTTAATTTGCC  TGTCAGGCGTTACCCAAGTCAAAGATGGACTCGATTTAACGGACATTGTC  CCACGTGGTACCAGTGAATATCGTTTTCTACTTAATCAAAGACAATATTT  TAATGTATACAATATGTACGCTGTTACTCAGGGTAACTTTGAATATCCCA  CCAATCAAGCGCTTCTAGTTGAATATCATAACTCATTTACCAGAGTTCCA  AGGATAATTAANNNNNNNNNNNNNNNNNNNNNNNNNNNNNNNNNNNNNGG  TTAATCATGTTCAGAGATTGGCTTCTTGGCTTACAGAATGCATTTGATAT  TGACTGGAAAAACGGCTCAATCAGTCAAGAAAGATGGTTTGCCAATGCAT  CAGCTGAAGGTATTTTAGCATATAAATTGCTCGTTCAAACTGGTAGAGTT  GATAATCCGGTGGACAAAAGTTTAGTTACCAAAGTTAAATTAGTTTATAA  TCGTTCGTGGTGTCATTAATCCAAAAGCTTTTTATAATTATCTAACTGCC  TGGGTTACTAATGATGCTATCGCTTATTCAGCATCTCAGGCCAATTTTCA  TCCTGAACCCCGTCACTGGATTCATGTTGCTTCAGATTTTGACCTGAAAA  TTCCAAAATCTCAGCCCTTAGTCTACACTCAAATACCTTTTTATCTGCAC  AATATGAACTCAACAGAAGAAATTACTTCAACCATTCAAGAGATTCGAGC  CATTTGTAAGAACTACGAAGACAAAGGTTTACCCAATTTCCCGACGG  >Unigene14076_All Similar to Nuclear receptor co-repressor 1 [Strongylocentrotus purpuratus]  GTTTTTTTCAATAGAAAAATAAATTGTTAGAATTTTTTTTATTATTGTTT  TAATTAATCAACGGTGATTTGTTAATTGTTACTGGTTTATTTAAAGAGAC  ATAACTTAATCAAAGTGACCAATATATGATGAGCACTGATTCCTTAGTGA  AAACAGCTTACAACCCTAGAGTGGAAGCTATTTCACCAACATTGCCGTGT  GAGGAAACGCAACCAGATTACAACTCCACATTTAGGACGTCTAAAGATGA  ATTGTTACAGAATATTGATAAAATTGATAGAGAGATATCACAAACAGAGA  CTCAGATATCCGAATTAAGGGAAAGAAAGAAGCAGCTTGAATTGAAAGCA  GCTTCACGAGATTCACTCAAAGATGTAGAAAATGTAGACAACTCTTCAGA  GACTCGGCAGCTTAGTATTGCTCAACTAATATACTCCGAGAACAGGCGTA  AAGCCCGAGAGTCACATGATATTTTATCTAAATTTGGTCCAAATTTTGAC  TTGCCTCTCTATCACCAACCTTCTGACTCACCAGTCTATCATCAAAATAT  TGAAAAGTTTAAAGCTTTTAAACCAAGACTGATTAAATATTTCAAGAAAC  GCCATGAAGAGAAGAAAGCAAATCAAAAATACCTTTTGGAAACCTACGAA  AGGCTAATGTCCAAATGGCTGAAAAAACTTGAGAAGAATGAACTTAATCC  GGGTAAAAAAGCCAAAGATAACAAGACTAGAGAATTTTTT  >Unigene17722_All Daf-12 [Strongyloides papillosus]  AAGGGAATCAATGTGATATCAAGTTTTACACAACAATCAGGTAAATCTGGTTAGATACCTGCAGAAAAGTTGGCAAACAATCAACGAAAAAGTGATTTTTTNNNNNNNNNNNNNNNNNNNNNNNNNNNNNNNNNNNNNNAAATATGATCAATCAATTAGCAACAATTAACTTTAGATGTGACAATTTGATCATCAACACACTAAACCTAATTAAAGGGAGAGAAAACTTAATGGAATTGAAGAGTTAATAATCTAATTCTAATCCAGGATAGTTAATTGTTGAAAGGATCGAAAAGTTGATTTTATCACCACGTTTTCTTTGCAATTTAATGTCAAACAATTCAAGTTCTTCCAATGATCCCAACAATCAACATGTTTGTCGTATTTGCTCAGACCGAGCAATCGGTTATAATTTCAGTGTCCTCACATGTGAACCATGTAAGGCCTTCTTTAGACGGACAGCGGACAAACTAGAGGAATTACAATGTCCGTTTAATAACGACTGTGATATTGATAAAATTACCAGGAAAATATGTCGCCGATGTCGTTTGGTAAAATGTCTTAAAGTTGGCATGAGAAAAGATTGGGCTCAAAGTGATGATGAGAAAAAATCTCGATTTGAAAATTGTACCAATTTGTCTCGTGGGTGTGGTAGTGTCAAAAGTTCTACTATCAGTTCAAGATCATCTAATTACAATTGCAAAGATAATTTATTACGAGATTTTAAAATGTGTAACAAATTAACTTCAAGGAGCTTCGTTAAAGATAATGATTCCATCTCATCTAATCGTAATACCTGGTCATCATTATCTCTGCCCACGCCTTCACC |
| >Unigene19644_All SNF2-related domain-containing protein [Polysphondylium pallidum PN500]  CCCCTGGTAGTCTAGTGTCCAAAACCTCAAGGCTTTTCTTCAACGCTTCATAAGCAGCCATAGGTAAAGAAAAAAGGTAAATAAAAGTGAGAGAGAGACAGACAGAGAGAAAGAGAGAAGGAGAGAGAGAGAGAGAGAGAGAGAGAGAGAGAGAGAGAGAGAGAGAGAGAGAGAGAGAGAGAGAGAGAGAGAGAATTCAATTCAATGTAACGTTAATATTGTTCCAGTAGGTGAACTTGTGGATTGGGTGTTGACAGAAAGATGAACAATTCTAGTTTAACATTATAATCTAAGAAAAAAACGATTGAATTAAATAATAAACAAAAAAAAGAAAAAG  >Unigene20916_All Similar to neurofibromin [Nasonia vitripennis]  CAAGGAGATGACAACTCGCTACGAAGAAGCTACCAATGTAAAAACGCTTCTCCGGGAGCTCTGCCAGTTCCTGGATATGCCAAACGACAATCCCATGGCCTCTCAACTCAAACTATTGGCATCTCGAGTGCTTTTTGCCCTTAGCTTGAATAATTTTAATGCTATGTTTAATCGTATATCAACAAGACTGCAAGAACTGGC  >Unigene21484_All Neurofibromin [Culex quinquefasciatus]  CATCGGAGTGTTTACAGAATCGGGTTAAATTGGGTAACCAGGGAGTCATATATTCAAGGCATAAATGTTTCAATTCAATATTTGATGCTCGAAAACCTTGAATACACTCTTCAAGAAATTCTAATGTTAAATGTGAAGCATTGGTTGCCAATTTTTCACTTATCTGTTTGATGAATATCGTATTGTTGGATGGAATACAGAGACCCGATGTTTCCAAT |
| >Unigene23300_All Phosphoinositide 3 kinase catalytic subunit [Xenopus laevis]  GGGAAAGATTTCCCCAAGCAAGGGGACAAGTTTCACCCGTTTTCTTGGTACGAGTTGTAACTGAACAAATTGAGATGGAAAGTCTTGCACTTCTTGGTAAATCGGCTAAACAAATGTCAAATTCAATCTCTTGGTCCCATTTTGGATTCCAAGGGTCAACTCTAGAAGTCTCTTTAGGTGGACAAAGAAGCTCAGCTCCATGGTAAAGGCCACATTTAACGAAAATCTTTTCAACCTCCGATACATTAACATAAGTTGCCCAAAGAATTTTAATTTTAAAATAATTTTCGAACCTCCATAAAAGTAAACATTGGCCTGATTCTAAAGCTCGATGTATATCTTTGGAACCAGGATCAACCTTTGAGAATGACGATGAACGTTTACTACAAGATGGAATGCGAAATTGATGATTCTCAATCTCCAGAGAGA |
| >Unigene26711_All Similar to neurofibromin [Nasonia vitripennis]  GTCAAAATTGGCACGTAAAAAGTCATTGAAAGATAACATGTGCTGCTCTTTACTAAATTCAACATTATTTGCAATGTTTTGTAAAATTTTGGACATTAACATCAAACCTCGTTTCACTTTGTTATTAGGTTGNNNNNNNNNNNNNNNNNNNNNNNNGGAGATACAATAGCTGGATTGATGAAACGTAAGAAAATGACAGTTCCAACTGCACTTATATTGTGAGGAAAATTGGGAAACCTTTTGTTTAATACTTGGTACAAACAGTGACACATGGATCGTAGTTGAGGCGGGAAACTTTCTGCTGATGAGATGATCGCATTGAATACTGTTTGTGTGAGATTCAAAAGATTTTGATGATTTGACTCAACAGTTTCACCGGAATCCATTCGTGCTGCATCAATTTCGTAACTTATATCATTTGGTGTTTCAGCGAGTGGTTTAATTAAAGGTTCAAGAAGTGCTTTAAGGTATGATGTTCCATAGATTTTGAAACAAAATGCCATTATTTTACTACCTAAACTGTTACCACGGAATAGAGTTTGCATGCAATCAGAGACTTCAACCTCTTTGTAAAACATATTCCAAAGTAACGGAGAGAGTAAATGTTTCGCATCAAATAGGGTTACAAATAC |
| >Unigene2973_All Nuclear hormone receptor family member nhr-48  CAGCTTCACGGAATTGAAAGGCCAGTTCGATGATATTTTCCCGGATGTGG  ATCGTTTCTTGAAATTCACCAATTTTCATCATTAGATCATAAAAGTCACA  ACGAGAGTTACAAAATAAACAGCACCGGAATTCAAGGTAACGTTTCAACA  GGTAAACATAATTGAATTGATGGAGCCTAATAATGTCAGGATTCTGCAAA  TTCGGTGTGTCTGGATTGAATAACATGATAGCCATAACGAGCATATTTGC  TTTGGTGTCGGTTCGCCAGTTTGGCCTCAGGCTTTTCATGAAACTTATGT  AGGCGTTATAAGTTGAACTTGCGAAGCTACGAAGTCCACTTAGCTGTAGA  ACGACGGGTGTTTTTGGTATTTGAAGTGATTCTGTTTGAATGTCGATTAG  AACTGTAGCTCTGAACAATAGAATATCGGCAAAGTTTCTCTTAATGAGAC  ACATTCGATCTTCCATTGATAGCGACTCAAAAGTCGAGACTGTATTAAGA  GAAGGGACTAATCTAGTTAAACAACATTCGATGACATTGAAAAATCCTGG  GATATCAAGAGCTTCAGGGAATTCGCCAGCGGAAATGTTACAAATGGACG  TGGAGACGTTGACCCATTCGTCGAGTTTTTCTTGTTCCAAGTGATTGAGT  GTAACTCGAGGGGAGGAAAGTTCAGTGCGAAAACTGAGCGGCATAAAATT  GGAAGCGATCATCAAATGGTGCATTTTCCATGCCCATGGTTTCTCATCGG  ATTGATTAGGTTGTTGTGAGATTCCTGAACAAACTTGATTCTCACAATTG  GTAATGTAAGACGTTGAGGTGAAATACTCTGGGTGTGCACNNNNNNNNNN  NNACACGTGTTCCAAGCTCCATCACCCAAATTGAAACATTGCTGAGAGCA  AGTTTGAAAGATCGAATCGGTTTCCATTGCAAGTTGTTGTTGATAATTCT  CATCGATTGAAACAGTTTGTTCATCAAGATCCTCTAAAAGTCCATGAAGT  GTTTGATCGTCAAGTAGATTATCAAATACTGCTGATAACTTTTTTCCTTC  ACTATTCCCACCGGCATCATCTTCATCAGCTGATTCACTGCTGTGTGAGA  TGTGATGAGGTTTAGTGCCAATGTAACCAGATGTACCTGGAACGGGTTCT  TTGTCCTTCCCTTCATCTACTCTTTTATCTTCAAATCGAACCCATTCCCG  TTTCATTCCAATGGCAAAACATTTTTCCAATCGGCACTTGCGACAATGAC  GTCTTGTGATCTTATTCATCTCACAATTATTATTAAAAGTACAGATGAAC  TTCTCTTTTTTATCGGCATTTCGACGGAAAAAAGCCTTACACGATGCACA  CAAATGATAACCGAAGTACCGACCATTTGATTCATCGAAGCAAATCCGAC  ANNNNNNNNNNNNNNNNNNNNNNNNNNNNNNNNNNNTGGAATCATTTGGA  TAAGATTTTCTTCCCGGCGATCAACAGAAATCATTAATTACTTTTTTTCG  ACCTG |
| >Unigene31325_All Peptidase C13 family protein [Brugia malayi]  CAAACAACAAACCAAAATAATGTTTTCATTCTTAGCCATTTTCCCAATCCTTTTCACCCTTTCAGCTTCTACTCCTTTGGCTGATTTGTTGGATTCCAAGTCAAATGCTGGTTACAATATCCAAGCTGTACTGGTTGCTGGGTCAAATGAATACTACAACTATCGTCATCAAGCCGATGTTTGCCATGCCTATCAAGTTCTGAGAAATCATGGAGTTCCAGCTGAAAACATTATTGTCATGATGTACGACGATATCGCCAATGATCCTGAAAATCCTTACCCTGGTAAAATATTCAATGCTCCTAATGGCTCTGATGTTTATGCTGGTGTATCAAA |
| >Unigene31510_All AGAP003140-PA [Anopheles gambiae str. PEST]  GCTGGTAATCCAGTATTTTATTTCATCTTCCGACGATACAAGATCAATGAGATTAACGCTGATTTCCTCCTTTATCATGTTATCCTAACCCTGAGACCTTATTGTCATAAACCTTTTGAACTTGTCGTCGATTTCACTCACACTTGCACCGAGAATCGATTTAAGACTGATTTTCTGCAAAAATGGTTTGCCGTTCTACCCGAAATGGCCTATGAGAAGATTCATTCAGCTTATATTTACAACTGTAACTCGTGGGTTCGTGAATACACCAAATTAAATGATCGAGTTCTCGCTCCTCTCAAAGGAAATCGTAAATTAATTTTCCTCGATTATCCTCAAAGGTTAAGTGATTTTATTGATCCTGATCAGCAA |
| >Unigene31588_All Vitellogenin receptor [Dermacentor variabilis]  GTTAACAAAAATACAGGCCATGAATTGAAAGACATTTTACGTTTACATGAAGGCGGTGGTTTAGGTGTTCATGTTTCTCATCATTCAATGATGCCATCAAAACGTCGTCGTAATCCATGTTGGGCTTTTGAATGCAGTCATTTATGTCTTCTCAGACCTGGTAATAATTACACTTGTGCATGTCCTGATCATATGGAACTAAATGAAAGCGGTCGTCGTTGTGTTTCGAAAAATCGTCCATTCCTTTTGGTGTCAAATGTTGATACAATCTATGCAGTAAATTTCGGTGTCGTTGGACAACAGTCAACTATGAAGGTTAATTCACATAGTTTTCTAGTGGGCGCTTTAGCTTACAATTGGCGAAAAAAGATGATCTATGCTTTTGATCTCG |
| >Unigene31746_All Phosphoinositide 3-kinase isoform a [Panulirus argus]  AAAAAAAAAGAAAACAAGAAAAAAATGGATAACAATTAACTGAAAGTACGTGTAAACTAATTCTTGAAATGGGCAATATTATGGAAAAACCAATCGATCTTCGTGGTCCAAGCACCTCCGTGAGCCTCGAAAAATTGATTGTTGAAGTATTTCAGAGCTTCTTCTTCACTTCTTTCCACTTGTAGTGTTTTCCTTAGATAACCAATATCATCTATGGATTGAAGCTCAGGTATACCGGTGGAAAGCATCATTGTGAACAAAGTGATTAAAAGATTAGCATGTCGACGTAAGGATAAATATGCTAATCCACAAAGCTCGGTAAACTTTTCAAATTCTTTACTCTTTTTTGGATTCTCAGAGCCTTTTGAAATTACACATAAGAAATCATCGGTTAATACAAAAGGAACACGTTCTCTATTGATACCAAACTTTTTCTTAAAATGAC |
| >Unigene3294_All Similar to neurofibromin [Nasonia vitripennis]  AAATGTCAATCTAACTTGGATAATGCTGAACTTTTTGTCAATTGCCTTGA  GGCTATGGTAGAAACTCATTTACCTGTTGATGAGGGAGATACTGGTGACA  TGATGAGCTATTCTAGTGGCTTATGTGTATCTACTGCATCTAATTTATCA  ACTTCCCTTTCAAGTTTAGCAGTTAGTTCACCAACAGATAAAGATGCCAA  AACAGCGGATTATCATCATATTAATTCTAGTTCATCTGGAAAATTGAACG  ATTATCGTTCAAGGCATGCAGCACAATTAAGGAAACAAAGAAGTCTCAAA  GTTAAAGATAGCGGAAATGGTTAAGGAGAAAATAACAATAACAACAAAGA  TAATTAACCAGCCTGATTGTGTGTAAATTTATTGTAAATAAACGTAACGA  AAATGAACCAAAACAAAGCCAAGCCAAAAGAAAGATTATCCGAAAAGAGA  CAAAACAA |
| >Unigene6825_All Similar to neurofibromin [Nasonia vitripennis]  CCTCAGCGGAAAAGTGTAAAGTGTTAAGTTTTTCAGTGTTACTTAATGAT  GTTTATTATGCTTCCGAGATTGAAGAGGTTTGTCTCGTTGATGATAATCA  ATTTACTTTAACCATTGCCAATGAAAGTGGGCCACTTTCCTTTATCCATA  ATGATTGTGATACAATTGTCCAAGCGATAATACATATTCGTACTCGTTGG  GAACTCTCACAACCCGATTCGGTTACCGTTCATACCAAAATCCGACCAAA  AGATGTACCCGGTACCCTTTTAAATATGGCTCTTCTAAATCTTGGTAGTT  CTGATCCAAACCTACGAACTGCAGCTTATAACCTTCTCTGTGCATTAACG  CAAACCTTTGACCTTAAAATTGACGG |
| >Unigene6664_All GE14764 [Drosophila yakuba]  CAAATTACCTATTCATTTGAGATAATTGAGTGGACAATCAATGAGAGAGC  CAGTGATTATTATCGTCAAGTGATTAAACCTTCATTAGAGCCAAGTGAAT  ATCTTTTAATTGGATTGTCACTTAAAACTTTACTCAATCAAACGGTTTCA  ACAAAATCGATTCTTAAACCGTTCACAATTGACCCGTCAACGGGAATTGT  TTATCTGAACGCTTTACTTGATTACGAATTGATTACCAGTTATCGGCTTA  AAGTGACCGCAACTGATTCAGCCTATTTTGGTTCTCGAAACACTTCAATC  ATGATTAACATTCACGTTACCGATGTTAACGATAATCCGCCTTTGTTTGT  TGGCCTAAGGGACGATGGACGTTTAATTATGGAGACACTTGAAAATAATC  CGATCGGCTCAATGATCGGGATCGTCGCTGCCACCGATTTTGATTCACCT  CGTTTCTCACAGTTGACCTATGAAATTTTGCCTGAACTTGATTCGAGCTA  TTTTTCCCTTGGTCTCAAAAACGGTCACCTCACATCTTTGATCTCATTTG  ATTATGAAACCAAACGAACCTACTTAATTAAAGTTGTCGCTCGAAATGAT  CGTAAAATTTTCACAACGNNNNNNNNNNNNNNNNNNNNNNNNNNNNNNNN  NNNNNATTTCTACCTAAATTTAAATCACAACGATTCACCTTTGATTTGAC  CAAATCAATGACCGCTGG |
| >Unigene12605_All Similar to CG5482-PA isoform 1 [Apis mellifera]  TAACCGGCAGATATTGAAGAAAACTCTAGCTGAAGGTAGGAAGGAACGAG  CCAGGCCTGAGAAAGGCTGTCGTGTTAAACTTAATTTGACAACCAAATTA  AAAAGTTCTGGTGCTAAAATTGATTCTGAATGTTATGAAAATATCAAGAT  ATTTGTCGGTGATTGTGATATACACCATGCCGTGGATTTGGTTGTGCCTC  TTATGGACGATGGTGAAATCGCCAATGTTATTGTTTCTCCTCGGTTTGCT  TATGGTGACAAAGGTCGAGATCCTGATATCCCACCTAATGCTGCTTTAGA  TTTGACGATTGAAGTTGTTTCAATTGATTGGGTCGAAGAATTAAATGAAA  TCGATGCCATGGAGAGAATCAAAATAGCTGAAGGTAAAAAAGAAAAAGGT  AATTTCCACTTCTCCCGAAGTGAATATTCAAATTCTATTACCCTTTATAA  ACGAGCTATTGAATTTCTTGACATCGATCCATCGGAAATAAAGGATGATG  ACGAGAAGGAAAAAGTATTGAAAAAAATATCCGATCTGAAGTCCAGTTGT  TACAATAATTTGGCTCAAGCTCA |
| >Unigene13795_All Hypotherical protein [Schistosoma japonicum]  AGAATTTGAATATTCACTTCGGGAGAAGTAGAAATTACCTTTTTCCTTTT  TACCTTCAGCTATTTTAATTCTCTCCATGGAATCGAGTTCAGTTAATTCT  TTAAACCGTTCAATTGAAACAACTTCAATGGTCAAATCTAGAAGAGCATC  AGATGGGACATCAGGATCTCGACCTTTCTCACCATAAGCAAACTGAGCAG  AGACAAAAACATTGGCGATTTCACCCTCATTCATATGAGGCACAACCAAA  TCCAAGGCATGATTTATATCACAATCACCGACAAATATCTCAATATTTTC  ATAACATTCAGAATCAATTTTAACACCAGAATCTTTCAATTTGGTTGTCA  CATTAAGTTTAACACGACAACCTTTCTCGGGCCTGGCTCGATCCTTCCTA  CCTTCA |
| >Unigene14135_All Amyloid precursor protein [Manduca sexta]  TGTTAATTTGGCTGATGCAGCCTCATCTTCATCTTCATTAAATCAACAGA  ATTCAATCAATGGTGTTCCCGAGGTGGTTTTAAGCCATTTCCAACCGATG  GTTGCTATGCTTTGTGGTCGTGGTAAATTTCACAATCAATATTTGGATGA  AAACAAACGATGGATTTCTGACCCTGATCCCAAAGCGGTTTGTACAAAAG  ACAAATTGGAAATTTTGGAATATTGTCGCAAGGTTTACCCCAAAAAGGAT  ATTCGGAATATTGTTGAATATAGCAAATACATGGTTGTTGAGAATTGGTG  TAAAGTTGGACAAAAGTGCGGTGGTCGTCATTTCATCAAACCTTACCGAT  GTTTAGAAGGCCCATTCCAAAGTGATGCTCTTCTTGTACCTGCTTATTGT  CTCTTTGATCATATTCACAATGGATCGATCTGTCAAAGCTCAGAATATTG  GAATCGTACAGCTGCTTCAAGTTGTGCTGAGAAACGTAACATGAAACTCA  AATCATTTGGAATGTTACTCCCTTGTGGAGTTGGAATATTTGGTGGTGTC  GAATTTGTTTGCTGTCCAGCTTGGTCAACAACTACATCTCCACCAGAACT  TGTGACTCTTGGAACCGAAAAGAAACAATTGGAAAATCAAATAACCTACA  AAGACGCTGAAGATAATGATTCAGATAGTGACGAAAGCCAAGACGATAAA  GATGATCGAAATTATGACGAAGATGATGAGTATTATGACGATGAATATCA  AGAAGACGATGAAGAGGATGACGAAGAATCTGCTTTGAAGGCAAAATCAA  CCAGTACAACAACAACAACTACAACTACAACAACTACTACTACAACCGAG  AGACCCATTGATCATTATCTGAGTCACTTTGATTCCAGTCATGAACATGA  TTCGTACAAAGCTGCTCAGAAATCACTTGAAGAATCTCATCGAGATAAAG  TAACCAAGGTTATGAAGGAGTGGAGTGAACTGGAGGAAAGATATCAAGAG  ATGAAATTAAAAGACCCAAAGGGCGCAGCTGAGTTTAAACGTAAGATGAC  ATCTCGATTCCAGAAGACAGTTGAAGCCCTGGAGGAAGAAGGATCAGCTG  AGAGG |
| >Unigene22151_All Similar to class II bHLH protein ASCL4 [Taeniopygia guttata]  TGATGATACTAGTCAATCTCAACAATCACCAACCGGTGACCCATTTAGACCTGAGTGTCAAATACCTTTACCAGGTACGATGGATCAATACCCGTTTATGGATAATCGAACTTTTGTCCGACGTCGAAATGAACGTGAACGTACAAGAGTTCGTAATGTCAACGAAGGATTTGAAAGGTTAAGACAACATTTACCTACACCTAAAAGTTGTAAAGATAAAAGATTATCAAAAGTAGAAACACTGAGAG |
| >Unigene22406_All Protein patched homolog 1-like [Ailuropoda melanoleuca]  ATGGGAAACCGATGAGGCAACAAAATCCTAATCCAGAGGCGATGGCAAGGGCGATAAGTAGGACACCAGAAATACCAAGGGCACTTTGTGAGTAACCAGGATTACCAAGATTAATACGAGATAAACCGGTAAAAAGTAACAGTAAACAATATCCAATACAAAGATGGATAATATTGGGTATGGAAAAATTTTTCATAATATCAAGTAGGGCAACATTGGTAAACTTTTCAATCTTATAATCGGTTATTGAGTTTTCCTTAATGT |
| >Unigene23384_All Transcription factor Ash2 [Brugia malayi]  ATCATCAACAACAACAACAACAACAAATCAAACACCATTCATTGGATTATCAAAGTTTGGAGATGTAACAACAACAATTGAGAAAAATCATCTTAATCATTCCCGTCGATTAGTTAATGATAATCCTTATCCTGGTTTGGCTCAAAGTTATGCTTCCATTGTGGAAAATTATTCCACTAATTGTAATAATCGACGTACAAATAGTAATTGCTTCAATTCGCCTGATTATCGATTGACACTTCCAACGGCGATTAATCCAAGTTCACTTGATAGTGCGAGTTTCATTCGACGTCGAAATGAACGGGAAAGAGCTCGAGTTAAAAATGTCAACGAAGGTTTCGATCGGCTGAGAAAACATTTACCGTTAACTCAAAGTCAACGGGAAAAACGCTTATCGAAAGTGGAAACACTTCGAATGGCGATTAGTTATATTAAACATCTCGAGATTCTGTTAACGCCGAAGTAGTAACACGATGGA |
| >Unigene25005_All AGAP001038-PA [Anopheles gambiae str. PEST]  GCTAATTGTGATAATATCGCCAATTTGAAACCAATATATTCTTATCCGAATGGTTGGATTGGTGTTTGTGAATCAATTTATCTGAAAGATCGTGAAATTAAACGTGTTTTCTGTGGTCATCGAATTATTTTATTACGATTGTCATCTTCCAATAGAAAACCTTATGCATTGGATGCTTATTGTCCTCATTTAGGAGCTGATTTATCGGTGGGTGGAAAAATAGTGAAAAACTGTGAGACTGATTGTATCCGTTGTCCATTCCATGGATGGTCATTCAAAGTTACTGATGGTCAATGTGTCGATGTTCCTTATACCAAAGACCGAAAACCACCAAACGGAGTAGCAATTAAGACATGGGAATGTCTAGAACTCAATGGGTTCATTTATGTTTGGCATCATAACGAAGGTCAAAAGCCAACATGGGTCCCTGAAGAAATCAAAGAGATCACAGTCAACAAATATAAGTATATGGGCCGAACTGAGCACATTGTCAACTGTCTAATCCAGGAGATACCAGAAAATGGACCAGATTTCGCTCATCTCAATGAAGTACATGCACCGTCATTTCTTTGGGGAGGAAAAGTACAAAAGAACAACAATATTGTGATTGATTCCATCAGCCA |
| >Unigene3956_All Similar to Eukaryotic translation initiation factor 3, subunit 2 beta, partial  CTTGTTATCAATCAATTGATTGTTTTGATTTTCTTCTTGTGATAATTGTT  TTGTTTTCGCCGTTGAATTTGATTGTAAACCTTTTCCGTGTATAATCAAT  GACTAATGTAAATTCAAATTCAGAGGAAACCACAGGCCACGGGCCTCCAC  CGGTTCCTGCGAGGCCTTTTCACAATTCAATCTCTCGCCCTTGGAATTCA  GGTTATTCAAATTATGGTTTTGGTGGTAATTACAATCGTTTCAATGGATT  TACTGGTCTAGGTTATGGTGGAGGATATGGTGGCTATGGTGCTGGTGGAT  TCGCAGGTTATGGTGGAGGATATGGTGGATATGGTGCTGGTGGATATGGA  TCAGGTTATGGTGGATTTGGACCATCAATTGGAAACGATTTTATCAGAAT  AGCGGAAGAAACTTGTGGTCAAAGTTTCCAATCACTTGAATCGGTTGTCC  AATCGGTTTCATCTGTAGCCATGATGCTTGAGTCAACCTATTTTGCTGTT  CACTCTTCATTCAGAGCAGTTCTTGGTGTTGCTCATCATATTTCATCGCT  TAAAGATCAACTGTCACAATTTACTAATCAAATACCAATTGTAAGATTTT  TACTCTCAGTGGTGAAAAAGATTCTCTATTTTCTTGGAATCATTTCCTCG  AATAGTTTAATTGACCATGAAAGAGCTTGGAGAGAAGCCACTTCAGCAAA  TATACGTTTAAATGCTGACGGAACATTTAACCCATTTTTAAGTGGAAAAG  ACCCTGTAACAAGTAGATCGTCATCATTACCGATATTAATTTTTTTCGGC  CTAGTATTAGGTGCACCTTGGATGATTTGGACATTACTTAAAAGATCAAC  ACGAAAAGTGTCCCATGTAAACACTGAATGGTCAATCGGTAAAGATGAAC  ATTATATTGGTCAAGCTCTTTATCCATTTACAACCAATTCTCAAGGTGAA  TTACCCTTAACTACCGGACAGAAGATTCTAATTGCTCCCAAAGATATTCA  ACCTAAAATTGGTGATTGGTTATTAGCTGCAATCGATGGACAAATTGGTC  TAGTTCCCGCTAATTATATTAAAATAATTGAATATAAACCATCATCTAAT  CAACAATTAGATGATCAAGATAAACAATCTCAGGAAAAACAACAGAAATT  ATGATTTTTTCTTACCTGTTGTTGATAAACACAAAGTTACCTTATCATCA  TAATCATCATCATCA |
| >Unigene9107_All Cell division cycle 42 [Ciona intestinalis]  ATTTGAAAATTACACCGGAATTCAGGATTACCATGGATCAAATTATCAAT  TGGCTCTTTGGGACACTGCCGGTCAAGAAGATTACGAAAGACTAAGACCT  CTCTCTTACCCAGGGACCAATGTGTTTCTATTATGTTTCAGTGTTGCCAA  TTTAAATTCCTATCAAAATATTACAACTAAATGGTTTCCAGAAATCAGGG  AACATGCACCGAAAGTCCCGTTCGTTTTGGTTGGAACCAAAAGTGATCTC  AGACCTGATGATAAATCCTCATCTCCTTGGTATCGATCAAAGTGCATCGA  CTTGACATCTCAATCAAATGGATCTGAGGAGACTTTTATTACCACGGACA  TGGGTCGTAAATTAGCTCGTAAACTGAATGCTTCCTGTTACGTTGAATGT  TCAGCTAAAAGTCTTGTTGGAATAGAAAATGTAATTATGGAAGCTATCAA  AGCTGGAATTGGTGACGAAGACAAGAAACAACGATGCATAATTATCTAAA  TTTACATGACGATTTATAATTACCGATCAATCGAAAGGATTCCAGAAAAC  ACAACGAAACT |
| >Unigene12220_All Similar to Very-long-chain specific acyl-CoA dehydrogenase,mitochondrial precursor (VLCAD) [Monodelphis domestica]  GATAGAACCAGCATCCGATCCCGAACTTGGTTCAGTTAAAGCAAAAGCGG  CCAATTTACGTCCAGTCGCTAGATCGGGAAGATATTTACTCTTTTGAGCT  TCATTTCCATACAATAAAACGCCTTTGTATCCAATTGACTGATGAGCCCC  CAAGGTAATCGCAAGACCTAAATCACTTGCACCCATGATCTCCGCGAGAA  CAGCGTATTGAGTG |
| >Unigene14536_All Histone H3.3B-like [Bos taurus]  CCCATCATCCATAATTTAATATGGCTCGGACAAAACAATTCTCATCAAAT  GCAAGTAAAGTACAAAAGGCCAGAAAATCAACTGGTGTTGGTTTTAGTGG  TACTCCATTGGCAACCAAATTGGCCGCTAAACGTGGTAAAACCATTGGTA  AAGGAAAGAAAGATATACGTGACATGATTAAATTTAAACCTCGTCGATAT  CATCCTGGTGTTGTTGCTCTCCGAGAGATAAGGAAATTACAGAAACGAAC  TGATCTTCTCATCCCGAGAAATCCATTCTTACGTTTGGTCAAAGAGATTG  CCATGGACTATTCTCTCGCTCAGTTGAGATTCCAATCAGCTGCATTAACT  GCACTACAAGAAGCGGCCGAAGCCTTTCTGGTAAGACTTTTCGAGGATAC  ACAACTTTGTGCCATTCACGCTAAAAGAGTGACCATTTTCCCTCGAGATA  TTGCTTTAGCTCTCAGAATTCGTGGTGAAATATTTCTTCTTGGTTCTCGT  CCAATCCGGTAAACCCAATTCATCCCAAATATAAATAATGTATTCATAGC  CTTTACCTTAAACAAAACCAAAATACCTTTTAATCATAACAATCTTTATC  CAATTTCCAGAGAATCAATCACAAAACAAAACAAAAAAAACCATTCAAAT  TATAATCA |
| >Unigene1673_All Ribosomal protein rpl12 [Eurythoe complanata]  TTTTTTGGTGATAAACCAAGAGGACCAATTTTAGGAGCAAGTGATGAGGT  AGCACCAACTTCACCACCAACAGCTCTAAGGTAAACAATTTTGATTTCCG  ATGGATCGGCTTTTGGTGGCATGATTGTTTAATTTAAATAAACTAAATTA  ATAGCAAATTAATAAAAATACAACAAGAAAATACAATTAAATGTGATTTT  CTCAAAACTCAACTCAACAAACGGAAG |
| >Unigene17369_All Small nuclear ribonucleoprotein G [Amblyomma americanum]  AGCTATTTATTAGTTAATCCATCACCATGAGTCGAGCTCATCCTCCGGAATTGAAAAAGTATTTGGATAAACAGTTAACTCTTACCCTGAATGGAGCTCGTAGGGTTTCGGGTATTCTTCGTGGTTTTGATCCTTTCATGAATCTGGTTCTGGATGAGGCAACAGAGGAAACCAAAGAATCCAGAATTCCATTGGGAATGATTGTAGTTCGAGGTAATGCTGTTTACTTGTTGGAATCTGCTGACAGAATTTCTTAATGATCATGATCCTGGTTGGAAAAAAAA |
| >Unigene17804_All C. briggsae CBR-NCX-2 protein [Caenorhabditis briggsae]  CGAGGATGGAACAGCAAATGCCGGTTCTGATTATATTGGTGTTTCGGAGACGATTGTTTTTTTTCCTGGAGAAATCCAGAAACAGGTAACGATTCATGTTATCATGTTATCGACGATGACGTTTTCGAGGAAGATGAACATTTTTACTGTCGATTGTCAAATCCTCGATATGTTAATAATGATGGACGAGAATTGGGAAAGGGAG |
| >Unigene20015_All 7SK snRNA methylphosphate capping enzyme-like [Xenopus (Silurana) tropicalis]  GAAATCAACTGGTTTACTGTTATCTAATTTAATTCTTTTCACAGACGTGGGCGATTCGTCATCAAATGAAGATTGACTAATTAGATTTACTTTAGTAGATGGATCAGATTGGGTTGAAGAATTGATTGGAAGAGATTGAGATGAACTTGAAACAGCAAATTCAGTTGATTCTGTAGAAACGGTTGTCAGATTAGATTCAATTGGTGACTTGGAATGATCGTTTAATTTAATTGCTTCACCATTTAGATGATCCATTCCATTGGGAAAATTTTTCTCCTCGCCTTTAGCATCAATTTCATCACATTTTCCATTCTCTTCACCATTCAACTGGACATTTATTGGTTCAATTGATTGACAACAATTATGTTTACTAATCACATTTATATCCAATCGTTTCCTCAGAGAATCTCTAACTTTCGGATCACTTTTTACATAGTTTTTAATGTTACGTTTGGCAGCTTCAATAAGATTCTCATCTATATCAATACCAATTATTTTACAAGGATTAAAATCTCTTGCAATGGTCAGTGTAACATGACCAACATTACAACCAATGTCCAGAACATGTTGATCCTTAAACCAACAAGACTCAAAGCAGCTTATCCTTGGATCTATGGTAACATTTTGATTCCGATAACCATAATATCGGT |
| >Unigene22606_All Ribosomal protein L3 [Caenorhabditis briggsae]  CAGCCTCAACAACTTCCTTTTTATTAACCTTTGAACCAGGTTTGTCTACTTCACGGACGATGTGGGTCATACCAGCTTTGTACCCAATGAAAGCTGTAAGATGAATAGGTTTCGATGCGTCATCTTTGGGGAAGGCACCAATGCGACCGCGATGACGACGACTTCGCTTTTTCTTGAATCCCATGTGCCCATGACGAGGCGCTGAGAATTTACGATGACTCATTCTTATAAAATTAAATTAACTTATCACTAAAAAACACCAACAACTCAACTTATACTT |
| >Unigene29233_All Hypothetical protein CBG08717 [Caenorhabditis briggsae]  TGACCGAGAGCAACGTAGATACAGAAAAGTTTCTTCTTGTTGTCGTCAGATTCATTGAAACGCTTTTGATTGATAATAGTATCAATAGCAATGGCAGTTTTACCAGTTTGACGATCACCAATGATCAACTCTCGTTGACCTCTACCAATTGGTACTAAACTGTCTACGGCTTTGATTCCACTCAACATAGGTTCCCTTACACTGATTCTAGGAATGA |
| >Unigene3059_All Ribosomal protein L6 [Ixodes pacificus]  ATTTAATTTATTTCCAAAACTTATTTTTTTACTGAGTCAACCATCAAAAC  TTCATTTTATGAGGATACATGTTATTACGAAGGCAGAAATATGAACCGAG  ATAGCCAAACATTAGCTTCTTTTCAGGATGTTTACGAATAGCTTCAATTA  TTTGTTTGTCAACTTCAACTTGATCTTTACGGTGTTGAAGGTTTGGTTTA  TAATCGGCAGATTTAGTCTCGAAAATGTTACCTGCACCTTTCTTTCCCTT  AGCCTTTTGTCGAGCAAATTTACGACTTTTGAAATAT |
| >Unigene31674_All C. elegans protein Y18D10A.23, partially confirmed by transcript evidence  GCCGCGTATGGATTACGACATGACTGTCGGAACTTAGGCCATCTCTCCTCGAGAATGAGCCAACATTTACCGATCAGAATGCCACAGATTCCAGCGTCAAGCCAACACATGAGTAGCAACGGAAGTCCGCTCCATCCCGTGCGGTCAAAGGAAAAGGGCAAAGCCAAGACCCCACAACCTGCGTTCTCACCGGCGATAAACAAAATAGCCACCAAAGTGCTCATCCCATGTTTCTTTTTGATTAAATTTGATTCTTCTCCACCGCTGTAACTATCAAATGTTGAATTTGTATCAAGACTCGTCTTAGATTCAGTTTTATTTGAAGATTTTAATCCGTTAAACTTTTTATTTGGCATTTTAATTATCACAATTAACACAAAAACCTAATAATAAATTGAATTGGAATCACTTAATTGCA |
| >Unigene31885_All Adaptor protein complex AP-2 mu1 [Glossina morsitans morsitans]  CATTCTTGCTATCAAGAGAAATTTTCTCGTTTAATCCAAATTTACACTCAGGCATCCCGCTTAAATAGGTTTTCATAACCACTTTTCCAGCAACATGAGCACTCAAAACTTGTCCTTGGTGTGTCATTAAAAGGTTGACATACTCCATAACATCGAGAAAAAGCTCATTACGACGGTATTTGATGCCTTCCCGACGCCATCCTATTTGACCAGTTACCTGGGATGTTATTTGTGTTTGTTCTTCTTTAGAAGAAGATTTAACGCTTGCAGATAAAATAAACGATTTAAGTGTATTGGCGTCCGTTATTTGTGGATATCCAAAGTCCATCAACTCGTCGAGAACCTCAAGGATTAAAAAATAATTAGCTTTAAAGTTTTCTTCATTGAGTTTACCGAAATATGTTTGCATGATTTCGCATGAACGGATGAGGATCTCGAAAGCAAGAGCCGCGTTCACATTCTCCTTCGTGTAGACAACGAACCACATGTTGTTACGCTTTATATGGAAATAT |
| >Unigene3698_All Similar to H3 histone  ATTAATGAGAGAGAGATAAATGAGAGAGATAGATTTTGTATGATGGTGAT  GAATCCACCGAAAAAAGTAGAGCTCAATTAAGCTCGTTCTCCTCGGATTC  TTCTAGCCAACTGAATATCTTTCGGCATAATAGTGACTCTTTTGGCGTGA  ATAGCACAAAGATTTGTGTCTTCAAATAGACCAACCAAATAAGCTTCCGA  GGCTTCTTGAAGTGCTCCAATGGCCGCAGATTGGAAACGCAAATCAGTTT  TGAAATCTTGAGCAATCCCACGAACTAACCGTTGGAATGGTAATTTTCGG  ATCAACAGCTCAGTTGACTTTTGGTAACGACGAATCTCTCGTAAAGCTAC  TGTACCTGGGCGATACCGATGAGGCTTTTTAACACCCCCTGTTGACGGAG  CGCTTTTACGAGCTGCTTTGGTAGCCAATTGTTTACGAGGAGCTTTACCA  CCAGTTGATTTACGGGCAGTCTGCTTGGTACGCGCCATGGTGAGTTGCGA  AAAACAAACAAAAACAGAGTAAAGTTCAGAGAGATGTTAAAATTGATGAG  ACACAGAAATGAAAGAGCTTTGATGAAAATTGAATGAATGAAAATGAGAA  AAGAGTAAATACTCAACAAAGATGGCGAATTCTCATTCAACTGCGC |
| >Unigene4017_All AT15141p [Drosophila melanogaster]  AGAGAGAGAGAGAGAGAGGAAGAAAGAGAGATAGATAGAGAAAAGAGTTA  AAGTGAGAGAGAAAGAGAGAAAGAAAGAAAGACAGAGATGATAAAGAAAA  GAAAGAAGAGCTTTGAAGAAAACATTTTAAGGTGGGGTCACTGTGAGCGC  ATGTATTTTTTTTTTCTTTAATTTCATATTTTTTATTTGGATGTCATCAT  CGTCACAAATTCTTCATAATTTACTTGACCATCGCCATCAATATCAGCTT  CTCGAATCATTTCATCTACCTCTTCATCAGTCAATTTCTCTCCCAAATTA  GTCATTACATGTCTCAATTCAGCAGCAGAAATAAAACCATTACCATCTTT  ATCAAAAACACGGAAAGCTTCACGAATCTCTTCTTCAGAATCTGTATCTT  TCATTTTTCTTGCCATCATTGTCAAGAATTCGGGAAAATCAATAGTACCA  TTACCATCGGCATCAACCTCATT |
| >Unigene6789_All X-box binding protein 1 [Mytilus edulis]  GATCATTTTCTCCAAATTATCAAGTTTTTCTTTTAAAAATTTATTCTCCA  TTTCCAAGGATAAAATGCGCTGTTGATTTGACTCACTTTGATCCAACATT  TTCTTAAGATCTCCATTTTCTTGCATCAGCCGTTTATTGACCGCACCCAA  AGAAGCCAATTCAATCTCCAATGTAGACATAACTCTTTTCTTTTTATCCC  TTGACGTTTGAGCTGATAGACGATTTTTAATTTTTCTCCTCATCATTTTT  TCCTCCTCCGTAAGGTGATCAAGTTTCTCTCGTTTCCTTTTCCGTTTACC  ATTGCATTCATCACCAGTAATCAATGACGAGTCAACAAACATAATGGTTG  AATAAATTTCTTTTTTTTTCTATACAATTACAAATGACC |
| >Unigene9005_All Similar to Y4C6B.2a [Ciona intestinalis]  TAAGCCATTTTACCAATGGCTGGATAAGGATAACGACAATTTTCCCGAAA  CTCAGGCCATCTCTCTTCAAGAATCATCCAACATTTTCCAATTAAGATAC  CACAGATTCCAGCATCAAGACAACACATTATCAGTAACGGTAAACTGTAC  CAACCGGCCTGATCAAAGGCATAAGGTAAGGCCAGAATACCACATCCTGC  GTTTTCACCGGCGATGAATAAAATGGTCACTAAAACTCCCATTCCATGTT  TCTTATTGACCACTGAGCCCGTTTCACCATCGTCGTAC |
| >Unigene9127_All Pyruvate kinase, muscle, b [Danio rerio]  CCAGCCATTACAGCAACAGCTTGAATTGGATATTCTCCTTTGGCTGTTTC  ACCTGAGAGCATGACACAGTCAGCACCGTCTAGAACAGCATTAGCCACAT  CAGAACTTTCAGCTCGAGTGGGTCGAGGTTTTTTCACCATACTTTCTAGC  ATTTGGGTGGCACAGATAACCGGTTTACCCATCATGTTACATTTAGCGAT  CATCATCTTCTGAGCAAGGAAAACCTTCTCAGTGGGTATCTCAATACCCA  AATCACCTCGAGCAACCATAATACCATCAGATTCAGCGATAATTTCATCA  ATTTTTTTAACACCTTCATGATTTTCAATTTTAGAAATGATCATAATGTT  TTTACCCTCTTCACCTAAAATTTTTCTGATCTCTCGTACACCGGCAGCAT  TACGAATAAATGATGCAAATACCATGTCAACTTTATTCTTAACACCAAAC  AATAAATCAGCTTTATCTTTTTCAGAGACAGCGGGTAAATCAACAAGTGC  CCCAGGCAAATTAACACCCTTCTTGGATCCTAATAGACCACCGTTTTCAA  CTTCACATTGTAAATA |
| >Unigene3466_All Ribosome associated membrane protein 4 [Argas monolakensis]  TGTCCTCCTTTCTCTTTTTTTCTTAAACAGCTAACATAATAATAATAATC  TTTGATATTTTTTTTTCTTCAAATACTTGGAGACAAATACAATTTAGAAA  CCATATCTGATTGATTGGATAATCTGGAAAATAGCTGATCCACAAACGAC  AAAAATAAACAGAGCCAAAAGCCAAGGTCCAACTGGATATTTATCATCTT  GTCCTTTATTGGTTTTCGGAACATTCCCTCTCAGAGTAACATTTTTAGAA  GCCTTTTCATTAGCTACTCTCATTCTTTGTGTATTAACCATGATTCCAAA  TTGTGAACAAGTAAATTACTTAAAATTACGAGATTAACCGATGGCTTATT  AAATTAAAATAATCGAGTGACAATCATCAATAACAATTTAATTACAATTA  ACTCTTAAAGTGAAGA |
| >Unigene11430_All Similar to ENSANGP00000006233 [Nasonia vitripennis]  AGCTTGATGGAGAGGAGTATATCCATGCTGAGTTGTTATATCAACATTTG  CTCCCGCCTGCAGAAGCAGTTTGATCATACCAACAGCACCATTGTGACAA  GCGACATGGAGTGGAGTGTATCCTTGTTTCGTTTGAACATTAACATCAGC  TCGTTTCTTAAGCAATACTTGGGCAACAGCGACTCTATCACCCTGAGCAG  CAAGATGAAGAGGTGTCAATTCAAGCTTGGATTTAGCATTAACATTTGCT  TTGTGGCTAATCAGCAATTCACCCATGTCAGTGTGTCCCTCTTGGGCTGC  TAAATGAAGCGGAGTGAATCCAGCCTTAGATTCAGCGTTAGTGTCAGCAC  CATATTCCAATAGTGTTGTGGCGATTTCAATTTGCTTCTTTTTAGCGGCA  ATATGTAAAGGTGTGTACCCATTTTTAGCTGTTGCATGAGGCGATGCTCC  TTTTACGAGTAATAGTATTGCAACATCAACAAAATCATAATGAGCAGCCA  CATGAAGAGGAGTAACTCCATTCTTTCCTTGAGCATCAACAGGAGCTTCC  TTCTCAAGCAACAGTCCAGCAACGGTGATCTTGCCGTATTTAGCTGCCAA  GTGTAATGGAGTAAAGCCTTTCTTAGTTGTCGCTGTTAAAGATGCTCCTT  GTTCAAGAAGATAAGCAGCAACTTCCCGATGACCTTCCTTGGAAGCGATA  TGAAGAGCAGTGTATTGATCTCTGGTTGCGACATCAACCGAAGCTCCTCT  GGTCAATAATAGTTGAACGATCTCCGTGTTATTCAATCGAGATGCAATAT  GAAGAGCAGTTTGATCCTCTCTTGCAGTTTTATCAACAAGAGCTCCATTT  CTCAATAATATTTTAATTATTTCCGATTGTGAGGCTCTTGCAGCCAAATG  AATGGGTGTTTCTCCTCTCACGGTGGCTCCATCAACATCGGCTCCATTTT  GGATGAGATAAATAACAATATTGATACATCCCATGAAAGATGCAACATGC  AAAGGAGTTAATCCAGATTCCGTGGTAACTTCAATAGATGCACCATGTTT  AAGGAGTAATTCAACCACCTTGATACGATTCTTTTTACATGCAATATGTA  ACGGTACAAATCCATTAAGAGCTCTCGCATTAACATTAGCCTTGTGGTCA  AGTAATAGTTTAGCCACTTTAACATGTCCACAATGAGCAGCGACATGTAA  AGGTGTCAGGAAATCCACTGTAACATCATCTACAGGTGCTTTATAAGCCA  AAAGAGTACGAGCTGAGTCTATATGATCACCTTGAGAAGCCATGTGAAGA  GGTGCTAATCCATTCTTAGTCTTGGCTGAATAAGGTGCTCCTCTTTTTAA  GAGTAAATCAACAACCGGCTCATGTCCACTTCGAGCAGCACAATGTAAGG  GAGTAAGTCCATCCCTTGTAGCTGCATCAATTTGAGCTCCACGGTCCAAT  AGGAGTTGAACCATATGAGCCTTTCCCCATTTAGCTGCAACATGTAATGG  TGTAATTTTGTGCTTTGCAGTGAAATTAATGTTGGCTCCTTTATCTAATA  AGAGTGTACCAATATTTTCATTGCCATAATGTGCTGCTATATGAAGAGGG  GTAAATCCACTTTTAGATGTGACATCGGGCTTTTGGTCACTTTGGAGTAG  TAATGCTGCCGCTTTACAATCATCTTTTTTAGCTGCAATATGTAAAGCAG  GAAGTCGAACTTTTCCTTTGGTATCATTTTCCAAGAGAATGGTAACTACT  TTATCGTGACCCTGTTGCAGGGCAACGGCTAAGGGTGTAAAACCATCTTC  CGTTGCTAAACTTTGGTTAGCTCCATGGGATAAGAGATATCTGACCACCG  TGTCATGGTTTTCTTGAGCTGCCATGTAAAGGGGAGTAAAACCATTGAGA  CTTTGTGCATTAACATTGGCTCCATTTTCAACCAGAATCTCAACAACCTC  TAATTTACCACCAAGTGAGGCTATATGAAGAGCTGTATTTCCTTTATTAG  TTCCAGCATTTACATTGGCTCCTCGTTTTAATAGTTCTTTAACCATTTCC  GAATGTCCTTCTTTTGAGGCTAAATGAAGAGCATTCATACCATTCATGTT  ACTAGTATTAATGTCAAGGCTTCCATTTAGATATTCTAAAACCTTCTCCA  AATTACCGGCTCGGGCTGCTCTTAGGAAACTAGAGCTTCCCTCGTTCATT  AACTGAGGTGAAGTTAATTTAACCATAATTATGGTTAATCAAAAAGTTAA  TTTACACAAAATTGACAAATTTGGTTCACATTTAAAAGAGAAAGAAAAAG  AGAAAAAAAAATTCTGATAACAGTTAGAATATGGGAGAGCCTTAGAGAGA  GACAGATAATAAC |
| >Unigene14587_All Similar to ankyrin 2,3/unc44 [Tribolium castaneum]  CTAGTGCTGATCTTTGGATGGTTTCTGATTTAATATTATAATGGACATCG  GTAAATTGTTGTTGATAATTATTAATAACAATTGGTTCCTCTTCCAAGAT  TATTTCAGGTAATTTGATGTTTAAATTGCATATTGGCACTTGTGGAGCTT  CACCTCGAGTTTTTCGTGGTTCTTTCATGAAGGCAATTTTACCGAAAGGT  TCTTGAGATGAATCTCGAACTCTTATTAAAAAGGGAAGTCTATTCTCTCG  GAAAGCTTGGAAGGTTAGATTTAATTGTTCACCCGATTTGGTTACAGGTA  TCAGGTTACCAGCAAATTCGAGGTATTGACATTTACCCTCTAATACCTCG  ACATCACGACTCTTGGCTACTTCGGTAAATTGTTCTTGATTTTCAAGAGT  TTTCTCTTCCTTATCATCAGTCATACAGAATATTCTCATTTGAGCTTCAG  AGGGATCATGACGCTTTGAGAAAACAACAAATTTCGACATGAAAGGAACT  GCAGTCGCCTCACGGTAAAGGTCAGTAGCAAATTTGGTCGCTTCATTGAT  CTGACGACAATCCATTAACCAAAATCTTGCTGAGACCGTAGTTGTAAATG  AAACACATTGATTAACAAAAGTAAGCGGAGTTGAACCAGTGACATCTTCC  CATTGAGCTTTTGAGGTTCCTCCTG |
| >Unigene19432_All Similar to AGAP008678-PA [Tribolium castaneum]  CTCTGGTTCGTGTACAATGAGAGTTTGCTGGCGTAAATTGGGATCATTCAGGGGAATCGGTGATGAACTGATGAAACGATTTGATGGAGCAACTAAAGTACGTTTGGTGGCAAAGAAAACAAAACCTCNNNNNNNNNNNNNNNNNNNNNNNNNNNNNNNNNNNCAAGTAGACGAGATCTTGTTTTTCTTGAACCATCGCTCAACTATTGCGAACGAAATGAGACACTCGGAATTCTTGGTACACAGGGTCGTCGTTGTAATGCTACAAGTTTGGATACTGATTCATGTAAATTACTCTGTTGTGGTCGAGGATATCATACAATGGTGAGAAGTGTCGAGGATAAATGTAACTGTAAATTCATTTGGTGTTGTCGTGTTGATTGTCAAAAGTGTATGGCACAAAAAGAGGAACACTTTTGTAACTAAATCAATCACTTGGACAACAATAATCATCACAATCATCACGAATACCTGGAAAATTACCTCATCATTTCACTTTTCCTCTTCAAAAGAGCAACTAAACTAATCCAATTGTCATCATAATCTTTCATCAATAGATTTCCCTATGAAACATGAAGATGATGATGTTAATCCCACCCGATAACCAAGACTGATTTAATATCCATTAATTGAG |
| >Unigene28920_All Similar to GA14061-PA [Nasonia vitripennis]  CTCGTGAAACTCGGCAATTCGTTGTTTCATTGTCACCGTGGGCTAAATACACCATTCGTGTGATAGCTAAGAACGACGTTGGTAACAGTCAACCATCAGAACCATCATCAACATGTGAAACTAGTCCTGATAAACCTTTCGATCATCCGAAAGGTGTATCTGGAAAAGGTACAGATAAGCAAAATATGGTGATAAGATGGAAAGTCATGC |
| >Unigene13652_All Similar to low density lipoprotein-related protein 1B [Apis mellifera]  AGTGTATTTGTGTGTGTGTGTGTGTGTGTATATCGTGGTGGATTTTCAAT  TTTTGAAGTCTTTCTTGTGAATGAGTGAGTGTATTTGTGTGTGTGTGTGT  GTGTGAATATCGTGGTGGATTTTCAAGTTGATCTCCTTTTTTTCTGTTAA  TCATAATCAACGTATCCTGCACAATCTGTTTCGTCACTATTATCCCAACA  ATCAGTTACGTTATTACATTTCTTTTCTATTGCAATACATGAAGTATTCT  GTCCACAATGAATTTCATTTTCACCACAATCACAGTTTTTCTCGTCCGAT  GAATCAAAACAATTCATATGGCCATCACATCTTTTAGATAAAGGAATACA  TTGGGTATTTAAACACTGCCATTCACTTTCTAAGCACTTATAAGTCTTTT  CACAGACACAATCTTCAATTTGAAGAGATTGATGTTGAGATCGGGTTACT  TTGATACAATTAATATCATCAACTCTCCATTGGTTTAACTTATCATAACA  ATAACGGGTCCTTGAATGGACTCTCTCCTCTGAACAGGAGCCTGCTTCAT  TTTTACATGTTGGTCCCCATTCGGACCATCTCTCCCACTTATATTGATGA  TGTTCAGGCTCCTCTGGATCACTATTATTACTATTACTATTATTTTTATG  ATTTTTATCAATAACACTTTGATCACCCTTTGATCACCACCATCCACTTC  AGATCCATCACTGTTATCACTTGATGATGATGTTATTGTTGTTAGACCAG  TTTTATTATTATT |
| >Unigene26705_All Similar to ENSANGP00000015354 [Nasonia vitripennis]  CTCCTGAATTGCAGAATAATTACTGTATTTATGACTTACGAAAAAGGTTCCTTTCAATGTACAATTGTTTGGATTGCGACGTTCTGCACGGAATAACATATCATAGATGGCAATGTTGTTGTTGTTAACGGCATCGTCGAAAGAGATTATCACCATTTGTGGGGTTGCTTTCGGTTCTATTTTACCTGGAATTTGTGTGCCATCAACTGAACAGAAACAATTCGGTAGTAAACATGTTGACGTATCACAAGATGGAGCACCATTAGGATCGAGATCAGGCGTACAGATGTTTTCATCTGAACCATCGACACATTGACGTTGACCATCACAGAATTGTATTATTGGTATACATTCACCAGAACCACAGGCAAGTTTACCATCTCCACATTTACCATTGTTAAGTTGAGGCATCGCTTTCGTCGTTCTTTTCATTTGTTTACAATTTTTCACGTTAAAACGCCAGTCGCAAACCTGACGTTCGACATCGAAAACTAAGCCTGACGTACATTGAAGTGGCATTAATTTACCTTCGGAACATCGTGCCACTTGACGGCAATTATCTTCTGAAAGACGGAAATAATCATCACCTGTATGGTCTTTGCATGGTGTATTGTCTTGATGAAGAT  >Unigene14393_All C. briggsae CBR-PSA-1 protein [Caenorhabditis briggsae]  TTATTTTTGCGATTAAATTGGATTAGACCTGAGAAAAACAAAAAGTATGT  TACAAAAAGCGGAGTGATGAACACTTTATGAATCTTTATGATGTCTTTGT  TGATGGACGTATATCATCGATCAGCCTCAACACTAATTACCGCCATTTTT  TGTTTTTTTACTAACTTTTCTTTTTTTATTTCGTTTCCGTTGCTGTTTTG  GTTTTGATGGTGGTTGTGAACTTGGTGGTAAATAGTTGTAACCTTGTTGA  AGAGGTAACGATAAATGTTGTTGATAATGTGATGGATGCTGTTGAGGATA  TTGACCATGAGGTGGTTGATGGTAAGGATAAGGAGGATACTGATAATGTT  GAGCACCAGGATAACCAGGACCAGGACCAGATCCTGGGTGTGGTGGTGGA  GGATAATGAGACCCTATTGCTGGATTTCCTGGATGAGGATAATTGTTATA  ACCACTTTGCATCATTTGCCAATTCAGATGCTGAGATTGTTGGTGAGGTG  GTTGATACATTTGCTGTTGAATTCTAGGAAGTAAATGACCACCCAAATTC  CCATAACTGCTCGGTGGCTGTTGAGGTGGTTGTCCAAGATGATGATTGGG  ACCATAATCATTCATGGCTCCTTGAAGTGGATAATTTTGATGCTCAAGGT  AACCTTGTCCAGGTGGTTGATTATGACCTGTATGACCCTCATATTGAACC  GATGTGGTGGACTGGGGATAACTACCTTGCAAATTAGAATCAGCTGAACC  AGGAAAATAATATTTAATAACCTTCGAAGT  >Unigene31835_All Nuclear Pore complex Protein family member (npp-9) [Caenorhabditis elegans]  CACGTTCTTTCCACTCTTTTGTATCAGTTACGTATCGGTAGAGCTTCGCGCGCTCACAGAAAACCGTTTCTTCTTCTTCTTCACCAGATTTAACATCTATCAATTCAGGTAAAGGCACGACTGGCTTGAAATCAACTTTCGGTTCGTACTCGGGCACTTTTTCATCATCATCTTCTTCCTCATCATTCTTGGGTGTTGTACTTGTTTGTCCAAAACTAGTTGCCCATTGGGATGGTTTGAAAAAGTTCGAGTTTTCCTTTATCGCATCACTGAAAAAGTTGCCACTGGATGGTGTATTAAGTGTGTTTCCAAAGCAGAATATCTGTTTTGCAGGTTGTGGTGGTGTCACCGTTGTCGTTGATGTTTGCGGTTTCGATGCTTCATCCAATTTGAATAACGATTTATTGATAGATGACGTCGCTTTTGCATTGGAATCAGTTTCATCATCACAGCCTCTACAACCTGGACATGGCTTAGCTTTC  >Unigene11268_All Cniwi [Podocoryna carnea]  ACTGGTGAATACCCACCACCGGTACTTTTATGCCCCGAGTTGTGTTGCTT  GACCGGACTGACCGATAGAATGAGAGCTGATCACTTATTTATGAGGCAAG  TTGCTTCATCAGCGAAAGTCAACCCAGCGGGTAGAATTGCTCAATTGAAA  CAATTCTGTACTTCAGTTAACACTAATCCGCGTGTTATTGATGAGATGGG  TCGATGGCGAATGAGCATGGACCAAGAATTGGTCAAGGTAAAAGGTCGAG  TTTTAAGTGATGAAGTTCTAATTATGGGTAATAATACAACATCAACAATT  AAAAATGGTGAATTTTCCCGAGAAATGAGAGGACGAACTTTATACAAACC  GATTAACTTTGAACGTTGGGCAATGATTTTTACCGCTCGAGATGAAGCTC  TTGCCACTGAATTTAAAAACTCAATGGTTCGTGTTGGTGGTCCAATGGGT  ATCAACTTTGGTAATCCTCGACCATTTAAGATTGAAAATGATCGAGCTCA  AAGTTATCTTACCGTCCTTAAAGCAATACCCACTGAGGCTCAGTTAATTG  TTTGCTTGGTTCCAAACAATAATAAGGATCGATACGATGCAATTAAAAGA  TTCCTATGTATTGACCATCCAATACCCTCTCAAGTTGTCACTTGTCGTAC  TTTGAACAGAAAAGGTGGTATGCTTTCAATTTGTACCAAAATCGCAATTC  AAGTTGCCTGTAAAGCGGGAGCCGAACCTTGGTACCTCGATATACCACCT  AAAAAGATTATGGTCATTGGATTTGACACTTATCATGATTCCTCGGTTAA  AGGTCGATCAGCTGGTGGTTTTGTTTGCACAATGAACTCAACTCTTACTA  AATATTATTCGAGGGTAGTTTTCCATGAAAATCGTGAAGAAATGTCCGCC  AATTTGGGTATGCACACTGCTAAAGCGCTCTCGGTTTATGCTGAGGCTAA  TGGTGGTCCACCGGATCGGATAATAGTCTATCGAGATGGTGTCTCAGAGG  GTATGATTCCTCATATTTATGCGACGGAAGTGGCCATGGTAAGAAAAGGT  ATCCTTCGGGGTAACGAAGGTAAAAAAATACCGTTGACCTTTATTATTGT  CAACAAACGGATAAATGCTCGATTCTTTGCCGTCAAGGGTGACAGTTTTG  TTAATCCTCCGCCAGGAACTATAGTCGATAGTACTATTACTCGTGAAGAA  CGTTACGATTTTTTCCTGGTTAGTCAATCGGTAACCGAAGGTACAGTTGC  TCCCACAATGTTTGACATTTTGTGCGATGAATCAGGCTGGACTCCAACAA  ACCATCAAAAATTGGCCTACAAATTAACTCATCTTTACTATAATTGGGCT  GGAAC  >Unigene11426_All GK25452 [Drosophila willistoni]  CTTGTTGTTGCTATGTTAATGTTCATTTCTATGTTTACACTTTTCTGTTC  TGTTCGTCGAAACTTTTAAAACATTGTAATCAACTTTTAACAAATATGGT  GACCAATGGTATACCTTGTGTGAGTGTTTGTGTTGATGGTGATAGTAATG  GTGATGGTAGCGGTGGATCAGGTTTACCTGGTGAACCAGCAAGTGCTAAG  TCACCAATTGTTGTCAACGGTGATTTAAATGAAGGTGGTGGTGGTGGTGG  TGGTTCAGGGTGTTCATCAGTTAAAGTACCCACACCAGTGGTTTCACCAG  TTAATTTTTCCAGTGGATCAAATTTACTTCAGGTGGGGATTCCGTTTCCA  ATAGGATTAGGTCATGATCCACCTTTGCACCCATTTTATTCAGTTCACCC  GAGAATTGGATTCGATTCAAAGGAAAATATGGAAGCGTGGAGATCACTTC  CACCTTATGCCTCAATGTATGGTGGATATGAAGGAGCAATTCTAGCTGGT  GGTTATCCGATGCTAAGAAATGGATATGATTTGAATGGTTCGAGACGGAA  AAATGCCACTAGAGAAACGACCTCAACTCTAAAAGCTTGGCTTAATGATC  ATCGGAAGAATCCTTATCCAACTAAAGGAGAAAAAGTAATGTTAGCAATA  ATCACTCAGATGACTTTGACACAAGTGTCGACATGGTTTGCCAATGCTCG  TCGAAGGTTAAAAAAGGAAAACAAAATGACTTGGTCACCTCGAAATCGAT  GTGATGACGATGAAGATGAAGAAGATGATAATTGTAAAGAAGGTGATTAT  GGTTCTCTAAATGAAAGTAGATCTAAAGAACCCGAACTACTTGTTGATGA  TTATGATTCATCTTCAGATTCAATTGAAAAGAAACGATTCAAACGGGATG  AAACAAATTCTGAACC  >Unigene11442_All Similar to cytoplasmic dynein intermediate chain isoform 2 [Tribolium castaneum]  GTTGTTTTTTTCTGGTTCATCTTTCCAACCAAATGTTTCAATTATTTTCT  TAATTGTAATTTAATTGCAATTTTTTAATTTCTTTTCTCGTTAATTAACT  TTTTCTTTTTCCTTTTTCATACAAAGAATATAATTTAATTATGTCAGATC  GAAGAGCTGAACTAGAGAAGAAGAGACAACGTTTAGCTCAGTTACAAGCA  GAAAAGGAGAGACGTCAAAGGGAAAAAGAAGCTGCTCTTGCTGAGCAAGC  TAGTGCCAATGTTGCATCTTCAGGGAAAAAAACTGGAGATCTCAGGCGAG  AAGAGGCTAATGTCATATTTGCCGAGCTTGGAATTGATCCTGTTGGTGAT  ATTACACAATTAGTGACAAGTCAACCAGCAATCGAAGTGACCAATGAAGT  TATTACCTTAGAACATCCAGTGGAAGAGCCAAAAAAGAAGACGATCAATT  TAACATTGGTTAATATCAATGAGATTAACATTCCTCCCAAAGAAAGTGTA  ACTTACAATAAACAAACACAGACAGCGGATTCAGGAAGAGATGCACATCC  TGTGGACTACTATGTGCTCACCTATGATGACAAATACGCTCA  >Unigene12664_All Similar to corto CG2530-PA [Apis mellifera]  TGGTCATTTGTGTACGATGTCCACGTCAATGGGGTCGATTTAGCGAAGCT  GCTTGACCGTTGGATGTTATTGATATTCACTGAATTTGAGTGGTGAGATA  ATTGAGTTTGGTGGTGTTGTAGGTGTTGCTGTTGTTGTAGTGGTTTGTTG  AGGTTGTTGGTGGTTTTGATGGAGTGAATCGTGAAGTACATCGGTGAATT  GCCGATGATTGTTACGAGATTGTTGTTGTTGTTGTTGCTGTTGTTGGTGG  TGGTTTTGTTGGTGCTGTTGGTGTTGTTGTTGTTGGTTGAGATGGTGATG  ACTTTGAATGTTTTCTGATTGAAATCTAAACTGATTAGAGTGATGATTTA  GATTTGCTTGTTGTTGTTGTTGCTGTTGTGGTGTTGATGGTGGTGAAGTG  TGACTACTATTTAGATTGCAGATTGTTGTCTCTGTTGATGAGCTGTAACA  ATCATCTTCCATTTTGGTTTTAATTGTTGGTGTAATTGAGGAAGTTCCAA  TATTTAAAGATGATGTTAAGGGTGAGGAAAGTTTACAGGGTGAGACATTA  ATACATGACGAGGTGGAAAGTGGGGTTATTTTGATGGATGAAAGTAATGG  AAGTAAAGTTGAGGAAGAGAGTGGAGGAGAGGAAGATATTGATAACTTGT  AGCTCGAAGAGGAATCACTGTCCGAACTCGGGATACTCGGTCCATCTAAA  GCGGGACCAAAAGGTGTATACGAAAATCCTCTTGTATTCATGAGGAATAT  TTTACTTCGATAAAGGTACGAAAGGGAGAAAAC  >Unigene12720_All Neuronal pentraxin with chromo domain-like [Danio rerio]  CTTTATTGCTGTTGATTCAGCAGTGTTGAAATATTTGATACTTTATTCCT  GCAAATCTAATCTGTAATCCTTTATGGTGATTTCGTCCTGGTGTTACGTC  TGTTAACGAAAATCTCTGTTGATTGAGTAGTTGAATCACTATAACCATCT  CATTACTACTTCTAAGCCTTGCAGAAGTTAGGTAATCATGAAAAGCAAAC  AGTTGAATAAATCATCGGTTACAATGAACTCAAATAAAAGTGAAGCTGAT  ATTATAAGACGAGAAAAAGACATGGAATTATCTTCGGTTGGTGATCGTGT  ATATGCAGCAGAATGTATTCTAAAAAGACGATGTAGACATAAAAAAGTTC  AATATCTTGTTAAATGGAAAGGATATTCACCTAGACACAATACCTGGGAA  CCTGAGGAAAACATATTGGATGAAAGGTTAATATTAGCCTTTGAGCAAGC  TCAAAATGAAGGTTACAATTCTTCCTCGGGCCATCATCATCATCAGCAAC  AAACAACATCAAATTCATCGCATTTACACCATAATCATCATCACAACCAT  ACACATAAAGATAAGAAAGAGGATAAA  >Unigene12729_All Similar to cell differentiation protein rcd1 [Tribolium castaneum]  AGACGTAGATACTCAAAGGGTCGAGTCTTGGAATTGGATGATGATGTGGC  CTGAAGAAATGGATACAAATATAACGGGATATTAGCGGCCAGAAATTCAC  CTCGGGTTTCTTGGTGAGAGGCGACACATTGAAGTAAAGCAAGAGCGTTA  CAAACACGATTTGAATGATGAGGTGACAAAGTCGCCGGTGTAAGGGTCGG  GTAAACACTGACTATTTCGGCGAGAAGAGCGGAAATGGCACCAAATGAAT  TCCATAACATGGTCGCCAGGTCAGGGACAATTTCCCGTTTCTTTCCCAAT  TCGAGTAATGCGTTTTCACGAGTCTCGACACTAGTTAATTGTGAGATCCA  TTGGAAAACAAGCTCCTTCTCAAGAGGTTGATTATCTTTACTTTCATCAA  TTGAAGGTTTTCCGTTGACTTTAGTCTCCATTGACCTCCGGTGATTATTG  GAAAATAATTAAAACAATTAAAATTTAAAAG  >Unigene12733_All GATA zinc finger domain-containing protein 10  CCTTCTCTCCCTGTATTCTTGGCAATGATTGTAATTACTGATTCCATTAA  TTGTGTTAATTATGGATCAACTTTCAAGCCTTTTTGGACTTTGTCAACAA  CCATCACAACAACAACAACAACAACAACAATCTACATCAACATCAACCAA  TGATAAATTTAATATATTTGTTGATGATTCTGCTGCTTTTATATCCACCT  CACCATCATCATTATCTAAATTGTCACCATCAACTATACCAACAACATCA  GCAGTAACAACAATATTTGGATCGCAAACAATTAATACATGTCCATCATC  ATCGTCACCGACAACAATTAATTTATGGGATTTAACAACAAACCGGAATG  AAATTTATCCAGGTGAAGTGTCAATATCATCAACAACAACAACAACAACC  ACAACA  >Unigene12867_All Fringe [Schistocerca gregaria]  TGAATTGGAAACTTTTCTTACAACCAATAAAAAATGGTTCTGTCATTTTG  ATGATGATAATTATGTTCATGTGCCTAGTCTAGTTAAACATTTATCTAAA  TATTCACCAAATAAGCCTTGGTATCTTGGTAAACCCTCGATACCCTCACC  ATTGGTTATAGTTGATCCAGAAAATCAGAGAGAAAATATTTCTTTCTGGT  TTGCCACCGGAGGAGCTGGATTTTGTTTAAGTCGTACTTTAGCCAATAAG  ATGATACCCTATGCAAGTAATGGTTCCTTCATGGAAGTGGGTGAAAAAAT  TCGTTTACCTGATGATGTTACCATTGGCTATATAATTGAACATTTACTTG  GAATTAATTTAACTGTTGTTGAACAATTTCATTCTCATCTGGAGCCAATG  AAATTCATTAGGAAGGAAATTTTAAAAGATCAGATTACCTTTAGTTACTA  CGAGATGAACGTTGTTGATGTAAATAGTGTTAATGATACGATAGATAATT  TAGAAGATCCAACAAGATTTAAAGCTTTATACTGCCATCTTAATGGATGC  TGATAATTGAATTTATGGCCAATTTGCCCTACGGTTACTTCGTTTTTATT  TTTCCTTCTTCTAATGTTTCTAATTGTTACCACAAGATGGAGCCCCTACT  ACGACTTTCGATCCACGTGTTTTTCATGTTTGATCGATTATTTTAACCTA  TCGATCTTATAATTTTTTCTCCATCATTCATTTAACTAACATCGAGCTTA  TGATTGAAATCGATCTGAATACAATCGTTTCAATCTTTTAAAATAATCGA  TTTTGAGGCAATTTTTTACTGGAAAAAATCGATTTCAAATCTATGGGAAT  ACATCGATTGTGAGCAATCGACTGTGATTAATCTGTCTTTGTTCTCCTCC  CAACTCAGCTATTTGCGCCTCATGATAAATGTTAACAGTTTTTTTTTGTT  TTCAATAG  >Unigene13016_All Similar to past-1 isoform 1 [Tribolium castaneum]  GAAAAATTTTGATTATTACAAGTCAAATTTGGTTTACCTGTTTGTTAACT  GTTCTATTCTTGATTGTGATCAAAATTTCTCTTCTCTTGGTTTAAATTTG  CTGTTGTGACAATTATTCTATTTTATTAACAAGATGTACAGTTTCTTACG  ACGTAACGATAACCGTAAGGAAGCGGATACATTCATCTCTGTGAATGAAG  GATTAAAGAGATTATATAAGACCAAGATTCTTCCTCTTGAAGAGCATTAT  TTGTTTAAAGATTTTCACTCACCACCATTGGAAGATGCTGATTTTGATGC  TAAACCAATGGTACTTTTGGTTGGACAATATTCAACCGGTAAAACAACTT  TTATCAAATATCTTTTGGAACAATCATTTCCTGGTATTCGTATTGGACCT  GAGCCAACAACAGATAAATTTATAATTGTTATGCATGGTGAACAAGAGAC  AGTTACTCCTGGTAATGCTCTAGTCGTTGATCCTAAAAAACATTTCCGTC  CATTATCAAAGTTTGGTGGAGCTTTTCTCAATCGATTACAATGTTCAACG  GTTAACTCACCTGTTCTAGAATCTATAACCATTATTGATACTCCAGGTAT  TTTATCAGGTGAAAAGCAACGTGTTGATCGTGGTTACGATTTTACCGGAG  TCCTTGAATGGTTTGCTGAACGTGTTGATCGGATTATCCTTTTATTTGAT  GCCCATAAGTTGGACATTTCCGATGAATTTAGACGTAGCATTGAAGCACT  ACGAGGACATGATGATAAAATAAGAATTGTTCTCAATAAAGCAGATATGG  TTGACCATCAACAGTTAATGAGGATTTATGGTGCTCTCATGTGGTCATTG  GGTAAAGTTTTCAATACACCTGAGGTGTCCCGTGTTTATATTGGATCATT  TTGGGATAAACGATTACAATATGATGATAATCGTAAACTATTTGAAGCTG  AAGAGCAAGATCTTTTCGCAGATCTACAAAGTCTCCCTCGTAATGCTGCT  CTTCGTAAGCTTAATGATCTCATAAAGCGAGCTCGATTAGCCAAGGTCCA  CGCCTACATAATCAGTGCCTTGAAACGAGAGATGCCTACATTAATGGGAA  AGAATAATAAAAAGAAGGAATTAATCAAGCATTTGTCTAATATTTATGCC  TCCATATCCAAGGAACACTCAATCGCCGAGGGAGATTTTCCACCTCTCAG  TGAGATGCAGGAGAAACTTCTTGACCATGATTTCACCAAATTCAATGCAT  TAAATAAGCGATATATTGATGCAGTTGACAAGATGCTTAAAGAAGATATT  GCTCGTCTTATGTCTCAAATACCACAGGAACAAGCTAATCAAGCTGTAGA  CGAATCAGCTGTTAAAGGAGGTGCCTTTGAACATGCCCAAGAATCACCGT  TTGGTTTTGGCCGAGGCGAGGGTATTGATGAAGGTGCTTTCGACTCTAAA  TGGGTCGTTTCTAAAGAGAAGCCCAAATATGATGAAATATTTTCCGCTCT  TCACCCGCAAGATGGTAAAGTTTCCGGTGCCGTTGCCAGGGAGGAACTCA  TTAAATCAAAACTTCCCAATTCTGTGTTATCAAAAGTTTGGCGTTTAGCT  GATGTTGATCATGATGGTTCTCTTGATTCTGATGAATTCGCCTTAGCTAT  GCATCTTATTAATGTTAAACTAGATGGTCATGATTTACCCGCTGAATTAC  CGGCTCACTTAATACCACCTTCTAAAAGAAAGG  >Unigene14260_All GK16696 [Drosophila willistoni]  AAGGCCACATTCAGAGACTTCATCCGTTTCCGTTCACGAGCATTGGCCGC  TTGTCGACGTTTCTTCATGGTCGTTACTGGAACTTGTTTATGTTGACACT  TTTTCATCATGTAACATGAAAGTTTTTCATTCTCACCTTTAATTGTTGTT  GTCGTTGTTGTTGATGATGGTGAAAGTGATGAGGTTGGTGATAGTGAGGA  TGATGATGAGTGTCCAGTTGTTGTTGTCGGTGGTGATGATGAATTGAATG  AAGTTGAAAATGATGAGAATGTTGAAAAAGTTGATGTAATTGTTGCTGAT  GATGAGGATGAACAATCATTTAGCCAACAAGTTAACTCTTCACCCACTTC  CATTGTTATTTATTTCCAAATTGTTTTTAACTCACAGTTAATAGACTACA  AAAAGTTAGACACAGTTAATTACACTGGACAATTGGAGTGGACGAAATGG  TCAAGCAACAGGTAAGTGAGAGTTTTAAAAAGTTTTAACGCAAAAGTTTC  >Unigene14607_All Similar to Dihydrolipoamide dehydrogenase [Monodelphis domestica]  TTTTTTTTTGTTCTCAATCTGATTAAGACTAGGCTTTTTTAGTTGAAAAT  TTACAAACAAAAATTTAATGATTTAATTGAATTAAATCAACAGTTAATTG  CTTTACCACAATATGCCATAAGATTAGCTTCTCGGAAAGCTTCAGAAACA  GTTGGGTGAGCGTGGCAAACGCGAGCAACATCTTCACATGATGCTCCATA  TTCCATTGCCAGACAAGCCTCGTTGATCAATTCACCGGCGACTGAACCAA  TAATGTGAGCTCCTAATATTCGGTCGGTACTTTTTTCTCCAAGAATTTTA  ACAAAACCATCGATATCGTTATTGGTTTGAGATCTACTATTGGCTGACAT  ATTAAACACTCCAACTTTGTATTCGACTCCAGCCTTTTTCAAGTCTTCTT  CCGATTTTCCGACCCAACCAACTTCCGGGTGAGTGTAGATAACAGAGGGA  ACACAATTATAATCAATGTGAACTGGTCCACCGGCGATACCTTCAACACA  AACTATTCCCTCGTCTTCAGCTTTATGGGCCAACATAGGACCGGGGACAC  AGTCTCCAATTGCATAGATATTGGGAACATTTGTTTGGAATCGTGAGTTA  ACAACGATCCGGTTTCTTTCGTCTTTTTTAACTCCA  >Unigene1518_All Laminin receptor [Litopenaeus vannamei]  AGAAGAACAAATCTACTTTAACAGTTGTTTCCCAAGGAATTTCTCGAGAA  AGTTTTCCTCGCAATCTAAGAACTTCTCTTGCGAGCATCCACCACATTAA  ACCAATCGAAAGAGGAGCTTTGTTATTACATGGAATACCAATGTCTACAA  ATCTAAGCGGAGAATCAGTGTTACAGAAAGCAATTACGGGGATATTGACA  TATGAAGCCTCAGTTAGAGGTTGATGGTCTAATCGTGGATCAGTAACAA  >Unigene1556_All Similar to prohibitin [Tribolium castaneum]  TTAGGTGAATCCATTGCCAAGGATCCTGGTTACCTCAAGCTTAGGAAGAT  TCGAGCTGCCCAACAAATCGCAAAGACGGTCGCTAGTTCACAAAACAAGG  TTTATCTAAACTCTGGTTCTCTTATGCTCAATATTGCTGATAAGGAATAT  GACATTACAGCTGATACAATTAAACCTGGTAAAAAATAATGGAACCTTGT  CAAATGTTAGATGTTAATTGATATAAAAAACGATCACAATCAAAGAAACA  AAAAAAAATAATAGTCTATTCATCTTTA  >Unigene17312_All Rcd1 (required for cell differentiation) homolog 1-like [Saccoglossus kowalevskii]  ATCCCACTTTGTTTACGTATCATGGAAAATGGTAGCGACCTTAGTAAGACTGTGGCCACTTTTATCCTTCAAAAGATTTTGCTAGATGAAACCGGCCTAAACTATATCTGCCAAACTTATGATCGTTTCTCCCACGTCGCCATGATACTTGGAAAAATGGTCATCTCATTGTCCAAAGAATCATCACCAAGACTTTTGAAACATGTTCTCCGATG  >Unigene19624_All Similar to LOC496104 protein [Strongylocentrotus purpuratus]  TTGATTTTCTTGCCTTTTACGATCCAAAGGAGTTGTTAACTTTTCTTACTTCACCTTCTGTAAAAAATGTTTTGTTTTCTCTCTCTCTCTCACTCCACCAATGTATTTACCTTAAAGGACATGAGCTTTTCTCCTGTATGTGGAATCTCTTGAGGCTCTTCACCAGGATCAAAGTCTGTTAATGCAACCGCTGCGAAAACATTTGACCGAGATCGACTTGATATTGATGCTATGGGAGGCGATAAAAGGGAGGCAACCGCTCGTAGGCCAGAGGAAATGTGAGCTGGAAGAGTTGGATCACTTAACATATCAGTTACAAGACCATGAGCTTCCGTTATAAGTGCAACATCCACTGATGAACGAGATGATTGCTGTAAAATGATAAAGATAAAAAGACAAAGAAAAAAGAAGAAGAGAAAATAAATAAGAAAGATTCAAATCTAGTGAATAAAGTGATACAAATTCATCACTAGGGATAAGCCATTGAATAATGATTAGGTTCAACTACAACACACCAATATATTTGCACTCACTCAAAGATGGAAGACGAGAGAGAGAGAGAGAAAAAGGAAAAGAGAGCGTACACCCTGATATTATCTAACCAATATTCTGGATTCTGGATTCAGTTGAATTGACATGGACCTTTACCATTACCCAAAACNNNNNNNNNNNNNNNNNNNNNNNNNNNNNNNNNNACATATTAACTCATCAATTGATCTATGTTAAGTTACAGATTATTCATTGGAGAGAAAGGGGAAAATAAGCTCATCTTTATCATCTTGATGTGTATCCAATATCCAGTTATGTACTTGTGTATATAATTTGAAGAGATGAAATACTTTTCTCGGTGAAAAAAGATGAGGATAAAAGAAAAAGCCAAACCAAGGAAACATCATCATCATTCATCATCTTGAGAAGAAGCAAAAAAAGGAC  >Unigene19776_All Cadherin [Aedes aegypti]  TACCCGGTGGGGACATGTTGGGCTTCACTTTACCCGAAAGTACAAAAGTTGGTTCGGTTGTTTACCAGTTAAAGGCCAATAATCCAGGTCGAGGTCGTTTATCGTATTACATTTCAGGAGACTCGTTTAGTGTGGCCAAAGATTCAGGTGTGGNNNNNNNNNNNNNNNNNNNNNNNNNNNNNNNNNNNNNNNNNNNNNGACGTAATCATAACAATCGTCGACGATTCATCGACTGGAAGTACTTTGTCCATTAAACGGG  >Unigene19934_All Cell differentiation protein rcd1 [Culex quinquefasciatus]  TCGCCATGATACTTGGAAAAATGGTCATCGCACTGGCCAAAGAATCGTCGCCAAGACTTTTGAAACATGTTCTCCGATGCTATTTACGATTAAGTGATAATCCAAGGGCTCGAGAGGCTCTTCGCAATTGTTTACCGATGCCCTTAAGGGATGAGACATTCGCCGAGATACTTGATGCCGATAAGACAAGTCGCCAATGGCTCATCACTTTGCTCAAAAATTTGGAACGAGAAATCGAACAGTAAAAACCAAAAATTCAGTCTACTAAAATTTTCTCATTTCAATTATGCCAATTGGGGTTTTCCACTTATTCCAATTAATCCTCAATCGAACCCTTAATTAGATAAACTCCTTGATTCTGTGATAATTGTTGTTCATCTTTCCACTGATTTCAATGTCCCCTGNNNNNNNNNNNNNNNNNNNNNNNNNNNNNNNTTAACTTTCTCATACTGAATACTATCAACTCAGTTGGTGAGATGAAAATTGAATCAACCTG  >Unigene21618_All GH11064 [Drosophila grimshawi]  CCGCTGGGTTGACATGAGCCCCTGAGATGTGACTAAAACATTGGGTTAAAATGACCATCGCCAGGCCACTTGTAAGCGACATTACAATCCAATTGGGTGTATGACCGATAAGTGACCCAGTCCAGGAAATGTTGACCGCACATACAAGAAACACATAGAAAAAGGTTGATAAACACTCGGCGATTAAACTTCGCCAAAATTCAAGTGATTTAACTTCCTGGGAC  >Unigene23379_All Similar to LOC496104 protein [Strongylocentrotus purpuratus]  AAGTCAATTAACTTACAGAATATTTTATGAAGCGGGTTTATTGGAAGCATTTAAAATACCGATAGTTGAATTTCTAAGTTACTTTAGAGCATTAGAATTAGGTTACAGAGAGAAACCATATCATAATCGTATGCATGCTGCTGATGTTTTACATGGAGTTTATTATTTAACTTCTCAACAAATTCCAGGATTTCCTCAGATTCCATTAGATTCAGATGATTCACCATTACTGAAAAACTCCTCTGGACCACTTCCGAAAAGTTATCTTCGACACATATCAACCTGTGATGATAGTTATGGTATAATGGGTGCCAATTTCCCCGCTCTTGAGATAATGGCACTTTACACCGCATCGGCGATGCATGATTATGATCATCCAGGTCGAACCAATGCTTTTTTAGTTCAAACTTTTTCAGCTCAAGCTATCCTTTATAATGATCGTTCTGTTCTAGAGAATCATCATGCAGCCGCA  >Unigene23503_All Similar to cadherin [Nasonia vitripennis]  CGGGTGAGTTATCGTTCACATCAACAACAGAGACTAAAATGTTTGCCACTGAGGTGAACTTTTCTCCGCCAAGATATTCCTCTGAACCTTTTGCTGGCTGACCAACGGCTTCGACGGAAAATTTGATCTGTTTAACAATTAGTGATTCATAGTCAATACGACGATTGATTGTTATCACTCCAGTTAATTTATTAACGGAAAAATAACCCTCTGGGTCTGTTGAATCGATCTTCTTGAAGTTCGTGATCGGTTTACCAGTTAACGGATTTCTGGCCGATAAGACGAAAACGGTTGATCCAATTAGAGTTTCTTCTGGAAGGTTTATTTCATAGGTCGGCTCTGATGGTGTCCAGGGTGCGATGAAGACCGGATTACGTTCACCATGAGATTGAATATAAATAGTTAGATCGGCGGAAGCTTTTTGATCAAATGAAGATCCATCATCGGCTTGTCCGTTAATATCACGAGCAACTATTGGCACTACAATGATGGAAGCGCGTTCAGAATCAACTGTACCATTAACACTAATGACTCCTGACGCGGGATCAATTTTGAGAAGATTATTGAAATATTCTGATCGTCCAACCAATGCTCCAGTTTTAT  >Unigene26329_All Target of rapamycin [Blattella germanica]  GTGCTTTTAATTTACAATCGAATGTTGTTTGATACTCTAATTCAATTATTGCTTCGAAATTTACACTTGTTAATCGGTTACAATTCACTAATATTTGGATAAAAGGATAAATTTTCATTCTATTNNNCATGGGATCAAAGGAGACCCTTTTGGAGGGTTTTATCAAAGGCCTGAAAAGTAAAAATGAGGATACAAAGTACAAAACAGCGCGGGAACTACATCACTATGTTTCCACTGAACTACAAGAAATGTCAGTGGAAGAGATCCATGAATTTATGGAAAAATTTAACATCAAGGTTCAAATGATGATGTCTAATGGAGATATAAATGAAAAGAAAGGTGGTATTTTGGCGATAATTGTTCTCATAGGTGTTGATGTGGGTAATCAAAGTACAAGGGTATCTCGATTTGCCAACTATGTTAGAACCCTGGCTCCAACTGATGTTGGCTTAATGGAACTAGTCGCCTATGCTGTTGCTCGTATTGCCGTTGCCAGTGGAACACCAACATTAACCTATGTTGATTATGAAGTTCGTCGAGCCATCGAATGGCTTTCGGGTGAAAGGAATGAGGCTAAACGGCATGGAGCGGTTCTTATCCTTCGAGAATTAGCTACTTCAACTCCTGCTTGTTTCTTTCAACAAGTTCAACAATTTTTTGAATGTATTTTCACCGCTATTCGTGATAAAGAGAAGAAAATACGTGAAGGGGCAGTTGGCGCCCTACGGGCTGCCCTAGCGGTCACCGCTTCCCGTGAGACTAAAGAAACAGCTAATCCAACTTGGTATGCACACTGTTATAAAGAAGCATTGAAAGGATTTGATGAAGGTTTTCATATACAAATGACTCGAGAAAAAGTGACCCG  >Unigene28986_All GH18585 [Drosophila grimshawi]  GTGGAACTAAGTGCTGAGGAAGCTTTTCTGGTATTTCATGGCCGGAGATTTTAATGTTGATGAGATGCATCGCCAGCGCGAACTCATCGATATCGAGGCTTCCATCATTGTCAACGTCGGCGAGTTTCCATATTTTAGATAGAACTGAATTTGGTAGCTTTGATTTAACAAGTTGATTCTTTGCTTGTGCACCAGAAACTTTACCACCAAC  >Unigene29148_All CDC-like kinase 4 [Salmo salar]  AAGAAGAATCGGTCCCGATCGTCGCATAGTTCAAATGTTCGATACTTTCAAATACCGTAGCCATGTTTGCATTGTCACCGAGATCTTAGGTCCAAGTCTATATGATGTTCTGGGATAATGAATATCATCCGTTTCCACTGATCCAGATCAGTAAAATAGCATACCAAGTAATTTCCACAATCAGATTTCTGCATAGGAATAAACTAACTCACACC  >Unigene3022_All GD14493 [Drosophila simulans]  GTTTCGCGTGTTCAACTTTCTTTTTTATTTGTCCATTTTAATAACCAAAA  GAGAAATTGAATCAATGACAATTTAATCTTTAAGATGATCATTTCTAAAC  TAAGTCAAGTGTTGAGTAAATCTTTTAACCGGTTATCACTTTTTAAACTT  CATGAAGCAATTGAAGCGAAACATGACAAATATGGATCAATTCGTGTGGA  AGAAAAATATTGGCCTGTTAGACGGACAATCGTTTACGTTTTTAATCCAT  CCGATATTGAGAAAATTTTACGAGCCCAAGGTAAATTTCCCTATAGACCT  GCGAATGAATTTCTGGTTAAATATCGTTCATCTTTACCTGATCGTTATCC  GACAATTGGAATTGGTAATCTACAGGGTGACAAGTGGTTCTCTCAACGCC  AGTTAATTGCTCCTATTTTATTATCCAGTGGTGTTCTTAATTCCTA  >Unigene31940_All 14-3-3 epsilon protein [Bombyx mori]  AAGGAATTAAAAGATATCTGCTCAGATATTCTCAACGTTCTAAATAATCATCTCATACCTCATCTCAAGAGTGAAGAATCGAAAGTCTTTTACTACAAAATGGTTGGAGACTATTATCGTTACTCTGCAGAGTTTGCTACTGGCGCTGAGCGCAAAGAATCGGCAGACAAAAGTCTCGAGGCGTATAAAGTAGCAACAGAGACTGCCACATCTGAATTACCGCCGACTCATCCGATTCGTCTTGGCTTGGCGCTTAATTTCTCAGTATTTTATTATGAAATTCAGAGTTCGCCTTCTTGTGCCTGCCGATTGGCGAAGGCTGCTTTCGATGATGCAATAGCCGAGTTAGATACGCTCAGCGAGGAAAGTTATAAAGATTCAACCCTTATCATGCAGCTTCTCCGGGATAATCTCACACTCTGGACTTCAGATATGCAAGCAGATGGGGACGAAACCGTAGGAGGTAAAGATCAGCCAGCCGATTCGAATGAAGAAGTTGAAGCGTCATAGTCGGCCAATTTTAATTGTTTTTCAAGATAATGCACCGGTTCATAAAAGCCAGAG  >Unigene3365_All Similar to AGAP008814-PA [Tribolium castaneum]  GTTGTTATTGGCATCATCTCATTTGAATTTGTATTTTGTAATTTGTTTGT  TTTTTTTTAAGAAAATAAATTGTCATTATTATTAAATTGATGATTTGATC  CGCTGGGAGGCATGGGTACTGGCCCAGGTCCGATCAAATGTCCTTGGTGT  CCAAACTTGTTGTCTTGATCTTTCTCTTCTTCCCTTCTTTTTAAACATGC  TGTCTTTGGATTTAAGTTACGTTCTCTAACTTGTTGCTCTAAACTTGTAA  TTACTTCAACAGCTTGATGTAAAATATTGAGTTTGGTTTGTGCTTTTTCT  TGTTTTAAATGCTGCATGCACATTTTACCAAGTTCCTTAAATGCTTCATT  AATATCGCGAACACGAATTCTTTCTCGTGCGTTGTTGGCTTGTCGTCTTT  CTTTTTCCCTCTCTTGTTTAATCTCCGGAGGATCATCTTCATCAGCTGAA  CTACTGATGGAATGTCTTGATCTTGATCGTTTACCTCCTTTAATGACATT  TGTTGGTGGATTACTTGTAGTAGTTGTTGGAACTATTCGTGCTACAGGGG  CAATGGCAATTGTTGGTTGGATAGTTGGCAATTCTTGTTTTAGGGACACG  GGAGCAGGTTGTGGTTGAACAATTGTACCACTTATGGGACCACTTTGATC  TGAGAAACCTAAATTTGGCATGTGGCAATCAACCTGAGAGTAATTAATGC  TTTCTGCATGTGTCTTTAGGACATTTATGGCATCATCGAGTCTTTCCTCC  ATAAAACCATTGAGAGATGTTGACCATTGAGGACCAGGTCCTTCTGATAC  TGAGACTGGAGCTGTTGATGAGGTTGATGGAACTGTTGCTTGAG  >Unigene3660_All Similar to membrane-associated protein gex-3 [Nasonia vitripennis]  CGGTGTTGAAAAAACATGTAAGTTGGAAGATGGTGAATGTAAAGTCTATT  AATTTTCTTAAGGAATAATAATCATCGGGTAATTTTCAGATAATTGATTA  AAAAGAGGAAAAAAATAATAGAAAAAAATATCAAAGAAATATAAATGAAT  GATAATGATGATATTTTCCGGTGAGCATTTCATGCTTTTTGTATGTGTGT  GTGTGTGTGTGTGTGTGTGTGTGTGGAGATTAACAGAGAGCGAGAGAGAT  AAATTTTTAGGTTGATGGATTGGTTATCCCTTGATTCTTGTAAACTGCAT  GATAAGCATTTCGTAGTAGCGCATAAGGGAAACAAGACTCGAGTAAATCC  ATGGTTAGAAAAGGTGATTCTTTGACAATTAAATCTAATAAGATGTAAAT  AGATTCTCGATTTCGAGTTGCTTCTTTGTCTGGTTCAAGACCCAAACGAA  GGAGACTCGATGAGGCCAATGCTAAGAACTCCTTTAATCGGTCAACAGTA  TCTCCGTGGTTACAAATTGTAAAAAGTGCTCCTGCAATTCCGTTAATTGC  TTGAGCTAAACAATGAATATTATTACCATGAGCTTCCAATGATGGTTTAT  AATATGATTGTTCACCGCGAGCAAGTTTAGGTAAAGAAACAGCAATAAAA  ACCATAAGGAGACAAGCAATTTGATATTCATCTTCACCCAATTCACTTTT  TTGAGACAATAAAGCAGTAACTAAAGCTGGATCAACCTTGTTTGGTAACC  CAGCCGCCGAACACATTTCACTGATACTCTGGAGCTTAGCACTTTCAATC  ATTGATTCACCATTTGGAACATGATGCTTAAAGTCAAGAATTGAACTCAT  CAGAAAAGGTATTCGATCAAATAAAACATCGCTCAAAGCTTCTTGTGCCA  ATTCCCGGAAGCAAAGGATAACACCAATGATTGTCATTCGTTGTAAGACA  CTATCTACATTTTGTAGTTTTTTAAATAACTCTCTCATTTGTTCAGGTTT  ATCATAATTAGATCTTAAACCAAGAAGTGTTTCTTTATTCATAATAACCA  ATTTCTTTAATTCAGTCACTTGACTTGCAATATGCCACATCAGATTTTCG  TTACAATATTTCATTCCATAGGGTCCAATGAGTTCAGCTAATGCTCGTAG  CTCATTAACATCGGAATATTCTTCAGCACAAAAAGGTACTTGACCTTCAA  CCGGTAAGGTATCAAAGGCTTTTTGTAATGGAGAGTAACATATTTGACCA  GCACTGACTCGCCTTAAGAGTACCTCAAGGTACCAATTTGAATAGAGAGC  GGTAATTGTTTTGTCACCATGACTGTCCTGTGGTTGTGTTTGTAAAAGCA  GAACAGAGTTGAAAATATTGGTTATCTCAATTTGAACATAATTTTCAATG  GATTGGAGGACATTCATGTAAGCTCGAACACTGCTGAGTAGTTCAGATGG  TTTGGCGATTTCATTTGTTTCGGGATTGAACATGACCATTCCAACAAGAG  CTTTGTTAAATCGAGACTCTAAATGACCATGAAGATATTCACGAGGAGCA  AAAGTGTGATCCCAAACGTGTATAGATGGACAATAATTGATTGCAAAACA  AAGTTCAGTTAAAGCCATATGAAGTTTATCCATTGTAGTCAACTCTTCTC  TGGTTCGACGATAACTTTCAATTCCCGGTTTGTCGATTTCTCTTTCAATT  TTATTCTTCTTGTCTCGTTTCTTTAATTTCACCACTGAGACTATTTGAGG  TGCTACATGTTTAGGTAACAGTTGATCATTGAGCATACATTGATGATCGC  AAATTGTGGTGATTATATTTTTGGCCTCTTTTGACATTTCATCGAGGAAT  AGATTAACCATGGAAATGCTTCTTTCTCCAATTCGAGTTCGCTCTTCAGG  GGCAAGGTCATGGTATGAATTAAGGAAATGTGAGCAAATCATCGGGAAGG  CAATGATGTATCTCGTTTGCGCTGGAAACTCCAAACACATATGAAACTGG  TCATCGAAAATTTTACTATAAAAACAGAAAAGGGAAAGATCTGAAGTTTC  CGTTAATATATCTTCCAAATAATCAACCATCTTAGTATGGAAAACAACAA  TGTTCATCAAAGTGGCTAAGTTTTTGTGCTCAAAAAGGTTCATTGGGTAT  CGATTGACACTACTGTAGGCTTGTAAACGAAACCAATCCAATCGAAGTCC  TCGAAAATCAAATATTTCATTGTTTTCGATTTGTTTAACTGAAAGGTTTG  ATATTTGACTGTAAATAGATTCCAAAATTACTGAATCCTCTTCCGGCATC  ATAGAAACACCTTGCATAATATTATTCAAAGCTATTGCATCGTAACAAGT  TAAATATTGGACATAATAACGTTGAACTACTTGATTATATTTTCTCACTA  ATGATCTGAGTTCCTCAATGTGAAATAAAAGCTCTGGTAATTGACGATCA  ACAAGATCTTCCGCTTGAGCTTTAGGTCGACCTTGTCGAACGGGAATATT  GTCATAATGTCGCAATAGCCAATGAACCTCATCACGGGCAAAAGACAAAG  CCATGAAAACAAGAAGTGCTTTTGGTCCTAAAAGTCCAGGTTGATCTCCC  AAGATTAATCCCAATTCTTTCAACGCTGAGCGTAAAAATTTTCGCTTCTC  TCGATGAATATGTCCAGCATTTTGAAGGACATTGTTATAAGCATCTTTAA  CATCAGAGACTCTTTTGTTGTAACCTTTGATTCCTTCAAAAAAATGTTGG  ACAAATGCATGGGTATGAATAACTTCATCTCGGTGAAGAGTTGTTACCCA  ACCACATTGAAGAGCTTTTGAAAATAATTCATGAGCTGAAGTCTGATTAA  GATGTTGATGAATTAATAGGAATCCAAAAATTATATATCTTTCCATGGAA  TCAATTGAAAGGTATTCGGTGTTTATTGGATCTGTTTGTGCAGGAGTCAG  AATGTCTCTTGGATTGGCAACGATGCTTAGAATTTGACTTTTACGCCATT  CCTCAGCTGGAAGATTACGAAGATGGAAACTTTCTTGGAGAGATATGAGA  GCGTTAAAAAGTAACTTTGAGTGTGGAACAAAATCCTCTGACAATTTTTT  AAGTGGATTCTCATATTCGACAATCATTTGACCTAATCGGGGAAAACTGT  TCTCACTGTGTCCATGGGTAAGTTCGTGGGCAGTATTAAATAAACCAATA  ACCGCCTTTCTATCCTCAATTCGTGAGACAAGAATCATGAGTGATACATA  AGTTACCATGAGATCCAGATAGCCTCGAGTTAGATCAAAATTGATGGAAA  TATCGAGTGTCAATTGACAAGCATCCATGGTGGTTAAAAGTTCATTAACA  TGGTCTTTAAACTCCAAAATATCCACAAAGGTGTAATAGAAATGAGTTGT  AGCCTTAGTTATCTCAGTTCGAGCAGTAGCAACCGCACCAATTAAGCTAT  TATTACTCCTTGTATCTGTGTGTGGAAATCTTCTGACAATCTGTTTCATA  GCTGATTCCAAACCTTTGTCAGAAAGAATAGGAGGTCGAAGTTTAGGATC  ACTTGATTGCTTTTTAATATTATAAAGTCTAGTTAACATTCCTATTCCCC  GGTCATTGAGAATAGTCAATTTCTCAGCTAATTTTTGCTGAGATACATTT  AAATGACGAACCATTGTTCTTTCTTAATTAAGACTATACAAGTCTAAATT  ATTTCTACCAATGGACACAACAATTAAATCACTAGAAAATCA  >Unigene3988_All GK23020 [Drosophila willistoni]  ATTCTATTCTTTTTTGTTTATATTTTCTATTTTATTCTATTCTACTTTCA  ATTCTATTGGATTATTATATTGAAAATCATGAAGGTAGAAGTTAATTTTA  AGATGAAACACATTCTTAAAAGAAATGTTGATTCTCTCCATCATCATCAT  CATCATCATCGTAATTCCCGGCGACGAAGACGAAGACAACAACAACAAGA  ACCCGCTAATTTACAACGAAATTTTGATCAATTACTTACCGTTAAAAGAG  CTCTGTTTGTTGATAGTAATTTAATTGATCATCACCGAGAAACTAGTGAA  TTTTTAAAAGCCGAACTGGACAAAATTGAAAATGAACATTGTGCAAGATA  CAATTTTGATTTTAAAAATGAACTACCTTTAGATGGTAATTATCAATGGA  TTAAATTATCATCCAAATCATCATCATCGTCAACAATTTCATCTCCATCT  TCATCTTCATCTTCACCAACAACAACAACAACAATTGTAGAAACAAAAGT  TACTTCTACTTCATCTACATCATCACTACCAATCAAAGTTTCAAATTGGA  CCCAAAATCTACCAGATGAGAGAGATGATGGAGAAAGAAAGGTGAGAAAG  AGAGAGAGAGAGTGTGTAATGAAAATGAAAAAAGAAAGAGAAAAAGAAAG  TAAAATATACTAG  >Unigene4054_All Sex determining protein [Mus musculus musculus]  TTTGTTGTTGTTTATGCTTGTTGGGTTCCTCTTCTTTTCATTGTATCAGA  TCGATAACCACGAATGGAACCTGAATAACCAGGAACTCTTTTATCACGAA  AATTGATTGAGACAACAACACATACACAAAAGTGTTATTGAGAATAAGAT  TCCACCAATTAGTTTAATTATTAATTTTAATGAAATTGGTGGTTCATTGG  CTGATGGTGATGAGGAAGAGAAATTCCAATTTGTTGATCGTCTAGAAATA  CGAACTGTCCATGATTTTGAGTCTTTTTCTGTTGATGGTAATGAGAGAGA  TGAAGATTTAGATAATGGAGTTGTTGATGATTCTGAGCTTTCATTTGGTG  TAATTGTTGTTGTTGATTCTGTTGTTTTGGTTTCACTTGTCGTTGTGGAT  GAAGTTGTTGTTGTTGTTGTTGTAGTTGATGATGAAGATGCTGATCGACT  GTCAACCCATTTGAATAAGGTAATGAGCAGTAATGTGAATATAGTAATTG  ATTTAAGTGAGAACAGCATTTTACTTGAAACAGAAAATTCTGATCTAATA  ATTGAAAGATGTTGATTCTTGTGGTGATGGTGATGATTATGGTGAAAACT  ATTATGATGATGATGATGACGAGGCTTCTGTTGGGGATGCTGATCTTTTT  ACTGATGGTGATAGTGATAGTGATGGTGATGCTATTGATCGACACCATGG  TAAGCAACTAAAACAGGCAAGTTGAAGCTTTATTCTGATATTCTGGATTC  TGGATGATGAGGAAGATGAGGATGGAGAGAAAGAAAGAAATTGAGATTCC  TGGAGGCTGCGTCACCTTCTCATTTCTGTCTTTTTTACGAATTTTCTGCT  CTCCTTTTTCTCTTTCTCTT  >Unigene8828_All Sex determining protein [Mus musculus domesticus]  AACTCATCATCTTCCAAACTCAAACTTTATCAACACACAATACCAATCAC  CAGAACCACAACAATGCCAACAGCAAATGACTAATGCCCATCACTCTCAT  CATCTTCAACCACAACAACTACCAATTCAACAACTAGAGCAATCTAATTC  GCAACCTCTCCATTATCAGCAATTTCATAACTTCCATAATCAATATTCTC  AATCTCATCAACATCCTAGTCCAGATTCTCAGTATAATCGATCACCACAA  CCACAACCACAGCCACAAACTCACCAGTTTCAAGC  >Unigene9093_All Similar to Darkener of apricot CG33553-PG [Tribolium castaneum]  ATTTGAAAACTTTGATATTCTCTCTTTCTCTTGTTTGGCTTATTTAATTG  CCTTTCAACTCCATCTTGATTTTCTTTTCCAATCCTAATTATCATCAATA  TTCTTTCTCCTGTGTCCATTCATCTGGTTCAATTTGTTCTTTTTTCTCAT  TTTCATCTTTGTTCTTTTTTCATCCCCTTCTTTTCTTTACCCTCTCTTTT  TCTATGTTGATATTTTCCTTCCTTTATTCTCTTACCCCAACCTCATCTTT  ATTCTATCATTATCTCCACCTTTTATCACAATTACTGTCACTTTCTTGAT  CCGTCTGAACTATTGTGCTGATTCTCTCTGTTCTCGGCTGGTGGTGGTTG  GTGTTTGGCTTTCACTGTAATTAAAATTTCCCTCGGTTCTTCTTGCCTTT  TGGTTTTGGCTTTTGGAACATCATGTCAAATTATCGATCGCGTTCTCCTT  ACATAGCAACCAGAAAGGGTAGAAACTTATCACCAGAGAGAAGAAGGTAT  CGTCGAAGAAATATGGATGACGATGAAGATGGACATCTTATATATCGCCG  AGGAGACATTCTTCAAAAAAGATATAAGATACTTTCGACCCTTGGTGAAG  GAACCTTTGGAAAAGTCGTTCGAGTCTTGGACCTTCGGAGTGATCAAATG  GTGGCTCTTAAAATAATAAAAAATGTCGACAAATACCGAGAAGCTGCAAG  ACTGGAAATTAATGTTCTCGAAAAATTAGCTGAAGCAGATCCACATTGTA  AACAGCTTTGTGTAAAAATGTTAGATTGGTTTG  >Unigene11279_All Ubiquitin specific protease 9, X-linked-like isoform 2 [Oryctolagus cuniculus]  CAATTCAACATGCACAATCACAGTCTCAAACTCAACAATCTCCACCATCA  GACTCTACAAAAAACGATGTGATCACTGTAACTCCCTCATCATCATTAAC  ATCCATACCAAGCTCCTCAACTTTTGATCAAAGTAATCTCAATATTGAAG  AAGTTAATCCAATATGCACCACTTCAGCCACCCTTAATGCGGCTTACGAT  TGCCTTGTAGCTTTGGGTACTGATTGTATACCAAATTTAAAATATTTAGT  TGATAATCTCAATGAGATGTTCTATTCAGACAATGAACAACATCTTACTG  AATGGGAATATCAACCTCCTATTGGGCCTCGACCATCTCGTGGTTTTGTT  GGTTTAAAAAATGCAGGAGCAACTTGTTATATGAACTCAGTCTTTCAACA  GCTTTACATGATACCCGAGGTTAGAGATGGTATTTTATCAGTCGAAGGAG  CTGCAACTGATTTAAATGAAGATTTCTCAGAGGAAATGGTGGATAATATT  ATCAGCACCTTATATCCCAGTGTCCCTGAAGATGATTCAAAACGGTCCGT  CTATGAATCTCGACGTGATTATAATCTAGGAGTTTTCAAATATACCCAAG  CAATTTTTGGACATTTGGCTCTGTCCAAGCTTCAGTATTATGTACCGAGA  GGCTTTTGGAGGCATTTCAAACTTTGGGGTGAACCAGTCAATTTACGTGA  GCAACATGATGCCCTTGAATTTTTCAATAGTTTAGTCGATTCTATTGATG  AAGCTCTCAAATTGTTGGGCTGTTCTCCAATCATGACCAAAATTTTCGGT  GGATCTTTCGCCGATCAAAAGATTTGCAAGGAATGTCCGCATAGATACTC  TCGTGAAGAACCTTTTACAACTTTAAACATCGACATAAGAAATCATAGTA  ATCTATTAGATTCATTGGAACAATATGTTAAAGGTGATTTATTGGAAGGT  GCTAATGCTTATCATTGTGAAAAATGTAATCGTAAAGTTGAAACTGTTAA  ACGATTGTGTATCAAAAAGCTACCACCAGTTTTAGCAATTCAATTGAAAC  GTTTTGATTATGATTTTGAACGTTCCTGTGCCATCAAATTTAACGATTAT  TTTGAATTCCCTCGAGTTTTGGACATGGATGCTTATACTGTTAACGGTTT  AGCTAAAATTGAAGGTGAAATTATCGAAGAGGATTTGGAATACACAAGTT  CACCTGGATCTTCATCTTGTACCCGTTATGAGCTGTGTGGTATTGTTGTT  CACAGTGGTCAAGCCAGTGGTGGTCACTATTACTCTTATATACTTCATAA  AAATATTGATGGTTCACGTAAATGGTACAGATTTGATGACGGTGATGTAT  CAGAATGTAAATTGGATGATGATGAAGAAATGAAGGTCCAATGTTTTGGA  GGTTACTACATGGGCGAAGGATTTGATCATATGTGGAAAAGGATGTCATA  TAGACGACAAAAGCGTTGGTGGAATGCATATATTTTATTTTATCGTCGTA  TGGATACTCAAGAAAGTGAGATGGCTCTTCGTTTAAGTGAATTAACCTTT  ACCGATAACGGATCTTATCAGAATAAAATCAAAATGCCAGCAGCAATTGA  AAAAAGTGTCCAAAGACAAAATATTAAGTTTATGCACATGAAAAATCAAT  ATAGTCCAGAATATTTTCAATTTATGAAAAAATTGTTGAATGGTAATGGT  CAGATTGTCGTTTCGCAATTTCAACGGAATGAACCTTATCCAGGTGACTC  TTACTCATCAGATGTACAAAGCAACATTAAGAAAAGTGAAGCTGAAGAAA  TTGCAATGATCTGTTCCCAACTCGCCTCGAGATTTTTATGATGGAACAAG  AGAGAAAACGGAAAAGAACTGGTCAAAAGCCCTTCAAGACAAACAAAACA  AACATACACACATACACACATACACCTTGCATAATGATTCAAAGATGATA  TTGAAAATTCTGTGTATTTTACAGATAAATCAAACTTCAGGATATCTCTC  CCTGTTAAAATTTCTTTATCCTTCCAATATCAATTAACAACCCTGAAAAT  GTAACCTTGAAGTTCTGATACTGTTGATGTTTCTCCTCTTTCAATACAAA  AGGTATATAAAAAAAAAGATTTACACACTTGATGCCGGCTCTGTGCACTT  CTAGCCAGAATTATCATGATAATGATGATTATGGATGAGGAAAAATCATG  ATGAATGATGGAAAAAAAAGAGAATCAAAATTTTCTCTTT  >Unigene13902_All RNA-dependent RNA polymerase Family family member (rrf-1) [Caenorhabditis elegans]  TCTTGGATTTCACATGTGTCACTATGTAAAAAGCAGAGGCACAAGCCATT  TTTTGGTCCATTGATTTACCGTTAATGTTGAAGCGTCTGTCGGCTTTTTT  TACCAAAGTTTCATAGACAATTGAAACAACTGTATCAATCCTATCGGAGC  CTCGATTTCGTCCACTATAGTCTTGTGAATCTATTAGTGTAAGCAATTCA  TTTTCAGAAGCGTATCCAAGTCTTTGTAACAAAACTTTCAATGTTTCCTC  ATATTCAGTGTGTGCTTCTAACGCAGCCTTTGCAAAGGATTTCCAATCAG  AATGAATTAGTCGTTTATCCAATTCGAAGTTGGTTTCAGTTTCGCAAGAA  AATAACTGCGTCAATTCGGTGTAAGAAACGATATCTTCAAGAGCCTTGTC  TGAAAGATAAGTCAATGTTATTCCATCCTTTTCCATGAAACTGGGAATTT  CTTGACTCCTATCACGTGCGTTAAAACTGAATTCACTTGAACAACCATGT  TTACCAGAATCCAGCTCGAAAGCTAATTTGTTCGCCAATTCTTTACATTT  CTCGGAATTGATACCTTCCTTATCTGCTCGAGATAAAAGTGCGTTAGAAA  TTTGACCAACAGCACACTTTCGCTGATAATCGGTAAAAAAGTCAATACAC  ATTTTATCTGTAAAATTCTGTTCGCACATAACTGAGTTCTCAAAACTCGG  GAAAATCGCTGGTTCATTAATATCAATTTTTAAGAAATCTTCTAACCATA  TAACACAATATTCATCTCCATCAAGATCTGCTGATCCAGCCATTTGGTCG  GGAATTGGTCTTGTTCCAGCAACTGGGAAAACGATGCAATCTTTGATATG  GTGGAGCTCAGGTCTATTCACGGCAGTTAATCTTCGGATATCTCCAGAGT  TTGCGACGGGAAACTTGGTTACAATGATATCACCTTCCAGGGGACTACTA  AAGTCTGACGGCCAAGCGAAAACCTCACCATCTTTAAGTACTCCAGTTCC  TAAGCAAGAATCAGAACTTTGTAATTTGGGGATAACAGCATAACCATCGG  AATCAAATTCCAGCTGAGATTTAGGATATTTTACAGTTGGATCCGTGACC  CCAAACATTGTTCGGGCTTTATCCATAGGCAAGGCTATTCTAGCTTTAGA  TTTAATGATTTCTAAAGCTGAATGGTGAACAAAAGATAATATGTTTCCAA  ACAAAGGGTCACCGTTTAACTTGTAACCAACTCGAGCCAACGAATCAATG  TCAATAAAACGATTGATAGATGAATACGATTTTAAAGCTTTAACAGCAGC  TGAATTATCATTGAAGCATTTCTGTAGTTGAGAAACAGTTTCAAGTATCA  GATCAATAAAATATTGATTCGATAAT  >Unigene14104_All Apoptosis regulator BAX [Lepeophtheirus salmonis]  CGGTGGGTGAAGATGAGGAATCAAAAGGCTATCCAGATTTTTGTTAACAG  GTCAAAGGTTACGTTTAATGTACATTGCAAATGCAACTGATCCCAAAATA  GCACAAACAGTAACTGCAGTTCCTTGGACAAAATCTGAGATCACTTTGTT  CCAACCTCCTTGACTTCGGACCCAGGAGCAGATATGATTAAAGATGAATT  TGATTGTCCATCCAATGAGTTGATGAAAGTGGACAATTGGACTGGACGAG  TATGCACGAAGGGCAATGTCCGAGCAGAAGTAGAAAAGTACAACAATACC  TTCACGGGTTATGCGATTATCGGGAAATAGTTCACGAAGCATAGCACTGA  ATCTATCGTAAGAATCGATTCTTATGTTGACACTTTGCGCTCGTCGACGG  ATAATATCCCGTTCTCTTGATGCCTCAAATTGAGCAGCGATTCGTCTTAA  GTCTTGACCAACAGTGGCCATCATTTGTTCAGCATAGGCCTCGTATTGAT  TGAGGATTTCAAGTGATGAATCCTGAGGAGGTTGAATACCTTCATTATTA  AAGGTACTACGAAAGGCATCAGTTAAAAGCACCTGAGCTGTGGCGGTTGT  TTCATCATAAGGTACGACGATTTCCTCAGGGGAAGATCCACCACGAGTGA  TGTATAATTGCTGTTGACCTGAGTCACATTTTTCGATTCGAATTCCAATG  GCCATGATGATGGAAGAATCATGAAAAAAAGTTCACAAAACTTGGAAACA  AATAATTCACAGAGGCACAGAAAAAAAAAGAGATGATGAAGAGACAAAAA  GAAAAACGAAAACAGAAAATGCAAAATGCAAATGGCGGAAAAGAAAAAAA  CGAACCAAGG  >Unigene17510_All Bax-like protein [Acropora millepora]  TCCGTGAGTTTAAATGATGATATAACTTTCAGATAATAAAATATGAGAGAGGGAGAAAGAGAGACTTGATTATCTGATTTGGTTCATCATTTAAAAGTCTCAATCATTAAAGACCTTTGATACTATTGGTGTAAAATCTGCATGGAAATTGATATAAATGCGAGAATACCACCAACAAGAGCTGCCGTTTTCCAAAATCTAAGGTCAGAGAAATTTGTTGTATTGGGTTGATTTGAGTATTCGATTAGACCATTAAAACCGCCATGGTTATTCATCCAATCCAATAGTCTAGCGGAAACATAATTTATCAGCCAACCGGCAATCGATTCAACGATAACTTGATCATTCTTAATGTGATAAAATTCTAGGGCAAGTTCAAAGGTGAACACAAAGAGCGTTAAAATGCTACTCCATTTGATACCCTCGGTGAAAAGATGATCAGCAATTCCAATGAAAATAATGTAACCTCCTTCATCGAGCCGGTCAGGTTCAAGAGTTGAATCTAAATTATTGACAACGGTTTCCACCTTATCTCGCAGCTCATTGTAAGACTCTATGGAAGATCTAAAATCATTGGACAATTTGTTAAGAATATAAATGAATGAAGAGATTGATTGTTCCTCAGTTTGACTTAATCTGGTGGCAGCATTTGAATTCCTTTCAGTTGCTTCTCTTTCTTGGTCAACCGGTACACCGGCGGGTCTCCATTGGATATCTTTGGCTCTAAATTGCTCTCTAAAATAAGAATGCACAATAAATTCAATATCTTTCGCTAAAATACCGGGCATAACTGAACAATTCTAATTTGTAAATACACACACACAAAGAAGAAGAAGAGAAAGAAAGAAAAAGAAAT  >Unigene19747_All MutS protein homolog 5-like [Pongo abelii]  CGATAGCAAAACAGGAAATCAACCTGGAAAGATAAGAATCATAACTGGTCCAAATGCAAGCGGTAAAAGTATTCATCTGAAACAAATGGCTCTAATCATTTACATGGCTCACATTGGGAGCTTCGTGCCAGCAAAATATGCAAATATTTGNNNNNNNNNNNNNNNNNNNNNNNNNNNNNNNNNAAAAACAATTGAGTCCATTTCAACGGGCTTATCATCTTACGTCTTGGATCTCAAACAAGCGGCACTTATGACCCGAGAAAAAACTGAACGATCTGTTTTCGTGATTGATGAGCTAGGAAAAGGAACTGATCCAGTCAGTGGAATTAGCCTACTTTCAGCTCTCATTAATTATTTTTCTCAAGCTGATCATGTTCCTCATGTATTTCTGGCTACCCATTTTCTCACCATCA  >Unigene20792_All C3f [Aedes aegypti]  GAAATCGACGATCGGCTTGATGAACGGCGTTCAGGAAGAACCATTTTATGGACCCAGGGTGAAATAAGTATCCATGATATGTAAAATAAGTGACCTGAAAAATAGACGTGCTTCAGAACGGGAACCCATCGCTTGTATGTTAACAAAGTAAATGAAGCGAGACAATATCCGAACATGTAAACGGTGTAAATATGAAGAAAGATTAATATTGGTAAACGAATGTAACTTTGATTTAGCAAATTATCCAGAGACTTATTTGATTGTCGTAAATTCTTAATTATTGCCATCACCTCCCACTCCATTTTCATGATCAGAAACTCCATTCCAAAGGTAACATAATAACCAGATTTCCATCCATGCCAAACAGCCAAGAACATGAGAGTTGCAGCATGACTGATAATCCGATTCCCTAAGAATTTCAATCTCTTGAAAACATATTTTCCTGACCATTGGTTAGTAGTTATATTAAAGGCTTTGATTAGTCCAGTAAAATTTGGAGAGGTTTCAAAAAGATACGGATCTGCATTACAATAGCTTCTGAATGTTGAATTTGACTTGACGAAAGTGAATCCAGACATTATTAGACTTCCCTCAGCGAGAAGCCAAATTCCAATATATTTGCATGTTTGTATCTTCGTAGTTAAAGTAATTAAAGTGAAGCTCATGAAAAATCCTGAGGTTTTATAATAATCTGATAGCAAAAGTGATGGAGGGAAATATTTCTCTCCAGCAAGAAAGCAAATTAAGTATAAAAGTCCAAGTCCAAGTTTAATACTCGCTTGTGATGCACATGCAGGTAGAACATTATCTTTCTCTTTGAGAAATTGCTGGAATTTTTTATACTCAAATTGTGGTCCAACCATAAACGAAAAGGGAAGATAACAACATGCGAAAAGTTCAAGAATTTCAGGAGCCTCAGTTATCGGTGTTTCATTTTTAATTGGTTCGCCATCGACTATAACTTTAGCCGCGATTCGTTTACCATCGTAAAGGTCAAAAGCGATCGCAATTAGCCGGAGGGTTAAAACACACTGTGGAATAGTCCATTCGAAACTATATTCATTTGATTTGATTTGTGTAATAAAATAACCAGCTAATAAATAACCCAAATTAAAAAGAAACGCAAATGTTACAGAAAATTTAGTCCCACCAAACCATTTAAGAACAAAATACATGGTGAAGATTGTGATAGTTGTATGGAAAAAATCATAGCCATAATTGAAAAGTCCCAAGGCCAGACCAGTTCCTGAGAAGAACAATTTTTTTATCACTTCACTTCGATTGACGATTAAACAATTATAAATAATGGCCAGTGGATAACCAGATAAAAGTGAAAAGAGGATCTTAACAGCAACTACATTGGCACCAAAAGAAGTTGCAATATATACAATGGGCGAGAAAGATGAAGATTGTTGATGATGATCAATATCCATTGTGAGAGAATTAATTGTTGACTCTAATTGTGATGTATATTTTGTGTTTTAAATGGAAGCAAAGGTGAGATAAGATAAAATCACCG  >Unigene23753_All Similar to PIWI [Nasonia vitripennis]  GTATTCCCAGGTCAATGCAGCAAAGTTGTTTAATTGCCGCGTATCGATCCTCTCGTTGTGAAGATCCTGGAGTCATGACCACAATTAACTCAGCGCTTTTATCAACCTCTCTAAGTGTCTGTTTGTATGTGTTGGCGTGGTCATCTCGAAGTTCAGTTTTTCGAGGATTGTCAAAAGAGAAACCCAATTTTCGAGCAACTGTTTGTAACATTTTCAAAAATTCATCGGCTTTAGCTCGATCTCGAGCATTGTAAATGAAGTACCAACGTTTTATCGGTTTAGATGCCAAAATGACAGCGTCTTTAATTTTCATTCCCCATTCTGCTTTATCACCCGCTGAATGTTCAACACCATTACCAAAGGTCAGCTTTTCATATCCAAATTTACGAGCGTAAGTTTCAAATGGCCTGGCATCAATTTCCATACCCCAATCGGTGAGAATTTTTCTCGAGGCTTCACAAGAATTTGCATGCTGAATAAAATCAACTAATCGTTTATATTTAGCCTCAGGCGATATCTTCGTATGTGTAGCCAAATCTTTCATAACCCGATGGTCAGAACGAATATCATCAGTAAGTCCAGTTAAATATGACAACTCAGGTACCAAACAAATAAGAGGTGATTCCTCGGTTTGATTTCGCCTTGGTTTTGGTCTATGGAGGAGTAAAGGTTGTTTTCCATCTTTGATATCGACGCCCCAATGTTGTCGGAAATAATCAATGAAAGTGATCTCTTGACCTTCTTTCATGGTGAATTTGGATGAAGGGGTTCGAGTAAAGTCAATGTCGTCGATTCGATAGGCACGGTTATTATATCGAGTTAGAACAATCGCTCCGACGAGTAACTTGTTTGCTCTTATAGCAAATTTCGAAGGATCGTCTTGGAAGAGTTCGTGTAACATTTCACGGGCCGTTTTCTTACGAAGAACCCGATGGGCAGTGTCCACGTTAAGCATAAGTCCACCGTCGTACTCTTGGATCGCACAAACATAACCAGGCCAGACTTCTAGTTTATGGGCTGGAACATCAATCTTACCGGTGGGATTATAATGGTGCATGTTTAGTTCAACCAATTGCATCGCTCTCATCACCTTGTTGAATAGATTGTTGTAGAAATTAACTAATTCTTCAGTCGGAGGCACTTTAACATGAGTGATGGTTAGAATGACGGGAATTTCGACCGCTGGATTAACGGTGGAAATGATGCAAGGCTCTTCTTCCAATTTGTGAGGAAGGAAAAGATTAGTTCCAGTGAAGTTGGAAATAGTTCCAATCTTTTCTTGAACTTCAGGAGAATTTAGAATTTTTCTTCGCAAGGTTATTGAATCGATATTCGGCTCGAAGAAAACTGCGTAAAGGTGAATTTTTTTCTTGGATTTCACATTAATCTTCATATAATTTACAGCGACTTTAAATTTCTCTCCATCCTGGCCAACAACTTTGTTCTCAATTGGTCTCAAAGCCGAAGGAACTGGTGGACCAGCGATGGATTCGGTTGTAGTTGAAGAGACCGAGGGAACTGGTGATCGAGATCCTCCTGATGCTGGTGAAGAATCACCCGTTTCACTTGCCGCCGAAGATTGAGCTAAACTTGACTTCGGACTTGATTTCGGACTTGATTTACTGCTGTCACTAGTCTGAGTCGATGAATCTTCACTTTCCGATGGTTCAGGAGATTGGGATCTCGGAGTATCAGGTGGAGTCTTTTTGATGAGAAATCTATTAAATTTACCACGAATTTTAATATTCCGTTCTGGACTATCACCTCGAGATCCAGACGGAGACGAAGGCGCAGCAGGGGAATTGGAACCATGTGAATCGGTGTCAGAAGCTGAAGCAACAGATTGTTGTTCCGATGGAGGGTTAGAGGCTTCTGACTGACCGGAAGCAGGAGTTGACACCTCGGAGTCCGAAGTAGATTCAACCTCTTGTTTGGAGGTCGATGCTTGGGGTGGACTTCCTAGTCCTTGTTTCGCCTCTTCCTCTTCTCGGGCTTGTCTTTGCTGCTCAATTTTCAGCCGCTGAAGTGTCTGAAGGAGTAACTGTTTGCCCCCTCGGCCACCGGGTTTTCCTCGTCCAGCCATAGATGAGAAATGACACTTAACGAATATAATCAAAGTCCGATGGTAGAAAAAAAATGGAATCAAAATGGAAAAACAGGCGAAATCAAAAGAGATAATATAAAATCCGTTCCGATAATAGAAAAAAAATCGAATCAAAATGGAAAAACAGGCGAAAAAGAGATAATATAAAATCCGAAAGTAC  >Unigene32093_All Probable ubiquitin carboxyl-terminal hydrolase FAF-X-like isoform 2 [Pongo abelii]  CCTTCATTTTTTGATTCTTTCTTCAATTCTTCATCATTTAAACTTTCCAGAAAGATAGGCACACTTTCAATAATTGATAGAAAATAGGTTATCACTGTCTCAACGGTAAGAAGCTCATAACATTGTCCAAATGGTTTGAGCAAGGCGGCAATCAATGGCACAGTTAATGGTGGACCTTTTTCAAACCTTTCCATTAACTTTTGGAAACCATCTAAACGACCAAAATAATTAATTAGATCAACTAACCATCCTTGAGGACAACGAGAATCAGGAGATTGGGCATAAACTTTTTCTTCTCCATGATTATGAGATTGGTTAGATGAAGATGATGAAAGGCTCTCAAGGCTATTCATATTGAACATGTGAAACTTATTGTTTGGGTTAAAGACCATAGCTAAAAGGTCCAGAGTAGGGAAACAATCATCTTTAAGTTTGATAACAATTAATTCCAGAAGGCGTTCACAGTTTTGGAAAATGCATTTATGAATTCCAAATTTCCAACCGGTGACTGCTTCATCGGTTAGAATACGAGTAAATGATATGGTTAAACCTTCTCGGATAAATCTTTGGCAAGGCTCACATTTGGTATCTAAACCACGGGCAGCTAAATCGGTAGAAGCTCTTAACAAAACCTCTAAATGACCCTCAGGTAAAACAGGAATGATCCACCGAGGCCAGCTGATCATTTGTTCCAATCGATTTAAATCAGCAACAGGGAACAGTGAGATTCTTGCTTCATCTGAGATGTCCTCTGAATCTGATGATGAATCTGAATACATAATATTATTTTATAGACAATTTATTGTACAATTATGTAGAGTCAATGATGTTGGAATTTTGAAAGACACATGTCAGATTTGA  >Unigene3411_All Piwi [Botryllus primigenus]  GGAATTTGACCCTCTGAAACTCCATCACGATAGATGATGATACGAGCTGG  CCATTCATTGTTCTTTTGATGCCAATTCTTCAATCCAGCTCTGAAGTTAT  TAGCAAAGTTAGCTGACAATTCCTCTCGATTTTGATGATAAGTAGTTCGA  GAGAACCAGGTAGTAGCAGAGTAATTAGTTGAACAAACAAATCCACCAAC  AGATTGGCCTCGTTTCTCAGAGTCATGGTAAGTATCATATCCGATTATCA  TGGTACTTTTTGGCGGAATGTGAAGTCCCCAGGGTTCAGCTCCAAGTTTG  GTCGACATTTGGATGAGGATTTTAGTTGCAACGGACATTAGATTTTTCTT  GTTGCTTAAAACTCGAGTAGTTATAACCTGAGATGGAACAGGT  >Unigene6479_All Probable ubiquitin carboxyl-terminal hydrolase FAF-X-like isoform 2 [Pongo abelii]  TATTGTTGTTGATGATGTTATTGTTGTTGCTGTTGTCGTTGTCGTTGCTA  TTGATGATCCACCTTCAGGTTTATTACCAAGTCGCATGAATGAATTTGTT  GCCACACTGGCTATCACTTTGTTTATTTCATTGAGAGCATTCATTCTACC  ATTGAAAGATGAAATTTGTAATAGACGTAAAATTATTTTGAGACGAAATA  TTTCCAACTCTCGGATTCTTTCCTCTTGATTAGGTACACGAGATGCAAGA  TTTTTTAAACTTTTTACAACACCAGAGAGAACATCATTTTTCCCTTCATT  TTTTGATTCTTTCTTCAATTCTTCATCATTTAAACTTTCCAGAAAGATAG  GCACACTTTCAATAATTGATAGAAAATAGGTTATCACTGTCTCAACGGTA  AGAAGCTCATAACATTGTCCAAATGGTTTGAGCAAGGCGGCAATCAATGG  CACAGTTAATGGTGGACCTTTTTCAAACCTTTCCATTAACTTTTGGAAAC  CATCTAAACGACCAAAATAATTAATTAGATCAACTAACCATCCTTGAGGA  CAACGAGAATCAGGAGATTGGGCATAAACTTTTTCTTCTCCATGATTATG  AGATTGGTTAGATGAAGATGATGAAAGGCTCTCAAGGCTATTCATATTGA  ACATGTGAAACTTATTGTTTGGGTTAAAGACCATAGCTAAAAGGTCCAGA  GTAGGGAAACAATCATCTTTAAGTTTGATAACAATTAATTCCAGAAGGCG  TTCACAGTTTTGGAAAATGCATTTATGAATTCCAAATTTCCAACCGGTGA  CTGCTTCATCGGTTAGAATACGAGTAAATGATATGGTTAAACCTTCTCGG  ATAAATCTTTGGCAAGGCTCACATTTGGTATCTAAACCACGGGCAGCTAA  ATCGGTAGAAGCTCTTAACAAAACCTCTAAATGACCCTCAGGTAAAACAG  NNNNNNNNNNNNNNNNNNNNAGCTGATCATTTGTTCCAATCGATTTAAAT  CAGCAACAGGGAACAGTGAGATTCTTGCTTCATCTGAGATGTCCTCTGAA  TCTGATGATGAATCTGAATACATAATATTATTTTAT  >Unigene12751_All GG12359 [Drosophila erecta]  TTCGTTTCCCGATTGCGTGAATTTTTCAATTTCCATGCGTGATAAACTTT  TAGTCGTCGATGAAACTCTTCCTTACATGCTTCAAGAAGCTCAATATCAC  AGGAAGTGTTGATGATATCTCTCAAATCAGAGTACTTCCATTTGCACAAA  TCGTATTTTTTGTCTCCTGGTAACTTTTTCAATCCAATACCATTTGATGC  ATGAGGAGGTGAAGCGGGAAGTGGACTTGCTCGGGTAAGATCATCAGATG  TACCATTCGTTGTTTCTTGAGCCAATCTCAAAGCAAGAGCATGATCAAGT  CTCTCTTGTTCAACTCGTTTTCTCTGCTCTTCCAATTCCAGATCAAGTTC  ATGTTGTAGCTTTTTAGCCAGTTCGAAGTCATCAACTTCTTTAGATTTAA  CTGCAGCTTCCATCTTGAAACGTTCATCGAGTTCGGCTCGTTTTCGTTTA  AGTTCTTCTTCTTCAGCTTTCCGACGATCTTCATCCTCTTTCTTCATCCT  TTCACTAGCCATTTCTTCCTGAACTTTTCTTAGTTTCTCTTCCTCGACAA  TTTTACTTCTTAATAATTTAATCTGCTCTTCTAAAGCTCCACTAATCCTA  TTCACTCGATTCTCTAAATCTTTTGTATCAAGT  >Unigene14046_All Similar to 2410104I19Rik protein [Strongylocentrotus purpuratus]  CTTCCGTCTTTTCAGCATTTTTTTTTTTCAATAAGTAAAATATTTAATAG  GTAGAGATTGAGACAAACACGACGATTGTTGTTGATGATGAGGTTGATTT  AGTTATTTAAACCGTTTCGATTTGGCTTTTGGCTTTTTGTGTGAGTGAGT  GTATGTGTGTGTGTGTGTGGTTGTGTGGTTGTGTTTGTATTTTGTAGTTG  TTTATGAGAAAAGCAGGAGAGAGAGAGAAAGATAAATCTTCTTTTTTTCG  GGTAATCGTGGAGATGAATTAAATATTATAAATTAAATCACATGCTTTAA  CCAGAGAGATCTTTTTCATCGTATAGAGTTTGATCGTAAATTTCGGCTTT  CACAATTCGGCCTCCGAAAAATCTTCCATTTAATGAATCACGAGCTTTTA  TGGCTTCTTGACTGTTACTGAATTCAACGAATATTTTGACAATCACTTCG  GCATCATCATCCTCTGACTGTTTTTCTTCATAAATGATAACACGATTAAC  TGAGCCAAATTTACCACATTCATCGGTCACTTCAGATTCTAATGCATCGT  CAATATCTTCTACTCCGACCATGTTTCGTAAAACGACAACTCGAGACTCT  GTTTTTCGCATCAATTTTTGCATCAACATTTGTCGAGCATTAGATCCTTT  GATTACAACGTTCTCTTGCTGCTGTAAGGTTTGTGGCTCTTCTCCGGGGT  TACTTTCTTTAGCTTCGATTTCTTTGGAAGCAGCGAGTGCTGAAGCTTTT  TCTTGAGCGGCTGCAAGTCCACTTACAACCACCTTATTTACTAAATCATT  GGTGTTTGATGCTGAGGAGTTATTGTTATTGGTACTATTATTAAGTGTAA  TTGTTGATAATGGTGTTGTTATTGTGCTATTTGGTGAAACTGTAGTAGTA  GTTTGTTGAGTCACTATTGGGATTGAGAGAGTTGAGATGAGAGTTGGGGC  TGGATTTACTGTCACACCGGTGATGATTCCTGGTGCATTTGCGGCTAATA  CGGGTTGTACTTGTGTCGCTGCTAAAGCCATCAAACTCGTTGAGCCACCA  ATATTTAAAGATGTAGGAAGTGATAAACCGGTAGCAGCAATGGCTGTTAC  >Unigene17383_All ATPase family, AAA domain containing 1 [Taeniopygia guttata]  ATTGTCTTAATTGGTGCAACGAATCGTCCTCAAGATGTTGACAATGCTGTTTTACGACGAATGCCTTGCATGTTCCAAATCGATTTACCAAAGGAAAATGAACGACGGGAGATACTCAAATTAATCCTTAAGAACGAAAATTTGGCATCCGATGTGGATCTTGGAGAGATTGCTAAGCGCACTGAGGAATATTCTGGAAGTGATTTAAAAGATTTGTGTCGTCGAGCAGCAATGAAAGGCCTCAAAGAGTTGACTAAATCTCTCTGTAATTCAAGTAGATATGGAGCATCTTCTTTAACACCTTCATCAACATCAGTTGATCAATCAGTATATGTTGAGCAAATTTTATCAAATCAACAGGATCCATC  >Unigene17748_All Similar to stromal antigen [Acyrthosiphon pisum]  GATGATTGTTTATATAATTTACAAAAAAAGATGATTTGATTTGTTGATTAAAATATAAATTGATGCGTTAATTAAAAATTGGGATAAATGAATTAAGTTATTCAGCGATTTCGGATCCAACTTCGGATTCACGGTCAATCTGGTCTTCTTCGACTCCTTCTTCGTCATTATCATCACGATTTCTTTTTCTACCTCTGTATTGTCGTCCTGGAGCTCGATTCAGAGGAAGATCGAATTCGTTATGTTGTATCAAACTACTTCGATAGCTCATCAATGGCTGCCATTCGTCGTTGTGTGTTCCTGGTAATGCACTTTGAACACATTTGTCCAAATAAAGTAAAACAGTCTTTTTATCTTGTTTAATCAATTTGTTTGAAAATTCACTAAGAATTTCAAGAAAAGGTAAATTAGGTGGAGGTCCGAGAGGATCAATTGGATTGTCCAATTTATCAAAGCTAAAATGAATTCCTTCTCGATGTAAAGATGCTACGGCATCTCGATTTTTTTGATTATCAAGACCAAAAGATAAAGCAAATCTTTTAGCCAATTCTTTCAATGCCAAAAAGTTTTCGTCTTGTTTTGTAAATGCATTTGCGTTTTGATGGGATTCCGTTTGTAGCTCTTTGAAAACTTGGATCAAAGCACTGGCCATTGTTTTCGCACAAATGACCTTATTCATTTCTCGTAATTTACCAAGTGTGGTCTTGATGATATCTCCAAAATCGTTGAAAAATCTAACATAGTGCCTGATAACACAAATAGCATGTCTCATTGGGATTACGTTATAAATGATCAGTTTACAATATCCAGCGAGCAAGTGCCTTTTCTTGTGAAGTCGTTCAACCGAATTTTGGATTCCAGGCTCATCCTCAGATTCTTCATCATAAACAAAAACTCTCTCCATAATGAAAGTTTCCAATTGTTTGATCAAAGCGTTGTTCGGTCGATAAACTAATTGGGCTACTGCTGGTGAATCATTTTCGAGTTGGTGATCAAACATGAGAAGTAAATCGCAAATACTGAAATAAGCTTCTTGAGCTAAGTCTTCATTAGGATGGGTCAATATGTCACGTAATTCAGCAATAAATTCAATCAAACGATTACGAGTATAATGATCAACAGGTCCTTGATTTCGAGCTTCAGAGAGAGTGTGCAGTTCCCAAATCAAAGCATAATAACAAACACTTATCGAATACTTAATAGCTTCTTCGGGAACACTGTCAGCGTTTTCTGATCCAGCTCCTCTTATCCATCGCTCGAAAATTGTGTCCCACATTTCGTAAGAACTCATATCATGACAGGATATGAAAGCTGCAATCTTTTTCGAAATCATGACAACAGCTTCTCTCTCATCTCGTCCATTAGGGCGTTCAGAATAAGCTTCGACTGCATCTTTGTGTTTTCGAAGAACCAGAGTATCCAATAAAGAACTTTTAGAAACCCAGACTTCTCTTTTGAATGTGAAATTTTCGTCGTACAAGTACTCATATGCTTTGGCACAAGCTTCCAAAACTTCCGAATCCGAGTGAATTTCAACAAGAACATTTATCAATCTCAAAAGAGATTCAAGTTGATCATTAGTCTGTGAATAATAACTTAGCTCAAAATATTGAGGGATAGTCATTAGATTGGCGATTTTTTCAGGATCGGTCTTGTATTTGTCCAAAAGTAAAGGAAGAGCAACAATAAAATGCTCTGAGATACGGTTCTTGTCATCGGCCACTTGTTTAGCTTCTTTGGTTGATATTTGTTTTCGAACGGGACCTCGTCCAATAGGATATTCTCCCGTTGCCGCTTGTTTTATTGCACAAACCATAATCTCAATTAAACTGGTCTCTTGTCGATCATCTAGTGCTTCTTCATCAGGCCCTGGCTCTTCGAGCAGTAAATCAGTCATACATTCCCAATCTTTCATCATCGGGTGACTCTCGATTAAACTGTCAACCAGATATGCACCATGTTCATGCAGCTCACTTTCAATGAAGAAAGTTATCAAATCTCTAAGTAATGGAGTGTGAACCGATCGCTTTTTACCTCTTCGAGAGCATAAATTTGCTGTAACTTCTTCGTCAACCTGGAATAGTCTTTCATTCAAAAATTCACCAGCAGCTTGAGCGACATTCCGATGAGTAGCGTAAACCAACTCGTAAACATGTTCGCAATCTTTATCAGTTAAAACTTCTCGGTGATACTTGTGAATACTGATGATCAATTTGACCGCTTGAACTGCGACCTCATATTCTTTGTCCAATGACATGGCGACAATTCGTTCTTTGAACTTGTTGGTAAACAATTCCATTTTTCTTGCAATTTCTTCAGTTTCATAAAGTGGTAGCAAGGATTGTAAACATTTAAGTCGCACATCACCAACTTTATCATGTAATGTCCACCCAACGTATTTGAGGAAAGAATCATCGAGGAAATAATTTGGAAATCTTTTCATCCAAATGCCAATTTCATACATACACAACGCTCTGATTTCTGGAATAATGTCTCTGTATCTATGGACGAAAATTGATTTGAACAGATATCCAAGCATAGTTTTGATATCTTCCATGTTGTCATCAAGTTCTTGTCGTTTAATTTGCAAGCCATCAAGTTTATCTCCTCTTCCTCCTTTTTCTAGACGGGCTTTGTCATATTGACGCTGAGTATTATCAATGTTGATACTAAGAGTAAGAGCGACATCAACTAATGCAGTCATTAGTTTCATAGCTGCTAAAGTTGCTGTATGAGTTGATACTAAGAGTAAGAGCGACATCAACCAACGCAGTCATCAGTTTCATAGCTGCTAAAGTTGCTGTATGACGGAAAGCTCGAACTTGAGAGTCAGATAATGTGATCAAGAAGGAAATCATATTCTCCATAAGATACTGGTCATAAATTATACTGTATTGACATTGTCGGATTAAAGTGTGGACAAATTCACAGAAGTTGGACCTAAATTTTTTCCATTGAGGGCCATTGGTAATTATTGGATAATCTCCACCAGCCTGGTCATCAAATTCTTCAACCAATTCTCTGATGATTCTTCCATTGTCCATAGTTTCTTTCATTTGTGGTGTTATTTTTCCCTTACAACCAGAGGCTAAGCTGAAAAACTGTAGAAGCTCAAGAGTTGCCGTATTTCTGTCTTGTCGATAAGATTCAATCCATTCATCAACAACAGACTGTAATGCAGCTCGACCTTTTTTGACACTGGCAAACAATGAATTTCCATATGATTTAAGAAGCTCAATGTCCATTTCATCTTGAACTTTGCCATCTTTATC  >Unigene19525_All Stromal antigen [Culex quinquefasciatus]  CAATTAAAAGATTTGATCTCTTTCTTCATTGAGTGTGACACTCATGAACATTGTGCTTATCTGGTTGACAGTTTAATCGATAGTCACCCAATGATTAAAGATTGGGAATGTATGACGGATCTTCTTCTAGAGNNNNNNNNNNNNNNNNNNNNNNNNNNNNNNNNNNNNNNAAACAAGAATCTGTACTAATTGACTTGATGTCTTGTGCTGTTAAACAAATATCCACTGGTGAACCTCCTATTGATCGTAATATTAGAAGATCAAAAATTTCGGCTAAAGATTTGGCTAATTTAAATGAAGAGAAGGAGAAAATTTCTCTACACTTTTCTTCCGTTATTCCTGATCTTTTGAGAAAATACAAAACGGACACACAAAAAGTCATCGATCTACTGGAAATACCGTTACATTTTCTCAAAAATACTTATTCACAAAATTCTGACGATATGGAAAACCTATTGACCGTTATCTCTGAACTAATCGAGATTCATTCGGAGACCGAAGTTATCGAAAGTTGTGCTCGTATCTATGAATTTATGCAAGACGAAAATTTTACTCACTATCGATTGGTTCGAGTTGAAGTGAGCACACTTTTGGATAAACTGTTACAAAAGTATAAGGAATCACTTACTGGCCTTTGCGAAGGATCTGATGATAAATTGGAGACAATTAGTTTTGTGACAAAGAAAATTGC  >Unigene19859_All Similar to Cniwi [Hydra magnipapillata]  AAGAAAACTAAATTGTTCGAAAATCAAGAAAAAGTTGCAAAGATAAAATAAAATTCGTGAAGCAAAATCGATAGGAACTTAGAGGAAGAAGAGAGTGTGTGATAGAGCGAGATGATGCTCTTTGTGCAAAGCGGTTCCTGTAAGATGGGCCAATTTGTGGGCATATTGACATGGAGCTGGGACAGTAATGGTTCCAGCCCAATTGTAGTAAAGATGGCAAAGTTGCAAAGTTTATAAGCCAAAGCCAATTGTTGATGATGAAGCGCCTTCCATCCTGTACTATCAGCGATGATGTTGTACATTGTTGGGTTTACGGTACCTTG  >Unigene25015_All Similar to Cniwi [Hydra magnipapillata]  ATCAGTCAATCCGTTCGCCAAGGTACCGTAAACCCAACAATGTACAACATCATCGCTGATAGTACAGGATGGAAGGCGCTTCATCATCAACAATTGGCTTATAAACTTTGCCATCCAATTGGGCTGGAACCATTTACTGTCCCAGCTCCGTGTCAATATGCCCACAAATTGGCCCATCTTACAGGAACCGCTTTGCACAAAGAGCATCATCTCGCTCTATCACACACTCTCTTCTTCCTCTAAGTTCCTATCGATTTTGCTTCACGAATTTTATTTTATCTTTGCAACTTTTTCTTGATTTTCGAACAATTTAGTTTTCTTATATACGATTAGTTTTCTATTTCTTTTATCATTAGTTAATTAGTTAATTCATTCATTTATCATAAC  >Unigene3540_All Beta-catenin [Aplysia californica]  CTTTTTGAAAGTTTCTTTTTCTTTGTCAATTATTTTTTTACTTTGTTGTT  CTGACAATACAATTAATTGTTCATCAGTATGACTTCATTGGAATTAAATA  GATCTGAGCGTATTAGAGCTGCTCTATTCGATGATTTATCAAGTGACATT  TTATACACAACATCGATCGACAATAAATCAGAAACATGTGTCCTTAGACT  CTCTGAACCTGCACAAATGATTAAATTGGCGATTGAAGCTTTGATTAATT  ACCAGAATGACGCTGATTTGGCAACAAGTGCTGTTCCTGAGCTGTTGAAG  CTATTAAATGATGAAGATGAAGTGGTGGTTAACAAAGCGACATTAATGGT  TCACCATCTTTCTCGAAAAGTCGCTTCCCGACACGCACTCATCAATTCTC  CTCAAATGGTGTCCGCTCTTCTTAGGGCGATGTCAAATGCCGAAGCTGAA  ACAACCACTTGTGCTGCGAGTATTTTACATAATTTATCTCAACATCAACA  AGGTCTTCTAGCGATTTTCAAGTCCGATGGAATCTCTGCCTTAATTAAGC  TTCTCACCTCTCCAATCAACCGAGTTGTTTACTATTCCTTGACAACGATC  TACAACCTGTTGCTTCATCAAGAAGGATCAAAAATGGCCGTTCGACTCGC  CGGAGGGATCCAAAAGATGGTCACATTGTTGAATCATCCCAATCCTAAAT  TCCTCGCTCTTTTGACCGAATGTTTAACTTTACTTGCTTATGGAAATCAA  GAGTCTAAGTTGATTATTCTCGCCTCAGGCGGTGTTCCAGAGATTGTGAA  CATTATGCTTGTCCATAACTATGAGAAATTACTTTGGACAACATCTCGAC  TACTCAAAGTTTTGTCCGTATGTCAAAGTAACAAACTCGCGATCATCCAA  GCCGGAGGAATCTACGCTTTGGATATGCACTTGGAACAACCCGGAAGATT  ACAATTAAGTTGCCTTTGGGCTTTGAGGAATTTATCTGATTCTGCCACCG  ATATTCTCAACATTGATCATCTTCTCCAAGTTCTACTCAATTTACTCAAA  AGCGAAGATCTTAACGTTGTTGTTTGTTCGGTCGCTATTCTATCCAATTT  GACCTGCAACAATACCCAAAACAAACTATTCTTATGTCAACTAGATGCAA  TTGAAAAGATTGTTACAACCATCAAACAAGCTGGAGATAGACAAGAGATC  GTTGAACCAGCAATTTGTGTTCTCAAACACCTGACACATCAGCATGAGGA  AGCAGAAATAGCCCAGAATGATGTCCGTCTTCATGATGGTTTGAATTATA  TTGTAAATTTCTTGAATCTACCGATCAGTTGGTCACTTATCAAGGGAATT  GTGGGATTAATTCACAATCTGGCTCAATGTCCAGCCAACCAAGCGCCATT  ACGAGAAGCTGGAGTTATTCCCAAGTTGAATCAAATTTTATCCAAAGCTC  AACAAGAGAGAAACTCATCTTCAGGATACATGGATGGTGTTAAAATCGCC  GATGTCATCGAGGGAACGATGTATTCATTCAAGATGTTAGCTCGAGATGT  TCATAATCGATCCATTATCACAGAAATGAACTTAATCTCAACATTCGTGC  AACTTTTATATTGTGACGATGAGAACGTTCAACAATCTGCTGTTGGTGTT  CTCAGTGAACTGTCCCTCGAAAAAAAAGCCGTTGACATTATTGAATCCGA  AGGAGCTACTGCAGCTCTTACTGAACTTCTCCATTCTAGAAATGAAGTGA  CAGCCACTTTCGCCGCCGCTGTTTTGTATCGAATGTCCGAAGACAAACCT  CAGGATTACAAGAAGCGTTTATCATTGGAACTGACAGCAACCCTTTTCCG  TGACGAACCCAATGATTGGACTTCAGCCGGTGAAGTGGAAAACGAGGTCT  TACCACAAGCTGAATCTCACTACGGATCACTCGCCTCTCCCCTCAATCCT  TATCCACAATCAATTTACGTCCAAACCACATCAGATATGGATGTTAACAA  TCCCTATTCATCACCATATTACAACGAACAGGACCAAGTCGCCAATAATG  ACCAATACTCCGCTTGGTACGACACCGACCTTTAACTTATCAACAATCAA  ATCAAAAACCGTTTCCAGTGACTTATCACCAATATTTGAGTGGCCTTATC  AGTTTAATCTTATTACTCATATACAAATCTGACACTTTAAATAATAATTT  ACATTTTAATTTCTTACTATTAATATTTACCCAAT  >Unigene6665_All Similar to myosin vi [Tribolium castaneum]  TTTTTGGTTAATCACTTTTTCCGAGCAACACTTGGGTGATATTTAACTGA  TTCTCCTCCATTTTTGGACCATTCTTTCTCGAACTCTTCAGCCAGAATTT  CAGCTCCACGCTTACTGGTCAATCGAGTTTCATCCAGACTCAATTCACAC  ATTTGCATATCATCTTTACCTGCAACTAGAAGTATTGGTTTCTTTTCCGG  GTGAATTTCCATTTGCCGAGCGATCCATTGACCATCGAAATGAGCCCACC  ACCAGCCTCTTTGTCCGGTCGCTGATGAAGGTCGAACAAAAGGAATTCTA  AAGTATCGCTGTTCATTGTTAGAGAATGATAATGTCGCTCTATTCAATAT  TCCATCGTTTAATCCGTGAATATCAACCGATCCTGGATTGTTCATAATAG  AATCTGGAGCTCGTTCTTCTTCATTCGTTTCCCGATTGCGTGAATTTTTC  AATTTCCATGCGTGATAAACTTTTAGTCGTCGATGAAACTCTTCCTTACA  TGCTTC  >Unigene11470_All Ubiquitin protein ligase [Ixodes scapularis]  ATGGCTTTAAAACGTATAAAGAAAGAATTGGAAGACTTGAAAAGAGATCC  ACCAGCCCAATGTAGTGCTGGACCTGTTGGTGATGATTTGTTTCATTGGC  AAGCAACTATAATGGGTCCTCCTGATAGTCCTTATCAAGGCGGTGTTTTC  TTCCTAACTATTCATTTCCCTACGGATTATCCTTTTAAACCACCCAAAGT  AGCTTTCACAACGCGAATATATCATCCTAATATAAACAGTAATGGGTCTA  TTTGTCTGGATATACTGAGGTCACAATGGTCTCCTGCTTTAACTATATCT  AAAGTACTGCTATCAATATGCTCCTTATTATGTGACCCTAACCCGGATGA  TCCGCTTGTACCTGAGATCGCCAAGCTTTATAAGACAGACAGGGAGAAGT  ATAATGAGATGGGACGAGAATGGACAAGAAAATATGCCATGTGATGTTGA  TTAGG  >Unigene12895_All GL17699 [Drosophila persimilis]  CTCATTTTTTAATTGTGATATGGATGACCATAAACCGGCTCTTGTTGATA  CTAAAATTGAGTCAAATGTTTATGATGAAATCGTGATTGACTCTGACTCT  TGTGAGGAAGATGGTCCAACAACCACCTGTGAAAATGGTCAAACCGCTGA  TGTCAAGTGCGAGGTTAAAAATAGAGTCTTGGATACTTTGACTCTGTCAA  GCGATGATGAAGATGATCTGATTGTTGGTGAAACTAAACCTGTTGAAGAA  AGTGAATCAAAACCAATTGGCCCATCAAAAGAGGTAGTTAGCAAAGTTAT  TGATCAGAAAATTAGTGAAAAGGATGATAATTTTGACTTGCGGTGTCAAA  AAATTGAAGATGGTTTTGAGTTGTGCACCAAAGATTTAAACAATATTTCA  GATGAATTTCGCTCTTGTGATGAGATTTGCAGTAAATTTGCGCCAAAAGA  GACACATATCACCAGTAAAGGAATCATAGATGTCAAAGACGAATCATTAA  CAATCGATGAAACCGTTCAAAGAATCGTCTTAATAGATCCTGTACAGAGA  AAAATTGCCGAAGATTTACCAATAGATAAAGAAAAGTCAACAAAGGTCAA  CTTAGGCCACCTTACCACTGTTACATATTTTCCCGACATTGATGTTGTCC  AAACCAAAACTGCAGCTGATGTCCAAGAGATTTACAAATTACCAGCAGTC  GGGCCAATTGTCAGAAAACCATTAAACGTTGGTGATCAAGTATTCGCTGT  TGCCAATCCAAGTAATATGGCCAAAAGTGGTTGGCAACCTGCAACCATAA  TCGAAACCATAAAAATCTATGGTAAAATAAATTACAACGTAACTTATGAG  GACTCATTTAAACCTTGTATGCTGTTGAAAAAAGCGCAAATAGCTTATCG  ACAGACTCCTTCAGTTATGCTTTCACTCAAATGTCGAGTAGTGGCCGCAG  TTGATGTCAGTCAAAAAGGATCTGCGCCGCGTTTGTTATACTTTTCCGGT  TTAATAGCAGAAATTGCCCGAAGAACAAATTCATTCCGATACCTGATTTT  CTTTGATAATGGAGC  >Unigene12944_All Cytochrome P450  CTCATTCTCTTGGAATTAATTGAACCCGGAAACAATCATTTACTTTTAAT  GCTAGAATACCAAATTTAACATGTTCTCTGATTGTTTCAATTGGGTCAAC  AGGTTCGACCTTGTATTTGAGTAAAAGGTTACAGAGAATAGCGAATGTTA  TATTTTGGGTCAGTTTGAATCCGAGGCAAGATCGTTGACCAACGGAAAAT  GGCATGAAGACTTCGGGCTTTTTGATCTGCCATTTACTTGGGGAATTGTT  GTTAGTTGAATAGTGGAGGAATCGAGAGGGTTGAAATTGTGCTGGTTCGG  TGTAGAAATCGGAGGAAAAGTTCATTCGCCAATTGTTGAATAAAATCATT  GTTCCTTTGGGTACAAAATATCCTCCAATTGTTGTGTCCTGAGTGGGAAC  ATGGGGCACAATTGGTGACGATGAAAGTCTCAAAGCTTCTAGAATTGAAG  CTTCGGCAAGTGGAATGTAAGGTCGATGCTTAATACTAATAATATCGGTA  TCGTGTTCACGAGCAGAAGCCGCTATTTCTTGGTACATTTCCTCTTGTAC  TTCCATGTCCATTGCTAGATGACCAAGGATTCTCATCAACATATTAGAAA  TGGCAGCAGAGCCTCCAACAAGATCACCGATTGACAATAAGAAATGTTCA  TAAGTAAAATCTTCAGGATTTTCGATATGATATTTGTACATTAGGTCAAG  AGAAATCATCGAATCGCAAGAGCTTAGGTCAAATTTTCCTTCTAAAGATG  ATGATTTCTTGATCTCAGCTAATCGAGGCTTCACTAGAACATCTTCAACG  AATACTCTCAAAGAATTAGAGATTGTGTCCAATTCCTTGATATAACCACG  GAAAAAGCCTAATTGCTTTAAGAACGGCATAAAGTCAACTGGACCACATT  GACTGACATCGTAGAAAACGTAATCGTAATTGTGAGCTTGTCTAATGAGA  TTAGGATCATTGTACTCCAGCCTTGAGGCGCAAAGATATTGAGTGAAAAC  GTTTGAAGAAAATTGTCTGGTCCAACATTTAGTCAGAATTAAATCTGAAG  ATGATTCAATGTATTTTAAAAATTTTCCCACTTCCGATGTAACCGCTTTA  TCGAGCATATCAAATGAATTTGAACTAAACTTGGGTGCCGTTGATATCGC  ACAAAGGACACGACGAGTTTTTTGTAGCTCAGACCAATCGCACAAAGCCA  ATGCATTTTGTCGATCACCTCCGAAAATTAAATGATATCGATAGAAATCT  GGCCTATCAGAGAAAATTTTACCTTTGGTAAGAAGAACTTCTTTAATTGT  TTCCAAAGATGAGACCATGATTGCATCGACTGATCCTAATCGGAGAGAGA  CAACATCACCAAATCGCTGACGAATTGCCTCAAATCCTTTCCAAGGATCG  TCAGGGAATTTAGCTAATAGATGAAGATGTCCAATTATTGGAAATCGAAA  TGGTGATGGAGCATATTCACCAAATTTGGGTGCAGATCGAAAGATGAGTT  TGTTCAAGATGAACAAAATGATTCCAAGGCAAAAGATGGAGTAAACAAAA  GAGAAACTTATTAAATCCATTGTAATGGTCAGTTAGTTGGATGGACAAAC  GTGGACACAACTATAATCAAGATGAGGAGAAACTGCTTTTTAAAGACCAT  GGATTAAGAGGAGTATCTTTAAAATGGGAGAACCTGAT  >Unigene12972_All Similar to domino CG9696-PD, isoform D [Apis mellifera]  CAACAATTCCTTCAAGTTCATCCTCACCAACTCCTAACCTTGTAGCAACA  CCTACTACCACCAATAAACAGCATTCCATCGCGTCCGTTTTTGATAACTT  CATCGGTGAAGATGGCGCTTATCGAGCTAAGCAAGAAGCCATTGTCATGA  GTAGAATTGCCGAATTAAGAAAAGAAGGCCTCTGGTCAGCTAAAAGATTG  CCAAAAGTACAAGATCCTCCAAGAGCCAAAGCACATTGGGATTATCTATT  AGAGGAAATGGTCTGGTTGGCCACAGATTTTGCTCAAGAAAGAAAATGGA  AAAAGACGGCAGCCAAAAAATGCGCTAGGATGGTAATGAAATATCATCAA  GATAAATGTATGCAAGCAGAAAAGGCCGAAAAGGAAGAAATCTTGAGGCT  GAGAAAAATAGCTGCAACCATATCCAAAGAAATTAGAACTTTCTGGTCAA  CAGTTGAAAAATTAGTGGAATATCGACAACAGACAAGACTCGAAGAAAAG  AGAAAGAAAGCATTAGATTTACATTTGAATTTTATTGTCGATCAAACTGA  AAAATACTCATCTTGGCTCGCTGATGGCTTATCTAAACCAATTATAAGTA  CAACATCAGGAGGTAGTGATAATGGTTCAACAATTGAATTAAATGCTAGC  TCTAAATTAAGCAGCGTGTCTGGTGATCAGGAATTCGAGCCAAACACGTT  AAATGAATCTGATGACGAAGAAACTATCGCCAAAGACGAAGAGGAATATG  GAACGGAAGATAATAAGAAAGAATTGGATCTGCTTAAAAAAGAAAGTGAT  ATGTTAATTGAAGATGTAATTAGCTCTCAGACTGATAATGTTCAACAAAC  GGAAATAATGACAAAGGAAAACGCTGATCAGACTAGTCGTGATGAAGATT  TCAAATTAGAATCAGCTGAAGAAGAAGAAGAAGACGATGAAGAAACAATT  GCTGAACAAGAGAAGCAAGAGAAAAATATCGATTATAGTGGTGAAATTAA  GGAATTAGAAGATGAGGCCAACTTATCCATAGACGAATTAAGGAAAAAAT  ATACAGATCTAGATGAAATCGTTCTCTCCGATGGCGAGGAAGGTTACGAA  AGTACCGGAGAAAGTGACGATGAGACTAATGAAGATACTGAAAATGATGA  CGAAGCTTTAACAGGAGATGAATCTGAACCCGAAGAACTTGGTATGGAAT  CGTTACTTGATGGTGACTCAGAAAGTAAAGAGACTCCATTGATTGAAGTC  GTTTCTTCCGATGGCAAGAAAGTTGCGGCTGATCCAAACAAAGACATCAG  TGATATTGCCGCTGGAGCTGAAAGTATTAAACCAAAAGGATTTACATTAT  CCACAGCGAATGTTGTTACCAAAGTTCCTTTTCTTCTTAAACATACACTC  AGAGAGTATCAGCATATCGGTTTAGACTGGCTCGTAGCGATGTGTGATAA  AAAATTAAATGGTATTTTAGCTGATGAAATGGGTCTTGGTAAAACGATTC  AGACCATTTCCTTATTAGCTCATCTTGCCGTTGACAAGGGTATTTGGGGT  CCTCATCTTATTGTTGTGCCAACAAGTGTAATGCTCAATTGGGAAATGGA  GTTTAAAAAATGGTGTCCCGCCTTTAAGATACTTACTTATTATGGTAATC  CGAAAGAAAGAAAATTGAAAAGACAGGGTTGGACTAAACCAAATAAGTTC  CATGTTTGTATCACATCGTATAAACTTGTTATTCAAGATCATCAAGCCTT  TAGGAGGAAAAAGTGGAAATATCTTATTCTCGATGAAGCTCAGCATATCA  AAAACTTTAAATCTCTTAGATGGCAAATGCTTTTGAATTTTAATTCATCT  CGTCGTCTACTTCTCACTGGAACTCCATTACAAAATAATCTCATGGAACT  TTGGTCTCTTATGCATTTTCTGATGCCCAATGTATTCGCATCTCATAGAG  ATTTTAAAGATTGGTTCGTGAATCCTGTCACCGGTATGATTGAGGGTAAT  CATGAATATAATGAAAATATAATTAGGAGATTGCACAAATTACTGAGACC  ATTTCTGTTGCGTCGTATCAAAGATGAAGTCGAAAAACAGTTACCAAAGA  AGATAGAACATGTGATCAGTACTCGTCTTTCCAAGAGGCAAAGATATTTA  TATGAAGAATTTATGAGCCTTGCCAAAACCAAAGAAACTCTTGCCTCTGG  TAATTTTCTGAGTGTGATAAATGTTTTGATGCAACTAAGAAAAGTATGCA  ATCATCCAAATCTGTTTGAACCGCGGCCAATTACCTCTCCGTTTTCCATG  GAAGGGATCACTTACCATACTGCATCATTAGTTACTACACCTTTGGATTA  TGATCCATTTAGGAATGTCAATCTAGATTTTTTAAATTTAAGATTTGTCA  ATTATTCTGTTCCTCCGTCTTCACTTAGTGCAATAGCACAACATAGAATA  AGTGAATACCAAGCATCTCATAGATTAATTGAAGAAATCGATTCAGCTCC  TCCCCCACCACCCAGAATTCCCAAAGCAAAGCTACAGCTTCGCATCAACG  TTAAAACAAATACATCTGCCATCCCATCTCCATCAATACAACTTCAGAAA  GGTTTTCAAATTCAATCAAAAACATTACCCTCTGTACAGATTAGATCTTT  TAATAATATTCCATTGATTGTTGGCTCTGGTCAAACTCAGACGGCTCAAT  CAAATTCAATCCAAGGACCTAGGTTATTTTTATTATCCGCTCCAAATAAT  TCAGCTTTTACTCCTTTAAAAACCAGTACAACAATAGGTAACTCAGGTGT  CATTGTCACTGACAATAATTCCTCAGTATTGGGTGGGACTCCTGGAAGTA  TTGTAAAAAA  >Unigene13786_All E1a binding protein P400 [Aedes aegypti]  GTTTTTGTTAAACGATGAACATCAGGACCACCGGTAAATAGACCTTTCAA  TTTTTGTAGACGAAGCTCACGACGAAGTTTAAGCAAAGCTGGAATTGTAT  TATCAAATAATGATCTAGGAATATTGATAACTTCATCTTTGCGTAACTTG  TGTTGTTTATTACGAGCAATGACTCTATGATCTAATTTCAAACGTTTGTT  TTCTTTGGCAACATAAACAGGGGGGACTTGAGATTCAGGCATAACCGAAG  TTTCGTAAGTGAATGCCAAAGAATAGTCCATGTAAAGATCATTTGAGTCT  TGTGGTGGTGTTGGTGGAGTCCAAACAGGCATTTGCTCCATTCCAGTTGA  ACTGTATGAGATGTAAACC  >Unigene19977_All Cytochrome P450  GCCACAGCTCTTCAACACAAAACTGATACTTTAACCTTGAAGCACCGACCTCATATCCCACTGGTTGACTCTTGTGTCCTGGAAGCTCTGAGAATCGCATCTTCACCAATCGTCCCTCATGTATCAACTGAAGACACAACAATCGGAGGTTACTTTGTACCAAAAGGAACAATGATCTATTTCAATAATTGGCCGTTAAACTTTTCTACCAAATTCTACACTAAACCAGAACAATTCCTTCCTTCTCGATTCGTATGCCCTTCGGACGATAATAATAACAAGACAATGCAGATAAAAAAGCCCGAAGTTCTAATACCTTTTTCAATTGGTCATCGAACGTGC  >Unigene23477_All Similar to tyramine beta hydroxylase [Tribolium castaneum]  ATTCTTCATGGTGGGAACATGGATTTTCTGGTTTCAATCGGATTCTAATTTCGGGCAAAGGAGTTCCATCCCAATCACCTGGAAATAAAAGACCATCCGAGCGACGACACTCCATAGAAAGTGGACTAAGCGAATATAATTTGGCTAAAAATCGAGAACGATAGGGTGTCCATTGAATAGAATGATAATTGTCGGCAACTGATTGACCTGAGGAAGTATTTTGACCTTCGTTTATTTTCAAGTAATCAAAGTAACTATTCAACACGGAAAGATCAACCGACGATTTACAAACTTCCAACGACGATCGAGGATAATAGTGCATATAATTGACACACATCTCATCTGAAATACCATAACCACCGAGTGTTATTTTCTCCCGTTTCGATGTATCATAAAGACAATTGTTAATGAGCACATCACCGGGTAACAATTGGATACGATGTTTAAGTGCACGAATCTCTTGAAAATGGGCAGAATAATGATTATCTCTATTTAATTCAGCTAATTCAATGCCACCTCTAAAATGTGAGGTCCAAACTTTACGACCAGTTAGGTGGGTA  >Unigene26708_All Similar to domino CG9696-PD, isoform D [Apis mellifera]  GTTTATGGTCTGCTAAACGTTTACCGAAAGTAAAAGAACCATCAAGAACCAAAGCACATTGGGACTATTTACTTGAAGAAATGGTATGGCTTTCAACTGATTTCGCTCAAGAGCGTAAATGGAAGAAAGCTGNNNNNNNNNNNNNNNNNNNNNNNNNNNNNNNNNTACCATCAAGAGAAAGAAATGTTGGTTGAAAAAGCTGAACGTGAGGAAATGATGAGGCCGCG  >Unigene2924_All IkB [Carcinoscorpius rotundicauda]  GATTTGTGCGACAATTATTTATTAACCAATTTTAATTGTCGTCATTTTCA  TCATCACAAAATATCTTCATCAGATTCTTGGCCTGAATAATAATCAATAT  CTGAATCGTAGTTTGTAGATGAATAATTTTCGCAATCAACTCCACAATCT  TGCAAGATTTGATAGATTGCTCTTAATTTACCACTGTTTGGATTAACTTC  TAATAATCCTCTGAGAAAATGCATTGCCGTTAACCCTCCATAGGTTCGTA  CATGCGGTGAACATTTACAATTGTAGATTAAAAATTGAATTAAATCTAAT  TTCTGTTGCAATATTGCATAATGTAGTACGGTTTTACCACTTTTACCCTC  CTGAGCATTGATATCAGCTCCATGATTAACAAGATA  >Unigene2928_All RCG26088 [Rattus norvegicus]  GTTCATTAACGATCGTTTGGTTGATTGTGCTCCTCTTAAAAAGTCAATTG  AAGTAATTTACTCAAATTTTCTAATCAAAGGATCTCACCCGTTTGTTTAT  CTCAGTTTAAAATTGAGTCCATCAAATTTGGACGTAAATGTACATCCAAG  TAAAAATGAGGTTCGATTCCTGTATGAAGAGCATATTCTGGGCGAAATCA  AAGATTCCATTGAGAGTAAACTTTCCACCTCGACAACAACCCGAGTAATT  AATACAACATTTTCTCAACCCAAATTAACCATAACTCAACAATCAACCAG  CTCATCATCAAATAAGCTAAATACAACTATTGGTAGTTTAACATCAAGCC  AATCGACGCCCAAAGTTTATCCATCTCAAAAGGTTCGAACCGATACCAGA  AATCAAACTCTCCATGAAATTTGGCAACGGAATCAATTATGTTTACCATC  GGGAG  >Unigene31853_All Ubiquitin protein ligase [Ixodes scapularis]  TTTGAAAAGAATAAAGAAAGAACTTGAAGATTTAAAGCGTGATCCGCCCGCACAGTGTAGCGCCGGTCCGGTTGGTGATGATCTTTTTCACTGGCAGGCAACGATTATGGGGCCACCCGATAGTCCATATCAAGGCGGCGTATTCTTTTTGACAATACATTTCCCTACTGATTACCCTTTCAAGCCTCCGAAAGTAGCATTCACAACGCGCATATATCATCCAAATATTAACAGCAACGGAAGTATATGTTTGGACATATTACGTGCACAATGGTCACCTGCGTTAACTATATCGAAAGTATTGTTATCGATATGTTCTCTATTGTGTGATCCGAATCCAGATGATCCCCTCGTACCGGAGATAGCGCGTATTTATAAAACGGATCGTGATAAATACAGTGAAATGGGCCGCGAATGGACACGAAAATATGCCATGTGACTGCCGCAGAAAAACACCTTGTATTGTCTATGTATTTTCTTCTCTACTGTCTGAC  >Unigene3396_All Similar to spinster CG8428-PD [Acyrthosiphon pisum]  GTTTTGGTTATATTTGTGCTGATTTACTTTCAAGATCATTCGGAGATTGG  AAATATGCACTTCGATTGACTCCACTCTTAGGTGTCTTATCTGTTGTTTT  ATTGATGCTTTTCCTTGAAGAACCGCCAAGAGGCGAGGCTGATGGTGTTG  AGCTGATGGAAAAATCAAATGTTAAAGAAGATTTAATTTATTTAACTAAA  GTTCCCTCTTACATTTGGTCGACGATTGGATTTACTTGCGTTTGTTTCAC  CACAGGATCACTCTCATGGTGGGCTCCTCATTTCATGGAAAATGTTTACA  AACTTCGCGATGGAAAAGACCATGACGGG  >Unigene3759_All Amidophosphoribosyltransferase [Salmo salar]  AGGTTGGGTTGTTTCCAGCGAATCGTGTTCATTTCCGTCGATATGTGCAA  CCCTATTTCGAGATGTCGAACCGGGTGAGATCGTTAAACTTGAACGTAAC  AAGGAGCCCAAATCACTTTGTATTATCCCTCGTCCCGCGAATCACGATTA  TCCGGCCTTTTGTATCTTTGAATATGTTTACTTTGCCAAAGCCGATTCCA  TTATCGAAGGTCAAATGGTCTACACAGTCCGAGTCAATTGTGGTCGTCAA  CTCGCTCGAGAAGCACCAATTCTACTTGACCCTTCAAAAGATTATATCGT  TTCACCAGTGCCCGAATCATCAAATCCAGCAGCTCTTGGATTTGCTCTCG  AGTCTGGTGTACCTTTTGTTGAAGTATTTTGTAAAAATCGTTACGTTGGA  CGGACATTTATCCAGCCGTCGACTCGATTACGACGTCTTGGTGTGGCCAA  AAAGTTTGGTCCGTTGGTTGAAAATTTCAAAGGTAAATCAATCATATTGA  TTGATGATTCAATTGTTCGAGGAACAACAATCGGTCAATTGGTTCGTCTT  CTTAAAGACGCTGGAGCGGACGATGTCCACATCCGTATAGCCTCACCTCC  CCTTCACTATCCATGTTACATGGGGATTAATATACCAACACGAGAAGAGT  TGATTGCTAATCACTTGAATGCTGAACAATTAGCTAATTCACTTGGTGCA  GCTTCATTAAAGTACTTGTCAGTTGAAGGGTTAAAATTGTCCGTTCAAAA  TGGTGTCCGTGAGCGGCAAAAGATGACCAATGAATGTAAACCAATCGGAC  ATTGTGTCGCATGTTTAACTGGTGAATATCCGGTTCCGTTGGACTTTTAA  ATTAACTTGAAATCAATTTAGTTTTATCATTGTTTACATTAATCATCAAT  CATCAACCACCCAATCATCATAATCATCATCATAAATGTCACAAAAACAA  AACTAATTGATCAACAAATTATGAGAGCTGAGCAAATGACTTTATATACA  ATTTGATTGCAACAATTAACTTTAATTTCTCAGGTTAATTTTTCGTTTCT  TCTCCTTTCCGACAAATCTTTGGTTCTAATAATAACATTTTAAATTGTTT  CGATAACATTGGAAACAATTTAAAAATCTTAATCGTTTAAACAAATCACA  A  >Unigene4334_All Similar to histone-lysine n-methyltransferase [Nasonia vitripennis]  GTTAATTAGAACTACAATTAGGTAGACCATTCAGTTGATTTGATTGGTAG  TCCAATCATGGGGGACTTTGGAAATATTTTCGCTTCGTTCATTGATAAAT  GTATATCGAAATCTATGAATAAGATTTGCCAATCAAATCAAATTAATGGT  TCCATAGGTTTTGATGATTCTTTGGCTAAATTGGAAACTGATTTTCGTAA  CATTGACTCGACACTAATTAAATTCAACACTTTCGTTGATGCCATGAAGA  ACGATATTCAATATATCATCGTTAGATTCTTACAAGAGGAAAATGGCGAT  GAAAGGAAAGAAAGTAAATCAACTGATTCAATTGAACGAAATGGAGAGTC  TCATATCAATAATGGTGCAATAATGTTTTCCAATCTCGACAAACCTTCAT  CTTCTAATTGCATGGACCATGTAAATGGAACTGGTCACTCATTTTATGGT  AAAAGAAGGTTACAAATTGCTCGTAAAAGTCTCGCCGTTAATCATCCGGT  TACTCTTCAATTGATTAATTCTGCGGTCAATTCTAATTCTAATGGTGGTC  AATCGTTTCCTAGATTTTGTATGGACTCGATACAAACTTTTCAAATCACT  CGGCGACCCAACATTTTAATTGAGACGAAAACTTTTACCCCTCATATTTG  TGGTCCACTTTGTATCCCTATTACAGTGATTGACCATCAATCAAGTATAT  TCAGTGAGGAAATACCTTATGCAATTCCGATTATTCTTGGTTGGCAGCGA  CAAACGGTAATCGCTAAGATGAAAATCGAACAGAATAAACAATCAGTCTC  TGCAAAGGAAATTATCTATGTTGGTCCATGTGGACGTCGATTACGAACCA  TCGGTGAACTAGATGAATATCTTGCAATGACTCGATGTCCAATATCGGTT  GACTATTTCACCTTCGAACCTTATGTTTCACTGTTCAGTGAGAGCTCGGC  GACACCGTTAAGGTGTAATTGGCTCGAAAATGACATCACCAATGGTCGGG  AGATGAAAAAAATATCGGCTGTAAATACCTGTGATAATGCTCAATTTCCA  CCTGATTTCTGTTACATTCCAAATCGATATGAAGGTCGTAGTGTCGATAT  ACCCTTGGATCCATCTTTCCTAATTCGATGTGATTGTACTGATGGTTGTC  GTAATCGTTCAGCGTGTGCATGTCAAAGGTTAACCATTGAAGCGACTCAA  TCACTTCCCGGAAGGCGGAAAGATTTATCAGCTGGTTACTCTTACCAGCG  ATTGAGAAAATTTCTGGTCACCGGTGTTTACGAATGTAATCCCTACTGTT  CCTGTAACGAAACTTGCCGAAACAAAGTTGTCCAAAATGGAATCAAAGTT  CGTCTTCAAATTTTCAAGACCGTTTCTCGAGGTTGGGGTGTCCGATCTCT  TCACGATATTCCAGCGGGAACTTTCATCTGTACCTATGCCGGTGAAGTAA  TGACCGAACATGAAGCCAATCGAAACGCAACCGTTTTCGGTGATGAATAT  CTCGCTGATTTAGATTTTATCGATGTTTGTGAAACGGCCAAGGAAAACTA  CGAGGACGCACCGTTGGAGGAAATTTCAAGCGATGAAAATTCTGATGATG  ATGACGATGATATTTACGAGGTGGATGATCACGAATCGGATGAATCATTT  TCCTCGGTTCAAGTTAAATCGAAACGGAAAAATAAATCTCATTCAAACAA  TCAGAACAATAAAAAGTCCAATGGTACTAATCAACCGGCTGGAGGCAATA  ATTGTGTTAATAGTAATTCCAATGGGAAAAGTTACAAATATGATTCATCT  AGTAATCAATCTGATTCCTTGATTAAATACAAATCGATACGAACCATGTT  CGAGGAAAATTTTGGTTACACTCTTGATGCTGCGAGATATGGGAATGTTG  GTCGTTTCCTTAATCACTCATGTTCGCCTAATTGTTTCGCTCAAAGTGTT  TTCGTTGACACTCACGATTTACGATTCCCTTGGGTCGCTTTTTTTGCCGA  CAAATTTATCCCAGCATTTAGCGAATTAACTTGGGACTATAACTACGAAA  TTGGTTCGGTGGAGGGTAAAATGATCCGGTGTTTCTGCAATTCAAATGAG  TGTCGATATCGATTACTTTGATTTCATCTCATAACAATCAAACCAATTAA  CCAAATC  >Unigene19663_All Alpha-catenin [Parasteatoda tepidariorum]  AGTAACAATCAATTGAAAGACTTGGACGACAATCAGTTTATTGATGCATCTCGGATGGTTTATGATGGAGTGAGAGAGATCAGAAGAGCCGTTCTTATTGACCGATCTCCTGAAGATATTGATTCGGAAACTGACATGGATNNNCATGGATTATGAAGATAACATTTATGAAACTCGAAGTAAATCAAGTGTTATAACCGATTTTGATGAATTTCCCGATGTTGCTGAAATAAGTAACACCAGAGAAGCTTATAGAATGAT  >Unigene23433_All Similar to alpha Catenin CG17947-PA [Acyrthosiphon pisum]  GCCACATTATTGAGATCACCTTCAACTGCTTCCAAAGATTTGAGTAAAGATTTTACGTCAATTTTATCGGCAAGAATTATCAGCTCAGCGACACGATAGAGTAAATTTCTTGAATTTTGCACCATTTTATTTCTTTTAACAGAAGAAAATGGATCATCGGCAAATTCTCTAGAAGCCTCTGACATATTCTTACCCGCTTCACGAATCTTATCAATACAACCATTCATTTCTTCTTGAAATTCATCATTTTCACGGGCGATCTCTTCGGCAACGGTGATAAAATTTTCAGTTGCCTTTTCAATCGTAGCCACCAGAACTTGAGGTCTAGTTGATCGCCCACCTTTCTTTCTTTCCGATGTATTGACAAGTGTAGTTACCTGGAGAACCAAAGGAGCCAATGTTTCTTCGATCGATCGCGTTTCTATTCTCAAGGCCATATTTTAATTAAAAATGTAACAATTAACTAATTAAATAATAGAAAACAATAAATAATCAAACATTCAACTGACAAAAGAAAAA  >Unigene23453_All Similar to supernumerary limbs CG3412-PA [Apis mellifera]  GATCCATCCTCTTCGTCGAGCTAGGCCTCTCCATGAGGGATCTGTTTTAACTTTATTCTCTATCAATTTTCTCCATAACATTCCCTCTGATATAACACGATACCATTCGCGGCACACTAACTCAGCACAACGTAGAGAGTCAGCATCCAAATAGGATAAAATAGTCTCAGCAACATGATCAAGACCTTTCTTTGGTAGAAGAGAGATAAAGTCTCTTTTTAACATTGGTCTCAGGTAAGTGTTGATTTGACTATGTTGATGATGAGACATTTTGGCTAATAGAGATTGAATGAAATCTGTTTGTTCTGGGTCAGCCCAATTTTCGAAGAATTTACAACAATCTTCTTTCTGCTGGTGGAAAAATGGACGAACTTTCCCATCCTCACCCTTTTCTTCACTAATATCCGTCATCTTTTGAATCTTTCCCAAGTAAATCAATGTTCACCTTATTTATCCCTTTGATTACCTTAACTACGGAGGAAATTAAATACTACCAAATAATTAACAAAAAAACAGGACGATGATCAAAAAAGTT  >Unigene3502_All Similar to spectrin [Tribolium castaneum]  GTTTCATGAAATTGCGAAGGAAAAAAAAACTTAGCAAGAAACTAATTCTG  GAATAAAGTTTGTGTAAAATCAACGTAGTCATAGGCATTGGTTATTTCTC  GTCCTGATTTGGGGTCAACATAACGTTTCATACGACGTAAACAATAATCT  GCCATGTCTTTGGTGAGATTAGAATATAATTCCTCGGAGGTAACATAAGG  CTTCTCCTCGGCGATGGCCCGGACGGCATTTTCAAAGTCTTCACGATTAG  AAACATTTTCAGTTTCCCGACCAATCATAAAAGCCATGTACTCTTGTAGA  GTTACAAAACCATCACGATTGGGGTCAACTTGGTCCAATAAACCCTCGAA  TTCAGGATTGAACTCCCCTTCCTCGACCATTTTAAGATCATAACCCAAGG  CTCGTAAGCAGTTTTTGAAATCTTGGTGATTAAGTTTTCCACCCTTCTCC  TTGTCAAAGTGTTTAAACATCATACTGAACTCACGAAGCGATTCTTCAGA  GACACCAGATTGATTTCGGGCTTGAATTTGTTGCTCAAGATTGTGCTGCA  TTCTCATGCATAGTTGATCCAATTGATCCCATTGTTGGGCAAGTCCAACA  GTAGAGTGTTCGGTGTATCTATTGTCAAGAATAAGATGTTCCTCCAGAGT  AGCACCCAACCTTTCAATCTGCCTCAGGTCATCTCTCTTCAGTTTAATCT  CGGTCGCTTTACTTCGAGTTGCCTCGAGTTGAGCTTCCAAAGAGCCTGTT  CCCTCTCTCATCGCTGAGCCATCAAGTAGCCACATTCTTGTATCTGCGAG  CCATTGATGGAAACTGTTGGCTTGTTTAGCGAACTCTTTTCGTAGTCGAT  CATTTTCCTCTTGACGAAGTGATTCCTTGTGAAGTTCAGCATCACGATCG  TGAATAATTTTCTCCAAATTTCTCCAGGTATCTTCAAGAGCTTCCATGGT  GAACCAGGTATACGGATTAGAACCAACGTTGAAAGATTTAATTTGACTAT  CAAGTTCAGCCAATTGTTGGAAATCAGTCTGAGCTGAGGTTAGAGAAGCT  TGGAATTGTTGATGAGCCTCTCTTAAAGCTCGTATCTCTTCAATAGAATT  ACACCTAACAGGATCAGTTAAATCTTCTTCAGCATTCTCAAACCAAGAAT  TAAATGCAGAAGCTTTTTTAGCAAAGGTAAGGAATGAATCTTCAATCATC  TTAAACTGCTCCTGCATGTGAAGAAGTTTCTGTTTTCTTGCTTGTGAAGC  AGCCAAAAGTTCATTCCAACGGTTCATTACATCACCATGACGCTTCATAA  TAGCCTCATTTTGAATGTGATTATTGGCAATCAATTGGTCTTTCAATTGT  GTAATTGAAAGGATACCTTCTTGCTCAAATGCAACCAATCCAGTATCAAA  TGCTTCTTGTTTTGTCAATAAAGTTTGAACGGATGAAAGATCCCGACCAT  AGTCTTTGCTGCTCACTTGAACCTCTTTATCTGCAATCCAATTTTCAACA  ACATCAGCTTTCCACATGAATTCAAGGTAAGCTGCATTGTCATTCAATTT  ACCGGCTCTCTTACGAGCACTTTCTTGTAAATAATCAAGCCGAGCGTGAA  GAGCATTCAAACGTTGATCAATGGTATCTTTGTGATGATTACCTTCAACA  ATCAATTGTTGACCAGCGGAAATTATGTCAGCACATCTTTCCCGATGAAC  AGTGAAATCAGCTTCAAATGCATCATGTTTTTTGATTAAACCTTGAACAG  CGGCCATTGTATCACCATAATCCTCAACGGCGATCAGTTTTTGCTTCTCT  GAGATCCAAGCTTCTTCTTCTTCTACTTTGGCTAAAAATTGTTGGAACAC  AAGACTTTCTTCAAGCTTATTTCCTCTAACACCCGACAATGACTTCAAAT  CTTGCCAGTTATTTTCTAATGCTTGAAGTCTCGATTCAATCTCTTGAATT  CCCAGATTAGATTGATCCATGAGTTTTTGACCGGCATTTTGGACGGCTTG  TATGGAAGGTTCGTGAGTCGCAAGCTCAGCTTCAAAACGTTTGTGTTTCT  TGCGAAGATTAAGAACACCGGTTAAATCACGGCCGAAATCACCCGAGCTA  ATTAGTAACTTTTTATCCTTAATCCATGATTCCTCATCGGCGATATCTCG  GAAAAAGTGTTGTAAAGTATTGGCTTCATTCAGTCGTTGTCGACGATTAT  CGGTTAAAGTTTTAATTCTTTCATAACGTTCCTTGATCGATGAACGTTTC  TCTTGGATAACAGAAGGATCGAATTGTCCAGATTCTATTAAACTATCAGC  TAAATGGTCAAGATCTTTGATTCGATCATCTCGAGCAGAAATATCAACCT  CAAGAAGATCATGTTTCTTGATGAGATTTTCAACTGATGTTAAATCTTTA  CCAGAATCTTCAGTTTTCAATAAACCTTCAATCTCATCCAACCAAAAGTC  TAAATCCTTGACGGCAGCATTAAAGGTTCGCTGTTTATTGGCATCTTTTA  ATTTCAATGATTTTTCAGTTGTCTTTGTTGATAAATGTTGCCACTGCTCC  CTGATTGAGGTTAAACGATTTTGAACAGCATCTTCTGAGCCAGCACATTG  ATGCTGATCAATCAGTTTCTCACCCATACCAATGATGGAAGTGATTCTGT  CTTTGTTAGCATTAAGTTCAGCTTCAAATGCTTGATGTTTTTGATGTTTT  GATTGTATGTTTGCAGGATCTTTGTAACTTTGATCTTGTGCCATTTGAAG  TTTTTCCGAAATCCAATTTTCAACCTCATCAGCATCTCGTGAAAATTGTT  GTAAAGTCTGCGATTCACCAAGCTTAGAACGCTTCTCAATCAGAGCTTCT  TTAAGATTCTGCCATCTATCCAAAACTTGATCTTTCTTGTCCTTGATTGG  TTGCGATGCATAATGATCATGACCAATCAATTGGTCAGCAAGTGAAGTTA  ATTGTGCAATCTTTTCTTCTTGTGCACTTATCGCTTTATCAAAGTCTTCA  TGTTTTTTAATTAATACTTCAACATTATCTCCGCCCATATCATCCGAAGA  TAGGAAAGCCTCACGAGAGGCCATCCAGTTTTCGGCCTGTTCACAATCTC  GATAAAACAATTGAAGTTCCAAACATTGGTCAAGCTTAGCTCGCCGAGCA  AACCAAGCTCTTTCAAGATCATCTCTTGCCTGAGCGATCTCTCTCATCTT  CTCAGCGATTTCATCAGAAGCATAATGACCTCTGTCCAACAAATGGTGAC  CAAATATTTCAAATGTTTGGAAAGTTGAGGCTCGAGCATCAATTTCAGTT  CGATGTTCCTGATGTCTTTCAAGAAGAGCTTCAGCTCCGGTTACATCATT  TGCGAGCTCATCAGAAGAGACTAGAGTTTTCATTGAGACGATCCATGACA  TTAGATCACGATAATCAGCAAGGAAACGTTGAAGTTCATAGGAATCATCA  AGTTTCTCACGTCGAGCAGTTGCTTTAGCAATTAATTGATCCCATTCGCG  ATTTATGTCAATTTGCTTTTGGTATGTTTGATCAGCGGTTTCTGGATGTG  TTTTCATCAGTCTTTGGGCGATCTCGTCAAGAGATCTGATCTTATCGCCC  AAAGCAGCGAGATCTCGTTCCAAACCTTCGTGTTTTCTTTGTAACGTTTG  AACTGATCGTAAATCTTTACCGTAATCGTTATTGTTTAGAGCTTCATCTT  TTTCACGAATCCAATCTTTAGTTTCTTCGATATCTCGATGGAAACGTTGA  ACTTCATGGGCTGAACCAAGTTGTTCAGCTCGTTTGTCAGCTGCTTGTTG  TAGGTTAACCCATTTAGAGTTTAATTCGTCAATTTGTTGATTAATTCGGA  CAGCGGCTTCAGTTTGACCAAGATTAACAAGTTTGGTAGCAACTTCATTC  ATTTCTCGTAGACGAGCTTCGTTAGATTTCAATTCAGATTGGAAATCGTC  AAACTTTTTCTGATGAATTTCAACTTCTTCCAAATCATCACCAACAGTTT  GAACTTGTGCATGTTGCTCTTTATCGCGAATCCATTGGCCCAATTCAGCG  GCCTCTCGAACCACTTGATAAGCTTTACATGATTCATGAAGTCGAGTTGC  ACGTTCACCGCCAAGATCTAAAAGGTTAACATATTGCTTATCAATTTGAC  CTTGTCGAGCTGAAATTGAATTTTGTTCAGCAAGATGTTGTCGACTAGCT  GAAAGACCAGCTTCAATCTTTTTGACATAAGCCGCTGGGACGAACCCTTG  CCTGTCGTTGACTTCGACTTTCCACCAGTCTTTATTATTGGAATTAAGTA  AAGTGAGAACATCTCCTTTCTTCATTGAAACCTCACGAGGAGATTTCTCA  ACATAATCGTAAAGAGCCATGACAAACTCTTTACCAGTTTGATCAACAAT  CGGTGTTTCTTGTTGTTTACACGCAGCTGCCTGATCACGTAGACTCGAGA  TCGTGTTCCTAAAGGCCTCAAGATCGGCCATGAGAGCCTCGTGTTTCTTG  AGAAGAGCTTCGGTTGAATCTTCATCTTTACCGAAATCTTGCCCACCAAC  AATCGGCTCCTTTTCCTTCATCCATGATTCTGCTTCATTTGCATCCGCAA  AGTACTGATGAGCTTGAAGAGAATCATCTAAATCCTGCTTTCGTTTGTTT  GCTTTTTCCTTTAAACTTTGCCATTTATCGTTCAAAGAGAAGAGTCTTTT  TTGTATCTGTATCTCATCGGATGCAAAATGACAAAATGACCTTCGTTGAT  CATATCTTCACCATTCGAGCAAACAGTTCGGATACGATGTTCATGGTTAT  TTATCTCATTCATGACAGCTTGATGTTTCTTAATTAAATTTTGTACACCG  ATTAAGTCACGACCACGATTAGTTGATGCAGCAATTGGTTCTTTCTCTCT  CATCCAAGCCTCTTCATCTTCAACATCACGGAAAAGCTTTTGCAACCGAA  GAGCATCTTTCAATCGATTACGTCGGCTAATCATTGGCTTTTTAAGTGAC  TTGTATTTGTCGACGACATTGTTACGCTTTGATTTTATATTATCAGCATC  AAAGTGTCCATTAACGATAAATTCATCAGCCTGGGCAATGGCATCGTTGA  TTTTTTCTTGATGAGCAGCAACATCCGATTCAAGAAGAGCATGTTTCTTG  ATCAAATTTTGAACTGAGGTTAAATCTTTACCATAATCTTCAGACATTAA  TTGTCCTTCAACCTCATTTAGCCAAAGTTCAATATCTTCAACCCCATGAT  TGAATAGTTGCTGATCAGCTGCTTCAGTGAGTTTACTTCCTTTACGTTCA  GTTGCCTCAACTAAACTAGACCATAATTCACTGATCTCTTGAGCTCTTTC  ACTGATTCTGGGTGCGGCATAATGATTACTTTGGATGAGAGACTGTGCAC  TTGAGTTAAGTTCACTAATCCTGGGCTCATTGGCGGTCAATTCTTGCTTG  AATGTCTCATGTTTCTGAAGTTTACCAGTTAAATTAGTTGAATCTAAATA  ACTTTCATCTGATGCTGTCTTCAACTTTTCCATGACCCAGCCTTTAGTTT  CATCATAATCTCGTTCAAATTGTTGTAATTTATAGGAATCATTTAAAATC  GCTCTTCGAGCTTCAGATCTTTCAATGAGAGCGGCTCGTCTCTCAAGAAG  AGTTGCTCTTCTCTGTGCAACATCTTCAGCTGCATAATGTTGACCTTCGA  TCACTTTGGTAGCAAATTCATCTAATGCTTTGATTTTCTCTTCTTGAGCT  GCCAAACTTTTATCAAAATCTTCATGTTTCTTGATCAGCGATTCAACAGA  ATCTAATGAATCACCAAGATCTTCATTAGCCAAGAAAGCTTCTTGTTTAG  CCATCCAAGTGTCGGCTTGTTCAGTATCACGATAAAATAGCTGCAAATCC  ATACATTGTTCATATAAAATTCTTTTGTTCTCCCATAATTCAAGGAGTGA  ATTCTTTTCACTGGAAAGGCTCATTAGTTTCTCACGAACTTCATCAGTTG  CAATTCCTTGGTCGAGTAAAATTTTACCAGCATCGTCAGTTGCCTTAAAA  CTATCCATTCGAGCGTCGATTTCACCTTTATGCTCTTGGTGTCTTTCGAG  AAGAGCCTCAGCTCCAGCAACATCTTTAGCAAGTTCATCGGCATTGATGA  TTGATTTCATATCATTAATCCATGAAGTCAGGTCACGATAATCAGCCAAG  AATTGATGAAGAAGATGACTTTCATCGAGTCGTTGTTTTCTTCCTTGAGC  TTTAGATTTTAATTTATCCCAATTAGAAACAATTTCTGAATGTTTAGCTT  GAATTTTGTCTTTATGATCAGGATGAAGTTCACAAAGACGATAAGCTTCT  TGACCTAAGATGAGAACTTTGTCTTCCAAAGCAGCTAAATCTCTTTCAAT  TCCTTCATGTTTTCGTTGGAGAGCTTGAACACTAACAAGATCTTTACCAT  AATCATCTGAAGATATAACTAAATCTTTCTCAAGGATCCAGGAAATAGTT  TCATTAGCATCTCTGTTGAATATTTGAATCTCTTGGGCACCAGCGAGTTT  CTGTTGCCGAAGTAAAGTAAGTTGTCGCAATCGTTTCCATGCTTCTTCAA  CTTCGTTTCGACGGCGAACAATCACTTCATGATCGGGATGATTTTCTCCG  ATAAGCTTATCGGCTTGTTTGTGGACATCTTGAACTCTAAATTCTTGGCT  TGCCATCTCTTTCTGAAATTCATCGAATTGTCTTTGAAGTACTTCAACAT  GTTCCAAATCTTGACCCAATTCATCGCTGGTAATAAATGTTTCCTTGTCA  TTAATCCAGAACATAACCTCATCACATTGACGGTTAAATTGAACGAGTAA  AAGAGCTTGTTGAAGTTTGAGTCCTTTCTCAATCAATTTGGAAAGGAGAA  GCTCCCACAATCGGTGAAGTTCATCAAGACGATGGGTGATAACTTCAGAG  GCAAAATGTCCCTGGTTAATCATTTCCCCTCCACCAGCACTGAGAGAAAC  GATGGCATCTGAATGAGCAGTTACTTCCGCCTCGAAGGCTTGATGTTTCT  GTATTTTAGCCTGTAAATTGGTTGGATCTTTGTAAGATTCATCTGAAGCT  GTTTGTAGTTTCTCCAAGATCCAAGATTCAAGCTCATCGGCATCTCTTTT  AAAATATTGAAATTTCTTCGAATCTTCTAATAGACTTCTTTTTTCTTTAG  CTTCTTCCTTAAACCTTTGATATCTCCCTAGAACTTGTTCCCTTCTCTCT  TGAATATCTTCAACAGTTTCCAAGATTGAAATCTCTTTGGTGGTAACACT  GGATGTAGATGTTTCCATGGTGGAAACTTGCACAATAATCAATCACCTTC  CAATAGATAATTTAACCACAATTAACACAGTATTTAGTTAAATTCGATTG  CGATACTCAATTAATTCACTAATTTAAACACAACTCAGGGCGTAGTCGAG  TATATGTTGTG  >Unigene3895_All Similar to CG5629-PA, isoform A [Apis mellifera]  TGGAAATTAAATTAATTGATTTACTTCATCAACTAATTTAACAAACGTTT  TAATCATTTTTAATTCATTAAGATTGTTATTAACAAGTGTTTTTAATTAT  TTTAAAGAAAATGAGTTCCATTGATCAGGTTGACGGTAGTTCAATTGGAT  CCATGCAAAAGAAATCAACTAATTACCAAGACCAAGCTGACGATTTTTTC  TCAAATTTCTTGGAGCCAAATAATTTGAATCAAATCCGTTCTCAAGTTAA  ACAATTTATTGATTGTGATACCAATCATCCAACAGTTCTAATCACCTCTG  GAGGAACAACAGTTCCTATGGAGCATAATACTGTTCGATTTGTGGATAAT  TTCTCAGCGAGAGGATCAGCTTCAGCTGAATATTTTCTTGAAAAAGGTTA  TAAAGTTATTTTCCTTTATCGTTTAAAATCATTGGAACCATTTACAAGGC  ATATTAATGTTTGGCAATTATTGGAATCACTTTCCTCATCGGATTCTGGT  TCACAAGTTGGTCCATCTATTGGTGACAATTTATCCAAGACTGTGAAAAT  CTACAAAGAAAATAAAGCGAATATACTTATGATCACATTCACCACCCTTG  CTGAGTATCTTTATTACCTGAGGGAAATTTCATTCTCAATGCAGCCCTTG  GGGCCGAAAGCAGCTCTTTATCTTGCAGCAGCAGTTTCAGACTTTTATAT  TCCACCCACTGACATGGCAACTCATAAAATCCAATCTAAAAACGGACCAA  TTAGCCTTGTTTTTGAATTGGTTCCTAAAATGTTACGACCGTTGGTCAAG  TTCTGGGTCCCTTCTGCATTCGTTGTCTCCTTTAAGCTCGAGACTGATGA  ATCAATCCTTTTGGATAAAGCAAGAAAAGCTTTACTCTGTTATGGTCATC  ATTTGGTCATCGCAAACATATTACACACACGAAAAAGTCAAGTATTAATT  GTAACAGAAAAAGAATCTCATCAAATAACCTTACCTAATCCAGAGATGGA  AATTGAATCTCTAATTGTTGATGAAGTAAT  >Unigene14583_All TGF-alpha [Gryllus bimaculatus]  TGTTGGTGTTGATATTGATGTTGTTGAATCTTTTTTTTTCCTGGTGGTCA  AGTTAAATTTCAATTGTCATTTGTTATGTTGTTAGGGTGGGAAGTTGATG  TTGCTGGTGTCGCCGGTGAATTGTTGATGATGATGATCGTCTACTATTAA  AATGTGTCCAAGCAATATAAACATAGATAACTAGGATTACAGTTAAAGTG  AAAAAACCGGCCACATTGGCCATTCCAATGGTCACTTTGTCATTCCTTGA  GGTGGAAGCTGAAGGTGAGGGAAGGTAAGAACCATCAAGATCCTTGAATT  CACACCTTTGGCCCATATATCCATGGGCACATTCACAACTATACAGAATA  GATTCACCTACTCTAATTGAGAAACATGTTGCACCATTAAGACAATACCA  TTTTGCATATGCCTCGGGGCAGGCATATGTTTGATAGGTTATATTGGGTC  GGGTGGTTGAAGGGGTTAGTGAATTAAACGAGGTTGGAATCCTCGGTTTT  GGTGTTGATCGAGATGAACAAGATTCAGTGAATCGTAACAATAGTTGGAG  GAAAAAAAATGGTAACATTTTGCACATTTGATTTAATGTTTTTTAAGGTG  ATGATGAATTTTAGATGAGAACAGATAAGAAATTAAGAAAATGGTAATGA  TGATGATGATGATGAAGAATAAAT  >Unigene3044_All Similar to ENSANGP00000014048 [Strongylocentrotus purpuratus]  AAAAAAACAACGAAGAGAAAGAAACAAACAAGCTGCCGCTCGATGTCGAC  AACGACGAATCGAATACACCAATAAATTAGCTCGGGAGACGCAAGAACTT  GAAACGGTCAACAATGAACTTCGCTCTACATTAGCTGCTTTAGCCGCTCA  AAAAGTTCAACTGGAAAATTCACTTAAAATTCACAGTTCCATGGGTCTTC  AAATGATGTAACAATTGACAGTTCTATGCATCTTAATCAATTGGATTCTA  TTTTCTTAACAAACACAAACACAAACACAATAG  >Unigene3209_All RE62284p [Drosophila melanogaster]  CGTTGATCCAAGTAAACTTCGTCCACTTAAATTGCTTCCACTTCCAGAGT  TGTTGACATTTCCAGCGCCAACACCACCGCCGAGATTACTGAGACCCATA  CTTGATCCTCGTTCGTTATTTAATCCTGGGTCAGCATCGAAAGCCAATTT  CATCAATTCTGTTAATCTTTCTCTCGCTTCACTTTCAAGAATCAAACTTG  TTGATGGTTTTGATGTCATTCTCTCTTCATGTAAATTGATTAATGAACTT  TGAAGGATATACAGTTGATGAGCCATTTCAGATCCAACTACTGATCTTCC  CAATGTTGTTGTTGTTGTTTTTGATTTTCCATTAGACATTAATGATGATG  TCGTTGATGCGACACTTGCAGATATTAATCCATTTCCTGTTTCTCCTCCT  CCACCATTAGAACCATTCAAAGT  >Unigene4144_All Epidermal growth factor receptor [Gryllus bimaculatus]  AAATTGTGCTAATTATTTATTCTATTCTGTTGATTACGCACAGATTAATA  TGCTGATCTACCTATGGATAAAAACTTTTGTGTTCACTTGATGGTTGATA  CTTTATATCGACTAATGGATATTGGGTGATCGCTTTCTCCTTAAGTGCTT  TGTATGTGCCTTTGCCTGTTGTGCCTTCATATTTGCTTCCATTTTTGTGA  TAGCTTAATATCTCATCGTCTTTATCATCCCATTTATCACCAAGTTGTCT  GTCTCTCTGTCTCTCTCTCTCTTTTTAATATTTGTCTAATTTAACAGGCA  AATATGATGACAATATCAAGGAAGATACCTAATTGTTGTACATTATTTTC  TGTGTATATTTTTGTAATTATCTTTGTAACTACATTTGATTCCATTTTGA  GTCAATCTCTTCTATCATTATCTTCTTCTTCTTCTTCGTCATCCTCACCT  CATCTTATTCTTCTTATGATCCAACCTCAACATCTTCCACCAAAAAGGGT  AAAATATGTATTGGTACAAGTGGACGAATGTCAGTTCCTTCAAATCGTGA  ACATCATTACCAAAATTTAAGAGATCGGTATACCAATTGTACTTATGTCG  ATGGTAACCTGGAATTAACTTGGTTGGAAGATGAAAATTTCGACCTTAGC  TTTTTAAACGAAATACGAGAGGTGACGGGATACGTTTTGATCAGTCAAGT  TCATGTTAAAAAGGTCTCACTGCCCAATTTACAAATCATTCGAGGACGAA  CTCATTTCAAATTAAATATTCGAGATGAAGAGTTTGCTTTATTGGTTACT  CTTTGTAAGATGGAAAATCTTGAATTATCTTCATTGCGAGATATTCTTGC  AGGAGATGTTGGTTTCTTTGACAATTATAACCTTTGTCACATAAAGACAA  TCGATTGGGAAGAAATTCTATCTGAACCAAAAGCGACCACCGTTTACTCT  TACAATTTTACCCACCCTGAAAGAGTATGTCCACCATGTCATCCCCTTTG  TCCAAGAGGTTGTTGGGGTGAAGGTATTGAAAATTGTCAAAAATTTAGCA  AAATCAATTGTTCACCTCAATGTCATCAGGGTCGCTGTTTTGGTCCCAAT  CCACGGGAATGTTGTCACCTTTTCTGTGCTGGTGGTTGCACCGGACCAAA  GCAAAGTGATTGCTTAGCATGTAAAAATTTCTACGACAATGGAGTCTGTA  AACAGGAATGTCCACCCATGATGCGCTATAATCCAACCACCTATTCTTGG  GAAACCGATCCGGAAGGTAAATACGCTTATGGTGCAACATGTGTCAAAGA  CTGTCCTGAACATCTACTCAAAGACAATGGAGCTTGTGTCCGTTCCTGTC  CAGCCAACAAGAAAGCTCAATCAGGTGAATGTGTCCCCTGTGACGGACCT  TGTCCCAAGATTTGTAAAGGTGTTGATGTGATGCTTCATTCTGGTAATAT  TGACGATTTCCGTGGATGCACCCAAATTGATGGTTCTCTGATTATTTTGG  AAAGTTCATTCATCGGATATCAAGAAATTTTTCCCAATCTTACCTTTGGT  AAAAATATTCCTCCCATGCATCCGGATAGATTAAATATTTTCCATACTCT  CAAGGAAGTTACCGGATTCATTTCCATTCAAGCAAATCATCCAGCATTCA  CTAACCTCTCATACTTCAAGAATCTTGAAACGATTCAGGGTCGAATCACT  TTCGATATGTTTGCCGCTTTACATATAATTAAAACTTCTCTGGTCTCACT  TAATCTACGATCGTTGAAGAAGATAAGTTTTGGCTCTGTTGTCATATCGG  AGAACAAAGATCTTTGCTTTGCAAACAGCATTGATTGGAAGAAACTTATT  AGTTCAGATGCCCAAAAATTCCTGGACGGAAACGGAAAAGATGAAACTTG  CCTTGCCAGAAATTTAGTTTGTGACCCTCAATGTGGTAACGATGGATGTT  GGGGCAAAGGTCCTGATGAATGTTTATCTTGCCGATCTTATCGTTTACTT  GATACTTGTATTGATGGATGCAACTCATCACTTGGTTATTACAATGATGG  TGGTCACATATGTAAACATTGTCACCCTGAGTGTTTAGGTAAATGTTTTG  GTCCTGGAGCTAATAATTGTACTAAATGTCGAAATGTAAACGATGGACCT  TATTGTGTGAACCAATGTCCAATCTCAAAGTACAATGATTCCGGTGAATG  TAAACCTTGCCATGAAAATTGTGTATCAGGATGTACTGGACCTGCGAATC  GACCTGCTTATGGAGGATGTAACTCATGTGAACGAGCGCTTGTTTCAATT  GAAGATCCAAATGTAGTTGAAAAATGTCTCAAAGCAGATGAACCTTGTCC  TGAAGGATTTTACCATGAATACATTGGCTCCAAAGAGGATGGAGCTCTTA  AGCCTTTGACTGGAAAGTCCGTTTGTCGTAAATGTCACCAACGGTGCAAA  AACTGTACAGCTTATGGCATTCACAAGTCTGTTTGTGAATGTTTAAGTTA  CTCTTCCGCTGAACAATGTGAAGATACTTGTCCTCGAGATCATTGGGCTG  ATGAACCGAATCATGCTTGCTATAAATGTGCCGATGAATGTCAAGGCTGC  CATGGACCAACTAACGGCCATTGTGTAGCGTGTCGTAACTATCGAGTCTA  TTGGAATGATGATTCATCTTCATTCAATTGTACAGCCACATGTCCAGCCG  ATAAACCATATAAAGTAGTCGCCGTTAACGTTGCCGAAGATCCATATTGC  TCGGAGAAAGAAGCTGAACAGCTACTTCCCGTACCAAAAGATTCAAATAC  TTTCCTAATTTTTGGAGTCATCGCATTGACCGTTTGTTGTCTCGGCTTTT  TCCTCGCATTTTTCAGCTACCAAGGCTTACAAAAAGCACGAACTAAGGAA  AAGACTATGCAACTAACTATGAGAATGTCCGGCTTTGAAGATAATGAGCC  ACTTAAACTGACCAATGTCCGACCCAATTTGGCTAAGCTACGCATCGTTA  AGGAAGCCGAGCTTCGTAAGGGTAACGTACTTGGTTGTGGTGCTTTTGGT  ATGGTTCACAAAGGAGTCTGGGTTCCAGAAAATGAGAATGTCAAGATTCC  GGTTGCTATTAAAATCCTTCGGGAAGGAACAGCTCCGGATACAAATAAAG  AGTTTCTCGAAGAGGCTTATATTATGGCTTCTGTTGATCATCCCAATTTA  CTTAAATTACTCGCCGTCTGTATGACCTCGCAACTTATGCTAGTAACCCA  ATTAATGCCTTTAGGGTGTCTCCTTGATTATGTTAAACAGCACAAGGACA  AAATTGGTTCCAAACCTTTACTCAATTGGTGTACCCAAATAGCAAGAGGA  ATGGCCTACCTTGAGGAGAAACGAATGGTCCATCGTGATTTAGCCTTGAG  AAACGTTTTACTTCAAACACCAGGGTGTGTTAAGATTACCGACTTTGGCT  TGGCTAAATTGTTGGACATTAACAAAGAGGAATATATTGCCGAAGGAGGA  AAAATGCCAATAAAGTGGTTAGCCCTTGAATGTATTCACCATCGCATCTT  TACACACAAAAGCGATGTCTGGGCGTTTGGTATAACCGTTTGGGAATTAT  TAACTTACGGTGGACGCCCATATGAGGGTGTTAACGCCCGAGATGTACCT  TCAATTTTGGATAAAGGTGAACGTCTCCCTCAGCCCGCTATTTGTACGAT  TGATGTTTACATGATTATGATTAAATGTTGGATGTTGGATGCTGAGTCTC  GACCATCGTTCAAAGAGTTGGCCATGGAATTCGCAAAAATGGC  >Unigene14021_All Similar to ENSANGP00000022750 [Nasonia vitripennis]  TGCAGATAATTGTTTTTGGTGAGTTTCTCGGTGTTTCTCTAGTTGCTCTT  CTAGTGCAGTCTTCAAGTTTTCTGCTGAATCTTTTTCCTTTTCCTTTTCT  TCGTGAGCCATTTTGTGCATCTCATCAGCAGCTCGCAGTTTAGCGCACTC  TTCTTCTAATTGATCGACAATTTCTTCCAATGAACGTTTCTTATTTTCAA  TGTCTTTCATTGAATCTTGTAATGAGCTCATTTTGGCCTCATATTGACTA  ATTAACAAACGACATTCGGTCAATTCCTTTTCGTTTAGCTCTATCTTCTT  GTTACATTCGTTTTGGAAACTTTCCATTTGATTACATTTAGATGCTAACG  CTTTTACTTCCGATTTCATTTTGGTTATGAAAAGACGAGCAACTGTAAAT  TCGTCTTCGATCTTTGCAGAAGTCAGATTATCTCCGGCATCCTTTAGACT  AGGAGCTGTTGCACTGGTACCTAACACAACACCAATTTCATATAAATCTT  TCAACAGGTTACTCAACATTTCATTGATCCTTCTTCTCATGTGGGTGTTG  TTTTCCTTCATTTGTTGGAGTTCAGATTTGGCAGTATTTAAGGCCATCAG  TTTAGTATTCAAATCTTCATTCAATGTTTCGAATTCACGATTCTTGTTTT  CCACTTCTTGACTTTTCTGATCATAATTGACTGCTAATTCCTCTAAAGCT  TGTAACACTTCTTTCACTTCTTCTTTTGATGATTCATTCTCTTGTTGAAG  TTTGTTAATTTCATGTTGGAGTGATTCATTATCCCGTCTACAGTTGCCGA  TTAGCTCTTCTTGTTCGAGAATTTGTTCTTTTAACTTCTCGATCATTTGG  CTTTGCTTATCAAGTTCGTCATCTTTTTCGTCAAGTTGAGCATAAAGTCG  AGTTCTTTCCTCTTCGAATTTCATTCTTTCTTCATTGGAAATGGGCTCAA  CTGCGGCTGAAAAGGCTCCGGTCAACGGGGTATTAGCAACAGAACTGAGG  TTTTGTACTGACTCAGATAAACTGAGAGTACTTGATTCCAAATCTTTGAA  ACTCGCCTGTTCGTCTGAAGGCACAGCGTCTCCGATGCGCCAACGATTCA  ATTCCATCTCGACACGGTTTACATAGTTTTTAAGCTTAGTTACTTTTTCT  CTTTCTTTTTCCCATCGTCTTTTCCATTCTTCTGCGGTCAGTTCTTCATT  TACAACAACAACATTAGTAATTGTTTTAGCTCTTTTACCAAATTCGAGTG  TTGATTTGGTTTCCATCTCATTAAATGAGGCTGGAGAACAGCAAATTATA  ATGGTAGTTCTAGAGTTTCCTCCTAAAGATTCTTGGAGTATCCTGGTCAG  TTTACTATCTCGGTACGGAATATGTGATTTATTGCCATCAGCTAAGGCGG  AAATAACATTACCAAGAGCCGACAATGACTTGTTTATATTTTTCGCTTCA  TCCAAAACCATTCCCTCAGCTCCAGTTTTACTAACTTTCTCTGAACCAGC  TAAGTCAACCAGATAGAGTTTACCAGTTAATTTCTTTTGATTTTCCTGGT  TTTCTTGTTTAACATTGATTAAAAAGACAGAATGGCTTCTACTAGAATGT  TCATTCATATTTGTAACAGCGATGTGTCGATTTGATTTTCCTTCGTCAAT  TACTTCCATAACTTCCTCAGGACAAGTGACAAACCTCTCGGTGGCTCCTT  TAACGAATGGGACACGATTTTTATCTTCATGCACTGACAAATTGGTCTTA  GAAACATCTAAGAGATCTCTTATCTTATCAAGATAGATTTCAAAATATGA  AACCTTAATGTGGAACTCAATGTTTTCGTCCATTGAATAGATGTGGTTGA  AAATGTCATCGACAATTCTTGGAATAATTCCTTGGAGTTTAGGATCTCCG  AGAACACCTTCCATGGTATGAGTTTTACCAGAAGATGTTTGCCCATAGGC  AAATATTGTTCCATTATAACCCATGAGAACATCTTTGACAATAGTTTTAG  CAGCTTCTTCATAAACTGTATCTTGTGTAGCGTTTGGTTTGAACACTTTA  TCGAAGACGTAATTTTTACCCCCGATTGAAACACAACCTTCATTATGAGC  TGGATATTTAACAATAAATGCAGAACCAGCTTTCACTTCATTCTCGTTGA  GTGGCCTGAACCTACAAACCACTTTAATGTTACAAACAGCATCGTTTATG  TCCCCAGACATAGTTTAACTAATAAAAGACAATATATTCAATAACAATAT  TCTCATTAATTAAATGTAAAAATAGAATTAAAAAGATCACAAGC  >Unigene23680_All Actin related protein 3 [Suberites domuncula]  TTGATTGTTTCTAGTTTTGTTTTTCACCGTTGATCCGAATAACCCAACAATTAAGATTCGCAGATTTAATTGATTGGCGAGGCAATTTTGCTTGTAATCAACTAATTATAGTGTTTTCCAAGGTGTGAGAGCAAAAGATGAGAGAATGATAGAGAAAATTAGAAGAGCAAACGAAATTTGATGCGAATTAAATCATCAGGTAATTATTGAAATTTCTTTTTTCACGAGAGTTAATAAATTGTTCATTTAAATGAGAGCACCGAAAACTGGATTGTGTCGACAAATACTAGGACCATATTCTTCGTATTTTTGTTTCGTGTGCGAAGTTGTATAAAATTCATTCGTCGAAGCTAAAATCGATCCACCGAACCAAACAGCGTAACGTTGCATCGAATGGGAAACAACTTTGACCGTCATTTGACTTGGAGTGATTGTTCCTGATGATAATTGTGTACTTAATTTCTGTCTAGCATTGCATTGCCGTTTAATATCACGTTCTAAACGTTTATCGAAATCTTTGAACATAGTTGATCCACCAGAAAGAACGATATTCTTATAAAGAGGTCGACGAACATCAATTGGACAGTTTTGTATACAAGTGTCAACAATGTCAGAAATTGATGTTGAAAAATCAGGATTAGCGATCTCAGGATTGAAAAATATCTCAGGTCCAAGGAAACGTTCAAATCCAACATCAACATCAAAATTTGATTTGGTTATATGATTAACACCAGAATATTTTTTAACACATTTAGATGGGTTCTTGTCAAATTTGTCAAACTCTTTAGCAATATCTGGACAAATGTAGCAGAATTTTTCCTTAATTGCTTTTGCAGTTTCCAATGATTGTTCGGGTGGAATGTTAAATTCACGTTCACGTAGAAGACTTTGGATAAAATAAGTAATATCACGACCTGCAATGGGAATATGTTTGATACAACTACCGATAACATAGCCATCGGCAACAGGGATAACATGGGTCACACCATCACCCGAATCAACCACAATTCCGGTTAAACTTTGAACTCCATTGGTTGCCGACCAAGAGGCCACCAGGGCGAGAACAGCTTGAACGGCAATGTATAAACCAGGTACATTAAAAGATTCAAACATAATCTCAGCAGTGTATTCACGGTTCTCTGGTGTATTCAAGGGCGGTTCCGTTAGTAGGAAATAATGATCCTCTGGTTCAGCTCGAAGATATTTGAAAATACATTGTTCCCAGAAGCGTTCCATCAAGTCCCAATCTTCAACGATACCATGACGGATTGGGTATTTAATTGAATAACCATTTAGATCAATACAATCATCGCCAATGAAAAAATCTAAATCATCAATTCCTCTTGCTAACCGACGAGAAGCTTGATCACCAACTTTAGATGTTTCCTTTATTGCTATGGACGATGGGATAATATACTGAGGTCCAGTATTACCAGCATAGCCTAATTTAGTGTAACCAGTACCATTGTCGATAACAATTGCAGGTAAATGATTAGTCATGATGATCACCAACTTGACACAGAAAATGAATCCAACACTGTGACAAACAAAAAACTATTCAAAGAGAAGATTAAATGGAAAGAGAGAGAAAAGGAGAAAGAGAGAAAACTTTTAGAGTATTGCCTTAACCACC  >Unigene13932_All Similar to AGAP010206-PA [Tribolium castaneum]  AACATCTTCGGGATGTTTTTCTATGTGTAAATAATGACCTGGTGATAAAA  GAAAACGAAGGAGAAATTATCTCTCTTTTCCTTGTGAAAAGGTAATAAAT  TATTTCTGGTTTTACCATGAGTGAGGTGGAAAAATTATCTTCTGAAAGGG  ATGCCCTTAAAAAGGAGATTGAAAGGCTTCAAGAGGAACTCACCCTTTCA  GCTAAAGAGAAACACCAATCTGCTGAGTTGGGCCTTCATTTGTTGGATGA  AAAAGATAAACTTCAAGTGCGATACGATGAACTGGAATCTTTACACGAAG  CAACTCGAACAGAATTGGAAGCCTTGCGAAATGCTTTCGCTTCATTTCAG  ACAAGTCAAAAGGTATCAGCGGACAGAGGGATCGAGCAAGAGGAGAGTTT  ACTTCAAGAATCTGCATCCAAGGAGGCCAAGTTTGCGACCGCTTTACATG  AGCTTGAAAGAGAATTGAAATTGGTTCGTTCGGAATTGGCTCGAGTGGAA  GCAGAAAAGGAAAGGTTACTCTCTGAGCATAGCGATCTATCCAAGCAGCT  GGAAATCTCAGAATGGGAGAGGAAAAACATGAGAATAGAGATAAAAGAAC  TGAAAATGAGAGAATCAAGATTATCTAGTGACATGAATGAATTGGAAGAT  GAGAATATAAGTTTACAAAAGACCGTTTCTAATCTTAAATCGAATCAAAT  CGATTATGAGACTGCTAAACACGAAGTTCGACGTTTACATGAAGAGATTG  AACTAAGGCGACTTCAAGTTGAAGAATTTGAAACTTTGAAAAATATCGCC  GAAAAGCAGATGAAAGAAGCATTGGAAGCTTTAGAATCGGAGAGAGAACA  AAAATACGAGCTAAAGAAAAAACTAGACGAACGATTAACCAGTGATCCAA  TGTTAAACATGACCAATTTTGGCCTTCGATTTCCAGGGTTAGCTGTTTTC  GAGAGTCAAGTGTCAGCTGATGCATCGGCCGGCGAGGTACTTGACGAGGA  AGGATCTTCACCTTTACTCCACCAATTGGAATCTGATATGTTAAATGCAA  GGAATGCATGTGAAGGATCTGAACACGATTCTTATGGCCATGATGGATCA  ACCTCACAACCTTCATCTCTTTTCGGTGAAGTTCATCTAACCGAAATCAA  GAAGCTAGAAAAAGGTTTAGAATTGGCTGAGAACGAGAAAAATCAACTTA  CCCTTAAACTCAGTGAGACTCACAATCTGTTGGAATCAACGAAAGCTGAA  TTATTGGCTCAACAGTCACGACTTTCCAAGTTAACCAATCATATCAGCAA  TATAATTAACAGTGAAGGCAATAATGAGATGGTTAATTCATTTGAAACTG  AATTCCCTGAATGTAAACAATTTATTGGTCAATTGTGTGATAGAATAAGA  AAATCAAAAGAAAGTCCCGAAGGAGAAGATGAACTCAATGAAATTAAACG  ACAATTAAAAGAATGTGAACTCATTCGTGATAGTCTTACATCGGATCTTG  ACCTTTTAAACAATTTTGCCGATGAAACAACTTCCGGTTCCATCGCAACC  GTTGAAGATCTTAACCGGATATCGGAAAAATTGGCCACCCTTTATTACCA  TATTTGTTCCGTTAGCGGTGAAGAACCATATCGAATTATTTTAGATCATG  CAAATAAATCGGAAGCACCTTTAAGCCTACGAACGGAAGGACTTAAAGAT  CGTTTAACTGATGAACGAAGTCGTAAACTTTTAGCCAATTGGAAATCTGG  AATTCTAGCCACCTCCGAGTGTCGTAAGACAATGGATACCATTGACGATC  AGATGAGACACTTGAACAATGCAGTTGAATTGATTATCGAAATGAAAACA  CCCGCTCGAACCAAAATTACCACCGAAAATAATAATACATCATCCGCCTC  TCAACAACATTCCCTTTCCACCAATTCTTCCGGAATTGCTTCATCACCTT  CACCCTATTCGGTTGGTGATGC  >Unigene13980_All AGAP011399-PA [Anopheles gambiae str. PEST]  TCTGACTTTGTGTCTTCTTCTTTTTTTTTAAATTAAGAATATGAGTCAAA  CGGAAACCATGGAAAAAGAGTTTTCTGAGACGGAATTGGCGGGCGGTGAA  AGTTTGATTGAAGATGAGGATTTGTCAGACGAAGAGTATGTTGAGGCTCT  TGGTGAACACGATGAAGATGGTAATGAAATTGTCTCAAAGAAAGTTAAAT  CACCTGTTTCATCACCCATTCATCAGCAGAAGAAAGATTCTGGTAAAGAT  GTTAAAACTGTGAAGAAAAGTGAAAAACCAGCAGACGAGAAACATGATGT  TGTTGCCGTAGCTTCTTCTTCTTGTATTGTTGATACTGATGTTAATGCTA  CTGATAAAGAAAAAGAGACAAGCGAAAAAGATGAAGCCATTTTAAAAGCC  AAAGATGATAAGGATACTAATGATGATATTGATCAAAATGATGATAATGT  GATGGATAAGACTCAAGTCAATTTGGGTGATGATGATGATGAGGATGATA  ATGAAGAAGAAGTAGAAGGTGGAGAAATTGAAAAAGAGGAAAACGAAGAT  GAGGACGAAGAACATGAAGATGAAGAAAATGAAGATGGAGATGATGGTGA  ATCTATCGAAGATGAAAATGAAGCTGAAGATGATCAGGAAAATTCTGATC  TTTCCAAACAAGCTAATAAAGAATTGGATGATGATGAAGACAGAAGAAAT  CCTCAATATATACCAAAGAGAGGTGCTTTCTATGAACATGATGATCGTTT  AGGAACTGATGATGAAGAAGAAGACGAAGAAAAAGAACCAGAAAAGGCTG  AAGTTTCAGTTGAAAAGAAACCGGAAAATCCTGTGGTTGAAATAAGAAAA  GCTAAAAAGCTTTGGTCCGATGATGCTAAATGGGGTCATGATATGTTTGA  TGATGATGAACAGAGGCCCAAACCTAGAGACGAAATTGTTTCTACCTATG  GTTATGATATAAGAAGTGAAGATGGTCCACCCAAAGCAAGAAGACGCCGT  CGATATGGTCGAGGTCCAAATAGATACACTCGAAGTTGGAAAGATGAAGG  TGCTTATTCCAAACCTAATGAGGCCCCAGCTCGAGAAAAGGCCAAACCTG  AAGCTAATGATAACCCGAAAAGGGAGAAAGAGCCAAATAGACCTTTTATC  ACAAAAGATGCCAGTGAAAAGCCCATCGATAATAATAGAAGTGGTGCTAA  AGGTCAACAACAACCACTAGCACAACCACAACAACAACAACGACGTACTG  AATCTAAATCAATAGCAATGGAAGGAAGTGATCGTAAAAGACCACAACGT  GAAAGAGACACAAGAGATTCGCGAGACACAAGAGATTCACGAGATACAAG  AGATTTACGTGGTCAACAACAACAACGTGATAATGATCGTAATCGAGATC  GTAGGAGAAATAGTTCCAATAATAATATGAGCCCCGGGCCCTCTAAAGAT  CTAAGAGAGTTTATTAATAACAAAAATGAAAGATTAGCTGGAGTTGGAGG  TGGAGATGTTAGAAGAAATCAAAAAAATTTCTTTGAAAATGATTTTCCTG  AACTTCCACAAAACAGTAAAGATAAAATGATGATGAGACAACAGCATCAT  AATCAACAATTACATCATCAGCAACAATCAAGTGCAACGGGAGACACAAA  TCAACCCAATGCTTGGAGTAAATCACGACCAAAGCATTCAGATGCTAGAG  AAACTATTCGAGGAGTTGAAAGCTCATTAGATTACAAAACGGAGCCCTTA  AGGGTACCCGAATCTTTTGACAATAAACACCGATCATTTAATCTTTCCGA  GACAAATCCCAAGATCGAGATCGTTAAAACTCAAACTTTTGAAAACTCAC  GTTACTTTAATAGGCGACCATTAACCAAAGGAAGCGGAAGTCATCATCCA  GAGCCATCGAATAATATTAAAAAAGATCTGTCAATCCATAGGAATAATAG  AGGACGAACAAGACCAACCGGTGAACGGGATTTTAGAAGTGAAGTTGGTC  GACCGATAACTAAAACAAGTCATTTGACTGATGATTATGAAGATGGTGGT  AATAGTAGTGGAAATAATATGGAACAGCAACAAAGAAGAAATCGTTACAA  TGTTAATAAAGAGAGAGAATATCGAGGTAATCATCATGGTCCAGATGATC  GTGATCGACGTGGAGATTCAGATCAGAGAGGAGGGCGAAGTTATCTTGAC  GATAGACATTATGAGAGTCAATTTAATGATAATCAAAATGACATCTCAGG  TGGTCTGAATAAATTAACACTAATTGACAAAGGTAACAATCGATTATCAT  CTTCAGAGGTAAATGATTCAAATAATTTATCCAAACCTAAACGATACTCA  TCATTACGCCAACATCAAGCTCAACAGCAACAGTCAGAAC  >Unigene17446_All Similar to atypical protein kinase C CG10261-PA isoform 1 [Apis mellifera]  ACATTATCCAATTTAAGATCTCGATAGATGATGAACTTTTCATGCAGATAATTTAGTGCCAATGAAATTTCGGCAGCGTAAAATCTTGCGTGATCTTCGGGTAATCTACGTTTACGTTGCATATGGAACATTAGATCACCTCCGCTAACAAATTCAATGACAAAAAATAATTTCGAAAGTGTTTGAAAGCAAGAATGAAGTCCGACCAGAAAATCACATCCAGTGGCTATTTCGAAAATGTGTTTCTC  >Unigene3333_All Similar to CG10261-PE [Nasonia vitripennis]  ACTCGGATCGAAATGTTTCAAGGGATCGTTTTCCATTTGAGGTACATAAG  GTGGTTGAATCTTTTTCTCGGCCAAAAGTTCCCAGTCCAAACACTTAAAG  AACGGGTGATTTTTAATATCGAGGAATCCGTTGCCACCCAGGGCACAACC  TAATCGGGTCTCTGGCTTTTTATCGAGAAAACCTCTTAGGGCGTTTTGTG  CTTTCACGGAAATTGACCTGGGAATAAGAATCTTCGTGGTAATTATAGCT  TGGAATAGAGCGTCTTCTTCCTCTTGTGTTACAACGAAAGGAGATTTACC  GATTAACATCTCGAATAGTAGAACACCGAACGCCCACCAATCAATACTGA  ATGAATACTCGCAACCTTTAATTATCTCCGGAGCGATATAATTAGGTGTA  CCACAGAATGTGGCGGTTGTTTCACCAGGTCGAATACCCTCTTTGCA  >Unigene6877_All Similar to atypical protein kinase C [Tribolium castaneum]  CCAGTGGCTATTTCGAAAATGTGTTTCTCCGCTTGAACCCAGTCCATATC  TTCATCAGCAGCAACGATCTCCTTCTTGATTACTTTCATTGCGTATCGTC  TTTTGGTCTCTCTCAATTCAACTAATAAAACTTTGGCATATGAACCTCGA  CCAATAACTCGTAGAAGATCAAAATCATCTAAACAATAATGACGAGCTCC  ATTTTCAGCGACCACCAGTTCATCTGATCGCTGTTGATTGTCTTCGCCTT  CAGCTAATGAAGCTTCATTGTCATCTCTAGAATCTCGTGTCCGAAAAATA  CTTTTCTTTTCGTACCGTCCAGTTCCTTTTCTACTGGTAAAAGATCGCTT  AGGCTCCGGTGTAACAGATTGGCTGGAAGGTCCATTGTTGGTAGGAGACT  GACCCGT  >Unigene11299_All GM21966 [Drosophila sechellia]  CCACAGCGACAGAAACTTCTTCCTCTAGCGTTCCAGTGACTGAAGATAAA  ACTTTGAGCTCATTAACCACAACTACAGCTGTAGCAATCTCCTCCTCAAT  CACTACTAATACTCAGACGACTACAATAGCATCAACAGGCTCGTCGACAA  GTTGTCATTCAACCTCGTCAAGTTTACGTCCAAAAGGACACACGAAATCA  GCTTCACTGTCCTCGTCTACTGGACGTTCTTCAAGCAGTTCTGAAGCCCG  CTCAGAATCTAATTCTGAAACAAATCGACCGAGCTCATCAACGGTGAAAG  GAACCTCATCAACCCCCAAAACCGATAGACTGGTTGTTAATCGAGCCTTT  CCTCGAGGGACCGCAAACCGTAGTACCTTTCACAGTGGTCAAACAAGAGA  AAGAAGGTTCGATCGAATGACATTCGATGGATCAGCTGGAGCACCTCTGC  AAACACAGGACACTCTATCTGTTAGCCAATCCAGGCAATCATTTTTCAGC  AAATTATCATCCAAATTTTCTAAAAGG  >Unigene11395_All GF14047 [Drosophila ananassae]  TTTTTTGGTGAATGTCCAAAGTGGATTCCTTGAGTAGTCGATATCTGATA  TTCTGTAAGTTTTATTATTGAAATGAGTAATTACGATGGTTCCGGCAAGC  TCTTTCTGTGCATCTATCTGCCATCGGGGTGATGCACCGTCTCTGAAACG  CATAATCGTTTCTCTGAGTGTCTCTTTACGAATAAATTTAAAAATGGTAT  CAGTTACTGTTAGAATACCGCCATCGTGAACGTTTACCGCGGTTAGAAAA  CCACGGAAAATAGAGACACCACCATGTTCTTCTAGATCACGACAAGACTG  TTTATCGTAAAAGTAACGGCCGATTAAATGGAGACCAATATGAGCCAGAT  TACGACGCATTTGTGTATTGTACAAGCGTAACATTTCAAATGCACCATAA  CCAATGTCACCCGTTTGACGAAGTGTCACATTTATCATTTCACCGGTAGT  GTTTAAAACTGGATAAGTAATTGGACTTTCAATATCAACACACGATTTAA  GGTTAGACATCCCATCGAAAATGTAAGCATGGCCAAAATGTTCGGCAAGG  GTATTGATTAAACGTCGACGGACGAAAATTGCTTCAACGTTTGGCTCGAA  GTCAACACGATAGTCGAAAACACGTTCATCATCAGGAACTTTTATACGAA  AATAATTTGATAAAAGAGGGATATCACCTCCGCCCGTGCCAATTGTGGTC  GTTATGTTGGGAGGTTTGGTTATCAGTTGTGGCGATCTTAGTCCACCACG  ACCTCGAAGTGCTTCACCATTACCACCACCACTTCCGTTTTCACTGGAAC  TTGAGCTTTCACCCGCCATGGCTTCTTCTTCGGTCATGAAGTGTTGACCG  GGAGCAAGAACTCGAGGTCGAGCTCGGGTACCAGGACCTCGAGCACGTCC  AGTCATGGTTGACTTTCGGGTTCAATCGTATTCAATATAAAATCTATTAG  AATTTATCGAACAATTAAAATGAAAAGAAAAGGAGATTAAACAAATTAAA  CAATCTCTTGATTAGTAAGATCAAAATACAATTAGCAGGGGAAAAAATGA  ACGAAATCAGAAAACAAATTTTTTTTTACTAATTTGAACTACACTCA  >Unigene3437_All Similar to spire CG10076-PA, isoform A, partial [Apis mellifera]  ATGAGGATGAGGATGAGGATAAACTTGTGGGCTCAGTATTGATTGATGAT  TGTTTCTCATCATGATGATGACCATCGGTGACCTTAATCTTGGCAATAAC  TGTTGTTGTCGATGAAATCGATGAAGATGAAGATGAATTTAATTGTGTTG  TTTTAATGATTTTACGTTGATTGTTATTTGGCACCAACAATTGAATTGGA  ATTGAATTGTAACGTTTGGCATTAATTTGTACATTTGATGAGCATCGAGA  GCAGACTGGCCTTTCACATAGTCGACAACAATCAGCCCAAGGTCCAAATA  ATTTGAATCGAGTCTTTAAACAGGTAGAACATAATTTACCTCGAATTAAT  TGTTCACGAACTGTTTGGGTAAAGGACATTGAGTCCAGTTCCTCGCGAGC  CAATGCTTTCCGGATATGTCCAAGTTCAGTGAAAGTTAAAGATAAATTAA  AT  >Unigene4335_All GI20575 [Drosophila mojavensis]  CCAGGTAAATCTTTTCGAGCGACCTCTCGGAAGTTGTCTCGGTTTCGAGA  TAAGTACTGTCGCAACATGTCTAAGGCACTTTCTCGGCGAATAACTTTAT  GGATCATGTCAGTGCACATTAGAATACCACCGTCGTGTTCATTGATAGCA  GTTGAAATTCCAGGCCAAATTTCAATGTCATGGTCAGATAGTCGTTTTCG  AGCAGCTTTGTCATAATAGTCACGACCAACCTGAATGAAACCAAGAGAGA  CAAGATTCCGACGCATTTGAGTGTTGTAAAGACGAAGCATCTCAGCGTGA  CCCCAACCAACGGTCGATGTTGGCTTAATACGAATGGCAATTGTTGTACC  TTCTCGAGTTGTAGCCTCGGTAGTGGTGGTTGTTGTGATTTCTTGCAATG  ATTTAATCTCATTACCACCATCAAAAATATAAGCTTTGTTGAAAACGGAC  GAAAGACTGAAAACCAAACCTCTACGAACTCGAATAGATTCAACATTTGG  ATTAAAATCAACTCGATAAGAATATATAACCTGATCATTTGGAACTCGAA  GTCGGAAATAGTTGGTGACGATTTTCATTCCACCCAAATTATCACCCGTT  GTATTGGTAACTGTTGGTGGGATAGTTCGTAGGGTTTCAGCAGGTCGAAA  AGCACCTCTACCTCGAGTCGCTGGTTCACCTTCAGAGCCTCCGCTGGTTG  TAGGCTCATCTCCGCCAGTTGATTCGGTATCTTCGGAACCTCCTTGTCTA  CCTCGAGCTCGACCACGGGATCTACCGACATCCATGATGAGTTAGATTAA  TTAGAGAGAATTATGAACGTAAATTGTTGAGTCACAAATTAGAAAAATAA  ACTAGGTGATGATGAGAAGGACCAATAATTTGGTAAAAAGTGATTAGCCA  ATTTGAAGAAGGAAAAAAATTGAAGTTAACAATACAAAAGATTG  >Unigene4228_All Similar to LIM and SH3 domain protein F42H10.3 [Apis mellifera]  TGAAAATAAGTGTTTCCGTTGTGAGAAACGTGTCTATCCTATTGAAGAGT  TAAAATGTTTGGATAAAATTTGGCATAAAAGTTGCTTCAAGTGTCAAGAA  TGTGGTATGACACTTAACCTTAAAACCTACAAGGGTTACAATAAGATGCC  CTATTGTAATGCCCATGTACCCCAAGTTAAATATACAGCGGTTGCGGATA  CACCGGAGGCTCGTAGATTGGCTGAAAATACAAGGATTCAAAGTAATATT  AAATATCATCAAGATTTTGAAAAGCTTAAAGGAAAAGTCACCCAAGTTGC  TGATGATCCGGAAACATTGAGAATTTTAAATTCCTCAAAGATGATTAGTA  ACGTGGCTTATCACGGAGATTTGGAGAAAAAGAAACAAATGGAAGAGAAA  AGATCACTTCTTTCGGATACAACCGAATTGCCCGTTGAGCCTCAAAATGT  TGTTATTAATCAAGTTAATGATACTCATGTTGTACGGAAAGTTGGCTCAA  TTGTTAATTATGACCCATTGAATGATAATTACGGATCAATCGCTTCTGGT  TATCGGCGAGAAAATCCAACAACCTTATATTTTAATCAACCAATCAATGT  GATGGGTCAAGAAGCAAATATGGGCTATAAAATGCCAAGTCGCTTATTCC  GAGCCATGTACGATTATACCGCTCAAGATACTGATGAGATCAGTTTCCGT  GAAGGTGATGTGATCTTTAATTGTCAATCAATTGACGGAGGTTGGATGAC  AGGTACTGTTCAAAGGACTGGTTTCCGTGGCATGTTACCGGCCAATTATG  TTGAACCCTATAACTGAATTAACTTTCAACAATCAACGACAAC  >Unigene4216_All Homeobox protein engrailed [Artemia franciscana]  AAAAAAGCCGACGAGAAAAGGCCTCGAACAGCGTTCACAGCCGAGCAATT  AGCGCGATTGAAGCAAGAATTCAATGATAACAAATATTTAAATGATAAAC  GTCGTCAAGCATTGGCACGAGATCTTAAGCTAAATGAATCTCAGATTAAA  ATTTGGTTCCAAAATAAACGAGCCAAGATTAAGAAATCAACCGGCTCACG  TAATCCATTGGCTCTTCACCTAATGGCCCAAGGGCTTTATAATCATTCCA  CCATGGAAAAGGAGGATGGAGAAGAAGACGAAGATGATGATAATGAAGAT  GTTGTCGATGATGATGAAGATTCTCAAGCTTTTTTCCCTTCTTCCTCTTC  TTCAACGACGAAAAAAAATTCTTCATATTTGTAACCATAGAT  >Unigene31268_All C. briggsae CBR-LIN-48 protein [Caenorhabditis briggsae]  CTCGTCTTTCTTTGTATGCATATGAATGTGGTATGCCATGTACTTTTTGTGTGTGGGATTCTAGCGAACATCGCTGTGTGAATGATTTTTCGCAATGTGAACATTTGTACGGTCGAACACCTGTATGTGTGCGTGTATGTCTTTTTAAATCGAACGTATCATTGAAACCTTTACCACAATAAAGGCATAAGAAACGTTTGATATTTGAATGGCATTTCATGTGTCGATTCAATAGCCGTGCGAGTGTGAATTTTTTGTAGCAAACAGGACATTGCATCGGATCTGATGTGTTACTTGGCGGTACAACTTTTTGTTGTGTTTGATTTT  >Unigene8777_All Similar to zinc finger of the cerebellum 4 [Monodelphis domestica]  CATCATCATCGATCACCTAACGGTACTTTAAATAGTTTATATGAAAGTTC  ATTAAATGGATACACCAACAATAATGGTAATATAAATAGGGAGACAATTA  TTGGCGGGGGCAAGCTCATCCATCATCATCACCTATTAATGGTAATGCTC  AACATCATCATCATCATCTAACTTCACCCTCACCCCAACATCATCTTCAC  CCTCAAACCCAACCACAAACCTCCCCATCACTACCTCCAGTTTCATCTCA  TCATCAACAATCACCATCAACATCATGTTTCTCTGCCTTCACCTCAGCCT  CAATGTTAGGTGCTTACCTTTACCGGCCCTCATCGGCCACCGCTGCTGCT  GCAGCTGCCCTCATGTCCGGATTACAGGGTCATCCTCATCATCATCACCA  TCACCACCTACCCCAGCATCTTCATCATCATAATCATCATCATCATTCAC  CCTCTTCCACTGCAACCGCAACC  >Unigene9101_All Engrailed 1 [Monodelphis domestica]  CTGATTTATTATTAATATTCTCATTGATTCTTTAAATGTTTTCTATTAGA  TAATTGAAATTAATTCTATCATATCATATTCTAAATTAGAACTCGACTGA  TTTGGAGGGAGAAAGTGATAGAGAGACAGAGAATCTCCATCAATTTGAAT  CAACTTAATTTCTAAATTGTCGCGAAAACAACAAATATAATCACCATTCC  CCGTCAACCAGTTCCCGGTTCCTCTTACTCTTCCTCCTCGAACTCAATTC  CACCCTCTTCATCAACACTCCACCATCAACCAGCGAATCAGTTACAATCA  TACACACACTCCCACTCTCTTCGTCACCACCACCACCACCTCCACCATCT  TCATCACCTTCAACAACCTGGACCCTCATCTTCATCACCATTGGCTCTTA  GGCATCCACAGCATCCACAACAACACCAATTGATTTCGAGTGTCACCTCA  GCCAATCCCAATCAACAACAATTGATTAAGAATCGACAACAACAAAGGGC  CGTCGGTGTCGGTGGTGGTGGCGGTGGAAACCTTGGTTCGCTTGGCGGAA  GAATGTACCTACTGGTGTCGAGCTACGAAAGTCGAACCGATGGTCCAACC  TATGTAACCACTCGAGATGCAAACATAATACAGAAATTAAAAGCTCTCAG  GATCATCGAGGAGCCACTTAAATTTACTAATTACAATTGTTACAATTATA  AATTTGAATATCCGGACATTTGTGTCCTCGATTGGTTTCAATTGCTTGGA  TATCGAGTGCAAACAATGGCCGTGACTTATGACCCCGGTGTCGCTTGTAT  CAAACAC  >Unigene13066_All Similar to homeobox protein prospero/prox-1 [Tribolium castaneum]  CAAAAAAGATCTCATTTAGTTTTAGATTATTAAATCGAGGATTCGATTAT  CTTTTGAGTTGTTTGTTTTTCTGTAAATAATCAAGATTTAAAAAGGGAAG  ATTATGATTCGATTGTTTGATTGATTGGATTGTGATTAGTTGATTATGAT  CGGCGATTCCACCTTCGTCTAACAGGTACATTCCTCATTCCAGTCTCTCT  AAAAAATTGGGCGATTTAAAATATTCGGGGACAGAATCATCGAGCCTCGA  GATGATCTTGTAGATTGCTTTTTTCCATGATTGTTCAGTGTCTTTTCCAG  CGGCGATCGATTTGAAAAACTCTTTCAGCGTTTGTTCAACCACGAAGCAG  AATCTTTCTGGAACGTCAAGATGATTACTACGATTGTAATGCATGTTAAG  CACTCGAAGTAGTTCGGCTTCTCGGTGGATGTGAATATCGTCAGCGCTTT  TAATTCCTTCGGCAATCGCTTGTCTTGCATATTTTTCCATTTGAATATAA  TAAAACTCTCTGAAATTAGAAAACCATTTAACTAATTGAGCTGTGTTGTT  CTTGTTGAATTTAATGTCGGGGAAAAATGTTTTCAGAACATTGGAACTTG  GATATCGAACATAGAAGAACATTAATTTAGCTTTTCGAAGATGCATCGGA  GTCAAAGTTGTTTGGAGAGCAAGTGAATTGCCACAAAATGACATTAAAGG  TGTTGATGTATCGTAACCGGTTGAATCGTTGAGCGATTCAGAGCCATCGG  CACCATTTTCAGATCTTGTACCACCAAATTGACTTAGAGATTCAGGTGAA  GCGGTGTCACCACCTCCAGCGGCTCTATTGGCTTGAGCTAGCATTGCAAG  GTGATTGGCTGCTAATGCATTGTGAATTGCTGCTGCTGTTGCTGCTTGAT  GGTGATGATGTTGCTGAGCTGCTGCTGCAGCGGCTGGGGCAG  >Unigene4276_All Timeless isoform A [Tribolium castaneum]  TCGGCTGGAAAAGACATTCAGGATTCCTTAAATCACCGGCAACATGATGC  ATAATAGTAAAGACACAATTGTTAATTAATTCATTATTTGATTGGAAAGC  TTCCAATACTCGACCATATTGTTCCATCACCTTGATTGTTGCAAATTGTT  GAAGATGATCAACTAGATTACAATTACCTCGTTTAAAGGGTTCATCAAGA  AGGAGCATTAAATGATGATGAGTAACAATTGAATCAACTAGAAATGATTT  AGGATGAATGACTGGTGAATAACAACGAGTTACGAGAAGAAAAAATTGCC  TCAGGGCAGTCATATTGGCTAATGATGTTCGTAAATTGTTTAATGATTGT  TTCTCTGCCTCATCGGATGATGATTTACCAATATAACCTGAAACCGTGAT  GATCATTTCTTTAAGCGCGGTAATCAACAATTGAAGTTTACGGGTTAACT  TTTTTATCAAATCATCATGAATCATGTTCACTTTAGCTTCAGATTGTTGA  TTATTTGAAGATGGAGATGAGGATGAAGATGCAAATGGACATTTGGATGA  TTTTGATTCTGTGGTCATAACTGTAACAGGTAATGATGATGATCGTGAAC  GAGGCGGTGATTCAGTGGTGGTTATTGGTGTAATTCCTACTGATCGGGTT  GAAAGAGATTCAATTTCCTCATTAAGCATTAGTCCATTGTAGACAAGAAA  TCCAAACATATCAATTTGAAGTATTGGAGATAAATGGTTGTAATTAAGAT  CAATTGATGAAGATATTTTAAGAAAATAACTGATTAACC  >Unigene3656_All Synapse-associated protein [Aplysia californica]  TGTTCGCATGTTATTTGTAATATTCATGTTGATTTTTGTTCTCATTTCAT  TTTAGTTTCTTTTCGTTTGTTTCAATTGGGATTCAACAAATAAAATTATC  ATTTAAAAACAATGTTGATTAAAATGACATGAAGACAAAGCGAGTGAGTG  ATGGGCAGTTGAGGGAACAGGTCAAAACATGGATTCTCAGAGATGGAAGA  AAGAAAGATCAGATGAAAGTTGGAAGATCCGATGAACAACAAAGATGAAA  AAGCGAAGCAATAACGAAGGCGATGCTTTTGTTATCATAATCGTGGATAA  TTAAGTAAGAATTCTGGAATCAATTAGAAGAAAAGAGAATGAGAGAGCAT  CAATCCAGAGATCATCTTTACTTGAGGCTTTCTGTGAGCAGTGGGTGAGC  GTCTGTTCGCTTACTATTAATTGATTTGTTCATAAATTGTTGATTGTTGG  TGATTTTCAAAAGTGTAATTTGAAAAGTTTGTTTGAATTGAAAATTTGAT  TAAAAGGAAAAAGAAAAATGAACATTTGTCTTCCTGCAGAAAAGGTGAAA  TTCATTTTGTCCATGCGAGTCTTTGGTTGAACATTAAACAAGCCTTGTAA  ATGTGTGTAAGTGTTTGTGTGTGTAATTAGTTTCGTGTAATTGGTTTCGT  TAATTGTAATATATATATCACGATGATTGGAGAAGATGAAGATGATGGGA  AAGAGACACAAAAGTTAGGGAGAGAGAGAGGAAAATACAATGTGTAAAAG  AGAGGAAAAAGACTGATGGTAAAATATTATGAAAGGAAGATCTGTTTTGA  TTTTTAGCAATTGGATTCTATTGGTAATCAGTAGATAATCTTCATAATCC  TTGATCTCTCGGGCGTATCCATATGACAGGGGAATCGTTTACGTTTATGG  CAGATTTAACTTTTGCATAGATCTCCTCAGGAGAATCTCCGATGACTATT  GATGTGAAATATTCAGCGAACTCTTGTTCCAATTTGATGGCTCGTTCATA  TAATTTTTTAGCTTCTTCCTCTGGAAACCTTTTATTCATCTCCATAAGTG  AAGCAATTGATTTGGGCTTAATAAATATGGCTATGGGTTGAAGTCCGGCT  GCTTGGAGTCTTTTAATTGCACTTCCACTC  >Unigene8949_All Similar to sex peptide receptor [Acyrthosiphon pisum]  CGGCCCTGGTAGTCCTTAATCTGGCCTTGTTCAGGGCTCTTCGTCGTGCC  CAATCAAAGAGGAAATCGTTATTCAAAGAGAATCGAAGGTCAGAATGTAA  AAAACTTTGCGACACCAATTGTACAACCCTAATTTTAATCGTGGTGGTTT  CGGTTTTCCTGGCCACCGAATTACCTTTGGCCCTTACGACGGTTCTCCAT  GTGATTCAGAATGCTCTTAAAATTCATATTGCAGGC  >Unigene19411_All Similar to slowpoke CG10693-PQ [Tribolium castaneum]  GTTGGTTTGGCTGTATTTGCGTCTTGTATTCCTGAAATTATTGATCTTATTGGGTCCCGATCAAAGTATGGAGGAACCTTCAAATCGGAGCGTAGAAGACATATTGTTGTCTGTGGACATATTACCTNNNNNNNNNNNNNNNNNNNNNNNNNNNNNNNNNNNNCTTCATGAAGATCGAGAAGATGTTGACGTTGAGGTTGTATTTTTACACCGAAAACCACCCGACCTGGAATTAGAAGGTCTAATCAAACGCCATTTTACAACAGTTGAATTTTTT  >Unigene21814_All Similar to large conductance calcium activated potassium channel pSlo spliceform 1-5A  AATTGTGTTTCATTAAGTTAAAGTTACTTTTATTGGCTATTGAAGTTGGAAGTGAAGATGGAGGTGCATCCATTGTGATCAATCCTAAAGGCAATCATCGGATTCAAAGTACAACTCAAGGTTTTTTTATAGCCCAAAGTGCTGATGAAGTTAAAAGGGCTCTTTGGTATTGTAAAGTTTGCCATGAGGATGTTAAAGATGAAAAGATGATCAAAAAGTGTAAATGTAAAAATC  >Unigene22663_All Slowpoke [Nasonia vitripennis]  AAACAAGATTGGGCGATAAAACCTAATTTAAGTTCACTAACACAAATAACATCATCACCTCTCCGCCAATTCCAAGAGGGTATATTCAAAAGATAGGCTTTATTGTGATATTGCATTAATTGAATAATAACCCTAATATCATCACTATAATTTTTAATTGAAATAACTCTCATTATATTTGCAGCGTCTTCTGCATCCGGATCTTGGCAATATTTATTTGCTAATACAAGACAAGCATCGGCATCATGAACCTTTACTCGTTGTAAATCAATGGGATTCATGACAG  >Unigene23313_All Slowpoke, isoform S [Drosophila melanogaster]  ATTTCTTCGAGCTCTTCGATTAATGTCCATTCCAGATATTCTTCAATATTTAAATGTTCTTAAAACATCGTCTTCTATTAGACTTGCCCAATTAATCTCAATTGTGATCTCAGTTTGGTTAACAGCAGCCGGAATTATTCATTTGCTTGAAAATTCTGGTGATCCATTTGATTTTAAGCCTCAACATACAATTTCCTATTGGGAATGTGTTTATTTCTTAATTGTTACCATGTCCACCGTTGGATATGGTGACATTTATTGTCAAACTATGATTGGCAGAGCTTTCATCGTACTATTTATCCTCGTTGGTTTGGCTGTTTTTGCTTCTTGGATCCCTGAAATAACTGAACTGGTGGGCCAACCCAATAAATATGCTGGCAATTATCACACAAATCCTTCCAAAAAACATATTGTTGTCTGTGGACATATTACCT  >Unigene12265_All Similar to zinc-finger homeodomain protein 1, partial [Acyrthosiphon pisum]  GACTAAATCACTTACAGATCGTAAATTTAAGTGTCCCGAATGTGGTAAAG  CTTTTAAATTCAAACATCACCTCAAGGAACACATAAGAATCCACTCAGGT  GATAAACCCTTCCAATGTTCCCACTGTGGTAAACGTTTCTCCCACTCTGG  ATCATATTCATCACATTTAACAAGTAAAAAATGTTTCAAAGTTAATCTCA  AAGTGTCCAACACCAACA  >Unigene13122_All GK21745 [Drosophila willistoni]  AAATGATAATTAAAAGAGGATAAGATACAAAACAATTAAATTTGATTGTC  AGACAAAAGTATTGATTAGCAAAAAACAATCGAATTGGTTTAAATTACAG  AATTAAATTGTCTTTTTATTTCAAAATGTTTCCAATTGCTCTTGATTCTC  ATCCTTTGGTATCGATTTATTATCACATTCACCGATTGACCATTGGTCAC  CTGAAAATCCTAAAAATAGGGTACAGATAAGTGTAACTCCAGCACCGAGA  ATAAGGCCAACCAGAACATCGGACCAATGGTGAATATTATCGGAAACTCG  AGAAAGTCCAACATAAAGAGCGATGATGAGGAACGACATTTGCACCGAAG  CAATTAGATACCTTAAACTGTTTGTCCGATTGATTAAAAATCGCTGACCA  ATATAAACAATAAGAAAGACCATCGAAGACATTGAATAAGATGAATGACC  GGAAGGAAATGAGGTTCTAATGAAAATCTCCTCCGATGGTTTAATATTGC  AAACATAATCGGTCAAATAGACTCCATGATTTTGTTGATCATCACAAGAG  ACACTCGATTTACATTGAGAGTAAAAAACTGGCCTCAATCGGCCGACCTG  AGTCTTTATGAATCCGGTCAACAGAGCGGTCAAGAACAAACTAAACAACC  AACTGATGATAATGGAGTTGATATTAACTGACCAAGAGGAGACCGAGTGG  TCAGTTGAAACTTTCGCTGCTTGTTCAATGTGATTGGAACCATTTACATT  TAATTGTCTTCGCCAGGATTTCCTTGTGTTTAGATTCAATTTATGAGCAA  TTAATTCACCCGAAATGATAACAATTAAAGGTATCACCAAGACTATAACA  GCTAATTGACCACCACCAACAGTGTCCGGTTGCCTTGGGTATTTAATTGA  TTCATCTGAGCACCAGAATCCTCGTCTATTGGGCTTGGCAATTGAAACTA  AAATGATTCCCACAAACAAGATAATTAAAGTTGTCACATTGATTAAAGTG  CATTTTAATTTCATGGCAATCAAATTGAACAAGAATTCAAATATCAAACA  ATTTAATTAAAAGGGTTAAAAAAACCAATTCACTTG  >Unigene13947_All BAB-I protein [Drosophila melanogaster]  CGACGTTTGTTTGTTTTCTCTTCTTTACAAGAGTTTTTATTGAAATTGGA  TTGACTATTTTTTCGTGACACTTGGAAAACCATGGCACCTCCATCAGCAT  CAGCTTCATCTTCATCTATATCGGATCAAGCATTTTGTTTGAAACTTAAT  AGTCACCAAAATCATTTGGTCTCAGCATTCCAAAGACTTTACAACGATGA  AATTTTAACCGATTGTACTATTTCCTGTCAAGGTGGAACCCTCAAAGCTC  ATAAATTGGTTCTCTCTGCATGTTCCCCTTATTTCACAGATCTCTTTGCA  TATTTTTCTAACCCAAACCAATATCCAATAGTTATACTCAAGGACATGTC  AATCTCTGATCTTGAAGCAATTATTGAATTTATGTACAAGGGGGAAGTTA  CAATTCCTCAAAGTAGTTTACCCTCAGTTTTGGACTCAGCTAAAGCGCTA  ATGATTATTGGACTAATGGACATTAAGGTTGAAACTAGTCCTAAGGATAA  GGAATCTTCGCCTCGCGGTAGTTACAAAAGGAAAAGACGTCAACGTACTT  CTATTAAACAACCACAAGCTGAAGAGATTTCAGAATCCGATGTTTCGGAA  AATGATCTCTGTGAAGATGAATCTCATGCAAAAGTTTCTAAAGGAGAAGA  AGGAGATCTCGTAATTCTCCAACACGAGGTCTTATCCAAACCCTCTGAAA  TAATAATCGAAAGTGCATCCAATGAACCACAGGCCAATGTTAATTCTCGT  TTAGCTGAGTATCCTCAAGTTTTAAACGAAGCAGATGATTCGAGTTGCTC  TTTACCTGGACCATCAACTCGTTACCCTCGCCCTGTCTGTAAATTGTCTC  CATCAGCAGCTTTTAAAGCCAATTTGAGACAAATGAGAATAGAGAAAATT  CAACACTCAACGGGTGGATTAAGTGGTCGAAAAATGTGGTCGGAAGAGGA  AACAAAAACACTTGTTGATGTTTGGGATGAAGAATCAGCCGGTATTTGGA  GATCATCAAGTAAAAAAATGATATCTCTTCAAAGGATAAGTGATATTCTA  CAACAGCGTAACGTGGATCGTGATGTGTCGCAGGTCGAAGGTAAAATCAA  AGCTCTAAAACGTGATTTCAAAGCAGTTAAAGCAAATAAAGCGATCCCAT  CAGTTCAAGCTCGAATGGCTCCTTATCTTGACAAATTGGAATCAATTTTC  CTAAGGGAAGAATTATGACAACAATTAACTAGTCAACAATCATAAAAATT  CAATTTACAATTTGGTCTCTAATTGACCAACCTTAAGCTTGGAATATATT  CAACATAAACTAATCTTTTCCCAACAACTACAACAATTAATTTTATCAAT  CAACATAAATGACGATAAAACTTCAACAGGTAAACTCTCTTCAATCGATC  CAGG  >Unigene29146_All Mismatch repair protein [Mesocricetus auratus]  ATAACTTTTATCGAAATCAAACCCGAAGTATTATCATTGACGGAGTACAAAGTATCTTCAAGCGAACCCAAATTACCCGGTGAAGCTTTAATGTCAAGAGTCCAATTAGCGTTACGGCAAGTGATGGGATATTATCAATATACTTGATAACTGTTTGAGTTTTCAAGATTTCATTAGCAACAAAAAGAGCATCAGATCCATGAATGGTGAAGAAA  >Unigene13124_All Cyclin D2, b [Danio rerio]  AACTGGATGCTTGAGGTTTGTGAAGAAGAAAGATGTGAGAAGGATGTGTT  TCCTTTAGCCATGAACATCATGGATCGATTTTTGTCCCAAGTTCGAATAA  GAAAAAGTCAACTGCAACTTCTTGGAGCTGTTTCCTTGTTCATTTCGTCC  AAAGTTAAACAAATCAAACCTCTTCATGCTCATCGTCTTATCCTCTACTC  CGATTATTCAATTACAAGAGATCAACTATCGAATTGGGAACTTTTAGTTC  TGAGCCATTTAAGATGGGATACAAGTGCTGTCACTCCGAACGATTTCCTC  AACCTGATTCTCCATCGTTTACCTACACGAACCAAAGGAAAAGATAAATT  AATGACCATCCAAAAACACGCACAAACATTCATCGCTCTCTGTGCCACAG  ATTTTCGTTTTTCCATGTTACCACCATCATCTATCGCAGCTTCTTGCATT  GCAACTGCGTTTCTCGGTTTAACCAGAAACAATTCACAAACAACCAAAGA  TTTGATCGCTCAACTTTGTAAATTAACCAACATTGAGAACACCGATCTGC  TGGAGATCAGGAGGACCATCGAGGAGGTTTTAAGAGCCCAAACCAACCAC  AAATGAGAGTACACTGGATATGGAAACGGAAATAGTGGACTTAAGAGAAA  TTCAAAGTTTTCTCGAATTCAGAAAAGTTACTGTAAATGCCAAAACAAAT  CGAAAATCAAACTCAGAGAAACAACATTAATTTCGCAATCATCAAGCATC  GCTAAAATCTTTATCCAATCCAAACAACTCATTCGCTTCCCAATACAAAC  CAACTGATAATAAGTCAAACTGATTAATGGTTGGTAATTGTGTCGTCACC  TCAATTAATTTTGTGAAATTCAACCACAATTTAGTCAACATCAGATCTTC  TTACTGTGCTCATGTGTGTTGCCCTATGATGATGTTAATCCCACTTGAAT  GAGAACGTGCAAATTATCAAGAACATCATCAATTTGAAAGGAACCTAATC  CATTTCATCTTCATTTGTATCATTTATCATCACCAAAACACTCATCTCTC  TCTCTCTCTCTCTCTCTC  >Unigene31926_All Similar to mutS homolog 4 (E. coli), partial [Taeniopygia guttata]  CGTGGGCACAAAGCTGTGTAATCGCTTTAAAACCAACCATATCATTGAAACATTTTCTATCTACTTTGGTGACGGTGATGGTGTTATTAAAATTCAGGAGAAGTTCGAGTAAATTTTTGTTAGCTCCTTCACCACAGGTTGATGACACTATGATCTCAGCTGGGTCCAGAATTTCAAGCTTGGTAAACAGTCTTGAATAAGTACAGCCATCAGGAAATTGAAAAAGTTTCAAAGTTGAACCAACAGCATCCATGGAAGCCAAACCTATTTCACCTTTAGCATTACCACGACCCTCAGCAATTGCAATTATTGTTGATTTAGTTCTGTTACCAGATATACTTGAAGGTCCACTAATTGAGCCGGTGGTTGAGTTGGCACGAAAGTCACTAATAGAAACCAGCGAATTCCAATCTCTATTTCTAATAGATTCAGTTTCAAAACTGTCCACACCAAAGATTGGAAGGTCATACTTTGAACCCATAATCAAAACTATGAACCAACACTTTGACCACAAGAGATATGAGAAAGAATCGATTACAATCAAACGTTTAAT  >Unigene6518_All MutS protein homolog 4-like [Xenopus (Silurana) tropicalis]  CATCTCGACTGTGGTAAAATACATAGTTTTACCTCTTTTTTGAACACGAA  CAAATTCAACCGGTACATGGTTATCCATATCAGGTCGATCAGCAAGGTTC  ATGGTCATATGATAACCTCTAGTAGCGTTGACATTAATTTTGATGGGAAG  ATTGTATTTTAATTTGAGTGAATCAACATAACGGCTCATATCCTCGATCG  CTCGATTGTAAATTTGTCTCTTTTTATCCAAAAATCCATTAATTTCCTGC  TTGATGGCAAACACTTTTTGATGTTTAATGTTTAATGCACCTTTCACTAG  TTTACAATCCTTACTGATTGCAGTGTTGATAATTGTAGCAATTGCATCGA  AATTTGGATCATCTAATAAGTAATGATAATGTTGAAAGATTGAATGTTGA  GCCGAATTTAATTGCTCTCGAAGTGGC  >Unigene31214_All Host cell factor C1b [Danio rerio]  ATTGTGGGCAACAAAATTCTTGTACTGTTTCTAATGCTCATTTAACTAACGCTCACATCGATACCACAACTAAACCAGCTATCATATTCCGCATCGCCGCTAGAAACGAGAAAGGTTACGGTCCCGCCACCCAAGTTAGGTGGCTTCAGGATTCGGCGAACTTCGCCGCAGCCAAAAATGCACCTAAACGCCCTAATCAACCTTCATCTAACGAGCATTTCACGACGAAGCGTATCAAGCTTGAAACGGAACAGTAATAGGACAAGATCTTACAGATCAACAACAAAGATTAAACTGTAATTAAGCGGAAAGAAGGCC  >Unigene4342_All Host cell factor C1 [Mus musculus]  ATACACCAGCCGTATTATTGGTTGATAAAGTTGATGATGAAATGGGAGTT  GTAACGATCTGTGGGGTTAATGATGCGGTGGTCGTTCTAACAACTGGTGT  CACAATTGTAGAAGCTGTTGTAGTAGCTGTGGTGGTTTGGCTTAATGTTG  GTGTTTTACTAAGATTTGTACTGGCGACAGTTGTCCTTGATTGGATAAGA  TTAGAATCACTAATTGTTCTGAAAACATTGGATATGACAGTTGGAGCTGT  CGTCACTAATTTAGACTCAGTGACCGTCTGAGATTGAATTGGCGTTTGGG  TTGGAGATTGAAGAGAAGTAAGTGAAGACTGAACTATTGTGGGAATTTGT  CCTGGATTTTGGGAAAGAGGCATCTTGACAACGGGTTGCTGAATGACTTT  GGCTACTTTCGATGAATTTTGGACAGGTATTGATGGTAAGGTAGCAGGTG  TCGCGGGTGGTGTTTTGACTAATGGTATACCAGGTGGATGTTGACCAGTA  GTACCACTCGAATTTGCTGTAGTCTGAGGAGGATCATAATTTTGAATTTG  AAGAATATAACTGTCTGCAGTTGGTACAACACCCCAACATACTTCTAGTG  TATCTGTTGCTGTACGAACTAATTGTACTCTACTGGGAGGTGAGGGTTTT  TCTGTTTCAAGGAACCAAAGATCTTTACAACAGACTTGATTATTCCAAGC  TTTACGATAACCGTCTCGACCTGACCATATATACAATCGAGTTGATATTG  CAACTGCACAATGACCAGCACGAGCTCTTGGTACACTGTCATCAAATGAT  TCATTGGTGATTAATTCCCATCTATTTGTGTCTAAATTAAGGGTTGCTAA  GGTATTGGTACATTTCCATTCTTTTTCATGTACATTACCTGGCTGTTGAC  CTCGATTATCATCATCATACACAAGTGGTACCCAGCCACCGAAAACATAC  ATTTTATTACCAATAAGAGTAGCAGAATGAAGTGATCTAGGTAATGGAGG  AATACCCTCAACGAAAGGTTTTGACCAATGCATAGTTTGAATATCCAACT  GCCATAAATCCCCGAGTCTACATCCTGACATCCCGCCATATATGATAAGC  TTTGGATGTTTACCATCAGCTCCCATGTACGCAACTGCAGAATGACTCTC  CCTTGGAGGAGGAGGTGTACCATAGGTTTGCGCTAATTCCCATTGCATGG  TGGACAGAAAGGGTTTCATTTCAAGAACATATAAATCATTTAAATATCTG  GGAATATTGTTCTTTGGATCATCTGAATCATTAGCTAAACCTCCGAAAAG  ATAAACTTTGTTCTGTATAAGGGTAAATGAGTGTCCAAGCCGTGGACATG  GATAAATCCCATTCTTGGGTGGTCTTGGTTTAAGTTTCCTCCATTCCCAT  CTAGATGCTTGTAATTCATATAGTTCATTACTGTATTTTCCATATTCGAT  CATGCCCCCAAATACGAGTAAACGAGTACCATCGCAGACAAAACCGTATG  CCGCACATCCCGGTGGTATTTCACCTTTTACTTGTGGTATAAACCATTGG  TTAGTAGTTGTATTGTAAACATGTAATTCATCAACTATTCCCTCATTGCC  ACCACCAAATATTATAATCAAATCTTTTATAGCAACCGCACGGTGACCAT  GCCTTGGACGCGGCGACGGCCCAGTACTATTAGCCACTCGCACCCACTTC  ATTGATTGTAACAATTAAGCTTTCGGATAGAGTTACGACAAATTGTAGGA  CCGGTATTTTGTCCGAAACAATAGTTAAATAAGATTTAAATTGTAAACAT  TTTTAATTAAAGAAAATGAGAAAAACAAAAACAAATTATTAGTTGTTG  >Unigene17844_All GK16650 [Drosophila willistoni]  CTTTTATCGGCTCTTTGTGTTTATAGTAAGGATGGACATTGGAAAGCTCTGCAAACCCTTAATCACTATCAGGCACTTAAACGAACTCGATATCGATTTGAANNNNNNNNNNNNNNNNNNNNNNNNNNNNNNNNNNNNNNNAGAATTTGGAATATAAGACCAAACTATTGGCCTTCATCAATTGTTTAATCATATCAGCGGGC  >Unigene17339_All Similar to Ini1b [Strongylocentrotus purpuratus]  AGATCTAAATCATCGCAAAGTACTTCAGCAAATAATTCGGGAGTAATTAATTGTTCATTTTTATTCCTCGTAAATGTATCTCTTAATTTGTATCCATCAATTTCCATATCAAGACGAATAGGGACAAGAACATCGGGTTGCTTAGCATTCTCATGGATGATAGCAGAGTCTAAATCATCGAATGCCAAAGGAAAAGTTCGAACCTTTTTACCAGAAAGACGATTTCGACTGACT  >Unigene17433_All Similar to Ini1b [Strongylocentrotus purpuratus]  AGTCAGTCGAAATCGTCTTTCTGGTAAAAGAGTTCGAACTTTTCCTTTAGCTTTTGATGATTTGGATCCTGGAATAATTAATGAAAATGCTAAGCAACCTGATGTTCTTGTTCCTATTCGTCTTGACATGGAAATTGACGGGCATAAATTAAGAGATACATTCACTTGGAATAAAAATGAACAATTGATTACCCCCGAATTATTCGCTGAAGTACTTTGCGATGATTTAGATCT  >Unigene3588_All Suppressor of Ty 5 homolog [Saccoglossus kowalevskii]  AGGAGGAGGACGAAGATGAAGAAGAAGAAACCACTGGGAGAAGAAGACCC  AAGAAAAAGCCTCGCCATGGTGGTTTCATTTTGGATGAGGCTGAAGTTGA  TGATGAGGTTGAGGAAGATGAAGAATGGGAAGAAGGAGCAGAAGATATTA  TCGAAAGAAATAGACCTAGTGAGTCAAGCTCATCTCGAGAGCTTGACAGT  CATCGTAGATTACAAATGATGCTGAATAATCAAAATGAAGACGAGATTGA  AGAATATTACAAAAGGAAATATGCCGAGACAACAGCGGCCGAAAAAGGTC  TTTATGATGCTGAAGGTGATCTTCCCGATGATATTGCCCAACAAGCTTTC  ATGCCCGGAGTTAAAGACCCCAATCTTTGGATGGTTAAATGTAAACCCGG  AGATGAAAAGGCAACAGTACTTCAAATTATGCGAAAATTTATCGCCTATT  CCACCACTGAAGAGCCTTTGTTGATTAAATCAGTTGTTGCTCCTGAAGGA  ATCAAAGGTTACATTTACATTGAGGCTTACAAACAAACTCATGTTAAACA  AGCAATCACTGGAATTGGTAACCTCAGAATGGGGCAATACAAACAAACTA  TGGTACCGATTCATGAAATGACTGATGTTCTTAAAGTCACCAAAGAACAA  GCTCTAATCAAACCAGGAACTTGGGTTAGACTGAAAAGATCGGTTTACAA  AGATGATTTAGCTCAAGTTGATTATGTTGACACGGCGCAAGGACAAGTCC  ATCTTAAGTTAATTCCAAGAATTGATTACACACGGAAAAGAGGAGCTCTG  AAAACAACGGAAACAGAGATCGCTAAAAGAAAAAGAACCAAACGTCCACC  AGCTAAACTTTTCGATATTGATGCCATTCGCTCAATCGGAGGTGAAGTCA  GTAATGATGGAGATTTTGTCATTTTCGAGGGTAATCGATATCGACGAGGT  TTTCTGTATAAAAATTTTCCAATCGCTTCACTCATAATCAGCGGTGTTAA  ACCCACTTTAGCTGAGCTCGAAAAGTTTGAGGAACATCCTGAAGGTGTGG  AAATCAACGAAAGTGCAGCTGATGATGAAGAATCTCATGGCATTTGTTCA  GGTGACAATGTTGAAGTTTGTGAGGGTGAATTGGTTCACCTGCAAGGTAA  AGTTGTCGCTGTCGATGGAAGTAAAATCACCATGATACCGAATCATGAAG  ATCTCAAGGATCCACTTGAATTCCAAGCTCATGAATTGAAAAAATATTTC  CGTGTTGGTGATCATGTTAAAGTAATTGCTGGCAGATATGAAGGAGATAC  TGGTTTAGTTGTTCGAGTGATAGAAAATCAAATTATTCTATTTTCTGATT  TATCCATGCACGAGATTAAAGTTTTACCCAAAGATCTTCAACTTAGTCCT  GATATGGCCACTGGTGTGGACAGTCTTGGACAATATCAATGGGGAGATTT  AGTTCAACTTGATGGTCAAACTGTCGGAGTGATTGTGCGTCTTGAGAAAG  AAAATTTCCAAATTTTAGATATGCATGGGAAACTAATTCATATCAAACAT  CAAGCGGTTAAAAATAAACGTGATACTCGTAAAGCTGTTTCATTGGACTG  TGATCAGAGTCAAATTCAAGTTAAAGACCACGTTGAAGTGGTTGATGGAC  CTCATTCTGGACGAAAAGGAGAAATTCGTCATCTCTACCGTAACTACGCA  TTCATTCATGATCGTTTACACATGGAAAATGGTGGTATATTTGTTTGCAA  GACTAGAAACATTCAGCTATCTGGAGGCTCAAGATCTACATCTGTTGTGT  CCGCTGGAAGAGGAGCTGCTCCTTTCATGTCTCCGAGACTTCAATCACCG  GCTCATCCTGGTGGTGGTGGAGGTGGTGGAGGGAAAAGACCATTTGGTGC  TAACAAGGGAGGTAGAGATCGTTCAGATTTAGATTTAATTGGTAAAACTA  TCAAAATCATTCAAGGTCCATACAAAGGTCATATTGGTATTGTTAAAGAT  GCAATGTCAACAACGGCTCCAGGGGAGCTTCACTCAACATGTCAAGTTAT  CAATGTAGATAAACTTCGTATTGATGTGGTCGGTGGAACCACTGGTCAAC  GTACAGGAAGTGCTTCAACATATTCAGCCCGAACACCAATGTATGGATCT  CAAACACCATCATATGAATTGGGTGGTAGAACACCAGGTAGAGGTGGTCA  AACTCCAATGCACGATGGCTCACGAACTCCAATGCACGGCGGTAGCTCCG  TTTGGGATCCTAATACTGGAGCCACTCCGAGACCGAACTTTGATGATGAT  GCTTGGAGTGCTGAAAATCCTGGTGGATTATGATAACCACCTACTCCAGG  TTACACAGGTCCAGACACTCCTCAGGCCGGGGCATACACACCTCAAACAC  CCGGAATGTACACTTCTGACCATAATTACTCCCCATACCAACCCGTACCC  TCCCCTTCAGAGGCCAGTTATCAAGAGTCTGCACCAAGTCCTGCTAGTGG  ATCTTACATGTCACCAAATCCAGGAGGTTATGGATCTGGAGGTATGACAC  CAAGTCCACAATTCGCTGGCTACTCTCCCATGACTCCTGGAGCACCATCA  CCAATGTTCAACCCACAAACACCAGGAACTGGCATGGATCAAATCAACAT  CGACTGGCTAACAACTGATATTGTAGTAAGAATAAAAGAAAACCCTGATA  GTGATCTTATTGGACAAACTGGTGAAATCACTGGAATCAACGGTGGAATG  GTTTCCGTTTTTCTTATACGAGAAGATCGTGTGGTCAATTGTTTCCCTCA  TCAATTAGAACCAATTTCCCCAGAACCTAAAGATAGGATTAAAGTAATTT  TCGGTGAAGAAAGAGAGCAAACTGGTGTTTTATTAAGTATCGATGGTGTT  GATGGTGTTTGTCAAATGGACTCAGATCAAGATGTAAAAGTCTTACACTT  ACGGTACCTTTGTAAGATGAAAGCACAAGCATAGGAACGAAGGTCATTTG  AATTGACAATCGCTCATCAATTCACAAAATAAAATAACTTTTTCCGATTT  TTTTAATGAAAACCTCATGACTGAATCAAAACAAATCGTCATGATTTTCT  GAGATCTTTAAATTCTGACTTGATGAATATTCAGTTCTCAGGAAAACCTT  CAAATGTTGCTGCTGATTTGGTCTTCGCTCTTTGTCATCTCTCAACATCG  GAACCAATACAAATTAACGACGAACATTCAGATTGACTTATTCATTTTTT  ATCTCAATCTCCATCCACAGAGATTAAAAAAGAAAACCTCAAATCTACTT  CAAAAGTTCATTGATTATTTTCCTCAATCTTTTGATCTTCCTTCCC  >Unigene13029_All Tropomyosin-2 isoform 4 [Bombyx mori]  TTTTTTTCTCGCTCTTGGAGTAAATCCTGTTCCAATAGATCTACTTCCTT  TTGCAGTTTGGCAACAGTTCGTTCAGCAAATTCAGCTCTTGATTCAGCCT  CTTTCAATTTGTTCTTCAACACTTCAATCTGATTTTCATATTTTTCTTCT  CTTTTTTGAGCCTTTTCTTCGCTGATTTCAAGTGATTTTAGGTTATTACC  AATGGAACGAAGCTCTTCTTCCAAATCAATAGCTCTACGTTCACCTTGGG  ATGCACGTTCTTCAGCACGTTCCAGGTCAACTTCCAACATTGCAATACGA  CGAGCCATCTCTTCGTACTTCTTATCCGATTCTTCAGCAATAATCCGAGC  TTGAGATAGTTGAGTTTCTAACAATGTAATCCTATCATCTTCCATGTTCG  TCCGGTTTTCAAGGGCTTTTCGTATACGTTCACTCTCATCAGCGGCCTGT  GTTGCCTCTTCCATTTTCAGTGTTACCTCACGAATACGATCCTCTGCCTT  TTCCAGACTCTGTTCCAACAATTGTATCCTCCGACTAAGCGAAGCCGTTT  CCAATTCAGCCTCTTCACGTTTCCTAACCTCCTCCTTGTATTTAATCAAG  GTGGTCTCATTCAGCTCATTGGCAGCCACCAATTCATCTTTTAGAGTTCT  CATCTTCTTCTTCAAGATGTCACGTAAATTTTGATTAGAATTAGCCATCA  TTATGTCCACATCGCGAAGTGTTGTTGATTCTTCCTTACGATTTGCCATG  ATACACAAATAGTATTTCTCTTTTGTTTTAATGTTCCACACAAAAATTGA  CTAATAGAAAATTAAATAGAACACAGAAAGACAGAGGAAAGAAAGAGAAA  GGGAAGGTTAAGAGAAAAAAAGAGAAAGGCAAAGATAGAAGAAGAACAAA  AAAAACAGAGAAACAGAGAGATGAAAATGTGAAGAGGAGA  >Unigene11211_All Beta-1,4-galactosyltransferase [Aedes aegypti]  CTCTGTTGATGTTAATTTTTTAATAAAAATGCTTGTTAAATTGATTGAGG  TGCCGGTGCCGAGTAAGAATCATCCAGGTTATACAATTTTTGTAAAAAGT  CATTTATGCAAATTAATTGCTGTTTTACTACTATTGTTAATGTTATCTTC  ATCAAATGTAAATAATAGTGCCTCAGAGACAATATTTCCATTGATTAACA  ACTATGGTTCCTCAACAATCAACATTTCTAGTCAAAAAAATCACCATCAA  CAACATTCACAACAACATTCCCAACAACCACAATCACAACCACAACCACC  ACAACAACAACAACAACATCAACAAGAGCAGCAATTACAGCATCATCAAG  ATCATCAACAACTTTGTCCTTTAATCCCTCCTAAATTGGTTGGCCGTTTG  AAGGTTATGACCGAGATTAAAGATTGGACTGAATTGGAAGATGAATTACC  AAACATTGAGCCAGGTGGATGTTTTCACGTTAAAAATTGTACAGCTCGTC  AAAAAGTTGCCATCATAGTACCATATCGTGATAGAGAGCTCCATCTACGA  ATATTAATTCATAATCTCCATCCTATGTTACAACGTCAGCAAATAGACTA  TTGTATCTTTATCATAGAACAGATGAAAGATACGAGATTCAATAGAGCCA  AATTGTTTAATATTGGATTTGTTGAAGCATCAAAACAACAAGATTATGAT  TGCTTTATTTTCCATGATGTTGATTTGGTTCCTGAAGATGATAGGAATCT  CTATAGATGTGCTGAACAACCAAGACATATGTCAGTTGCTATCAATACGA  TGAAGTACAAACTTCCTTACAATGGTATCTTTGGTGGTGTCAGTGCTCTT  TCAAAGAGTCAAATGAATGTTCTCAATGGGTTTTCAAATGAATATTGGGG  TTGGGGTGGTGAAGATGATGACATGTCTCATCGTATTAGTTTTCATGGAT  TCCGAATAACCCGTTACCCTGAAACAATTGCTCGATACTCAATGTTAAGG  CATCGAAAAGAAACACCAAATCCTGACAGATACAAAAAACTATACTCAGC  TAAACGACGATTCAGAACCGATGGATTGAGTAGCCTTAAATACAAAAAAC  TTGATACCATCTATCAGAAACTTTACACTTGGATACTCGTTGATGCCCTA  CAACCCAAAAAATAATTGGTGATGATAATCACTTCTCAATAATATCTAAA  TCAAGCAAAAGGCAAAG  >Unigene11329_All Hormone-sensitive lipase [Salmo salar]  ATGCGAATGGATTTCGCACACTGATCAAATTGATGGAAAGCTTTTCGAAA  TTAGCTTATGATGATATGAAAATGAAATCATTCGGTGGTGTGTCTCCTCA  TTTAGTCAAAATCGCTGAAGTTTTTGCTAACATGTTGGGTCATCTCGATG  ATCGTAAAGTATTTGAAAACAATAGTTTAATGTGGTCACCAGATCCAGAA  TTCATCCAATTCGCTTTTTCCTTCGAGCCTTTGATGCATTCGTTTTATGC  TCGTAACCACAATTTCTGGTTGTGCCCTTCAATCCGATCCATTCTTGGTA  ATTATATTTCATTGGTCGCCTACAAGTCCGTCAATTTCTGTGACATGTAT  CGACTCTTTTCACGGGCCAGTACTTCTCGTTTATTCGCGGATTATGCCCT  CAACGTGTCCATCGATTTCGTTCGTATGGTCTGGGGTATCTCCGAAGCCG  CTTTCTACAAGAAACTTTATCCAATGATTCTTTTTGGGAACCGACCTTCT  CGGAAGAAAACGATGTTCGCACCAAGACAAGAATATGCCAAAATTGTTGG  AGAGAACGGGTTAATCGACTTGTCGGACTCATTAAAAGATACATGGAATT  TGAATAAACCTGTTCGCTTTCGCCTTCTCCAAGATACTAAAAGTGGTTTA  TCAACATCTACTCTGGTTTTCCACTGCCATGGGGCTGGTTTTGTTGCTCA  ATCGCCGGAGTCTCATGAGATCTACTTAAGAGATTGGGCCATAAAGCTCC  GCGGTGTCCCTTTGATTAGTGTTGATTATCGTTTGGCTCCAGAATTCAAG  TACCCAAATGGACTCCAAGATTGTCTCGATTTGTATCTTTGGGTGACCAG  TGGACTTGACGAAGTTCTTGAGCTGATCGGCTTCCATCCGAAGCGAATTA  TTTTGTGTGGCGATTCTGCAGGCGGTGGTCTGTGTATCAGTTTAACTTTA  GTTCTAAATGAATTGAGAAGATCGATGAATATTCAAATGCCTTCGGCATT  GATTCCGTTTTATCCCGTCTGTTCATTACAAACTTGTGCCTCTCCATCTC  GTTTGTTGACATTTTTTGATACTTTTCTACCAATTGGTGTTTTACTCAGT  ATCTTGGACGCTTATTTCGTCGATAATAACTCATCCGATCCTCTCAAAAT  TGACGGAAAAGAAAAAAGTAATGCAATCAAACAGAAACAATCTTTCGGCA  AGAATCAAGATGTAGGTCCACATTCCATGAGGCCTCCGTGGTACAAGGAA  ACCGATTACAAAGAGAGATTGGCATCAATCAATCGAATAGCAGCCGATCC  ATTTGCATCGCCATTAAATTATCAAAACTTTGCCACATTAAACGATGTAG  CACTTAACATAATTGTTGGTGAATTCGACCCTTTATTAGACGAAGCAATT  GAATTTACCAGAAAATGGAAAGGTCCAGTCTCTTTCGATGTTGTACCCAA  CATATCACACGGGTTCTTGTATTTCGCCAACATCAGTGCCCACAGCAAAA  AAGCGACCAATTTGTGCTTTCAAAGGCTCATCGAGGCCGTTAATTTCCCT  CAAGCAAAG  >Unigene12786_All Selenium dependent salivary glutathione peroxidase [Ixodes scapularis]  AACCAGGTAAATTGTTGGTAATCATTTGGATAATTCATCATTCACAATTG  TCGCTGTGTGGTTTTTAAATTGTCAATCAACTGTGCTAACTATTAAAACT  AATCAACCAACAATTTGATTGGTTATACAAAAAGTAAAGTCAACAATCAT  TATGATTGACCAATTGTGCTGCTTATTGACGTTAATGGTGATGACCATTA  CCGTCAATGGTCAATGGTTGTCCGAGAAAGATTATGTTGCTGGTCAAGTT  AATTACACTCGATGTTCACCTCCTCCCCCTGATTACAGTATCTATGAATA  TACAATTAAGGATATTCATCAGGAAATCGACATTTCCTTAGCTGACCATC  AACACAAGGCCATTCTATTGGTCAATGTGGCCACTTACTGAGGTTATACC  CATCACTATATCGGAATGAATGCACTTCTCGAACGTTTTGGACATGATCA  GTTAACTATAATCGGTTTCCCATGTAATCAATTTGGAATGCAAGAACCAG  GATCAGGGGCAAAGGAAATTCTCAACGGAATCAAATATGTCCGACCAGGT  AAAGGTTTCGTTCCTGATTTTAATTTAACCATGAAAATTGACGTTAACGG  GGGCAGTGAACACCCGCTCTGGTCATTCTTGAAACGCTCGTGTCCATCGA  CTAAGATTAACTTTAATAGTCAATCGCAATTATTTTATTCACCTTTCAAT  GAACGAGACGTTAAATGGAATTGGGAAAAGTTTCTTATCGAACCTGAAAC  TGGACTAATCTACCAACGCTACGATGCCTCGATCGATCCTTATTATATTG  CCGATGATATTGAATATCTTCTAACACGAAGATCAACA  >Unigene19520_All Glutathione peroxidase 3, isoform CRA_a [Rattus norvegicus]  GGCCACGCTCCCCGTGTCAGGGGGCGGCATCGCCATGAAGGAGGGGCCCGAAGCCTGCGTGGGCGGACCTCCCTTGAGCCTGTCTGAGGGGCCAGCCTTTAGTGCATTCAGGCTGAGGCCCCCGGCCAGGGATGCCACCCCACTCCCTCGGGGGGTGTGTCCTCTCCCCTCACCCTGACCTGCTGGCATGAGGCTCACCCCTGTCTGCCTAGTAAAGGCCTTTCTGCAGCA  >Unigene21905_All Toll-like receptor [Tachypleus tridentatus]  AGAACCTTTTCTCAGGTTCAGGACGAAAATTGAAACGTCTTTACCTGATGAGAAACTCAATTGAATCTCTTCATCCATTTGCATTTACGAATCTCAAAGAGCTGGAAGTCCTTGACCTATTTAGCAATAAGATTCAATTATTACCTGATTCACTTCTCAAGGATACAAGTAAATTGAAACAATTAAGAATCAAAGGAAACAATTTCATCACTTTACCATTGAATCTCTTCACCTCTAC  >Unigene4137_All Similar to Furin-like protease 1, isoforms 1/1-X/2 precursor (Furin-1) (Kex2-like)  ATTTTTCAATTTTCCAATTGATTTAAGACGAAGAAAATTAGATGAAGATC  TTGGTTCCTCCGAGGGAATGAATCCATCATAGAATCGGGTGGGTTTTCGC  TTGTTAAACCAAGAACAACAGCAGCAATTAAATAGATTTGGAACTCTTTC  AATTATTATATAGCAAGCGAATGAGAAAGTGATAATAAAAAGGAAGACAA  TGATTCCAGTGACAACAGAATAATATAATTCCAGTGAATAAATTTCATCT  CTCAAATCTTTTTGTTCACATTTTCCTTGTTCGTTGAGAATTGAATCACC  ATAACAAGAAGTACAATTTATATCGTTGGGACCATTACAAGTTAAACAAC  TATAATAACAATTAGCGCAGATGAATGATTGATCATTTGAATGGTTCGGT  GAGATTTTTGATTCTTTAAAATAGCCGAATGGACAATTAGAGACACAATC  AGATTGGAATAGAATATAATTTGGAAAACAATCTAAACACCCAGAGTGAC  CTTTATTCCTG  >Unigene9041_All Segment polarity protein dishevelled homolog DVL-3  CTTCATAATTGATCCAACATAAATACCACTATTTTCAGGATCACCGAATT  GACCAATTAAACTCATACCAAGAAAATTGTTTTCATCTAAATCAAGAATA  ATCGTTTTAATTGAACGTTCAATGGCAGATGATTCAGAAACACTGGAAAT  GGAAGATGAATTGGACATGACTCTCTCTCGTCTTGGATTATAACGGCTAA  CAGAGGGTTCATGGAGATCTTTGAGCTTTTATCATCGTCCATATAATCAT  CAGATATACTAGAATAATGAGATGAACCATGATGATGGTGAGATCGATGG  TAAAGTCCACGATGATTACGACGATGACTACTACGTCCGTCGGAACAATT  AGTTACATCATTAGCTTGAGATTCAATGTAACAAATAATGTTACCTTTGA  AAACATGGAGTTTGGCATTATCATCACTTATTTCCTCTTTAACTACACCA  ACATCAGCATCTTCTGTCTTAAAGAAAAACTTATATTTACAATTTGATTT  AAGATTAAGAGCAGAT  >Unigene9083_All Derlin-1; AltNam  GTTTTTTTTTGTTTGTTTCTTGTTTCATCTTAATTTTGATTAACTGATTT  TGGACACAATTTTGCCTTTCATTCATCATGTCAGAGATAATTCAATGGTT  TGATAGTTTACCAAGATTTACTAGATGTTGGCTTGGGTTGTCTGTCGCTT  TTCCCATCGTCGGAAAATTTGGTCTCTTCTCGCCAATGTCTGTCATGTTG  ACTCCACAATTTATTACTCAACTTCAGCTTTGGAGGCCCTTTACAGCTAC  ATTTTATTACCCAACGGGATTTCATTACCTTATGAATCTCTATTTTCTTT  ACCATTACTCATTACGACTTGAAACAGGTCCCGAGTTTGCATCGAAACCA  GCTGATTATCTTTTCTTATTGATATTCAATTGGATTTGGTCTGTAATATT  TGGTTATTTTTTCAGTATTTACCTTCTTATGGATTCAATGGTTTTATCTG  TTCTTTATATTTGGTGTCAATTTAACAAGGAGCAAATTGTTTCATTTTGG  TTCGGTATCAGAGTAAAAGCAATGTACTTACCATGGGTTCTACTCGGATT  TAATATGCTCATGGGTGGAAGTGTAATGTTCGAGATCATTGGTATAGTAA  TTGGTCATCTTTACTATTATACGATGGTCAAATATCCTGCTGA  >Unigene20098_All Protocadherin 15 [Tribolium castaneum]  TGGACGTTTACGATATGCTATTTTTCACGTTTCCAATCTTGGACGAGAAAGATTCCGAATCGATTCATCGACTGGACAAATTGAGGCCATCGGTAAATTAGTCGCTGGTGAACAATATAGTTTAACGATCTCTGTAACTGATTCTAGTGGTAAATCGACCCAAGGTATACTCGAGGTTGTCGTTGTTCGCGGTCCAAATNNNNNNNNNNNNNNNNNNNNNNNNNNNNNNNNNNNNNNNNNNNNNGAAATTTCTGAAGGAATTTCCGTTGGATCAACAGTCATTACACTAAAAGCGGTCGATCCCGAAAATGACC  >Unigene21825_All AGAP002311-PA [Anopheles gambiae str. PEST]  CTTCTATTTACATGCAAATATCTTGCCTGGAGAAACGATCTGGAGTTAAGAAATCAATTCCAATCCTGGTTAAAGTGATGGATATTAATGACAATCCACCAATATTTAAATCTAATCATTATGCTGTCAATGTATCAGAGTTAACTCCCATTGGAAGTACAATTTTCAAAGATCTGGAAGCGACCGATTCAGATTCTGGTAACAATGGACTAGTTGAATACTTAATCCAACCAT  >Unigene20149_All GA18508 [Drosophila pseudoobscura pseudoobscura]  GCGGGGTGGAAGAAAAATCCAAAGGCGATTATTTTTAGTACACATTCAACGGTGAAAAGGGCAAGGAAAAAATATTCAATCTTCTCGAGTAATTGATTAGTTGAATTTGTATCATGGGCTGGAAATGGTGTTTGTGCTCCTAAAGCGACACAATTTAAACAAATAGTGATTAAAATTAACCATTCAAAGGGTTTCCATTCAACCACTGNNNNNNNNNNNNNNNNNNNNNNNNNNNNNNNNNNNNNNNNNNNNNCTAAGGTGAATAGGTAAAGTGATCGAGGTGCTTTTTCTGGTAATTTTGGTACTTTTCGAGCGGGTTTTCGGGAACTAGTTGTTGCTGTTGTTGCGGCTGCAATTGTTGTCGGCGGTGGACCAGCTCCTGGTATTGTTACTGGTGTTACACCCACACCACCACCGACAACACTCACTCCGGTTGGTACACTACCGGTTGTTGTTGTTGCTGTTGTTGTTGTTGCCGCTGTTGCTGTTAATCCGATTGGTGTTATCGGTGTAACTGGGGGATAATTATGGTCCATGGTTGGTTTTAAATACTGGCCAATGAAATGGGTCAAAGTGAAAAGGGTTTTAATTTAAAAATTCACATTTGAATGGAAACAATTAACTGATTTAGTAAATGATATGTTGATTGTTAGAGTGGTGATGTTGTTGTTTCACTGGTTAGAAATGTGTTAAAGGCGAAATGAGATGTTTCTGTTACTATTTCAGTGAATGATGATGAAGATG  >Unigene26336_All Protein FAM91A1 Family with sequence similarity 91, member A1 [Danio rerio]  CAGCGGTAAAATTTGGTAAAGTGTCATAACTCCTCTCGTTTTCCAATAATTCATGAATCATTTGAATATAATATTGAAACGGTGTTATCCTCATACCATAGATCAAATAATCGGATAGATGATANNNNNNNNNNNATGTTCACGACTGTAACGTAACAGCTCAAGGTAATAACCTTTTTCACTACGACGAACCGTTTTCACCAAATTGTTGATGAACCTCAGCTGATTTTTAATACTATATTCCAGGATATTGTGATCGTAATCAGTTTTGGTTCCAATGATCATCTTAACCTCATTGTCGAGCTCATCCCAGGGAACATTATCACGAACGTATTTCTCAATCTCTTCAGACATTTCAAATGTCGATGATAATCTTTCACTAGTTAATCATGTATCAAGTCTAATTACTCAGTGAACTTGAATGATTCAGGTAAATCACACCGATGGAAACATAACTGAAAAAAAAGGTAAAAATAGACAAATGAAAGAGAGGAAAACAAATATAGAGATAATTGTGAACACGTTTGGTTGATTGTTCTCAGTGTTTACATATGGACAATCGGGTGAAGGG  >Unigene26489_All Hypothetical protein CBG07909 [Caenorhabditis briggsae]  GCTCGGAGCTTTAAGAGTAGCCAGTTCATGTTGTTTGCTTCTTCGATACTCTGAATGTTACCTCAAGTGATCTCGGCGTTGAGTAGATCAAGTAATTATGAAACCATTTGACTTTCAATTGGTAGTTGTTGATTTAAAAGAGACAAATAATATTGAAGTTCACTGTGTTGAGTGATCAGAATACCTTCTCCTTCTTTATCATATTGAGGTCGACCAGCTCGACCGAGCATTTGCGCTCCAAGTTCAGTCCATCTTTCTTTATGAGGATTGTAAATTTGAGTTCCTTTGATGA  >Unigene3959_All Succinate dehydrogenase/fumarate reductase, flavoprotein subunit  CTGCTGAACAATTGGCAACTCGATTACCTGGTATTTCAGAAACAGCGATG  ATCTTTGCTGGTGTCGATGTAACCAAAGAACCTATTCCCGTTCTCCCAAC  CGTTCACTATAATATGGGTGGCATTCCAACTAATTATAAAGGTCAAGTGA  TAAACTATGATGAGAAAAACGGTGATCGAGTAATACCTGGTCTATATGCC  GCTGGAGAGGCCGCTTGTGCCTCAGTACATGGAGCAAACAGATTAGGTGC  CAATTCCTTATTGGATTTGGTTGTTTTCGGACGAGCCTGTGCAAAGACCA  TTGCCGAAGAAAATAAACCAGGAGAATCGATC  >Unigene6496_All Voltage-dependent p/q type calcium channel [Aedes aegypti]  CTTCGATTGGTCTCAGGGGTTCCTAGTCTTCAGGTTGTGCTTAATTCAAT  CATCAAAGCGATGTTACCGTTATTTCATATTGCACTTCTTGTGATCTTCG  TAATTGTCATCTATGCAATTATTGGCCTCGAAATGTTCAACGGAGTCTTA  CATAAGACCTGCTTCGATAATATAACAGGTGAGATGATGGAGGAACCCAC  GCCCTGCGGGGAAGAGGCCTGGAACTGTGATGACGAGGATCTGGAGGGAC  ATTATGTTTGTCGGGCTTATTGGGCTGGACCCAATTTTGGAATAACCAAT  TTCGACAATTTCGGACTTGCCATGTTAACGGTTTTCCAATGTATCACCAA  TGAAGGTTGGACTCAGGTCATGTATTGGATGAACGATTCAATTGGTAATA  CATTTCCTTGGATGTATTTTATCAGTCTCGTTATTCTGGGCTCATTTTTT  GTCATGAATCTTGTCCTTGGTGTTCTCAGTGGAGAATTTTCAAAAGAAAG  AGAAA  >Unigene8865_All AGAP011116-PA [Anopheles gambiae str. PEST]  CACAGATTTTCCTTCCCAGTTTAGAGGGAAACATGGCTGATCGAAATGAG  TTCGGTAAAATGTTGGACTGGAGTCATATTGCCGCTGCTGCTTTCAAGGG  TCTATTTGCTTACGTTGGGTTTTTAACCTTCGGTGAAGCGACTCAGAAAG  AGATCACCAACAATTTACCAACCCATGGATTTAAGGCAATAATCAACATT  ATCCTTGTAGTTAAGGCTTTACTTTCGTACCCGT  >Unigene31647_All AGAP005522-PA [Anopheles gambiae str. PEST]  TGTGGTTTTATTTTCAAGTGATTATGGTTAACGGAAAAATTGTGATTTCTTGTTTGATAGTCTACCAGCGGGAACAATTCCTTTGGTTCTTAATCGTCTAACAGCTTCTCGTAGATGTTTAGGTTTAAGTGGTCCAGTTTCACCACATTTTTCTAATACATCTAAAGCCTCTTCAACTACTTCACCGGCGAAAACTTTGGCAACACCAGAAATTGCAATAACCACATTCTGAGAAACGGAACAACCAGTAATCGATTGCATCAATCTTTTCACCGAAGCTTTTGGGAATGAAGCTCGACGGAACATTTCATATCGATTAAGTTGTTCTTCACTAAAATTGGAGACCAAGACTTGCATTTTCTCACGTTCTTCCTGGTGAAGATCCTCTTGAGATTCTTCTTGTAAAACC  >Unigene1485_All Type IIA topoisomerase(DNA gyrase/topo II, topoisomerase IV),B subunit  CAGTAAATGGCCGAAAATCATGGTTGGAACAAACATTTTCTCATCTTTAT  GTTCCACAACGGGAATACCTTTACCATTATTGTAAATGACAATTTTATTC  TCTTCCGGCTTTATATCGACCCTTAGAACATCCATTCCAGGATCTCGTTG  TTTATTGTCTGCAGCATTAACCAAAATTTCATCGAAAATTTTGTATAATC  CAGGTACAAAGGTGATTTTCTTGAAAACCATGCCACTCTCTTCTCCCTCA  TAGACCCACATCTCTTGCTCCTGTGGCTCAGCTGAGCCAATATAAGTATC  AGGTCGTAGTAAGACATGTTCAAGCGGTGTCTTTTTCTGATAAATTTTCT  CCACGGTTAACCGGGAGTCACCACCAGATCTGTCACCCATAATTCAATAT  AATAAAAGTATGAGAATAGAAAAAAAAAGCCAGAAC  >Unigene1594_All FOG: Zn-finger  CTTTAAGATTACCCTTAGTGGTAAAGGGTCGACTACAAATTTGACATTTA  AAGGGCTTATCACCGGTGTGGGTTCGATGGTGGATTTGTAATGCACTGTG  ACTTGAAAAGGGTTTATTGCATTTCTCACAAACATGTTTAGTGCTATTTA  ATGAAGATCGAGATGAAGATGAGGTTGATGATGAATTTGTTTTGGCTTCA  CCACCTTCACCATTCGGTGTGGCCACCGAGGATGATGAATGTCGATTATG  ATGATGATGATGAGAATGATTTGAATGGTG  >Unigene17733_All Fibroblast growth factor 17 b [Danio rerio]  GTTGGTGAAGAGAAAAAAAAAGTTCTGATGATATGATTTTGATGATATGATGATGAGAAGGATGAAAATGTTGTTAATTATTTAATCCAATTTACTAATTANNNNNNNNNNNNNNNNNNNNNNNNNNNCATAACAGTGTTGACCGATATGTTGACGTCTAGATCCATTTCGTTGGGGCTCATGTCGGTTTATTGTCCCTCGAATGGGTCGACCTTTATAATCGAAACCAAGGAACCAATTATCGTTGTAGAATGAGCGATATTTGTATTTATTATATTTAGAGATCTCTCGGAATATGCAGGAAATATCCCACTGACCATTGTCCTTTGCAACTATTTTGCCCTTCTTGTTGTAGCAGATAAACTTTCCGGTCATTAAGCCTTTAAAATGGACCAACTGACCATGACCTTTAACACCTCGGATTCCTATTCCGGTTACATAAAATGCATTGTCAATTAGATGTTGAGTTTTTGTTGCTCGTACAGTTTTACCTGATATGTGAATATGATTTTTACTGCAACTTGATTGTAGGTAAATTTTTCTGGATCGATGAACTTTAATTGTGTGACATTTTGATTGTTGGGATTGATTTAATTGTTGTAGTTGTTGTTGTTGTTGGTGTTGTTGGTGTTGTTGTTGATTACGGTGTTTCTCATGTCGTCGTCTATGGTGATGTTGACTTGAAGGTGGGTCAAGTTGAGGTGAAAGTTGAGAGTTGATTGGTATTAATCCATTGGATGAATTGAGATACACTGAATTTGATATGTTTTGATCATAATTAATTGGTAATTGATTAGGTTTTACTAATTGTGATGATGATATTGGTGGTGATGACAATTTATTAATT  >Unigene14312_All Cellobiohydrolase A (1,4-beta-cellobiosidase A)  AATTGTTCGGGAATTTTTTTTCTCTTTTTTCTTTCAATAGGTTGACCATT  GGAGTTTTCCAATTAGAGTTAATTGTTATTATCGATTATCATCCGATAAT  ATCACTGACCACACTTTCCACTCCTGATGTGATCTCTTCCAAAAGTCCAG  TTCCATGTTCCATGAATTCATCATGTTCGATGAGACTTAGAATTAGCCTT  TGAACTGAATCAATTTCCTCGGCTGTTGATTGTTCATTGTGCGATTGTTG  TAATTGAACCGCTTTCAAAATCATTTGTAATTTAATTGCTTGTTGAATTT  CTGGTGGCTGAATAATCATTATTCTGTATATTCTTTGTAGCTGTTCAATT  GTGCTCGATTGTGATATTTCTCGTCTTAATTGTAAAATTATCAAGTCTTC  TGTTTGATCGCCAGTTAATTGTTGATTGGTTACTTTTAATTGATTTCGTA  AAGCATTTAGTAGATTCATCAAGGATTGACTGGTTCGATGGTGAGTCGTG  GCTTGACCTTCATGCGCTGATTTGATTCGATATTGAGCCTCAGCTGATGA  TGAACTTTCTAATTGTTGATTATTTAATTTCTTTCTTTCTAAAGTTTCAT  CTTGTTCTGATTGTTGGCTATTTGTTTTTGATTTTGATGATTCAATTGCT  TGAGGTTGATCTGCAGTTAAAACTGATTGGATTTGAGATGAAGATGAGGA  TGAGGATGAGCTCGAACTTTTTGATTCTGATTGAGATAAATTCACTTGTT  TTTGATCCTGTGATCTTCGTTGAGCTGCTTGAGACGAGGAATCATCCTGG  TTACCTTGAACATTTACCTGTGGAGATGAAGCTGATCGAGAGGGATTGAA  TGGACTTTGTCCAAATGTTTCCAATTGTTTTCGTTGGTTATTAGTCGATT  GTGATTCCCCTGAAGATGCGAACTGTCCTTTTAAGATTTGACTCAAAGTT  AATGGACTTTGAAGTGATTCTGAGTGAGATGCAGCTGAAGACGACGAATC  TTGAGATGATGATATTGCAGAAGAAGGTGTTTGTTGTTGATTTATATCGA  ATTTATCAA  >Unigene6445_All Glutamate dehydrogenase [Litopenaeus vannamei]  CCCGCCAGCCATTGATCATGACCCATTCACCGTTGTCCCGTTTAAGTGGA  AATCCAACTTCAAGAACAGCATGGCAAGGACCGATGATGTGAAGATAACC  ACGAACACGTCTTCGCTTTTCCTCTTCCGACAATTTACTTTTCCATTCTT  CAACCAATTTATCTTCGACTACTTGCCAACCTTTGTGGTAGAAAAACTCG  ACCATATGGAAGAACGATGGATCAGGACTATCTTCAACATAGCGAACATG  TTCAGGAATCTGATGAGGTTCGTTAATATTGGTTGAGGCTCCAGTAGAGT  AACAAGCTTTGCGCAAGGCTTGGCACAAAGGCCTCAAAGAAGATGATTTA  AGCAATGGAAATCCAGCTGACCCGAGCATTCTGATGATTTCTCTCTG  >Unigene19566_All Zinc finger protein [Ciona intestinalis]  GGATCTTTTCGCTGATTTCACTTCGACATAATGGACACAATTCCAACGATTTTGAACAATCTTCGCAAGTACAAACATGACCACAGGGAATCAGGATGATTTTGCTCTGTTCGTCCAAACAAATCACACACTCAATGAGCTGATATAACTTCATCGGCGGAGCACTTGGTTTATGATCAACTGATGAAGTACTTGGTGGACTTGATTGTTTCGTTGGTAAACAAATATCGATGTGTTTTCTAATTATTGGAATATGTTCCTCTTTGATCCCAAGTGATTTAAGTTGATCATTGGTTGAATCTGCTAATTGTTGAATCGA  >Unigene11465_All UV excision repair protein RAD23 homolog A [Salmo salar]  AATATTCAGGGAATCCCATAGCTTTCAGCCGATCAATGGCTTCTTTATCT  GCGGGAGTTACTTCAACTGAACCAACCAGTTGATCGATTCCTCTAACACC  ACCACCGCCACTTGTTGGATTGCCTATAGTTGGTGGAACAGCCCCAGTAG  CTTGTCCACCAGGCTCATCATTGAGCATACGAACAAAAGTTTCTTGATTA  CGGTTAATTAGATCGTATAGAGTGGGATTAGTTTCACGAATTTGTTGCAT  TAAACTGTAAAGAGATAAAGGATCGGCTTGAATCATTTGTCTCATCTGTA  AAAACATTGGCTGAGTACGAAGGAAAGCCAATGGATTAGAATCAGATGAA  GCAACGTGTATATTTTCAGTCCTTGGTGTGGGTCCACTGGATTCAATTCC  TCCGACTGGAACATTAATATCATTTCCAGCGACATCCAATGGTAATCCAT  TAATTAAATATTCGACAGCTCTTTCCGGATTATTGAAACTGGCTTTAAGT  GCTAATTCCACCTCAGGGCGATTATAACCCATCTCCATAATCTGTTTAAT  CATATCTTCAAATTGGTCTCCTAAAACTAAATTTGATTCGGCAGAGGGAG  TAGATACATTTCCTTCTTTGCCACTTGTTTCCTTTTTAACTGAGGTTTTA  CTCGCTTCTGTCGATGTACCAGTTGAAGCTGCACCACTAGTACCAGAGGC  ACCGATAGCTGGTGTAGAAGTGGATTTGTTTGGAGCACTTGTAGTGGCAC  TTGGTGTCGATTCTGTTGTTGGAGCCGCTTTGGGTTGGAGAACCATCAAG  ACAACATATTTGGCCTCATCGATTTCACATCCACTAATCTTACTATCATC  ACTCAAAATTTTCCCTGCATAAATCAATTTCTGATTAGCCACTGCGAATG  TTGGTCCTTTCGTTTCTTCAATTTTCTTCTTCAAATCAGCGACCTTTTCT  TCAGGATCAATCTCAATTTTGAATGTTTGCTGGTTCAATGTCCTCAAAGT  AACCAACATCTTGAAGCATGTAATAAAAATGGTTAAACAATCAGGCTCTG  GAATATAAGAGAAATTAAAGAAAAATAATAACGATAAACAAAACTATTCA  CAAGAAAATGAAGAAAATTAAACT  >Unigene12635_All 5-3 exoribonuclease 2-like [Saccoglossus kowalevskii]  TGGACTTGGAACGTTTTATAGATGATTTTGTTTTCCTTTGTTTTTTCGTT  GGTAACGATTTTCTTCCTCATTTACCTTCACTGGAAATACGAGAAGGTGC  AATCGACCGATTAGTTAGAATTTATCACGATATAAATGAAAAATTTTCTG  ATATAGAAGTTTATCTAACTAAAAATGGTATAGTTAATATGAAGAGATGT  CAATTGATTCTAAAACAATTGGGTCGTGCTGAGGATGAAATTTTCCAAAG  AAGAGCAAATAATGATGCTCGATGGCGAAATCGAGTGAAAGAAAAAAACA  AACAAAGAATTGGCTCGAGCTAAAGAAGCTGAAAAGAGGCGGGAAGAGGC  TTCTTCTGGTTACTTATGGTTAACACCTCAAGCTATTACCGGAACCGATC  AGACCACCGATAATATGTCAAGAATTAAAGCTTTCAAATCAATGTTGGTC  ACCGACGAAGCAAAAAATTCAACTCAACAGAATCAAACCAATAAAGGACC  ACAAAAAAGACGCTTTGATGATGTTGGCGGTAAAGAATCATCGTCATCAT  CTGATAATGAACCTGATGATGATATCTTATTAGGCTCTGAAGGTTGGAAA  GATCGTTATTATTTCCATAAATTTGATGCACCAAAACCCGATGGTATAAG  TCGTATCATCTCCCGTGAATATGTACTTGGCCTTTGTTGGGTCTTATTAT  ACTATTATCAAGGTTGCCCCGATTGGCAGTGGTTTTATCCTTTTCATTAT  GCTCCATTTGCATCTGATTTTGTTGACATCGATGAGATGGAAATCAAATT  CAACAGAAAATCTAGACCATTTAAACCTCTAGAACAACTTATGAGTGTTT  TTCCCGCTGCTTCGTCAAGCAATTTACCAATTTCTTGGAGGAAATTGATG  ACTAATCCTACCTCCAATATTATCGACTTTTATCCCGACAATTTTAAGAT  CGATTTAAATGGTAAAATGCAAGAATGGCAAGGAGTCGCTCTTTTACCTT  TTATCGATGAATCTAGAC  >Unigene12752_All Similar to IGF-II mRNA-binding protein CG1691-PA, isoform A [Apis mellifera]  TGGCTTGAAGACAATTGTTGATCATTGGGAGGTGTTTGTCTTGATTTTTC  ATCTTTAGGAGGCTGAGCAACCTTAATACTTGCACCTGAAGAGTTTATCA  TATCTCGAATGGTACTACCTCCAGTACCAATTATGGCACCGACAGCGGCA  TTAGGTATATACAAGTATACAGTTTCCTTGGTTTGCTCTGATGTCAAAGT  TGATAAAGGTGATCCACCGGGACCTGGGGCTAATGGCAATTGAGGAGGAG  GTGGTGTACCATAAGGCATAACGGGAGGTATGTAAGATGGGCTACCATAG  AGACCGTATGATGAGGGCCCAGGTCCGGTTGGAGCAGTTGCTGGATGATG  AGGACCTAATGGATGATGATGATGATGATGACCTGGACCACCAGTGTTAT  GATGATGTCCAGTTCCACCACCACC  >Unigene13824_All TATA-binding protein [Eptatretus stoutii]  CGAGTGAAATATACAAAATTAATCAATAAACTTAATTTGATTATGATAAA  AGTTATTAATTGATGGATGGTGAATATTTTTCATTGTTTCCTAAATCCCT  TGAGAATTGGATAAATATTGTCAAAGGCTTCATAAATTTCGGATCTGACT  TTGGCTCCAGTTAATACAACTTTCCCTGAGACAAAAATGAGTAAGACAAT  TCTGGGTTTGACCATTCGGTAAATTAAACCAGGAAATAGTTCGGGTTCAT  AGGAAGAGAACTGACTGTGTGTAAGGACGAGACCCTCTAAACGAATTGGA  AATTTAACATCGCAACTACCCACCATGTTCTGAATTTTAAAATCCAAAAA  TTTGGCATCGAAGCCTAATTTTTGTACAATACGAGCGTATTTACGAGCTG  CCAGTCGAGATTGTTCTTCACT  >Unigene14157_All Similar to DnaJ homolog subfamily A member 1 [Tribolium castaneum]  AAGTTACATCTTCGACCAGATCACCTGTAGGAAGGTTAACAACGGGTCGA  GGTGGAAGTAGAGGTTCTAACTGAGGTACTAGGGATGGATCTAATTTCTC  AGGGAATTTTACATTAAATTGAATAATGAGTCGCCCCTTTTCATATGGAT  TCTTCCAATGAGGCATACCCTCACTCATGATACATTTAATTGCCCCATGT  TTAATTACTTCTCCAGGTAAAGATGTGATAACGAGGGTTCTATCATCCAA  GGTTTTGATTCCCTTTTGCATTCCACAAAGAGCCTCAGTTAATGAAATTT  CAAGTACCATAATGAGATCATCACTCGAAGAGCGAGATCTCTTGAACGTT  GAATGCTCTTGTTCGTCTATGATTACAATTAGATCACCAGGTTGGTCGAT  TCCTGGTTCCATATCACCTTCTCCAGAGAAAACTAACCTTTGACCATCTT  CCATACCTTTATCAATGTGAACCTCAATAATCTTTTTTGCTTTCACGTAT  CTTTGACCATCACAAGATTTACATTTATCTTTTGGATTTAATATTTCACC  AGCCCCCGAACAAGTATGACATATAGTTTGTACTTGCTGAACAATATTAG  GACCCATTGCTTGCACTCTAACCTGTCTACCAGAACCATGACATTTTTCA  CATGTTTCATAACCTTTTTT  >Unigene19429_All Tyrosine-protein kinase [Culex quinquefasciatus]  CGCCTCTCGATTCAAAGACGTTGCTGGTTTGGTTCACCATGATGATACATTTTACTGGATCTCAGGAACTGAATTGTGCAAGGAAGAGGTTAATCCAGATGGAGAGGAGATTTATCATAATTCATTCGCTTTGGATGACAAATTAATCTCCCTTGTTTTGGTCCATCCAACGGTTCAACCGATGCCCTTTGTCCACTCAACTGCTGACCATGAGATCTTGATTATTCATCCTTCATCTTCAGCCGATTTCAAGACTCTTTGTTTGACTATTCTTGGATCATTAATGTTTTTTATTATTTTGCTTTTTGTTGCCAGAAAAGTCATGAAATCGCAAACTAATGAGAAAGAAGTTCTCAAGGAATCTGAACGAGGACATGAACGAAATCTAATCAGCTTGACCCAACTTCCAGATCATCCCCATGAAGACAATAGACTCTATCTACCAGGAGATACTGGAATAGAAAATGAATTGTCGTCCATCAGATTAATTTCACCAAGCCAATTCAAACAAACAGATCGTTTGGGTTGTGGAGCTTTTGGTCAAGTATTTCGTGGCTTGTTCACTGATCCTGAGCTTGTCGGAAGCCTCGATGCTTCATTGCCCGTTGCGATAAAGAAATTAAAATCAGATGCAGTAATTCTCTTTAATTCTAATGGCTGATCAATTATACCATTAATAACACTAATTGAGTTGATTTTTTCACAGAAATTAAAATCAGATGCATCAGAACAAGAAAAAGATGATTTCATGAAAGAGGCTAAAATCATGGGGAACTTCAAACATCCACATATTCTTAAAGTTCTTGGTGTTTGTCTTGATCCGGGAAATAATTCCATTTTATTAGAGCTCATGGAGGGAGGCGATCTTCTTTCGTATCTTCGAGATGAGAGACCGACAGAAAAGAAACAGTGTGATTTGACTTTAGATGATCTTCTCAGTATCTGTGTGGATGTTGCTAAAGGTTGCCAATATTTGGAAGCGATGCACTTTGTTCACAGAGATTTGGCCGCTCGAAATTGTCTTGTTTCGAGCAAAAACCGTGAATCAAGAATCGTTAAAATCGGTGACTTTGGTCTAGCTCGAGATATTTATAAAAATGATTATTATCGTAAAGAGGGCGAGGGTTTGCTGCCAGTTCGATGGATGGCCCCGGAAAGTTTAGTTTACGCCATTTTCACGACCCAATCGGATGTTTGGTCATTCGGTGTTCTACTTTGGGAAGTTATGACTTTAGGTCAACAACCATACCCAGCTCGTAGTAATCAAGAAGTATTGAACCATGTTCGAAGTGGTGGTCGACCTGAAAGGCCACCAAACTGTTTAGAGGAGATGTTCCTTTTGATGAATCAATGCTGGTGTTACAATTCAGACATGAGACCAACATTTATGTCTTGTCTCAATTATCTGGAGGAATTAAAACTTAAATTGTCATCAGGAGACGGAATCATCACAAGTTTTTCAAACCACAGCTACTACACCAACGGTAAATCAGTTGCTTGTTTGTTAATTTACTTTTGCACCATCAAGATTTTATTGAATCCAATCTCCATCTCTCTCTCTTTAATTAATCGTCTTGATTATCGCACTTTCTTTTCTTTTTTTT  >Unigene19975_All Huntingtin [Saccoglossus kowalevskii  AAATCATTGGAAGCTTTGAAAGTACTCCAAGCATCAATTGACAAACCTGAAGAACCGTTGATATCAACAAAATTTATCAAGAAGGAAGCGTCTATCAGTAAGAAGGAGAAAATCTCTCACTGTATCAACATTGCGGATATAATATGTTGCTCTCAATTAAGGAGTATGGAGGATTTNNNNNNNNNNNNNNNNNNNNNCAATTGAAACCATGTTGGCTTGTTGCGATGATACTGATGCTGATGTCCGTTCCAATGCCGGTGAATGTTTAAATAGGACCATTAAATCAAATATCGATTGTCACCTTAATCGGATTCATGTTGAATTATACAAAGAATTGAAGAAAAATGGTTCAAGTCGAAGTATTCGTGCAGCTCTAACCCGTTTCGCTGAACTTGCTTATCTTCTTAAACCGGCCAAATGTCGTCCTTATTTGGTACAATTAATTCCAGTATTAGAAAAGATTAGTGCACGAACGGGAGAGGATAATATTCAAGAGACAATCACCTTAGCAATGAAAAAGTTAATGCCAGTCTTGGGTCGATATGCAACCGAAAATGAGATCTTACGTCTTCTTAATTGTTTCCTGTCCAATTTAAGTTATTCTCTGGCCTCTGTTCGTCGAACTGCTGCAATATCATTAACTTCGATATGTCAACATTCAAAGAAACCACCTTACTTTTATTCTTGGCTAATTAACAATCTTCTTAAATTATTTCACTCCAATCAAAACAAAC  >Unigene21663_All Cyclic nucleotide gated channel alpha 3-like [Saccoglossus kowalevskii]  GTCATCTTTGACTTTTTAATCGGTGTCCTTATTTTTGCCACCATTGTGGGTAATGTGGGTTCCATGATAACCAATATGAACGCATCTAGAGCTGAATTTCAGTCTCGAATGGACGCAATTAAACAGTACATGGAATTCAGAAAAGTTTCAAAAGAACTTGAGAAACGGGTCATCAAGTGGTTCGACTATTTATGGTCAAATAAACAATCACTCGACGAGGAAGCGG  >Unigene21919_All Thioredoxin reductase 3 isoform 1 [Rattus norvegicus]  CCTCTCCGTAAAGTTCAATCGCTTTTTCCTCTGACAACCCACAGCAACCATATTCTAACGGGGTGAAAACGGTTGTTGCGACACTTGAATAATCACATTGTTCATCACTGACACCAGCTAATCGACGAGCCAAAAGTATTCCAGCCTTGATTGCGACCGGGGTTAATTCGGGTTTGTCTTTGATAATGTCACCAACGGCGTAAATATTGGGAACATTAGTGGCTTCATTTGTACAAGG  >Unigene23398_All Novel protein similar to sine oculis homeobox homolog 1 (Drosophila) (six1, zgc:92332) [Danio rerio]  CTTTGGTTAAAGGCTCATTATAAGGAAGCGGAAAGAGTTCGAGATAAACCTTTAGGTGCTGTTGGTAAATATCGTATTCGCAGGAAATATCCGCTACCTCGAACCATTTGGGATGGAGAGGAGACATCTTATTGTTTCAAAGAGAAATCGAGAAATATTTTAAGAGAATCGTATAATATTAATCCTTATCCCTCGCCAAGGGATAAGAAGGAGCTTTCAGAAGCGACCGGATTAACGACAACTCAAGTTAGCAATTGGTTTAAGAATAGGAGGCAAAGAGATCGAGCTGCAGGATCAAAGGATGGAAATGATAAATCGGGTGGTTCTGGTTCCAAGTCTGGATTGAAAAATTTGAACGACTCATCGCAATCTGGTCATTCGAGCGACGATGAATCTCTTGGTGATAGTCCGCCGATGATTAAAAGTGAAACCCGAACTTCATCCGTATCATTAACTTCAGGTCATAACAACAATAACCTCGGCGGTGGTAA  >Unigene23547_All Similar to Phosphoglucomutase 3 [Sus scrofa]  ATAAAGTGACCTTTCAAGTTATCCAAACGATTTATTCTAATGGGAAGACATCACGATTTCTGAGAAAATATGTCGGTGTTTCGGTTGATTGTTCACTTCCTGGAATTAAAAATTTAATGAAGAAAACTGAAAATTCTGATATATCAATTTGGTTCGAGTCTAATGGACATGGGTCAGTATTTTACAACGAAAAAGCTTACAGAGTGGTCAAGAGTGCAGTCGAAACTGATTCAACTGGATCAGCTGATTTATTGATGAAAATTTTCCATTTAATGCATCAGAGTACCGGTGATGCTTTTTGTAATCTCCTTTTAATCGAGTCCATTTTGAGAGCCAAAGATTGGACAGTTGAAAATTGGGACTCCATTTATGAGACATCACCTTCTCGGTTCGTAACAGTGACAGTCAAAGATCACACTTTCATCAAAACAAACGAAACGGGCACCGTTGTCCTCGAACCCGATAAATTACAATACAAGATCAATCAGTTGGTCAACAGATTTGAAGATAGTCGAGTAATTGTGCGTCCATCGGGAACCGAGCCCGTTGTTCGAGTCTATGGTGAGTCAAGTACTCAAGAGAAAGCTGATCAATTAGTTGAACATATCGTCCAACTTGTACAAAATTATTCGAAATAATAACAAAAAAAACTTGATCGAAATCAAATTCGT  >Unigene25235_All RPB5-mediating protein-like [Saccoglossus kowalevskii]  AGCGTTTTCATTTGAAGCTTTTCACTTACATAAATCAATTTGCTTTCGTTAAATCAACTTATGTTTGTAAATAGAAAATGTTGGAAAATAAATTAACAAATAATCACCATAATTATCGTTAATTACTCTTGTTGTCTCATTAATTTCTCCCGCTCCTTGTGCAATTTAACTTTTCTTTTATGTTCTTCCCTCCATTTTGCTTCAGCTTCTTCATCATATTCCTCCATTATTTCAACCAAGTCTTTCGATTCCTCTGCTAATTGTGAGGTGAATTTGAACCAATTGTTAATTGATTCGATTTCCTTTTCGGTTTCGACTTTTCTCTCTTTGACAAACTTAATTCTTCTTTCGGCAATTTCAACGGCCTGAGAAACCGACCGGTCAGCAAACCAATTGTCACCCAATGAAACCATCACCTGATTAGTGTGGTCAAGATTTTAAAGGCCAATGGAGCGTCTTCCTTTCTCATATTGTGACAAATTGGAACCATAATTGGATGATTAACCTTTTTAACCAGCATTTCCATGTTACCTTTTAATGATTCNNNNNNNNNNNNNNNNNNNNNNNNNNNNNNNNNNNNNNNNGTTTTTGCCATTTGCAAGGCCCATTGTTGGCTGATCTTTAATTTGTCCAAAGTAAATTGAGGTTCCATTCAACAATTAGAAGAAAC  >Unigene28820_All Similar to testis enhanced gene transcript-like protein [Acyrthosiphon pisum]  GGCCAACATCAAAACCGATAAAGCCGATGACAAAGTACCACCAATGTACAACCATTTTCGCTGTTCACTGAATATGGTTGCCAAAGAAAAGCATGAAAAGATTAAGCATGTAAACAGAAATGCGCTAGGAACTATCGACGGTTCAATCATTGTCGCAATTTCAACCAACGGTCCAAGACCAACACCCATGAATAAACATGACGTAAGG  >Unigene31396_All GJ18657 [Drosophila virilis]  GCAGCAACTGGTTCACCTGTTGCACAACCAGCGACATTATCAACATTTAATGTTACAGCAGCCGGTTTAGGACATGCAACTGTACCGCCGGTTTCATTACAAAATGAACAACAGAAAGAAACTGTTTATTTGTATATACCTAATACTGCTGTCGGTGCAATTATTGGTACTGGTGGAAGTACCATTAGAGATATGATGAGTTATTCAGGGGCAACAATTAAGGTGGATCAGCCAGCTAAATATGACCAACAACAGCAATCACAAAAGCAGCTTTCTGATTCTGAAGAACAACAACAACAACAGCAACAAACACCACCACAACAAAATGAAAAGCAATATGAACAAACGGG  >Unigene32011_All Huntington disease protein homolog  CCTGAAGAACCGTTGATATCAACAAAATTTATCAAGAAGGAAGCGTCTATCAGTAAGAAGGAGAAAATCTCTCACTGTATCAACATTGCGGATATAATATGTTGCTCTCAATTAAGGAGTATGGAGGATTTTCAAAAATTTTTAGGAATTGCAATTGAAACCATGTTGGCTTGTTGCGATGATACTGATGCTGATGTCCGTTCCAATGCCGGTGAATGTTTAAATAGGACCATTAAATCAAATATCGATTGTCACCTTAATCGGATTCATGTTGAATTATACAAAGAATTGAAGAAAAATGGTTCAAGTCGAAGTATTCGTGCAGCTCTAACCCGTTTCGCTGAACTTGCTTATCTTCTTAAACCGGCCAAATGTCGTCCTTATTTGGTACAATTAATTCCAGTATTAGAAAAGATTAGTGCACGAACGGGAGAGGATAATATTCAAGAGACAATCACCTTAGCAATGAAAAAGTTAATGCCAGTCTTGGGTCGATATGCAACCGAAAATGAGATCTTACGTCTTCTTAATTGTTTCCTGTCCAATTTAAGTTATTCTCTGGCCTCTGTTCGTCGAACTGCTGCAATATCATTAACTTCGATATGTCAACATTCAAAGAAACCACCTTACTTTTATTCTTGGCTAATTAACAATCTTCTTAAATTATTTCAC  >Unigene3337_All Similar to Bromodomain-containing protein 2 (Protein RING3) (O27.1.1) [Acyrthosiphon pisum]  GGTGCAGCGGGACCAGCCAAAACACCAGCAAACCAAAAACCAGTTGGACA  ACCAGCCAAGAGACAGCGGACCAATAGCAAAGCTAATAAGAAGGTTAATA  AAGCCGTACCTGCGTTTGATAGTGAAGATGAAGACAATGCTAAGCCAATG  TCTTATGATGAAAAGAGACAGCTTAGCCTTGATATTAATAAGTTGCCAGG  TGAAAAATTAGGAAATGTTGTTCATATAATTCAATCTAGAGAGCCATCTT  TAAGGGATTCCAATCCAGACGAAATAGAAATAGACTTCGAAACTCTCAAG  CCTTCAACGCTCCGTGAATTAGAGGCTTATGTTGCCTCTTGTTTAACGAA  AAAACCCAGAAAAAGATATAC  >Unigene3338_All Spata5 protein [Mus musculus]  TCAGCACCAGAGTAACCATCAGTTTTATCGGCAAGAGTATCGATTTCATC  AATTACTTGAGAAGAAACCGCCATCTTCCCTGTTCTTATCTTTAAAATAT  CTTTTCGAGTTTCTTTATCAGGTAAAGGAACATAAATGATTACATCAAGT  CGCCCAGGTCGACGTAAGGCCATATCGATTTTATCTGGTCTATTGGTGGC  AGCGACAATTGTAACACCATCGAGTTCTTCGATTCCATCAATTTCAGTTA  ATAGTTGAGCTAAGACACGATCTCCAACCGTACAAGCTGAAGATCCATTG  GTACCACCGCCTCTTTCCGACGCTAGAGCATCTAATTCATCGAAAAATAA  AATTGACGGTGACGCTTGTTTGGCCTTTCTGAATATCTCACGAACTGCTT  TTTCCGATTCACCAACATACTTGTCGAAAAGTTCTGGTCCCTTGATAGCT  ATGAAGTTGAG  >Unigene6790_All Elongase [Culex quinquefasciatus]  ATCTGGTCAAAAATCGTTGAATTCGGTGACACAATATTCATTGTACTTCG  TCGGAAACCATTACAGTTTCTTCAAGTTTATCATCATGCGACAACTTGTA  TCTGTTGCTTTTTCTATTACTCAACGGGAACGGCCATTTGCCGATGGACA  CTTTGTATGAACTTTACTGTCCACTCGTTCATGTATTCATATTTTGCCAT  CGTTGGAATGGGCTACCGAGTACCTCGACCTTTCGCCATGATATTGACCT  CTGCCCAGTTGAGCCAAATGCTAATCGGAATGGGAATCAACATTTACCAT  ATTAACCGACTACTCGCTGGGATTCCGTGTGCCTCGTCGATCGGTTTGGC  CTCGTTCTCGATTACCGTTTACTGTTCATACGCGATTCTTTTTGCTCACA  TGTTCATGGTTAAATATTTTTCCACCAAATCGAAAATCAAATAATTATC  >Unigene25149_All Ribosomal protein S6 [Culicoides sonorensis]  GTTGGTTGCGATTTAACTGGTTTACCAGTTTTAGTTGTTGCAGCGGCGCCTGCTTTACCGGTTGGTTTTGTGGTTTTAGCGGTTGCTTCGGCCTTGGTTGATTTTGCTGATTTTGTTGCTGGTTTCGCCTCTGATTCTGATTTCTTTGCAGATGATGTCGATGATTTACGGCGACGATCGGAATCTTTCTTTTCTTTTGCTTCCTTTTGACGTGTTGCAAGTAATTTAGCATACTCAGCGGCGGCTTTCATTGAAGCTTGTTTCCTATTAATTTTAACCGCAAAACGTGCACGCTTTCTACGAAGAATCCTTGGAGTGATCAAACGTTGAATTTTAGGTGCTTTAGTTTTCGGCTTCTTTCCTTCTTTCTCTGGCATTGGCCGTCTAACAACATAGTGTCTAACATCATCTTCTTTGCTCAAATTGAACAATTTACGGATTTTGCTTGCACGTTTAGGNNNNNNNNNNNNNAGGTGATATCAGTTAAACCGGGAATTTCGTTTTCACCTTTTTGAACAATGGCTAACGCGAGAACACTAAGATTTGCGTCGACGATACAACCACGAACTGATTTACGTTACGCCTTTGACGATAACAAGAATGGCCTTTACTAAGAAGGAGGCGAACACGTTGGTTGGTCAAGACACCTTGTTTCATTGGAAAACCTTGTTTGTCGTTACCACCGGTTAT  >Unigene31875_All Similar to rap55 [Tribolium castaneum]  GCGCAACCTGGTTTATTTTTCAACCTTATTTTTTTTCTCATTTTTAGTGTGCAAAGATTTTAGAAGGGTTTTCAAATTTTTGTTTATCGATGAAAGTTTTTAATCGCCCGACAAAGACTAGGCGGCAGCAGAAGTCTCACGCTTCTTATTATTATTATCCCGCCGGTTGTTGTTGTTACCGTTATTATTGTTAGGCCCCGACCAACGTGGTTGGTATCTACGATAATTTTGTTGCCTTCCATTATAACCTCCAGCAGGCCTATAACGACCTCTCATATTACCACGACGATTAGCTGACGCACCGAATGTTTCTTCATTCACTTTACGCTCACGATTCCAATCCATCTTGTTAACATTTCCCTTTGTTCGTTCAATTGCTTCGCAACTGATTTGATCAAAGAATGATTTCTTTTTATCGTAGAACGCGTCTTCCTTTTTCTCATCACTAGCCACAGGATCTTCTGAACTCTCTTTAACTTCAGGATCGTCAATTTTAGTTGCTG  >Unigene31989_All Beta-C integrin subunit [Lytechinus variegatus]  AAGGTCAACAGGATAATCAACTGCTTGTTTGAATTCAGTGTAGAAACGTTGAGTATCATTAGGACGTAATTTAAGTGAAATCCGTTTTGGTTTAAGTTGAACCGCTGAATCTGGTGATAAAGCTTGATCTGAGAACTCTTTGGCCTCAGTTACATTGACCACACTAAGGGGAAAATGGATTGACTTGGGGTCACAACCTTCGGCGATTAAAGATTTATAAAAATCACACCGAGCTTTATTGGTCTCATCGGAGAAACCTCTCTGTGTACACCAAGCACAACTAGGATCAGCTCTTATACATTCATGGCAATTTTCACGAGAAACACATGGATTTTGTTGGTCAGAGGTTTGAGCAAACGTTAATCCAATAATTGAGAACAATAAAATGGATAATAAAATGAATAGTGAAGATGAACAATTGGTGGTTGAAGATGAAGATTTAGAAAAACAATTTAAGAAAGAAAAGAACACTACCGGTGACATGGTGATGAGAATATGTTGTTGTTCTCTGAATGTGATTAGAATTCAGAGAAGAATATGATTAAGGGTGGTGGTGATTGAGAGAGAGAAAGAGAGAAAGAAAAGGAGAGAAAAAGAAAACAAAAAGAAAATATAAAGTTTAGAAAAAAAATTGAG  >Unigene3200_All Superoxide dismutase [Fasciola gigantica]  TTATGGTTTGACAGCCAACGGCGGTTGTGATGCTGCTGGTCCACATTTCA  CTCTTTACCGTTGGAACCACGCTGGACCAACGGATAAGTATCGACACGCT  GGAGATTTGGGTAACTTGAAGGTTGAACAAAATGGCATCGCTTTTATCAA  TCAATTCAATGGTCAAGTAACTTTAATGGCCGCAAATAACACGATCCTTG  GACGGTCAATTGTGATTCATGAGTGGCCAGATGACCTTGGCCTCGGACGA  AAGGAATCAAATATCACCGGTAATGTCGGTAAACCAATTGCTTGTGGATT  AATCGGATTAAGAAGAGATAAACCTTATCTACCAATCAATTGTCCTCCTC  CAAATCTTTAATCAATTAATCAATTAATCTCTTTATCACATTTATCATTA  TTCTTCTTAAAGTGAACAATAAAGTTAAAGGTTAACAATTCGGTGC  >Unigene17265_All Similar to AGAP001957-PA [Tribolium castaneum]  TCTGTGATTTAAATTTACTCGGATCTTCACCTGACTTGAAATCCTTTGGT  GCTACAGAATCATTGGCAATATCGATGAAAACAACTTCCCGTTGTTTCAG  TTTATCATTGGTCAGTTCGTGAACATTTTTCTTTGACCCATTATCAGGAA  CATGCATCGTTTCAATGCAAATGAAAAAATTATTTTTCATATAATCAGGA  TTCGTTATAACAGTTTTACAATATGGATATGCATTCCAAGCCTCTTCGTG  GATCTCAAGTGACCCTTTAGGTGCCAGATAACGGACAAAAGGTGGTACCT  TACTTGCTAAATGATAAATCTTATAAGTGTATTGTCCTTTGGTAAATTTA  CCTCCCAAAAGTGGATGATCTTGAAATGGTTCATTCTTAATCACTTCGAC  TCCCTCTCCACCTCCGGTCTCATTTTTACTCGCCTCAGCCACCGAATACA  ATTGTCCTATTTGATACTCTTCAACAGTTAATGGAAGTGTAACTCGATAT  TCTTTGATAATCATTTCAATCTACAAACAAAATCATTT  >Unigene6795_All Myospheroid [Tribolium castaneum]  TGCTTTTGTGGCGATTCCGTAGATTGGACTTTCACCAGGATCCCATTTCG  TTAGCACTTGCCGCTCTTTTTCATACCTGGTGTACTCGCGACTATCATTG  ATTGTGACAATTAGTTTCCAAATTAGAAGGATCAATAAACCGGAGAGGAA  AATTCCAATAGTTATTCCAATTAAAATGGCGTAAATGTTGACTGGAATTT  TACAGTTCTTAGATCGTTGAGCTGCCAATTCTATTTGTTTGCCAAATTGT  TCTTCGTTAATGTAATATTTAAATGAGTACTTGCATTCATCTTCTCCGTC  AACGAACGTACAATGTATCTCACCTTGAGCTGCATCGACTTCGAGATCAT  CGACAACGAT  >Unigene12709_All Ubiquitin-conjugating enzyme domain-containing protein [Toxoplasma gondii ME49]  GGCGGCTTCGGTGACCAATGGATCATCGGCATTTGGGTCAGTTAGCAACG  ATGTTATCGAGATCATTACTTGAGAGATGGTCAATGCTGCTGACCAACGG  CCTTTCTTCAATATGTCCAAGCAAATGTTTCCTTCTTTACTTATGTTGGG  GTGGAAAATTTTGGTCTTGAATCTTACCCGCGGTGGTTCATATGGATAAT  CTTTCGGGAAGTTGATCTGCAGGAAGAAAATTCCTCCCTGATATGGTGAC  TCATCTGGTCCGGTGATCATTCCCTCCCATGAAAATAAATCGTCGCCCAC  TGGACCGGCCGAACATCCTTCCGGTGGATCTTTTTCAAACTGTTCCAATT  CCAATTTCAATCTATGAATGGACATAATTGAGTTAATCGAACAGCAGAAC  AAAAAACACAACCAAATTAGTCAACATTTAATTGTCTTTAATCGTTGATT  GTTGTTGTAAGAATGAGAGAAAGTTAATTGTTGACA  >Unigene24870_All Similar to Slc20a2 protein [Ciona intestinalis]  CGTACATGGCGGTAATGACGTAAGCAATGCCATTGGTCCATTGATTGGTTGTGTACTCATTTGGATGGACGGTAAAGTTCAAACTGGACGAGAGCAGCCATTTTATCTCTGCCGATTTACGGTGGTATCGGTATCTCGCTCGGTCTTATCATATGGGGTCGACGTGTAATAAAAACAATGGGTGAAGATTTAACCAAAATAAGTCCGTCC  >Unigene31108_All Actin 1 [Brugia malayi]  CGCCGGCGTTTCGATTGATTTAATTTAGAAGCATTTTCTGTGAACAATTGCTGGGCCTGATTCGTCATATTCTTGCTTCGAGATCCACATCTGTTGGAAGGTTGAGAGTGATGCAAGAATTGAACCACCGATCCAAACTGAGTATTTACGTTCTGGTGGTGCAATAATCTTGATCTTTAATGTTGATGGTGCAAGAGCGGTGATCTCTTTCTGCATACGATCAGCAATACCTGGGTACATGGTGGTACCACCTGAAAGAACAATATTGGCATACAAATCTTTCCTGATATCAATGTCACATTTCAT  >Unigene4356_All Similar to Aconitase 2, mitochondrial isoform 2 [Strongylocentrotus purpuratus]  GCCAAAAAAATGGTTGCTTATTTATTATCTAAGAAGAAGAAGCAGAAAAA  CTGTAACCTTGTTTTCTTGTCTCTTCAATAAATACGAGAGAGGTTATAAA  TCAATTCGTCTTCACTTGATTAACTTAATTTTAATTCCTCAATTTACCTT  AATTCTTTAAATATGGTTCTCCTCAAAAGACTCGTTTCAACAAAGGGTCT  CGACTATTTGTCGGTGAAAAGCCAAATAGCCAAATTACATTCTAGTCCAC  AATGTTTGGTGGCTATGAGTCGATTAGATAAGGAGCACATTCCTTTTGAC  AAATATAAGTCTAATGTTAGTGTTGTTAAGAGTCGATTGAATCGACCTTT  AACTTTGTCGGAGAAAATTCTTTATGGTCATTTGGATGATCCTGCTAATT  CTGAGATTGAACGAGGTCAATCTTATCTTAAACTTCGACCTGATCGTGTT  GCCATGCAAGATGCAACTGCCCAAATGGCCATGTTACAATTTATCTCCAG  TGGATTACCTCAGGTCGCTGTTCCTTCAACTATTCACTGTGATCATCTTA  TTCAAGCTCAAATTGATGGTGTCGCTGATTTGGCTCGAGCCAAAGATATT  AACAAAGAAGTTTACAATTTCTTAAGTACTGCTGGTGCCAAATACGGTGT  TGGTTTCTGGAAACCAGGATCTGGTATAATCCATCAGATTGTTTTGGAAA  ATTATGCTTTTCCCGGAGTCTTGTTGATCGGTACCGACAGTCACACTGTT  AACGGAGGTGGTCTTGGTGGTCTATGTATTGGTGTTGGAGGTGCCGATGC  CGTTGATGTTATGGCTGGAATCCCATGGGAACTTAAATGTCCAAAAGTCA  TCGGTGTTAGATTAGTTGGTAAACCAAGTGGATGGACTTCACCTAAGGAT  GTTATCCTTAAACTTGCCGGTATTTTAACAGTTAAAGGAGGAACTGGAGC  CATCGTTGAATATTTTGGTCCTGGTGTTGATGCTATTTCATGTACTGGTA  TGGGTACCATTTGTAACATGGGTGCTGAGATCGGTGCTACCACTTCCGTT  TTTCCTTATAACAGTCGAATGAGAGATTATCTGATTGCCACTGAACGTAA  AGATATTGCCGATGAAATCGACAAACACAAAGACTTTTTATCATCAGATA  AAGGTGCTGAATACGATCAAGTCATCGAAATCAATCTAAGTGAACTTGAA  CCCCATGTTAATGGACCTTATACTCCTGATCTTGCCACACCAATCTCCAA  ATTGGGTGAAACTGCTGCTAAACATGGATGGCCTATGGATGTTAAAGTTG  GACTTATTGGTTCATGTACCAACAGTAGTTATGAAGACATGACCCGTGCC  GCAAGTCTCGCTAAACAAGCAATCGATGCCGGTATCAAGCCTAAATCATT  ATTCACTGTTACTCCTGGTTCTGAGCAAATCAGAGCAACAATTGAACGTG  ATGGTCAAGCTAAAATTTTCCGAGATTTTGGAGCCGTTGTTTTGGCAAAT  GCTTGTGGTCCATGTATTGGACAATGGGACCGAAAAGATGTCAAAAAA  >Unigene21135_All Muscleblind, isoform F [Drosophila melanogaster]  GACGTTCAAAATGGCCGAGTAGTCGCTTGTTATGATTCAATTAAGGGTCGATGTAATAGAGATAAGCCACCATGTAGATACTTTCACCCACCTCAGCATTTAAAGGATCAACTATTAATCAATGGTCGTAATCATTTGGCTTCAAAAAACGTTATTCTTCAACAAATGCCCCAGCAAACCGTCCTCAATACTGGACAATTGCCTTTG  >Unigene28835_All Similar to tyramine beta hydroxylase [Tribolium castaneum]  GCCCCGCTCCGATAGCCCAAGCCGAGAGCACTTGTTTACATGCTTCCAGAATCGGTGGTTTATCCGGATCATGACAATGTCCATTCCATGAGGGCAATTCTAATTGAGGGTCAACTTGACAATGAAATATTTCCATGTGATGAACTAAAGCCTCATTACCCGATTGTACGTTGGCCTCATACTTAATTATATGGTGTTTACGTTTAAT  >Unigene18098_All Armadillo [Culex quinquefasciatus]  TTGGTCAATATAATGACCACTTATAATTACGAGAAGTTACTTTGGACAACATCTCGAGTGTTGAAAGTGCTTTCAGTTTGTTCATCCAATAAACCGGCTATTGTNNNNNNNNNNCGGGTGGAATCCAAGCCTTGGCGATGCACCTCAACTCAGAGTCTAATCGTCATGTGTCCAACTGTTTGTGGACATTACGAAATTTATCCGACGCGGCG  >Unigene11479_All U88 [Brugia malayi]  GATCAACAAGATAGTGTTTTATTTAACAACTTGAATGTAAATGAACTAGA  AACGTTTCATGAGATTGATGGTGATGTTAATCACAAGAAGAGGATAAAGT  TGCTTTCCAAGTGTAACTTTTTACTATCTCCAAATGATTCAAGATCTAAA  ACAAAAATATTACTCAATTCTGATTCAATACCAACAACAACATCAACATC  AACAGCAGCAGCAACAACAACAACACGATCAACAAGTTATGATGAGGATG  AAAATGATTCGGATGATACAACAACAACAGCACCAACAGAGTGTAAAATA  TCACATGAAATGGATGAAATATTTGGTGAGTTTGTTGAACCAAAGATCAA  TGATTACCAAATGGAAAACAAGAAAATGATGATGATGATAAATAACAATG  AAACCATAGAGAAGAAGAGGGAAGATGATGATAAGAGGGAGAGGAAAGTG  ATTTCAAA  >Unigene4229_All Merlin/moesin/ezrin/radixin [Aedes aegypti]  TTTTTTTTTAATGCCTTTGGAATGTTTTGTTTCACCTTAATACCCAATTT  CTAATAATCTTCAACTATTCCATCGATTTGAAACAAAAAAGAAAACCAAT  TTACCATGAATGGAGTATTAAGCTACTTAAAACAATATTCTCTTGACAAT  ACTGACCTGGTTATCTTGGATTACAATTTGGGTCTCATCATTTAAGAAGA  AAACAACAACCACTCATCATCTTTATCGGTTCTTATTTGCAATATATTTT  GGATCATCGGCAACCATTTGTCACCATGAAAAGTCTATTACGAGCATCTA  AAAGCAAAATGATGTCCGTTGAGGTGATCACCATGGACGCTCAATTGGAT  TTTGATATATTACATAAGGCAACAGGAGGTGATCTCTTTGAACTGGTCAC  TCGTACCTTAGGCTTGCGAGAAACGTGTTTCTTTGGTCTTCAATATGTCG  ACGTGAAAGGTTATATTGCTTGGTTAAAAATGGATAAACGAATCTGTGAT  CAAGAAGTTAGATTAACCTACAAACCATGTAACTCATCGAATTCATCTTC  ATCCAGTCTTTCATCGTCAAACAAAAACAAACCAGTGCCTCAAATGTCAT  TTCTTTTTCTTGCCAAATT  >Unigene11433_All Potassium channel [Panulirus interruptus]  TATCCGATTGGTCCGTGTCTTCCGAATATTTAAACTTTCCCGGCATTCGA  AGGGTCTTCAGATTCTTGGGATGACCTTAAAATCATCTTTACGGGAATTA  GGTCTTCTAATATTTTTCTTGCTCATTGGTATAATACTCTTCTCATCGGC  GGTCTATTATGCCGAATCGGGATCTGAAAGGTCCTATTTTAAATCCATTC  CTGATGCATTCTGGTGGGCCTTGGTTACGATGACCACTGTAGGTTATGGT  GACATGGTTCCACTTTCTTTCTGGGGTAAAATTGTGGGCTCTCTATGTGC  CATTGCCGGTGTTCTTACCCTTGCCCTTCCAGTTCCCGTTATTGTTTCCA  ATTTTACCTATTTCTATAATCGTGAAATGGTACAAGGTGATTTAGAATCA  ACAAATGAAAAATTCGTAAAGGGTTGTCCATATTATTATCTCGAGGGTCA  TCATGTTAAATGGCGAGAAACCATGGAAACGGAAACAATTTCATCCAATT  CAAGTTCAACTGTTGATTTTATCGGTCAAAGGGAAGACAGTGTTTATAAA  AGAGCCCATGGTGGATCAATTGTCAGTAAAGGTGATAATAATGATAATAA  TAACGGTGGAAATAGTTGTAAACGGAAACGGAAAGGAAAAGGTGAAAGTC  GCAAAGAATCACGTTGTAAAGCTGTTGATTGTAATGGCGAAGAGGAAGAT  GATGAGGAAGATGAAGATGATATCAATGAAGTTACCAGGAAACTAATCAC  AAGCATGGAACAGATTAAATGATCAAACTAATCATTAAAATTAACGAAAA  CGAAAGCACAATTTACATTAAAGGGAAGATAATTAAAAGAATCCATAATC  AACAAAAAGGAAACAAATGTGAAACCAGAATTATAACAATTGATGATCAA  GTAATAATACAATTTAAAAGAAAATTAAACAATTCGAC  >Unigene21880_All Voltage-gated K channel [Limulus polyphemus]  GGATAATTTAAATATACGGAAGACTCGGACCAAACGGATGACCCTCAAAATGGCCAGTGAAGCTCCTTGATTTTGTTTCTCATGAAAAGTCTTGGCAGGATCATCTTTAACCGCGAAAACGGTTCCCAAAGTGATAAAGAAAGGTACAATCGCCATGAAATCGATTGCATTCATTATATCCTTGAGAAATTCTATTTTCGATGGACAAGCTAATAGACGAATTATGAATTCGACGA  >Unigene23447_All Similar to AGAP000254-PA [Acyrthosiphon pisum]  TGGTGACATGAGACCTATCGGTGTAATGGGTAAAGTTGTCGGTTCTCTTTGTGCCATTGCCGGTGTATTAACCATTGCGCTTCCAGTTCCCGTTATCGTTTCTAATTTTAACTACTTTTACCATCGAGAAACTGACCAAGAAGACCTACAGTCCAATAATTTGGACCATGTGGGCAGTTGTCCATTTCTTCCAGCCAATCTTGGTGAAACCCGACTACGTTATACATCCTATTCTGATCTCAAGTTAAACTGTTACACCGGTGAAAATGGTCAGCAAATAACTCGACCACCACGCCGACATCAGGCCAAATATGGTTCAAGTGTCGGTGGAATAGCAACTTTAGCTACATCAAGTGGAACTGTTCCTATCAATACAAATCAATCGAGAAAAGGTCAAATTTCCGTTATGTCAACACAATTATGATCCAAATGGAAACTGTGAACAACTGAAAACCCCCTGATCATCATGTTAATTCAATCCACTCTAAGGAAAGGCGGTGGGAAATGAATGAAAAAACGATATTCATTCT  >Unigene11624_All Cacophony [Apis mellifera]  ATTATATTCCAAACATCGCGACAATAAGAGCCTGGATGAAGGAATATCCC  TTGATCGACAATTTTCAGTAAAAGTTCAACGGTAAAGACACTTGTAAACG  CTTTGTCGAAAATTTCAAGCGTATCATTGATCTGTGAATCTTCATTGACC  GGATCTTCACAGGCCAATGCAATTGATGAACAAAAGATGACCACCATAAT  TACAAAATCAAAGTAACGCAGGTTGACCACATAGTGGGCAGCTCGACGGA  TCGGATTAGTTGATGAAAGTACAAACATTGATGAATAAGGTAACATCGGT  TTAGGACCGACGACATCTTCCTCTTC  >Unigene3042_All GH11826 [Drosophila grimshawi]  CCTCTGCGTTTTCCTCGTCATCGTCTTCGGTGTTTTTACTCAAGGTGCTT  TTTCCCTTCTTGCTTTTCGTTGCCTCTCGTCGTCGCTTAACTTCCATAAT  GTGAATTCTCTCCTCTTCCGTTGTTCTTTCTTCGGCAAGTATTACATCCT  CTGCTTTAATTATCCATTCGACGTAACCTTCCAATTCACGTTCTAATTGT  TCTTGCCTTCGCAATCTTAAGAACGATTGTCGATTCTCGACTCTTTCTCT  TTCTTTGGCAAACTCTCCACTTAAGACACCAAGAACTAAATTGAGCATAA  AAAATGAACCCAGAATAATGA  >Unigene14562_All GK16710 [Drosophila willistoni]  AAAAAAAAACCTCTTATTATAAGAATTGTTTCACAAAAATGACTGAAGTA  GACCAAAAGCAAACTAACAGCAGCCCTGTGGGCAATAATGGCATACCCGC  AGTCGAAAATGCTAACACACCATCAGATCAGTCTTCTGTGAAGAAAGAAG  CAGTAGACAACAAAGATACTCAACAACAAGCCAATGGTGATTCTGCTCCA  CCTCCAGTACCGAAAAAAATTCTTGTCAAAGGTGTTACGGGAACTGTTAA  ATGGTTTAATGTTAAAAATGGTTATGGTTTCATTTCACGAAATGACAAAG  AAAATGAAGATGTTTTCGTTCATCAGACAGCTATTGCCAAGAACAATCCA  AGTAAAGCTGTTCGTAGTGTTGGAGATGGAGAGGAAGTTGAATTTGATAT  TGTCGAAGG  >Unigene23669_All AT rich interactive domain 5B (Mrf1 like), isoform CRA_c [Mus musculus]  GTTGTTGTTGTTGTGGTTGTTGATGATGAACCTTTGATGGTCACTCCTTCTTCGGACATGATTTCCTCTTCATTACGTATCTTTGGGTCACCGGTTGACAATCCATCAATTTTAACAACAAATTTACGCTCTGATTGTGACCTTTTAACATTCGATGAATTAAGATTATTCTCAACGCTTGGACTCCGTGATTTACATTTAGAATTTGAACTCTTTCCTTTTCTTCTCTTTCTTTTTCGTCTTCTTCCACCTTCTCCTCTATCATCTGCCTCATTATCACAAGCTTCATCTTCTTCTGTCATTGAATCACTTTGATTGTCTTTACTTTTATTTGTACCATCATTATCACCACCATCATCATCATCATTGTTTTCAGCACCATTATTATCATGACTCTTTGAAGATGATGAAGACAAAGAGTGACTCTCTTCATCTCCACCAGCACCAAGTTTACTTGAACGCTCCTTAACTGATGATCGACTATTATTAGTATTATTTTTAGTACTATTTTCAATATCACCTTCACCCTCTTCCTTAATTGTATCATCTTCTTGTGACATTGGTGAATAACATTCATCATCATCGGTAGCGGTTGATGAAGCATTAGAACCATAAGCTGACCCACCAGCGGTAACTGACGATGAATTATTATTACCAACACCACCACCAGTTGATGATGAAATTGAGGCGACACCTTTTAAACGTTTAGATTGTCGTACACCTTGACGGCGGTGAGAACCTCGATGAGATCGACGTTCGGTTGAGTTGACACTTCCAACCGATTGATAATCGTCATCACCTCCTCCTCCTTTGGTAGCTGGTGATCCACCAGGAAGTATTGGAGATATTGATCTACCTCGAGTTGATCTTCTGGTCACTACAAGTCCTCTTGCCTCAGCTAAATGATTCTCAAAGGGTAATAATAACCTTTCAAAATGTCGCCGTGTACAAGTTGCCGCACTGGTACTTCGAGGATCACCACCAAGTTCATCATAAACTTTTTTCCATTCCTTATTCTGAGTAATCTTCTCATAACCACCCAATTTCTGGGTAAAGGTAAAAAGGTAAAATAGGTCAATTTTCTTAAATCCAAGATGTGGTATCCTTGTTATGGGTGTGTTCCTTTCTTTCATAAATTTATTCAATTGTTTCATAAATTCTGATTCATTTTTGAGACATTTTTCCGCCAATTTAAGTGACATTGCTGGTGGTTTTGACTTGTGTTTATTGGTTGTTGTGGTTGTATTCCCTTGGGAGG  >Unigene3730_All Similar to AT rich interactive domain 5B (MRF1-like) [Nasonia vitripennis]  TTAGTAGTAGTCTTATTGTTGGCAGCAGAAGAAGAGCCATTTGAATGATT  ATTATTATGATGATTTGCACCACGTTTTCTAGGACGGCCTTTTAATTTGG  GAGCTAAGTGTTCACAGTTAAGATCATGATGTAAAAGTCCTTCATGTTCA  AATGTTTCTCGACAAAAAAGGATCCGGATACAGGGTTTTAATATTGGTGT  CACTGTAGATGATACACTTTTTGTCTCCTTAATATCAACATTTGATTCAC  TTGATGATGGTGATTCTTTGGTATCATTAACATTGTGATTTATACTATTA  TTATTACTATTAATGTGTAAATTTGATTGACAATTTACTGATGTTGAAAT  ATTTTCTTTCTCTTTAATATTTTCTTCCATTTTCTTGAGAACAGCTCGAT  ATCGACAATATTGTGGATATGAAAGGACAACGATGACTGATTGATTCCCT  ATTCGGGAATCAATAACCCCTATTAAATCATTTTTGTTGTTATTTGGTTG  TGATGACGATGAAGATGATGAAGATGGTGATGATGAGGTTAAAGTTGCTG  CTGATGATGATGAACAAGTTGATTGCTGTTGATCCCCTGGTAATTGTTTG  TTAACCTTATCTTCAACATTAATTGTATCAATATTTTTGCTGTTAATTTC  AATATTATGTTCATTTGATTTCTCATTTGATTGTTGTTGGTGGTTTTTTG  GGTGTTGTTGTTGTTGTTGTTGCTGAGGGTTACTGTTATCATGCCCTAAG  GGTATTTGCTTATTAGCATCATTAATTAAATTAAGAAAGGGTAATGTAAA  GGCAGGGGGATCAATATGAATTGCATGATAAGGCACTTCAAGACCGGTAA  CCCAATCAACCTCTTCGGTCATCCATGAGACCAGGTCATTTAATCGTAGG  ATCACAGGTTCCGATGCTGCAAGAACCTCGTGCTGACCATGATTAGATTG  TCTTCCATCGGGTGTTTGCTCAGGTAGGAAATATAATCGAACCGAAGCTA  ACATGGTATCTCTGTATTTATCTTTCCAAATGGACACAAGTTCACCAATA  CAAGGATCATCATGGCGACTAATCCGTACAAAGAAAAATTGATTCACTTT  AATCCGTTTGGTCACATTAATATGATTACTATTAACATTATTATTATTTG  TTGTTGTTG  >Unigene31744_All Superoxide dismutase [Cervus elaphus]  TTTCGTTTGTTATCCTTCTTCTTCACTTTTTTTCCCTCTCTCCTGTTTATTAGAAGAGAGATTTTATTTTGTTTATTAGAAGAAAAGAGAAAAAAATGGTCACAAAAACAATCGTTATTTTCTTTGTTTTCCTTTCTTCTTTCTGCTATGTTACACAGGCATTGAAAGCAACCGCTATTCTCTATGGTAATGGCAACGGAGTTCGCATGGGTCATGTTAACTTTGAAACTCAAGGTAACGGTATTCTTGTCACTGGTCACGTGAGTGGTTTAAAGGAAGGTCAGCATGGATTCCATATCCATGAATTGGGTAATATAACTGATTGTACAGCAACCGGAGGACATTTTAATCCATTGAAAAAGAATCATGGCGCGCCCACTGATACCGATCGTCATGTTGGAGATCTTGGTAATATAAGCGCGGGTTCAAACGGCTGTGCTGAGA  >Unigene28827_All TFG beta signaling pathway factor [Pinctada fucata]  GTTCGTCCTCTAGATGTTGAACAAATATCTTATATAGAACCTGCTTTTTGGTGTTCAATTGCTTATTATGAACACAAGAATCGTGTTGGCGAAATCTTTCACGCTTCAATGCCAAGTTTAACTGTTGATGGTTTTACAGATCCTTCAAGTCCAGAGAGATTTTGTCTCGGTTTACTCTCTAATATAAATAGAACAGCGCCTACCGAGC  >Unigene12646_All Thymidylate synthase-like [Ailuropoda melanoleuca]  GGGATAATAAAAGTTCCATAAGAAAATCACAAAGCCATGGGCATTTCTAT  CTTTGGATATGGTTTGTAATTGATTAGCTCGAAATCTTCAGCTTTGAAAT  CATCAATTGTTTCAACTTTTCTTTTAATTACCAATTTAGGAAATGGTCTT  GGTTCTCTGGTTAGCTGAACTTCAAGAGCATCAACATGATTTAAGTAAAT  ATGAGCATCTCCAATTTGATGAATAAATTCACCGGGTTTCAGATTCGTAA  TATGAGCAACCATGTGAGTTAGAAGAGCGTAACTGGCAATGTTAAATGGA  ACACCAAGACCCATATCTCCAGATCTTTGATCAAGTCGACAAGAAAGTTT  ACCATCAGCAACATAGAATTGAAATAGACAATGACATGGTGGTAGAGCCA  TTTTTGGAATATCAGAAACATTCCATGAACAGACAATTAACCTTCGGTCG  TTTGGATTAGTTTTAATTCTCTCAATAATTTGGGACAATTGGTCAATCCC  TTTACCCGAATAATCGGTGTTTGTGTCAACATAATGAGCACCCCAGTGTC  TCCATTGATGACCATAAACTGGACCTAAATCGCCTTCATCTCGATCGGTG  AAACCAATAGAATCAAGAAATGTTCTTGAACCATTTGCATCCCAAATGTG  AACACCTTTCTGGGCCAATTCTTTTGAATTAGTGTTCCCCTGAATAAACC  AAAGTAATTCTTCAACAAT  >Unigene13685_All Dual oxidase 1 [Lytechinus variegatus]  AAAATTGAGTAATGAAGATGTGAACACAGACCAAATTAGCGATGTCCTTT  TCCTCGAGGTCTCGAAGTATATCAACCAACCATTCAAAATGCTTCTGAGT  TCTAGTTACCCATAGGAAGTAAACCTTGTGGCAAGTTTGCATGCGATTAA  TTGAAGCCCTAAAGGCAACGTCTTTCAATATGGAGGCAAAAGGTGTAACT  CCAATTCCCCCACCGACCAGAACAGAAACTGGAAATCGAAACCAATCTTG  GTGAGTTTCGCCATAAGGTCCATCTAATCTTATATTTGGATAAATGGGTG  ACATTCTCAGGCCATTTGGGTCATAAATTTGTCGAATATTAGAAGTGAAA  GGTCCAACCGCTCGAATGAATAGTTGAAGATTCTCTTCGTGTGGAGCAGA  GGCTAAAGTGAATGGATGAAATTCATTCGAATTCAGGCCAACACTTGCGA  TTCGAACCCATTGGCCACTTTTATATTCAAAGTTCTCCGGCTTAGCTAAA  CAGAGCATCGTG  >Unigene1503_All Adiponectin receptor protein 2-like [Pongo abelii]  AAATAACAACAACCGTGAAAGTTTAAGGTCAAGAAGACGTCGATTTATTA  ATTACAATGAATTACCCGATTGGTTGAAAGATAATGACCTGGTCACTTCT  GGTTATCGTCCAGCGAATCAGTCTTTTTGGTCTTGTTTAGTTTCATTGGG  TTATCTTCACAATGAAACCATTTCGATTTGGTCTCATCTTTTGGCCGCAA  TTTATTTCCTCACCTTGTATGTAGGTCAATATAACGGTACAAATAATTGT  TCAGCGCTCAATAATGACGATCTTTTCCTGCGACTTTACCATTTATCGGC  C  >Unigene13112_All GI17112 [Drosophila mojavensis]  AAAAAGCGAGAAATGGTTGTCAAGTTGATGTGATTTCTTTTATTTATGTG  AGCAATTTATTTTGATTCCGAATTAATTAATCATCATTGTGATCATTATG  TGTGCTGAAAGTTAAAGGTGAAAGTTAGTTTATTACCAAAAGGGAAATGG  AATCAGTAACAACCGATACCTTAAGGACTCTGGTGAATCCTGTGGTCTCG  GCACCACCAGAAACACCACCGATAACAGTTGAGGAATGGCAACATCGATT  CAAAGTTGTAAAAATCGCTTCCAAAGAACCAATGAAACGCGGTAGATGGT  CATGTATGGACTTTAATGATCCTCCATCCAATATTTCGAGTTCAATAATG  TCTGGTAATACCTCTAATAGCACCACAACCACCGTGGGAACTTCAGTAAA  CACCATTCAAAGCACCGGAACCATTGGACAAACTGTTGTCACAGGAACAC  CATTAGCTCCAGGTACCAGTGGGATTACAGGTTCTTGTCTAACAACTGCT  ACTACATGTGTAACAATTAGTAGTGGAATTAATTGTGCCGATTCAAGTAA  TGAAGATAATCAAATTAACAGTGAGATGATTGGACAAGCAACAAATAACC  AACCTGTTTGTATTGGAGTGAGTGGAGTTGGTTTAACAAATATTGATTCT  AATACGATTCCAGTGATTAATCAAAACACTGAAGGTATTACGGTGGATGA  AAAGACTTTTGATCATTTGATGTCCGCTGTCAGAGAGGAAGTTGTTGTCC  TCAAGGAGAGAATCAATGAATTAACGTCAAAAATTACACAATTAGAATTT  GAAAATGGCATTTTAAGGACTCATGCTACTCAAGAAGTGTTAACATACTT  GTATGGATCGAATAACCAAAATGTTTCTAATTGAAATTAATTATTTTCTA  TCAAAATCTACACACACACACACACTCTCCCTGTAGGTGATTCTCTTTCT  CTCTCTCTC  >Unigene19968_All Similar to Sox21b CG32139-PA [Tribolium castaneum]  ATCGGTACCAAGTTCGTTCACAAAGAAGGAGTATGATCGTGTTAAGCGTCCAATGAACGCATTTATGGTCTGGTCTCGAGCTCAACGGCGTCGAATTGCTCTGGAAAATCCAAAGATGCACAATTCGGAAATATCGAAACGCTTAGGTACAAAATGGAAGCATCTAAGTGAAAATNNNNNNGCGTCCATTTATCGAAGAGGCAAAACACCTTCGAGCCCTTCACATGAAACAATATCCCGACTACAAATATAAACCTCGACGTAAACCAAAGCCAATGATGACCAAACAACCATGTCCAATGCCATACTTACAAACTCCGCTTGACCTTCTAGGTTTCCATAGGTCATTTTCCCATTCCCTTCATCTTCACCAAGCACATCAAGCGCACCAGGCACACCATGGTAACCCGGTCTTACACCAAGCGGCAACTGGACTTTCATCAATGATACCTCCTCCCTTACCACCCTCACCAACCTCACAATCACCAACATCATCAACCTCATCCAATACCAAC  >Unigene26396_All Similar to Sox21b CG32139-PA [Tribolium castaneum]  ATCGGTACCAAGTTCGTTCACAAAGAAGGAGTATGATCGTGTTAAGCGTCCAATGAACGCATTTATGGTCTGGTCTCGAGCTCAACGGCGTCGAATTGCTCTGGAAAATCCAAAGATGCACAATTNNNNNNNNNNNNNNNNNNNNNNNNNNNNNNNNNNNNNNNAAGCGTCCATTTATCGAAGAGGCAAAACACCTTCGAGCCCTTCACATGAAACAATATCCCGACTACAAATATAAACCTCGACGTAAACCAAAGCCAATGATGACCAAACAACCATGTCCAATGCCATACTTACAAACTCCGCTTGACCTTCTAGGTTTCCATAGGTCATTTTCCCATTCCCTTCATCTTCACCAAGCACATCAAGCGCACCAGGCACACCATGGTAACCCGGTCTTACACCAAGCGGCAACTGGACTTTCATCAATGATACCTCCTCCCTTACCACCCTCACCAACCTCACAATCACCAACATCATCAACCTCATCCAATACCAACA  >Unigene29176_All Independent phosphoglycerate mutase isoform 1 [Brugia malayi]  CGTCCATTAACGAATTTGATAACATTATCAACAGTTGTTTTATCACCGGTACCTTTAACTAAACCATCATATGCAATTTGTATACGTTCCCAACGTTTATCACGATCCATAGCATAATAACGTCCCATGATTGTAGATAGATTACCATATTTAAGTTCATTGATTTTATCTAAAACAGATTGTAAATAACCGACGCCTGATTTCGGTGTAGTATC  >Unigene12831_All Dynein light chain 2, cytoplasmic [Anoplopoma fimbria]  AAGAATTTGTCTATCTGAGATAGATGATTGATTAATTGGTGAAGAAAATA  CTATCCAGATTTAAAAAGCAATATGGCAACTTGACCCAGGTAAAAGTAAA  TGAAATGTTTAGTTTCATGGGTTACATATGAACCAAAATTACGACCCACA  ATACAATGCCAGGTTGGGTTGTATTTCTTGTCAAACTCCTTCTTTATAAA  AGCAGCAATATCCTTCTCAATATTATACTTGTCCAATGCTTGGATTGATG  TATTGATAGCATCATCTTGCATCTCGTCACTCATGTCGGCATTTTTAACC  ACAGCTTTCTTATCCGTACTCATATTGAACTAATTTTATTTACCGATGAC  ACCACAAAAATGTGACAAATAAATATCACTTTTTTAAACAGTTCTATGGA  TAGAAAACAATTGATAAAATAAAATTATTACCAACAATTAATTGTCAAAA  TTTATAAATCAAATACATGAGGTGAAAAAAACAAGAATCAAG  >Unigene22714_All Blimp-1 [Culex quinquefasciatus]  GTTTATCGTCATCCGAGAAAAAGACTTATCAATGTCAATTTTGTTCCAAGGTATTCGGTCAGATGTCAAATCTTAAGTGTCACGTTCGTACACATACTGGAGAAAGACCGTTTGTTTGTAGAATTTGTCAGAAATCGTTTATTCAATTAGCTCATTTACAAAAACATGAGCTCGTTCATACCGGCGAAAAGCCTTTCCAGTGTGACCTGTGTCTAAAACGATTCACATCCAAAAGTAATCTTAATAATCATAAATCTCGACACATATACGAACCAAACGAAGCTGACAAC  >Unigene28924_All Putative histone H3.3-like type 3  CGCGTTTACCATTTCAACGTTTGGTACGTGAGATAACACAGGATCTTGCACTTGAAGAAATGAAATTTCAATCAGCGGCATTATCAGCATTACAAGAAGCAGCAGAAGCGTTTATCGTTGGTCTTTTTGAAGATACACAATTGAGTGCCATACACGCCAAAAGAGTGACCATTATGCCAAAAGATATGCAGCTTGTTGTACGAATACGGG  >Unigene31329_All GF23557 [Drosophila ananassae]  CTTCTTTTTATTTCGTGAAATTATATCTATATTATATTGTAGTGAATTGAATTAATATCGCTATTTTAAATGATGGTCTTAGCACGTAACAAACACAACAAAATCAAGGACTGTGCTGAGAAACCGCAGAAACCTCCACGTACTTACGTTGATATGATCACAGAAGCCATTTTGTCAACTACAGATGGGAAAGCAACCCTTCAAGATATTTACAGGCATTTACAAAGGGTTGAACCTGAATATTTCATGGGGCAATATACTGGTTGGACAAATTCAATACGCCACAATCTATCATTGAGTCCATGTTTTATTAAGTTAGAGAAGAAAGAGAACGGCC  >Unigene31513_All 60S ribosomal protein L44  CTGAATTAAAATCGATTTAAACGGTTCTCCATTTGAACGGGTTTCGATTGTAATTAAATAAATTGATTAGTCCTTTGAGAAATTGAAGTAAATAAATATAGTAATGGTTAACGTACCGAAAGCAAGACGCACTCTATGTGCCGGTAAATGTGCTAAGCATACCGTTCACCGTGTGAGTCAATACAAAAAAGGTAAAGCCCGTAAATCAGCTCTTGGTGCCCGTCGTTACGCTCGTAAATGTCGTGGTTTTGGTGGTCAGCCCAAACCTATTTTCCGAAAGAAAGCTAAAACAACAAAGAAAATTCTTCTCCGTCTCGAGTGCGTCGACTGCAAACACAGACATCAACTTTCAATTAAACGTTGTAAGCATTTG  >Unigene31732_All Nuclear migration protein nudC-like [Saccoglossus kowalevskii]  TTTAATTAACTAACCAAAGCTATCAAAATGAGTAAAGACGAAAAATTTGATGCTATTTTATTGTCGATGGCACAGCAGCTTGAAGGCGGGATCGGCGAGTTTATTGATACAATATTCAGCTTTTTGTGTCGTAAAACTGACTTTTATATCGGCGCTGGTAAAGATGATGCACGTAAAATGGTTGTCAGTACTTTTGATAGATGGGAAAAACGTGCTCTTGAAGTGCATAATGAAAAAGAACGAGAATCAATCGCTGCAGAGTCGAGAAAAAAACAACTTGAATTAGAACGTCAACGAAGGAAGAAATTGGAAGAAGAAAATGAGCCACAAGTTGTTGAAATTTCTGATGATCAAATCGAAGCAGCCGAAAAGAAGATTGCCCAAGAAAAAGCATCCGGATCGTCGAAAGATAAAGACGAAGAAGAAAGTGAAGAAGAC  >Unigene3967_All SUMO-1-like protein [Artemia franciscana]  GAGAAGAAAAAGAAAAAGAAAAGGAAAGAAAAAGGAAAGGAAAAAAGGAA  AAAGCGAAGAAAAAGAGAAAAGAGAAGAAAATAGATAAAGATTGAGATTG  AGAGGTAAGAGAAGGTGACGCTTTTTTCTTTGAACATGGAATCACGAATC  TTTTGTTTTGTCTATTCTTTTCTTTTAGCAGCCAAAAAATATGTTGCCTC  CAAAGCCTCCAGATTGTTCTTGGTAAACCTCAATAACATCGTCATTTTCC  ATTTCCAATTGCTTAGGAGTATCTGTATCATTGATACGGCGACCATCAAA  TAAGAACCTCAAAGAGGTAACTGGTACACCCACTCGTTCACTGTAAGATT  TTTTCAATTTTCCCATTGCTGTGGTCATTTTAACTCTAAAATGAATCTCA  TTTGAATCTTGACCGACAACCTTAAGTTTAATATACTCAGAATTGGCATC  ACCACCTCCTTCAACACCGGATTTATTATCTTTAGGTGATTCCATAGTTC  AAAAAAGGGTAAGAATTTAATTAGTCAAAAGACAAT  >Unigene13915_All FGFR2 protein [Bos taurus]  TGCCGATTCGTTTCATGACCTCCATTTCCGAGACAAGATCAGCCATGTCA  GAATCATTCGATGGATCTTTCAACATTTTAACAGCCACCGTCATAAGAGG  TTCTTTATTTTTTCCAACTCCACATAGCTCTCCTTTATAAACAGTTCCAA  AGTTTCCTTGTCCAACAGGCTTATGTAATTGTAATCTATCTCTTGATATT  TCCCATTTAAGATCAATCGGCATTTCATATTCAACAATGGCTCTCATATA  TTTCTCCACAGGTAAATCGTTAGCATTCAATGGAGCCTCAATTTTGACCA  AAGGCGCTTTCAATGAATCTTCACAGTCAACAGGCTCTTCTAATGTGATC  TTTTTTGTTATCCTTAGAATTTGATTTTCTTTCTTTATTTTTAATCGCGA  GGCTCGAATTTTACAGAGACAACATATCAGGCAAAGTACTATTGTTGTCG  CACTGCCAATAATTAGAGATCCAATGAAAATAACTTGCTCACGAGTTAAA  CTGTTTGCAGCTAATTTATGTTCTTGATGATGGACTCTAAGAAATATATT  AGCTGAACGGAAGCCAAAACGATTTCGAGAGACATATGAATACCATCCTT  GTACATCTTGTGTCACATTCTTCAAAACCAAGTCGCTTGTTCCTTTTTCA  ATTATATCAGTATCGTTAAAATATTCACTTGACTCATTGATAAATCCTTT  GATCCAAAATTTTTCATCTTCAANNNNNNNNNNNNNNNNNNNNNNNNNNT  GAGAGCTGAGATATTATAAGACCAACAAGTTCCTATTTGAGGAGGCATAG  GTTCATTAACGGTGACATTAAACAGCCTTTCACTTGTAGCTATCGAATTG  CTAACTTCACAACGAAATAATCCTGAGTCCTCGATTGTAATATTTGGCCA  TATAAATATATTAGTTTCACCATCCAAACCTTCATATTCAATCCATTCAT  CTCCAACTTTTTTATACCAATAAGTGCTCTCAGCAGCATCTGATCCTGAT  TCACAATTTAATTGTAAATCATCACCAACATACAATGAACGTTGCGATAT  CTCATCATTGATTACATGAGGTGCGATCCATTCACTAACGTTCAATTGAA  ACCATATAGAATCGTTACCATAATCGTTGATAGCTAAACATGAATAATTT  CCAGCATCGATTGGAGTTAAACGAGCGAAATCNNNNNNNNNNNNNNNNNN  NNNNNNNNNTAATATCATTTCTGTCTAATTGTTGACCATCTTTGAACCAG  GTTATATTGGGACAAGGATTTCCACTGGATTTGCATTTGAANNNNNNNNN  NNNNNNNNNNNNNNNNNNNNNNNNNNNNNNNNNNNNNNNNNGAATAAATG  GCGCTGATGGAAGTAGGTCTGGTCTATTTTGACATTTAGCACAATAGACA  AGATTAGGCACAAAGAAAATGATAATAATCGTACAGAGTAACAGTAAACT  ATTATTACACAGATGATACATTGTTTGTTATTGTTGTTGTCACTTGTTTA  AATTAAAAAGATATTAATTAATTATGTTGTAATAGTTAGTAG |
